# Integrating molecular, biochemical, and immunohistochemical features as predictors of hepatocellular carcinoma drug response using machine-learning algorithms

**DOI:** 10.3389/fmolb.2024.1430794

**Published:** 2024-10-16

**Authors:** Marwa Matboli, Hiba S. Al-Amodi, Abdelrahman Khaled, Radwa Khaled, Marwa Ali, Hala F. M. Kamel, Manal S. Abd EL Hamid, Hind A. ELsawi, Eman K. Habib, Ibrahim Youssef

**Affiliations:** ^1^ Medical Biochemistry and Molecular Biology Department, Faculty of Medicine, Ain Shams University, Cairo, Egypt; ^2^ Faculty of Oral and Dental Medicine, Misr International University (MIU), Cairo, Egypt; ^3^ Biochemistry Department, Faculty of Medicine, Umm Al-Qura University, Makkah, Saudi Arabia; ^4^ Bioinformatics Group, Center of Informatics Sciences (CIS), School of Information Technology and Computer Sciences, Nile University, Giza, Egypt; ^5^ Biotechnology/Biomolecular Chemistry Department, Faculty of Science, Cairo University, Giza, Egypt; ^6^ Physiology Department, Faculty of Medicine, Ain Shams University, Cairo, Egypt; ^7^ Department of Internal Medicine, Badr University in Cairo, Badr, Egypt; ^8^ Department of Anatomy and Cell Biology, Faculty of Medicine, Ain Shams University, Cairo, Egypt; ^9^ Department of Anatomy and Cell Biology, Faculty of Medicine, Galala University, Suez, Egypt; ^10^ Systems and Biomedical Engineering Department, Faculty of Engineering, Cairo University, Giza, Egypt

**Keywords:** hepatocellular carcinoma, drug response, predictive biomarkers, machine learning, rats

## Abstract

**Introduction:**

Liver cancer, particularly Hepatocellular carcinoma (HCC), remains a significant global health concern due to its high prevalence and heterogeneous nature. Despite the existence of approved drugs for HCC treatment, the scarcity of predictive biomarkers limits their effective utilization. Integrating diverse data types to revolutionize drug response prediction, ultimately enabling personalized HCC management.

**Method:**

In this study, we developed multiple supervised machine learning models to predict treatment response. These models utilized classifiers such as logistic regression (LR), k-nearest neighbors (kNN), neural networks (NN), support vector machines (SVM), and random forests (RF) using a comprehensive set of molecular, biochemical, and immunohistochemical features as targets of three drugs: Pantoprazole, Cyanidin 3-glycoside (Cyan), and Hesperidin. A set of performance metrics for the complete and reduced models were reported including accuracy, precision, recall (sensitivity), specificity, and the Matthews Correlation Coefficient (MCC).

**Results and Discussion:**

Notably, (NN) achieved the best prediction accuracy where the combined model using molecular and biochemical features exhibited exceptional predictive power, achieving solid accuracy of 0.9693 ∓ 0.0105 and average area under the ROC curve (AUC) of 0.94 ∓ 0.06 coming from three cross-validation iterations. Also, found seven molecular features, seven biochemical features, and one immunohistochemistry feature as promising biomarkers of treatment response. This comprehensive method has the potential to significantly advance personalized HCC therapy by allowing for more precise drug response estimation and assisting in the identification of effective treatment strategies.

## 1 Introduction

Hepatocellular carcinoma (HCC) is a deadly form of liver cancer, ranking fifth among the most commonly diagnosed malignancies worldwide and being the third leading cause of cancer-related deaths (Sung et al., 2021). In Egypt specifically, HCC remains the most prevalent cancer and the primary cause of cancer-related mortality, resulting in approximately 26,000 deaths annually (Omar et al., 2023). HCC arises due to repeated cycles of inflammation, leading to the progression of fibrosis, cirrhosis, and ultimately, the development of cancer (Piñero et al., 2020). Patients with chronic liver conditions, such as viral hepatitis, and even those with alcoholic and nonalcoholic fatty liver disease or metabolic syndromes, are at a higher risk of developing HCC (Chidambaranathan-Reghupaty et al., 2021). Unfortunately, the lack of effective biomarkers for early detection, limited understanding of HCC’s heterogeneity, and therapeutic resistance contribute to the high mortality rates (Tunissiolli et al., 2017). Despite the availability of various treatment options such as surgical resection, targeted immune therapies, and radiofrequency ablation, patients with unresectable HCC face significant unmet medical needs and experience a poor prognosis (Xing et al., 2021; Llovet et al., 2022).

Numerous RNA biomarker subtypes, such as Circular RNAs (circRNAs), microRNAs (miRNAs), and long noncoding RNAs (lncRNAs), have demonstrated their potential as diagnostic and prognostic biomarkers in HCC (Tan et al., 2019; Jiang et al., 2019). Particularly, these biomarkers are involved in many HCC-related pathways such as autophagy, apoptosis, cell cycle regulation, and immune checkpoints (Takamura et al., 2011; Lee et al., 2018). CircRNAs, predominantly located in the cytoplasm and exhibiting relative stability, function as miRNA sponges or protein scaffolds, thereby facilitating protein-protein interactions (Shi et al., 2021). They play a significant role in the initiation and progression of HCC (Su et al., 2019). Dysregulation of lncRNA has been observed in various cancer types, where it can either suppress or promote tumorigenesis and tumor development (Kang et al., 2019). LncRNA exerts its influence through interactions with DNA, RNA, and proteins, as well as by acting as microRNA sponges (Huang et al., 2020). Moreover, they play a crucial role in tumor progression and contribute to the malignant phenotypes observed in HCC, including proliferation, invasion, and migration (Unfried et al., 2021). Considering the complexity of the RNA landscape, which involves various splicing forms, alternative polyadenylation, and chimeric RNAs, the incorporation of multiple RNA types could enhance the performance of diagnostic and prognostic panels (Eun et al., 2023; Fu et al., 2022; Schlosser et al., 2022).

RNA extracted from malignant growths has great potential as a valuable resource for determining drug efficacy in various cancer forms (Fang et al., 2023). Despite previous endeavors falling short of desired outcomes for clinical implementation, there remains a persistent interest in exploring novel avenues (Zhang H. Y. et al., 2023; Zhang J. et al., 2023). Presently, the technique of immunohistochemistry (IHC) has emerged as a promising tool for assessing the presence of HCC such as glutathione S-transferase pi (GSTP), proliferating cell nuclear antigen (PCNA), and tumor necrosis factor (TNF), indicating that integrating protein expression data with mRNA may offer a more effective means of predicting drug response (Márquez-Quiroga et al., 2022; Stärkel et al., 2015; Feng et al., 2022).

Our research endeavors were extensively conducted on animal models, aiming to identify effective drugs for HCC. During our studies, we explored diverse therapeutic approaches, including the utilization of proton-pump inhibitors (PPIs) such as Pantoprazole. Pantoprazole has garnered attention as a promising therapeutic strategy for gastric cancer, showing the ability to enhance sensitivity to antitumor drugs, attenuate liver tumorigenesis, and modulate autophagy in rat models (Zhang et al., 2019; Bridoux et al., 2022; Kim et al., 2022; Matboli et al., 2019). Furthermore, our investigations delved into the anticancer effects of natural compounds, with a specific focus on triggering cancer cell death (Zaghloul et al., 2017; Kooti et al., 2017). Hesperidin, a flavanone glycoside commonly found in citrus fruits, emerged as a prominent candidate as it exhibited anti-inflammatory, anti-carcinogenic, antioxidative, and lipid-lowering effects (Banjerdpongchai et al., 2016). Moreover, it has demonstrated an antigenotoxic effect by counteracting DNA damage induced by hydrogen peroxide (Fernández-Bedmar et al., 2017). Importantly, hesperidin has exhibited inhibitory effects on the development of various cancer types, including tongue, esophageal, and colon cancers (Tanaka and Sugie, 2007; Aggarwal et al., 2020). Additionally, Cyanidin 3-glycoside (Cyan), a notable anthocyanidin found in plants and fruits, has been associated with multiple beneficial properties, such as anti-aging, anti-oxidative, anti-inflammatory, vascular relaxation, antiproliferative, and anti-inflammatory effects (Safdar et al., 2023; Mirmalek et al., 2023). Moreover, Cyan has suppressed the progression of breast, lung, and liver cancers (Yin et al., 2019; Chen et al., 2021). To gain comprehensive insights, we employed a panel of mRNA signatures involved in HCC pathogenesis and their epigenetic regulators complemented by biochemical, histological, and immunohistochemistry analyses, these comprehensive evaluations provided valuable indications of the biological actions and potential clinical benefits of our therapeutic candidates.

Recently, the advent of artificial intelligence (AI) has enhanced all possible aspects of clinical care for HCC. AI holds the potential to revolutionize HCC management by addressing key challenges in several crucial areas by enhancing the prediction of future HCC risk in patients, improving the accuracy of diagnosis, and refining prognostication for patients already diagnosed with HCC while predicting their response to specific drugs (Calderaro et al., 2022). The primary objective of our study is to develop a machine-learning (ML) model that utilizes a comprehensive set of molecular, biochemical, and IHC features derived from HCC rat models in order to predict drug response.

## 2 Materials and methods

### 2.1 Chemicals and drugs

Diethylnitrosamine (DEN) with a purity of 99.0% and acetamidofluorene (2-AAF) with a purity of 98% were acquired from Sigma Aldrich (St. Louis, MO, United States) with CAS numbers 55-18-5 and 53-96-3, respectively. Hesperidin was also obtained from Sigma Aldrich, while Cyan was purchased from (Earth Natural Supplements in Colorado Springs, United States). Pantoprazole was supplied from (Controloc; Takeda GmbH, Oranienburg, Germany).

### 2.2 Experimental protocol

**FIGURE 1:**
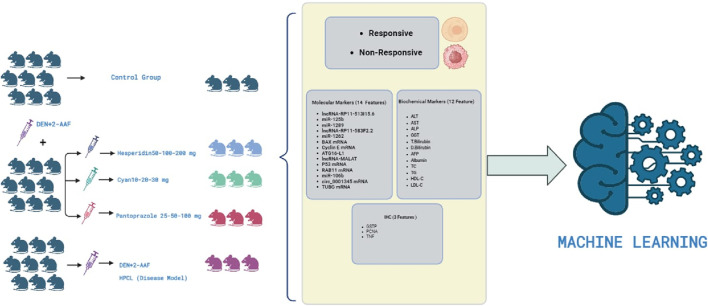
The chart summarizes the experimental design of the study.

One hundred and thirty male Wistar rats, weighing between 190 and 200 g, were procured from the animal house of Nile Pharmaceuticals in Cairo, Egypt. The rats were housed in a controlled environment with temperatures maintained at 22°C–24°C, following a twelve-hour light-dark cycle. They were provided with standard rat chow and had access to tap water, allowing them to acclimate for 1 week. Ethical approval was obtained from the Ain Shams Research Ethics Committee, Faculty of Medicine, Egypt, FMASU MD 32/2016. In accordance with the guidelines of the Declaration of Helsinki. Before each injection, the weight of each rat was measured to ensure accurate calculation of the drug dosage. To induce hepatic pre-cancerous legions (HPCL), the rats were intraperitoneally injected with DEN+2‐AAF. DEN was administered intraperitoneally once a week for three consecutive weeks at a dose of 100 mg/kg, followed by a one-week rest period. Subsequently, 2‐AAF was injected intraperitoneally at a dose of 300 mg/kg (Hasanin et al., 2021a). The rats were then divided randomly into eleven groups: I) The control group (10 rats) received intraperitoneal injections of 0.9% NaCl. II) The HPCL group (12 rats) received intraperitoneal injections of DEN+2‐AAF. III) The Hesperidin groups (12 rats each) included Hesperidin-50, Hesperidin-100, and Hesperidin-200. These rats were injected with DEN+2‐AAF for HPCL induction and then treated with hesperidin at doses of 50, 100, and 200 mg/kg/day, respectively, for four consecutive days weekly for 16 weeks. IV) The Cyan groups (12 rats each) included Cyan-10, Cyan-20, and Cyan-30. They were injected with DEN+2‐AAF for PCL induction and subsequently treated with Cyan at doses of 10, 20, and 30 mg/kg/day, respectively, for four consecutive days weekly for 16 weeks. V) The Pantoprazole groups (12 rats each) included Pantoprazole-25, Pantoprazole-50, and Pantoprazole-100. These rats were injected with DEN+2‐AAF for HPCL induction and then treated with Pantoprazole intraperitoneally at doses of 25, 50, and 100 mg/kg/day, respectively, for three consecutive days weekly for 4 weeks (Figure 1).

### 2.3 Blood sample and liver tissue assessment

At the end of the experiment, the rats were anesthetized with urethane (1.2 g/kg; intraperitoneal injection) dissolved in distilled water. The blood was obtained from the retroorbital vein and incubated for approximately 30 min to allow clotting. Subsequently, the samples were centrifuged at 5,000 rpm for 20 min to separate the serum. The livers were carefully dissected. Specifically, the right lobe of each liver was removed and sliced into longitudinal sections measuring 2–4 mm in thickness. These sections were preserved in 10% formalin for the histopathological and immunohistochemical examinations. The remaining liver lobes and sera samples were promptly frozen at −80°C for liver function tests and RNA extraction.

### 2.4 Biochemical analysis

Quantitative analysis of serum biomarkers was conducted to assess various parameters including alanine transaminase (ALT), aspartate transaminase (AST), total cholesterol (TC), triglycerides (TG), HDL cholesterol (HDL-C), and LDL cholesterol (LDL-C), alkaline phosphatase (ALP), gamma-glutamyl transferase (GGT), total bilirubin (T. Bilirubin), direct bilirubin (D. Bilirubin), albumin using commercially available kits and an automated Beckman Coulter AU680 autoanalyzer (Beckman Coulter Inc., CA). Additionally, the levels of alpha-fetoprotein (AFP) were determined using an ELISA kit (Abcam cat no. ab108838, Cambridge, MA, United States).

### 2.5 Molecular markers selection

We used the Gene Expression Omnibus (GEO) database (https://www.ncbi.nlm.nih.gov/gds/?term=) to find differentially expressed genes (DEGs) using the keyword “Hepatocellular Carcinoma,” We collected datasets that fulfilled our criteria, which involved having tissue samples related to hepatocellular carcinoma (HCC) along with normal tissue samples for comparison. Moreover, we verified that each dataset had an adequate sample size to ensure the reliability of our statistical analysis, and chose the GSE141090, GSE24600, GSE38199, GSE49515, and GSE41804 datasets (details in Supplementary Table S2), then we applied GEO2R/Limma R package tool to find significant DEGs, the *P* values less than 0.05 were considered significant with log twofold change (LogFC) value ≥ 1 or ≤ − 1, using the adjustment method of Benjamini and Hochberg (false discovery rate) (Supplementary Table S1). Based on our interest in cell cycle progression, autophagy, metastasis, and apoptosis, we selected BCL2 Associated X (*BAX*), Autophagy Related 16 Like 1 (*ATG16-L1*), *Cyclin E* mRNA, Tumor Protein P53 (*P53*), Ras-related in brain11 gene (*RAB11*), and Tubulin gamma 1 (*TUBG1*). The expression ATLAS (https://www.ebi.ac.uk/gxa/home) in addition to The Cancer Proteome Atlas (TCPA) (available at https://www.tcpaportal.org/) databases were used to confirm the differential expression of the selected genes in HCC (Supplementary Figure S2). Using the Kyoto Encyclopedia of Genes and Genomes (KEGG) and the Reactome databases we performed pathway analysis for the selected genes using the Enricher database (https://maayanlab.cloud/Enrichr/). Then we selected the epigenetic regulators of the DEGS, firstly, we retrieved the miRNAs (miR-125b-1-3p, miR‐1262, miR‐1298, and miR-106b) that interact with the selected DEGs using the mirwalk database (available at http://mirwalk.umm.uni-heidelberg.de/) and the RNA22 database (available at https://cm.jefferson.edu/rna22/Interactive/) (Supplementary Figure S3). Moreover, we chose the LncRNAs (lncRNA-RP11-513I15.6, lncRNA-RP11-583F2.2, and lncRNA-MALAT) based on our previous studies on HCC, (Matboli et al., 2019; Hasanin et al., 2020; Zabady et al., 2022; Matboli et al., 2021; Hasanin et al., 2021b) their differential expression in HCC confirmed by the TANTRIC database (available at https://www.tanric.org/), and their interaction with miRNA verified by the RNA22 database (available at https://cm.jefferson.edu/rna22/Interactive/) (Supplementary Figures S4, S5). The hsa_circ_0001345 was chosen based on our previous study that validated its role in autophagy in HCC (Zabady et al., 2022).

### 2.6 Total RNA extraction and real-time PCR

The mRNA expression of *TUBG1*, *RAB11A*, *ATG16-L1*, *BAX*, and *P53*, as well as the lncRNAs lncRNA-RP11-513I15.6, lncRNA-RP11-583F2.2, and lncRNA-MALAT, was determined using the RT (Omar et al., 2023) SYBR Green ROX real-time quantitative polymerase chain reaction (qPCR) Mastermix and Quantitect SYBR Green Mastermix Kit (Qiagen, Düsseldorf, Germany), specific primers provided by Qiagen (QT00176519, PPR42379A, PM00597373, UPFH0540166, UPFR1074538, SBRN-027Z, LPH05247A, LPH24879A, and SBH0655633) were used in conjunction with the Step One Plus™ System (Applied Biosystems Inc., Foster City, CA, United States). β-actin (PM00480207) was utilized as the endogenous control. For miRNA expression analysis in the liver tissue, we followed the miScript primer assay and miScript SYBR Green kit protocol from Qiagen (Düsseldorf, Germany) to investigate the expression of miR-125b-1-3p, miR‐1262, miR‐1298, and miR-106b_1 (YI04102304, SBH0386397, SBR1207111, and SI05465957). The endogenous control used was RNU-6 (SI03956260). To obtain the targeted circRNA junction sequence, we referred to the CircInteractome database, and a custom-designed primer assay from the primer-blast database was used for amplification (Zabady et al., 2022; Dudekula et al., 2016; Ye et al., 2012). The PCR program consisted of an initial denaturation step at 95°C for 15 min, followed by 40 cycles of denaturation at 94°C for 10 s, annealing at 55°C for 30 s, and extension at 70°C for 34 s. Each reaction was performed in duplicate. To quantify the expression of the target molecules, we employed the 2^−ΔΔCt^ method (Livak and Schmittgen, 2001). The expression levels of the target genes were normalized against the housekeeping gene for each sample and compared to a reference sample.

### 2.7 Histological and immunohistochemical examination

Liver samples were obtained from all animal groups and were treated with 10% neutral formaldehyde for 24 h for fixation. The samples were then dehydrated and embedded in paraffin blocks. Thin sections of 5 μm thickness were prepared and subjected to hematoxylin-eosin (H&E) staining to examine potential histopathological alterations. To capture the images, an Olympus BX50 Light microscope from Japan was utilized. Following routine dewaxing with xylene and hydration through a graded ethanol series, the sections underwent a 15-min incubation with a 3% hydrogen peroxide solution at room temperature to neutralize endogenous peroxidase activity. Subsequently, the sections were washed with gently running tap water, followed by rinsing with phosphate-buffered saline (PBS). Primary antibodies were then applied to the sections and incubated overnight at 4°C. The specific antibodies used included a rabbit polyclonal antibody against GST-P (catalog AB106268; Abcam, Cambridge, MA) at a dilution of 1/250, anti-PCNA (Santa Cruz Biotechnology, Santa Cruz, CA) at a dilution of 1/400, and anti-TNF-α (Cat. No. NB600–587, Novus Biologicals, Littleton, CO, United States) at a dilution of 1/200, with incubation conducted at room temperature for 2 h. After rinsing with PBS, the sections were incubated with a biotinylated secondary antibody for 30 min at room temperature. The peroxidase activity was visualized using a 3,3′-Diaminobenzidine solution (Vector Laboratories, Inc., Burlingame, CA), and counterstaining was performed with hematoxylin. To quantify the GST-P-positive area, images were captured using a charge-coupled device (CCD) camera connected to a Windows computer. For PCNA expression level calculation, the number of PCNA-positive hepatocytes was determined by counting 1,000 cells in each case, and the results were presented as a percentage (number of PCNA-positive hepatocytes/1,000 hepatocytes) at × 100 magnification. The area percentage of immunostaining for TNF-α was measured using the Leica Qwin 500 C image analyzer. Measurements were obtained from 10 non-overlapping low-power fields per section in each group.

### 2.8 Statistical analysis

The statistical analysis was conducted using SPSS 26.0 software. Continuous variables were reported as mean ± standard deviation (SD), following the verification of their normal distribution using the Shapiro-Wilk test. One-way analysis of variance (ANOVA) with Tukey *post hoc* test was employed to compare differences between groups. A *p*-value below 0.05 was considered statistically significant. The Chi-square test was employed to compare the categorical data of inflammation grades and fibrosis stages as the grade of 0 indicates no foci, grade 1 signifies less than 2 foci per 200x field of view, grade 2 indicates 2-4 foci per 200x field of view, and grade 3 represents more than 4 foci per 200x field of view. On the other hand, the fibrosis stage is assessed based on the location and extent of fibrotic changes. A stage of 0 indicates no fibrosis, stage 1 represents fibrosis around the sinusoids or portal areas, stage 2 signifies the presence of fibrosis around both the sinusoids and portal/periportal regions, and stage 3 indicates bridging fibrosis (Kleiner et al., 2005).

## 3 Machine learning models

### 3.1 Data

One of the goals of this study is to build a machine-learning predictive model to estimate the treatment response based on a set of observational measurements of the treated samples. Let $X_{i}\in R_{{+}^{n\times p_{i}}}$ be a set of one type of measurements, where this type *i* ∈ {Molecular, Biochemical, IHC} (Table 1), $R_{+}$ is the non-negative real numbers, *n* is the number of samples (Table 2), and *p*_*i*_ is the number of features per type *i* (Table 1), and let $Y\in N^{n\times 2}$ be the treatment response, where N is the non-negative integers. Response Y is determined by two features: inflammation grade and fibrosis stage. Values less than or equal to two for both of the response features indicate a successful treatment. So, a sample will be classified as responsive (binary 1) when both values of the inflammation grade and the fibrosis stage are ≤2 ; otherwise the sample will be labeled unresponsive (binary 0). According to histopathological inflammation scores less than 2 mean no or minimal inflammation (Castro-Gil et al., 2021). Moreover, according to our animal model experiments, the baseline stage (lowest degree) of fibrosis captured in our model was 3. So we considered less than or equal to 2 as an improvement of liver fibrosis (Mak and Mei, 2017).

**TABLE 1:** Features and their number for each data type.

Molecular (14 features)	Biochemical (12 features)	IHC (3 features)
1. lncRNA-RP11-513I15.6	1. ALT	1. GSTP
2. miR-125b	2. AST	2. PCNA
3. miR-1289	3. ALP	3. TNF
4. lncRNA-RP11-583F2.2	4. GGT	
5. miR-1262	5. T. Bilirubin	
6. BAX mRNA	6. D. Bilirubin	
7. Cyclin E mRNA	7. AFP	
8. ATG16-L1	8. Albumin	
9. lncRNA-MALAT	9. TC	
10. P53 mRNA	10. TG	
11. RAB11 mRNA	11. HDL-C	
12. miR-106b	12. LDL-C	
13. circ_0001345		
14. TUBG mRNA		

**TABLE 2:** Number of samples per control, disease model, and treatment drugs.

Condition	Number of samples
Control (Healthy)	35
HPCL (Disease model; untreated)	35
Hesperidin-50	12
Hesperidin-100	12
Hesperidin-200	12
Cyan-10	12
Cyan-20	12
Cyan-30	12
Pantoprazole-25	12
Pantoprazole-50	12
Pantoprazole-100	12

### 3.2 Predictive models

We built several supervised machine learning models using the classifiers logistic regression (LR), k-nearest neighbors (kNN), neural networks (NN), support vector machines (SVM), and random forests (RF) to predict the treatment response. For each classifier, we tested seven models (Table 3), where the main difference among them is the data type of the features used as predictors. Such distinctions in predictors can assess the differential predictive power of these models based on the utilized data type. In addition to the seven models listed in Table 3 per classifier, we constructed another seven corresponding models, which we designated as “reduced” models. We used the greedy forward sequential feature selection (SFS) approach with random forests as the estimator to pick up the smallest set of features that are the most representative of the model. This reduction in the number of features can reduce model complexity and computation time while enhancing model interpretability.

**TABLE 3:** Predictive models according to predictors data type.

Model	Data type
1	Molecular
2	Biochemical
3	IHC
4	Molecular + Biochemical
5	Molecular + IHC
6	Biochemical + IHC
7	Molecular + Biochemical + IHC

The LR classifier had the maximum number of iterations for convergence set to 1,00,000 with a stopping tolerance of 0.0001. The number of neighbors for the kNN classifier was 5 with uniform weights. The NN classification was done using the multi-layer perceptron (MLP) classifier with two hidden layers of a few numbers of neurons (5, 3) to avoid overfitting, the maximum number of convergence iterations to be 200, and the “lbfgs” optimizer as the solver. The parameters for the SVM classifier were the regularization parameter (C) with a value of 1 and a tolerance of 0.0001 for the stopping criterion. The RF classifier had 100 trees with a maximum depth of 10 for each tree. The SFS approach used an RF estimator in a forward direction with parameters of 0.00001 for the stopping criterion and cross-validation of 5 iterations.

### 3.3 Cross-validation and class balancing

We split data into two disjoint, exclusive sets. The first set was used to train the machine learning model, while the second set was used to test the model’s predictive performance. The test set represented unseen samples for the trained model to avoid overfitting. This splitting process was repeated *k* times to perform a *k*-fold cross-validation (kCV) assessment, where every single sample was used once for testing the model’s ability to classify the unseen data. This study contains groups of data representing different treatment drugs, one or more controls, and a disease model (no treatment). We did not follow the strategy of leave-one-group-out to mitigate the problem of under-representing a group which would increase the misclassification rate. Hence, we divided each group into *k* subsets, where at each CV iteration, only one subset is used for testing. To do so, assume that *s*_*i*_ is the index of sample *s* and *i* = {1, 2, 3, … , *n*}, where *n* is the total number of the study samples. The function *mod* (*s*_*i*_,*k*) uses the *mod* operator to get the integer remainder of dividing *s*_*i*_ by *k*, where the output of (*mod* (*s*_*i*_,*k*) + 1) ∈ {1, 2, 3, … , *k*}. So, for the *k*th CV iteration, the samples that give the result (*mod* (*s*_*i*_,*k*) + 1) = *k* will be used for testing the model and all the other left samples will be used for the model learning phase. This approach preserves stratified sampling at each CV iteration.

**FIGURE 2:**
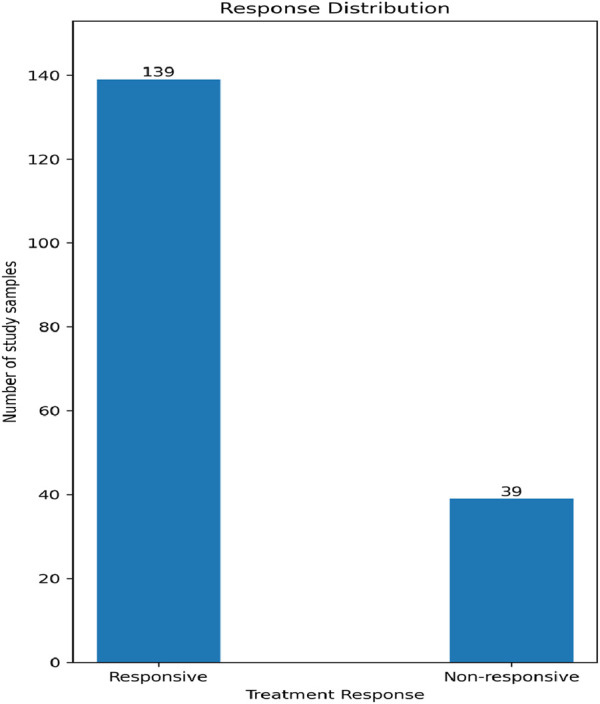
The number of responsive and non-responsive samples.

Our data contains two classes in accordance with the treatment response: non-responsive and responsive, with a relative ratio of 1:3.56, respectively, as shown in Figure 2. We used the Synthetic Minority Oversampling Technique (SMOTE) to sample up the undersampled class to have classes of equal size in order to minimize the classifier bias. The upsampling process was performed only in the training phase, and not on the tested samples.

### 3.4 Identification of potential treatment biomarkers

To study the potential for a certain feature to be a treatment response biomarker for a specific drug, we screened each feature against the control and against the disease model for each drug separately. We used the non-parametric, two-sided Wilcoxon rank-sum test for independent samples to test whether two sets of data come from the same population. The null hypothesis, *H*_o_, is that both sets come from the same population, while the alternative hypothesis, *H*_1_, is that they come from two different populations. For comparing the data of one feature following a certain treatment against the data of the same feature for the control case, rejecting *H*_o_ is a bad sign for that feature to be a potential biomarker for using that treatment drug, but failure to reject *H*_o_ could be a good sign for a potential biomarker since in the latter case the treatment made the values of this feature post-treatment close to those values for the control. On the other hand, in the case of comparing against the disease model, rejecting *H*_o_ is a good sign for a potential biomarker since the test indicates two different populations: the disease model population and the post-treatment population.

### 3.5 Software packages

Python 3.7 was used as the programming language for processing the data of this study. We used many Python-based packages and modules as well to ease the processing pipeline. “NumPy” (version 1.20.3) and “pandas” (version 1.3.5) were used to read data from files, store them in data structures such as DataFrames, and manipulate data. The machine learning models were built using “scikit-learn” (version 1.0.2) besides splitting the data into iterations of disjoint training and testing samples for cross-validating the results. The package “statsmodels” (version 0.13.2) was incorporated to get the statistical significance for univariate regression models and to correct for the multiple hypothesis tests via the false discovery rate (FDR) approach. Under-sampled classes were up-sampled using the Synthetic Minority Oversampling Technique (SMOTE) implemented in the package “imbalanced-learn” (version 0.11.0). The latter package also was used to construct the processing pipeline across all the predictive models. The Wilcoxon rank-sum test was employed from the package “SciPy” (version 1.7.3). Figures were generated with the help of the package “matplotlib” (version 3.5.0).

## 4 Results

### 4.1 Biochemical analysis of serum samples

We first assessed the expression of hepatic damage markers in the sera. The HPCL group had a significant increase in ALP, ALT, AST, GGT, T. Bilirubin, D. Bilirubin, and Albumin, in addition to the lipid profile (TC, TG, HDL-C, and LDL-C) and the AFP, the tumor marker, in comparison to the control group (*p* < 0.05). However, the nine treatment groups showed a remarkable decrease in the levels of hepatic damage markers (ALT, AST, ALP, GGT, T. Bilirubin, and D. Bilirubin) and improving the lipid profile markers (TC, TG, HDL-C, and LDL-C) in addition to decrease the AFP compared to the HPLC group. This indicates that the treatments were effective in mitigating hepatic damage and normalizing the lipid profile (Table 4).

**TABLE 4:** The effect of HPCL induction and drugs’ administration on serum hepatic damage markers and lipid profile.

	Control	HPCL	Hesperidin-50	Hesperidin-100	Hesperidin-200	Cyan-10	Cyan-20	Cyan-30	Pantoprazole-25	Pantoprazole-50	Pantoprazole-100	*P*-value
ALT	34.56 ± 5	131.15 ± 16.6^a^	158.33 ± 244^ab^	72.875 ± 4.5^ab^	63.34 ± 3.8^ab^	167.8 ± 2.59^ab^	75.062 ± 4.7^ab^	67.77 ± 4.1^ab^	169.5 ± 261.6^ab^	75.81 ± 4.7^ab^	42.78 ± 2.6^b^	0.00
AST	32.104 ± 10.5	91.6 ± 9.2^a^	79.4 ± 6.6^ab^	67.1 ± 3.1^ab^	59.75 ± 3^ab^	84.16 ± 6.9^a^	69.1 ± 3.2^ab^	63.9 ± 3.2^ab^	85 ± 7^a^	69.77 ± 3.2^ab^	37.95 ± 1.89^b^	0.00
ALP	33.7 ± 6.1	123.8 ± 9.6^a^	103.2 ± 10.9^ab^	67.9 ± 6.6^ab^	55.6 ± 6^ab^	109.3 ± 11.6^ab^	70 ± 6.8^ab^	59.5 ± 6.4^ab^	110.4 ± 11.7^ab^	70.7 ± 6.9^ab^	35.4 ± 3.8^b^	0.00
GGT	16.0 ± 3.2	70.5 ± 13.7^a^	72.3 ± 8.9^a^	38.1 ± 3.3^ab^	31 ± 1.5^ab^	76.6 ± 9.5^a^	39.2 ± 3.4^ab^	33.2 ± 1.6^ab^	77.4 ± 9.6^a^	39.6 ± 3.4^ab^	33.5 ± 1.7^ab^	0.00
T. Bilirubin	0.29 ± 0.07	1.61 ± 0.2^a^	1.21 ± 0.25^ab^	0.72 ± 0.06^ab^	0.57 ± 0.04^ab^	1.28 ± 0.27^ab^	0.74 ± 0.07^ab^	0.61 ± 0.0.4^ab^	1.29 ± 0.27^ab^	0.75 ± 0.07^ab^	0.56 ± 0.07^ab^	0.00
D. Bilirubin	0.15 ± 0.02	0.75 ± 0.15^a^	0.57 ± 0.11^ab^	0.32 ± 0.03^ab^	0.27 ± 0.02^ab^	0.61 ± 0.12^ab^	0.33 ± 0.03^ab^	0.29 ± 0.02^ab^	0.62 ± 0.12^ab^	0.33 ± 0.03^ab^	0.29 ± 0.02^ab^	0.00
AFP	19.98 ± 3.5	535.68 ± 81.8^a^	494.93 ± 60.7^a^	121.4 ± 9.2^ab^	46.96 ± 3^b^	524.63 ± 64.3^a^	125 ± 9.5^ab^	55.96 ± 10.75^b^	529.88 ± 64.97^a^	126.28 ± 9.61^ab^	43.05 ± 8.27^b^	0.00
Albumin	1.83 ± 0.28	3.62 ± 0.31^a^	2.62 ± 0.16^ab^	2.96 ± 0.16^ab^	3.66 ± 0.51^a^	2.78 ± 0.17^ab^	3.05 ± 0.17^ab^	3.92 ± 0.55^a^	2.8 ± 0.17^ab^	3.08 ± 0.2^ab^	3.96 ± 0.56^a^	0.00
TC	83.38 ± 11.77	152.25 ± 33.68^a^	134 ± 7.9^ab^	106.77 ± 8.7^ab^	87.53 ± 4.5^b^	142.07 ± 8.37^a^	109.97 ± 8.97^ab^	93.65 ± 4.81^b^	143.49 ± 8.46^a^	111.07 ± 9.06^ab^	94.59 ± 4.86^b^	0.00
TG	61.78 ± 19.36	185.08 ± 50.24^a^	135.13 ± 16.94^ab^	117.64 ± 4.25^a^	72.14 ± 3.6^a^	143.24 ± 17.96^ab^	121.17 ± 4.38^ab^	57.92 ± 2.93^a^	144.67 ± 18.14^ab^	122.38 ± 4.4^ab^	71.56 ± 2.7^a^	0.00
HDL-C	50.31 ± 16.6	27.18 ± 5.1^a^	36.6 ± 2.48^ab^	33.14 ± 1.22^a^	29.25 ± 1.72^a^	38.8 ± 2.6^ab^	34.14 ± 1.26^ab^	31.29 ± 1.84^a^	39.19 ± 2.65^ab^	34.48 ± 1.27^ab^	31.61 ± 1.86^a^	0.00
LDL-C	20.32 ± 8	81.97 ± 31.9^a^	70.33 ± 8.5^a^	43.69 ± 6.8^ab^	30.55 ± 4.98^b^	74.56 ± 9^a^	45 ± 7^ab^	32.69 ± 5.33^b^	75.3 ± 9.1^a^	45.45 ± 7.07^ab^	24.39 ± 1.74^b^	0.00

The data are presented as mean ± SD. The statistically significant difference between the three groups was assessed with the ANOVA-Tukey *post hoc* test, where “a” denotes statistical significance when compared to the control group, and “b” represents statistical significance when compared to the HPCL group. A *p*-value of less than 0.05 is considered statistically significant.

### 4.2 Differential expression analysis of hepatic molecular markers

The relative expression of *BAX*, *ATG16-L1*, *P53*, and *RAB11* mRNAs significantly declined after HPCL induction while *TUBG* and *Cyclin E* mRNAs significantly elevated compared to the normal group. Moreover, there was an obvious dysregulation in the relative expression of various non-coding RNA molecules in the HPCL group compared to the Normal group, including microRNAs; as miR-125b, miR-1289, and miR-1262 were significantly decreased, in contrast, miR-106b was significantly increased compared to the normal group. Interestingly, all lncRNAs displayed a significant increase in relative expression in the HPCL group. However, the relative expression of circ_0001345 showed a significant decrease. The treatments with either Hesperidin, Cyan, or Pantoprazole demonstrated a modulation effect on the expression pattern of mRNA molecules (Table 5).

**TABLE 5:** The effect of HPCL induction and drugs’ administration on the relative expression of the RNA panel.

	Control	HPCL	Hesperidin-50	Hesperidin-100	Hesperidin-200	Cyan-10	Cyan-20	Cyan-30	Pantoprazole-25	Pantoprazole-50	Pantoprazole-100	*P*-value
BAX	1.25 ± 0.42	0.17 ± 0.04^a^	0.21 ± 0.05^a^	3.67 ± 0.6^ab^	4.16 ± 0.69^ab^	5.42 ± 0.52^ab^	6 ± 0.1^ab^	6.67 ± 0.26^ab^	4.38 ± 0.55^ab^	3.34 ± 0.4^ab^	5.21 ± 1.18^ab^	0.00
Cyclin E	0.96 ± 0.14	17.54 ± 2.39^a^	4.51 ± 0.66^ab^	3.65 ± 0.28^ab^	2.13 ± 0.45^b^	2.44 ± 0.32^ab^	1.91 ± 0.11^b^	1.62 ± 0.21^b^	3.85 ± 0.0.7^ab^	3.66 ± 0.75^ab^	2.04 ± 0.33^b^	0.00
ATG16-L1	0.97 ± 0.41	0.17 ± 0.24^a^	1.61 ± 0.14^ab^	1.95 ± 0.16^ab^	2.55 ± 0.43^ab^	1.55 ± 0.26^ab^	2.44 ± 0.25^ab^	3.08 ± 0.4^ab^	1.63 ± 0.36^ab^	1.87 ± 0.27^ab^	2.51 ± 0.37^ab^	0.00
P53	1.26 ± 0.34	0.15 ± 0.04^a^	2.04 ± 0.16^ab^	2.65 ± 0.33^ab^	3.67 ± 0.83^ab^	2.81 ± 0.41^ab^	3.84 ± 0.14^ab^	4.44 ± 0.37^ab^	2.42 ± 0.44^ab^	2.76 ± 0.47^ab^	3.6 ± 0.72^ab^	0.00
RAB11	1.48 ± 0.63	0.13 ± 0.03^a^	1.65 ± 0.16^b^	2.15 ± 0.2^ab^	4.42 ± 0.72^ab^	3.22 ± 0.41^ab^	4.15 ± 0.22^ab^	4.98 ± 0.5^ab^	1.84 ± 0.32^b^	2.08 ± 0.34^b^	4.46 ± 0.9^ab^	0.00
TUBG	0.93 ± 0.08	12.66 ± 1.34^a^	3.58 ± 0.4^ab^	2.53 ± 0.38^ab^	1.53 ± 0.89^b^	1.82 ± 0.52^ab^	1.23 ± 0.05^b^	1.03 ± 0.23^b^	3.48 ± 0.5^ab^	2.6 ± 0.36^ab^	1.44 ± 0.55^b^	0.00
miR-106b	0.92 ± 0.38	14.28 ± 1.51^a^	4.38 ± 0.57^ab^	3.18 ± 0.36^ab^	1.29 ± 0.25^b^	1.41 ± 0.16^b^	1.15 ± 0.09^b^	0.84 ± 0.24^b^	4.11 ± 0.58^ab^	3.12 ± 0.41^ab^	1.21 ± 0.23^b^	0.00
miR-1262	1.32 ± 0.13	0.26 ± 0.13^a^	1.94 ± 0.19^ab^	2.84 ± 0.43^ab^	3.55 ± 0.63^ab^	3.03 ± 0.61^ab^	3.83 ± 0.17^ab^	4.26 ± 0.25^ab^	2.1 ± 0.39^ab^	2.56 ± 0.49^ab^	3.73 ± 0.46^ab^	0.00
miR-125b	1.43 ± 0.52	0.19 ± 0.06^a^	1.53 ± 0.17^b^	2.08 ± 0.31^ab^	3.05 ± 0.3^ab^	2.44 ± 0.56^ab^	3.02 ± 0.16^ab^	3.32 ± 0.2^ab^	1.61 ± 0.21^b^	2.03 ± 0.39^ab^	2.9 ± 0.4^ab^	0.00
miR-1289	1.53 ± 0.62	0.22 ± 0.04^a^	2.25 ± 0.43^ab^	3.33 ± 0.66^ab^	4.35 ± 0.55^ab^	3.85 ± 0.22^ab^	4.36 ± 0.15^ab^	5.03 ± 0.56^ab^	2.29 ± 0.47^ab^	3.02 ± 0.67^ab^	4.28 ± 0.49^ab^	0.00
lncRN-MALAT	0.91 ± 0.18	21.24 ± 2.24^a^	2.71 ± 0.53^ab^	1.99 ± 0.14^ab^	1.16 ± 0.35^b^	1.73 ± 0.2^b^	1.24 ± 0.06^b^	0.96 ± 0.21^b^	2.33 ± 0.47^ab^	1.98 ± 0.24^ab^	1.35 ± 0.46^b^	0.00
lncRNA-RP11-513I15.6	0.92 ± 0.57	10.69 ± 1.93^a^	2.71 ± 0.46^ab^	1.86 ± 0.17^b^	0.86 ± 0.16^b^	1.05 ± 0.15^b^	0.86 ± 0.04^b^	0.73 ± 0.08^b^	2.43 ± 0.68^ab^	2.12 ± 0.25^ab^	0.86 ± 0.25^b^	0.00
lncRNA-RP11-583F2.2	1.11 ± 0.45	6.04 ± 1.1^a^	1.65 ± 0.18^b^	1.07 ± 0.12^b^	0.49 ± 0.37^ab^	0.73 ± 0.04^b^	0.63 ± 0.04^b^	0.54 ± 0.07^ab^	1.56 ± 0.21^b^	1.12 ± 0.3^b^	0.71 ± 0.28^b^	0.00
circ_0001345	1.45 ± 0.65	0.18 ± 0.03^a^	3.53 ± 0.39^ab^	4.43 ± 0.43^ab^	5.8 ± 0.93^ab^	5.26 ± 0.23^ab^	5.95 ± 0.16^ab^	6.2 ± 0.36^ab^	4.38 ± 0.6^ab^	3.26 ± 0.44^ab^	5.63 ± 0.82^ab^	0.00

The data are presented as mean ± SD. The statistically significant difference between the three groups was assessed with the ANOVA-Tukey post hoc test, where “a” denotes statistical significance when compared to the control group, and “b” represents statistical significance when compared to the HPCL group. A *p*-value of less than 0.05 is considered statistically significant.

### 4.3 Histopathological and immunohistochemical evaluation

**FIGURE 3:**
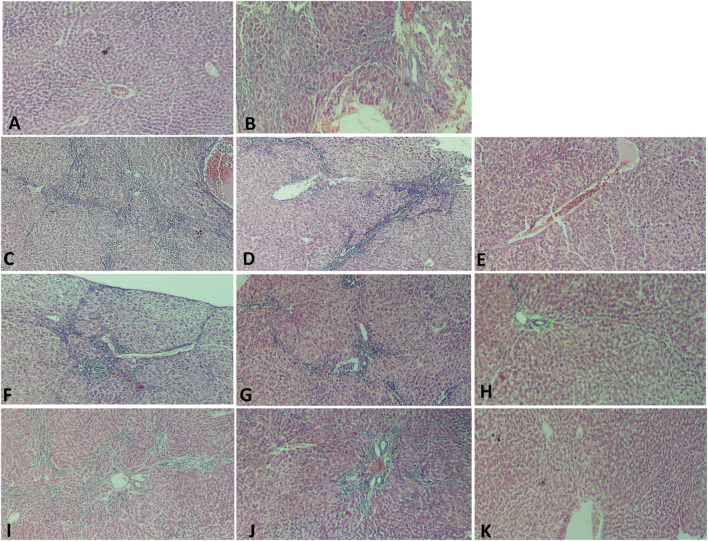
Photomicrographs of liver sections stained with H&E. **(A)** The Control group shows normal hepatic architecture as hepatocytes are arranged radially around the central vein, the structure of the hepatic sinus was clear and there was no pathological change. **(B)** HPCL Group shows massive destruction of liver architecture with infiltration of inflammatory cells and hemorrhagic area. **(C–K)** Treated groups; **(C)** hesperidin-50 Group, **(D)** hesperidin-100 Group, **(E)** hesperidin-200 Group, **(F)** Cyan-10 Group, **(G)** Cyan-20 Group, **(H)** Cyan-30 Group, **(I)** Pantoprazole-25 Group, **(J)** Pantoprazole-50 Group, and **(K)** Pantoprazole-100 Group. (magnification: **(A–K)**: × 100).

**FIGURE 4:**
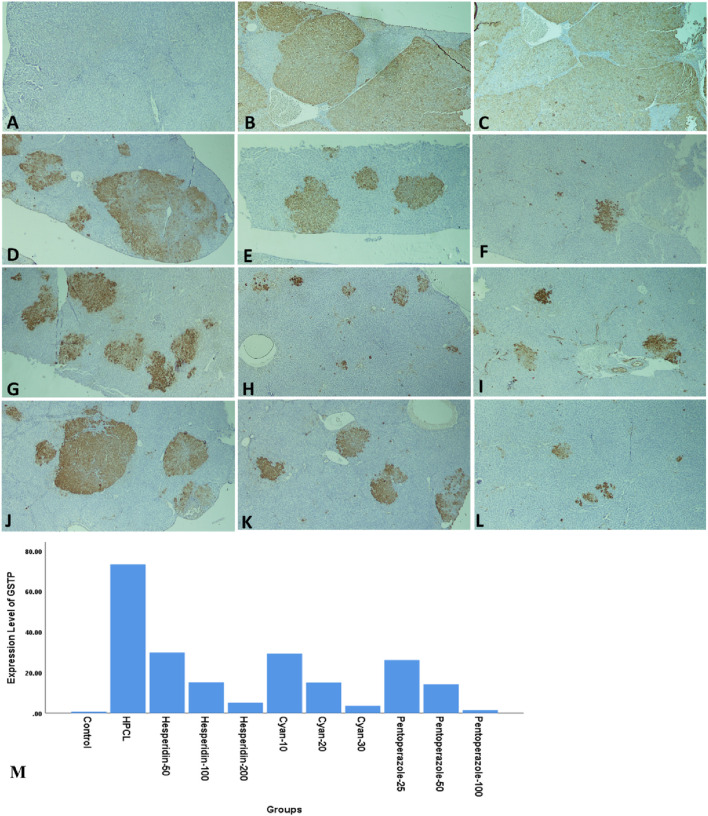
Photomicrographs of rats’ liver sections immunohistochemically stained with glutathione S transferase-P (GST-P) antibody. **(A)** The control group shows a negative reaction. **(B, C)** HPCL Group shows the massive distribution of large positive stained areas occupying most of the section. **(D–L)** Treated groups; **(D)** hesperidin-50 Group, **(E)** hesperidin-100 Group, **(F)** hesperidin-200 Group, **(G)** Cyan-10 Group, **(H)** Cyan-20 Group, **(I)** Cyan-30 Group, **(J)** Pantoprazole-25 Group, **(K)** Pantoprazole-50 Group, and **(L)** Pantoprazole-100 Group. **(M)** A bar chart depicts the levels of expression of GST-P across the different animal groups. (magnification: **(A–L)**:× 40).

**FIGURE 5:**
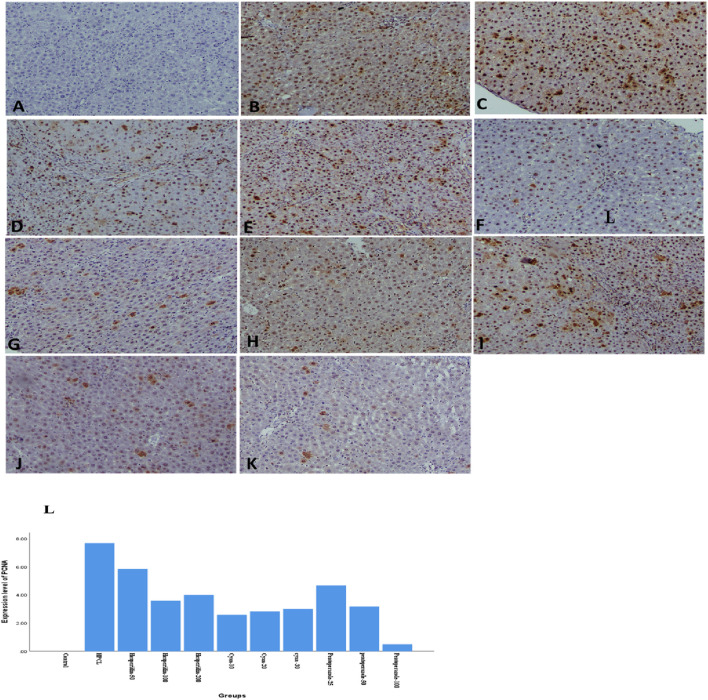
Photomicrographs of rats’ liver sections immunohistochemically stained with PCNA show brown stained nucleus indicating a positive immune reaction of hepatic cells scattered in between negatively stained hepatic parenchymal cells. **(A)** The control group shows a negative reaction. **(B)** HPCL Group shows a massive distribution of positively stained cells occupying most of the section. **(C)** Hesperidin-50 Group, **(D)** hesperidin-100 Group, **(E)** hesperidin-200 Group, **(F)** Cyan-10 Group, **(G)** Cyan-20 Group, **(H)** Cyan-30 Group, **(I)** Pantoprazole-25 Group, **(J)** Pantoprazole-50 Group, **(K)** Pantoprazole-100 Group. (magnification: **(A–K)**: × 100). **(L)** A bar chart depicts the levels of expression of PCNA across the different animal groups.

The histopathological analysis of liver sections depicted distinct histological features among the various experimental groups. The control group exhibited a normal liver structure with the absence of any pathological changes (Figure 3A). However, the HPCL group displayed significant liver damage, characterized by extensive destruction of liver architecture, infiltration of inflammatory cells, and the presence of hemorrhagic areas (Figure 3B). In groups treated with hesperidin and cyan, there were confluent lesions of inflammatory cells infiltrating different areas, including the intralobular, periductal, and perivascular regions (Figures 3C–H). Focal lymphocytic infiltration was observed around portal triads and as individual lesions in some sections treated with hesperidin-100 and Pantoprazole-50 (Figures 3G, J). Conversely, no notable inflammatory lesions were observed in the sections treated with hesperidin-200 and cyan-30 (Figures 3E, H) and Pantoprazole-100 (Figure 3K). The immunohistochemical analysis of liver sections from rats revealed distinct patterns of staining when GST-P antibody was applied. In the control group, no positive reaction was observed. However, in the HPCL group, a substantial distribution of large areas stained positive was evident, occupying a significant portion of the liver section. Upon treatment, the different groups displayed varying results. In the hesperidin-50 group, multiple scattered lesions with positive staining were observed. Similar patterns were seen in the treated groups. These lesions varied in size, ranging from large positive stained nodules in Hesperidin-50 and Pantoprazole-25 groups to small positive hepatic nodules of different sizes in Hesperidin-100, Cyan-10, and Pantoprazole-50 groups. Additionally, some groups displayed foci and collections of brown-stained cells scattered within the negatively stained hepatic parenchyma in Hesperidin-200, Cyan-20, Cyan-30, Pantoprazole-50, and Pantoprazole-100 groups (Figure 4) (Table 6). The results of the PCNA immunohistochemical analysis indicated that there was a higher expression of PCNA in the HPCL group when compared to the control group (Figures 5A, B). Furthermore, when examining liver sections from rats treated with different doses, it was observed that there was a decrease in the number of nuclei that exhibited positive staining for PCNA (Figures 5C–L).

**TABLE 6:** Inflammation grade, fibrosis stage, and IHC analysis with treatment interventions.

	Control	HPCL	Hesperidin-50	Hesperidin-100	Hesperidin-200	Cyan-10	Cyan-20	Cyan-30	Pantoprazole-25	Pantoprazole-50	Pantoprazole-100	*P*-value
Inflammation grade												0.000
0	10 (100%)	0	0	0	0	0	0	4 (33.3%)	0	0	5 (41.7%)	
1	0	0	1 (8.3%)	8 (66.7%)	9 (75%)	8 (66.7%)	8 (66.7%)	7 (58.3%)	0	1 (2.1%)	6 (50%)	
2	0	0	10 (83.3%)	4 (33.3%)	3 (25%)	4 (33.3%)	4 (33.3%)	1 (8.3%)	9 (75%)	11 (91.7%)	0	
3	0	12 (100%)	1 (8.3%)	0	0	0	0	0	3 (25%)	0	1 (8.3%)	
Fibrosis stage												0.000
0	10 (100%)	0	0	0	0	0	0	4 (33.3%)	0	0	4 (33.3%)	
1	0	0	1 (8.3%)	9 (75%)	9 (75%)	8 (66.7%)	8 (66.7%)	7 (58.3%)	0	1 (8.3%)	7 (58.3%)	
2	0	0	10 (83.3%)	3 (25%)	3 (25%)	4 (33.3%)	4 (33.3%)	1 (8.3%)	9 (75%)	11 (91.7%)	1 (8.3%)	
3	0	12 (100%)	1 (8.3%)	0	0	0	0	0	3 (25%)	0	0	
GSTP	0.68 ± 0.15	73.25 ± 4.11^a^	29.83 ± 1.47^ab^	15.18 ± 0.58^ab^	5.18 ± 0.58^b^	29.33 ± 1.6^ab^	15.08 ± 1.03^ab^	3.62 ± 0.67^b^	26.17 ± 2.11^ab^	14.17 ± 0.61^ab^	1.5 ± 0.2^b^	0.000
TNF	1 ± 0.06	38.2 ± 1.52^ab^	19.78 ± 3.59^ab^	12.62 ± 1.05^ab^	3.04 ± 1.07^b^	7.37 ± 1.16^b^	6.36 ± 1.77^b^	9.87 ± 0.51^ab^	3.15 ± 0.92^b^	9.95 ± 1.36^ab^	1.37 ± 0.18^b^	0.000

The inflammation grade and fibrosis stage data are presented as Number (%), and the *P* value was measured using the chi-square test (χ^2^). The numerical data were expressed as mean ± SD. The statistically significant difference between the three groups was assessed with the ANOVA-Tukey *post hoc* test, where “a” denotes statistical significance when compared to the control group, and “b” represents statistical significance when compared to the HPCL group. A *p*-value of less than 0.05 is considered statistically significant.

## 5 Machine learning-based analysis

### 5.1 Prediction of the treatment response using the machine learning models

Table 7 shows the features included in and excluded from the reduced models based on their importance to the classification process using the greedy SFS approach. Only two molecular features (miR-1289 and TUBG mRNA), two biochemical features (ALT and TG), and one IHC feature (GSTP) are kept.

**TABLE 7:** Features kept for the reduced models are listed under the “Included features” column, while the discarded features are listed under the “Excluded features” column.

Model	Included features	Excluded features
	Feature	Feature
Molecular Included: 2 Excluded: 12 Total: 14	MiR-1289 TUBG mRNA	miR-125b lncRNA-RP11-513I15.6 lncRNA-RP11-583F2.2 Cyclin E mRNA lncRNA-MALAT miR-106b miR-1262 BAX mRNA ATG16-L1 P53 mRNA RAB11 mRNA circ_0001345
Biochemical Included: 2 Excluded: 10 Total: 12	ALT TG	AST ALP GGT T. Bilirubin D. Bilirubin AFP Albumin TC HDL-C LDL-C
IHC Included: 1 Excluded: 2 Total: 3	GSTP	PCNA TNF

A set of performance metrics for the complete and reduced models can be found in Tables 8–12 for the LR, kNN, NN, RF, and SVM classifiers; respectively. These metrics are accuracy, precision, recall (sensitivity), specificity, and the Matthews Correlation Coefficient (MCC). The MCC provides unbiased, more accurate evaluation for binary classification problems, especially for the cases of unbalanced data (Chicco and Jurman, 2020). Although we used the SMOTE technique to even the number of samples between the two classification classes, it is advised to still use the MCC to compare performance between the different machine learning models. We used k = 3 for this study. Complete results for each iteration for each classifier can be found in Supplementary Files S2–S6.

**TABLE 8:** Model prediction using multivariate LR for both the complete models and the reduced models.

Model	LR		
	Metric	Complete (All features)	Reduced model (SFS)
Molecular	Accuracy	0.9644 ∓ 0.0135	0.9664 ∓ 0.0135
	Precision	0.9792 ∓ 0.0295	0.9722 ∓ 0.0260
	Recall	0.9787 ∓ 0.0174	0.9858 ∓ 0.0201
	Specificity	0.9333 ∓ 0.0943	0.9056 ∓ 0.0820
	MCC	0.9075 ∓ 0.0345	0.9064 ∓ 0.0332
Biochemical	Accuracy	0.8935 ∓ 0.0337	0.9438 ∓ 0.0082
	Precision	0.9200 ∓ 0.0549	0.9718 ∓ 0.0259
	Recall	0.9500 ∓ 0.0196	0.9574 ∓ 0.0301
	Specificity	0.7111 ∓ 0.1858	0.9056 ∓ 0.0820
	MCC	0.6864 ∓ 0.0983	0.8461 ∓ 0.0277
IHC	Accuracy	0.9494 ∓ 0.0138	0.9607 ∓ 0.0076
	Precision	0.9792 ∓ 0.0295	0.9721 ∓ 0.0259
	Recall	0.9574 ∓ 0.0347	0.9787 ∓ 0.0174
	Specificity	0.9333 ∓ 0.0943	0.9056 ∓ 0.0820
	MCC	0.8668 ∓ 0.0317	0.8891 ∓ 0.0169
Molecular-biochemical	Accuracy	0.9496 ∓ 0.0271	0.9664 ∓ 0.0135
	Precision	0.9594 ∓ 0.0429	0.9722 ∓ 0.0260
	Recall	0.9787 ∓ 0.0174	0.9858 ∓ 0.0201
	Specificity	0.8611 ∓ 0.1416	0.9056 ∓ 0.0820
	MCC	0.8580 ∓ 0.0721	0.9064 ∓ 0.0332
Molecular-IHC	Accuracy	0.9664 ∓ 0.0135	0.9664 ∓ 0.0135
	Precision	0.9792 ∓ 0.0295	0.9722 ∓ 0.0260
	Recall	0.9787 ∓ 0.0174	0.9858 ∓ 0.0201
	Specificity	0.9333 ∓ 0.0943	0.9056 ∓ 0.0820
	MCC	0.9075 ∓ 0.0345	0.9064 ∓ 0.0332
Biochemical-IHC	Accuracy	0.9215 ∓ 0.0152	0.9607 ∓ 0.0076
	Precision	0.9330 ∓ 0.0392	0.9721 ∓ 0.0259
	Recall	0.9716 ∓ 0.0265	0.9787 ∓ 0.0174
	Specificity	0.7556 ∓ 0.1293	0.9056 ∓ 0.0820
	MCC	0.7721 ∓0.0331	0.8891 ∓ 0.0169
Molecular-biochemical-IHC	Accuracy	0.9441 ∓0.0341	0.9664 ∓ 0.0135
	Precision	0.9535 ∓ 0.0511	0.9722 ∓ 0.0260
	Recall	0.9787 ∓ 0.0174	0.9858 ∓ 0.0201
	Specificity	0.8389 ∓ 0.1723	0.9056 ∓ 0.0820
	MCC	0.8423 ∓ 0.0911	0.9064 ∓ 0.0332

Results are shown in the format (average accuracy ∓ variance) for all the k-folds used to cross-validate the models. We used k = 3 for this study. Note that recall is also the sensitivity metric. LR, logestic regression; SFS, sequential feature selection; MCC, matthews correlation coefficient.

**TABLE 9:** Model prediction using kNN for both the complete models and the reduced models.

Model	kNN		
	Metric	Complete (All features)	Reduced model (SFS)
Molecular	Accuracy	0.9776 ∓ 0.0077	0.9720 ∓ 0.0156
	Precision	0.9787 ∓ 0.0174	0.9792 ∓ 0.0295
	Recall	0.9929 ∓ 0.0100	0.9858 ∓ 0.0100
	Specificity	0.9278 ∓ 0.0550	0.9333 ∓ 0.0943
	MCC	0.9363 ∓ 0.0180	0.9223 ∓ 0.0398
Biochemical	Accuracy	0.9157 ∓ 0.0139	0.9607 ∓ 0.0158
	Precision	0.9705 ∓ 0.0270	0.9650 ∓ 0.0194
	Recall	0.9214 ∓ 0.0257	0.9858 ∓ 0.0201
	Specificity	0.9056 ∓ 0.0820	0.8778 ∓ 0.0550
	MCC	0.7785 ∓ 0.0388	0.8874 ∓ 0.0430
IHC	Accuracy	0.9213 ∓ 0.0085	0.9268 ∓ 0.0446
	Precision	0.9711 ∓ 0.0264	0.9721 ∓ 0.0259
	Recall	0.9288 ∓ 0.0357	0.9362 ∓ 0.0757
	Specificity	0.9056 ∓ 0.0820	0.9056 ∓ 0.0820
	MCC	0.7935 ∓ 0.0199	0.8223 ∓0.0834
Molecular-biochemical	Accuracy	0.9663 ∓ 0.0003	0.9720 ∓ 0.0295
	Precision	0.9858 ∓ 0.0201	0.9792 ∓ 0.0242
	Recall	0.9716 ∓ 0.0201	0.9858 ∓ 0.0100
	Specificity	0.9556 ∓ 0.0629	0.9333 ∓ 0.0943
	MCC	0.9076 ∓ 0.0024	0.9223 ∓ 0.0398
Molecular-IHC	Accuracy	0.9663 ∓ 0.0003	0.9720 ∓ 0.0156
	Precision	0.9787 ∓ 0.0174	0.9792 ∓ 0.0295
	Recall	0.9787 ∓ 0.0174	0.9858 ∓ 0.0100
	Specificity	0.9278 ∓ 0.0550	0.9333 ∓ 0.0943
	MCC	0.9041 ∓ 0.0065	0.9223 ∓ 0.0398
Biochemical-IHC	Accuracy	0.9495 ∓ 0.0134	0.9325 ∓ 0.0367
	Precision	0.9787 ∓ 0.0301	0.9721 ∓ 0.0259
	Recall	0.9571 ∓ 0.0170	0.9433 ∓ 0.0658
	Specificity	0.9333 ∓ 0.0943	0.9056 ∓ 0.0820
	MCC	0.8629 ∓ 0.0361	0.8315 ∓ 0.0706
Molecular-biochemical-IHC	Accuracy	0.9663 ∓ 0.0138	0.9720 ∓ 0.0156
	Precision	0.9858 ∓ 0.0201	0.9792 ∓ 0.0295
	Recall	0.9716 ∓ 0.0265	0.9858 ∓ 0.0100
	Specificity	0.9556 ∓ 0.0629	0.9333 ∓ 0.0943
	MCC	0.9089 ∓ 0.0348	0.9223 ∓ 0.0398

Results are shown in the format (average accuracy ∓ variance) for all the k-folds used to cross-validate the models. We used *k* = 3 for this study. Note that recall is also the sensitivity metric. kNN, k-nearest neighbors; SFS, sequential feature selection; MCC, matthews correlation coefficient.

**TABLE 10:** Model prediction using NN for both the complete models and the reduced models.

Model	NN		
	Metric	Complete (All features)	Reduced model (SFS)
Molecular	Accuracy	0.9777 ∓ 0.0208	0.9718 ∓ 0.0081
	Precision	0.9722 ∓ 0.0260	0.9928 ∓ 0.0102
	Recall	1.0000 ∓ 0.0000	0.9716 ∓ 0.0201
	Specificity	0.9056 ∓ 0.0820	0.9778 ∓ 0.0314
	MCC	0.9378 ∓ 0.0551	0.9224 ∓ 0.0234
Biochemical	Accuracy	0.9440 ∓ 0.0284	0.9720 ∓ 0.0156
	Precision	0.9709 ∓ 0.0271	0.9722 ∓ 0.0260
	Recall	0.9568 ∓ 0.0174	0.9929 ∓ 0.0100
	Specificity	0.9056 ∓ 0.0820	0.9056 ∓ 0.0820
	MCC	0.8436 ∓ 0.0768	0.9213 ∓ 0.0391
IHC	Accuracy	0.9327 ∓ 0.0236	0.9664 ∓ 0.0135
	Precision	0.9712 ∓ 0.0272	0.9721 ∓ 0.0259
	Recall	0.9426 ∓ 0.0359	0.9858 ∓ 0.0100
	Specificity	0.9056 ∓ 0.0820	0.9056 ∓ 0.0820
	MCC	0.8210 ∓ 0.0532	0.9040 ∓ 0.0350
Molecular-biochemical	Accuracy	0.9832 ∓ 0.0136	0.9718 ∓ 0.0081
	Precision	0.9858 ∓ 0.0201	0.9928 ∓ 0.0102
	Recall	0.9929 ∓ 0.0100	0.9787 ∓ 0.0174
	Specificity	0.9556 ∓ 0.0629	0.9778 ∓ 0.0314
	MCC	0.9538 ∓ 0.0364	0.9224 ∓ 0.0234
Molecular-IHC	Accuracy	0.9832 ∓ 0.0136	0.9718 ∓ 0.0081
	Precision	0.9858 ∓ 0.0201	0.9928 ∓ 0.0102
	Recall	0.9929 ∓ 0.0100	0.9716 ∓ 0.0201
	Specificity	0.9556 ∓ 0.0629	0.9778 ∓ 0.0314
	MCC	0.9538 ∓ 0.0364	0.9224 ∓ 0.0234
Biochemical-IHC	Accuracy	0.8930 ∓ 0.0293	0.9325 ∓ 0.0367
	Precision	0.9117 ∓ 0.0222	0.9721 ∓ 0.0259
	Recall	0.9571 ∓ 0.0170	0.9433 ∓ 0.0658
	Specificity	0.6556 ∓ 0.1227	0.9056 ∓ 0.0820
	MCC	0.6595 ∓ 0.1170	0.8315 ∓ 0.0706
Molecular-biochemical-IHC	Accuracy	0.9776 ∓ 0.0077	0.9718 ∓ 0.0081
	Precision	0.9789 ∓ 0.0174	0.9928 ∓ 0.0102
	Recall	0.9929 ∓ 0.0100	0.9716 ∓ 0.0201
	Specificity	0.9278 ∓ 0.0550	0.9778 ∓ 0.0314
	MCC	0.9363 ∓ 0.0180	0.9224 ∓ 0.0234

Results are shown in the format (average accuracy ∓ variance) for all the k-folds used to cross-validate the models. We used *k* = 3 for this study. Note that recall is also the sensitivity metric. NN, neural networks; SFS, sequential feature selection; MCC, matthews correlation coefficient.

**TABLE 11:** Model prediction using RF for both the complete models and the reduced models.

Model	RF		
	Metric	Complete (All features)	Reduced model (SFS)
Molecular	Accuracy	0.9777 ∓ 0.0208	0.9776 ∓ 0.0077
	Precision	0.9792 ∓ 0.0295	0.9858 ∓ 0.0201
	Recall	0.9929 ∓ 0.0100	0.9858 ∓ 0.0100
	Specificity	0.9333 ∓ 0.0943	0.9556 ∓ 0.0629
	MCC	0.9388 ∓ 0.0553	0.9373 ∓ 0.0187
Biochemical	Accuracy	0.9325 ∓ 0.0142	0.9494 ∓ 0.0277
	Precision	0.9716 ∓ 0.0258	0.9722 ∓ 0.0260
	Recall	0.9433 ∓ 0.0401	0.9645 ∓ 0.0501
	Specificity	0.9056 ∓ 0.0820	0.9056 ∓ 0.0820
	MCC	0.8210 ∓ 0.0401	0.8692 ∓ 0.0626
IHC	Accuracy	0.9213 ∓ 0.0290	0.9100 ∓ 0.0578
	Precision	0.9715 ∓ 0.0265	0.9716 ∓ 0.0265
	Recall	0.9288 ∓ 0.0556	0.9146 ∓ 0.0900
	Specificity	0.9056 ∓ 0.0820	0.9056 ∓ 0.0820
	MCC	0.7999 ∓ 0.0489	0.7894 ∓ 0.1000
Molecular-biochemical	Accuracy	0.9777 ∓ 0.0208	0.9858 ∓ 0.0077
	Precision	0.9792 ∓ 0.0295	0.9750 ∓ 0.0201
	Recall	0.9929 ∓ 0.0100	0.9858 ∓ 0.0100
	Specificity	0.9333 ∓ 0.0943	0.9556 ∓ 0.0629
	MCC	0.9388 ∓ 0.0553	0.9373 ∓ 0.0187
Molecular-IHC	Accuracy	0.9777 ∓ 0.0208	0.9776 ∓ 0.0077
	Precision	0.9792 ∓ 0.0295	0.9858 ∓ 0.0201
	Recall	0.9929 ∓ 0.0100	0.9858 ∓ 0.0100
	Specificity	0.9333 ∓ 0.0943	0.9556 ∓ 0.0629
	MCC	0.9388 ∓ 0.0553	0.9373 ∓ 0.0187
Biochemical-IHC	Accuracy	0.9664 ∓ 0.0135	0.9326 ∓ 0.0138
	Precision	0.9721 ∓ 0.0259	0.9715 ∓ 0.0265
	Recall	0.9858 ∓ 0.0100	0.9429 ∓ 0.0359
	Specificity	0.9056 ∓ 0.0820	0.9056 ∓ 0.0820
	MCC	0.9040 ∓ 0.0350	0.8202 ∓ 0.0224
Molecular-biochemical-IHC	Accuracy	0.9777 ∓ 0.0208	0.9776 ∓ 0.0077
	Precision	0.9792 ∓ 0.0295	0.9858 ∓ 0.0201
	Recall	0.9929 ∓ 0.0100	0.9858 ∓ 0.0100
	Specificity	0.9333 ∓ 0.0943	0.9556 ∓ 0.0629
	MCC	0.9388 ∓ 0.0553	0.9373 ∓ 0.0187

Results are shown in the format (average accuracy ∓ variance) for all the k-folds used to cross-validate the models. We used *k* = 3 for this study. Note that recall is also the sensitivity metric. RF, random forest; SFS, sequential feature selection; MCC, matthews correlation coefficient.

**TABLE 12:** Model prediction using SVM classification for both the complete models and the reduced models.

Model	SVM		
	Metric	Complete (All features)	Reduced model (SFS)
Molecular	Accuracy	0.9777 ∓ 0.0208	0.9777 ∓ 0.0208
	Precision	0.9722 ∓ 0.0260	0.9722 ∓ 0.0260
	Recall	1.0000 ∓ 0.0000	1.0000 ∓ 0.0000
	Specificity	0.9056 ∓ 0.0820	0.9056 ∓ 0.0820
	MCC	0.9378 ∓ 0.0551	0.9378 ∓ 0.0551
Biochemical	Accuracy	0.9777 ∓ 0.0208	0.9607 ∓ 0.0287
	Precision	0.9722 ∓ 0.0260	0.9718 ∓ 0.0259
	Recall	1.0000 ∓ 0.0000	0.9787 ∓ 0.0301
	Specificity	0.9056 ∓ 0.0820	0.9056 ∓ 0.0820
	MCC	0.9378 ∓ 0.0551	0.8910 ∓ 0.0808
IHC	Accuracy	0.9720 ∓ 0.0156	0.9607 ∓ 0.0076
	Precision	0.9722 ∓ 0.0260	0.9721 ∓ 0.0259
	Recall	0.9929 ∓ 0.0100	0.9787 ∓ 0.0174
	Specificity	0.9056 ∓ 0.0820	0.9056 ∓ 0.0820
	MCC	0.9213 ∓ 0.0391	0.8891 ∓ 0.0169
Molecular-biochemical	Accuracy	0.9777 ∓ 0.0208	0.9777 ∓ 0.0208
	Precision	0.9722 ∓ 0.0260	0.9722 ∓ 0.0260
	Recall	1.0000 ∓ 0.0000	1.0000 ∓ 0.0000
	Specificity	0.9056 ∓ 0.0820	0.9056 ∓ 0.0820
	MCC	0.9378 ∓ 0.0551	0.9378 ∓ 0.0551
Molecular-IHC	Accuracy	0.9777 ∓ 0.0208	0.9777 ∓ 0.0208
	Precision	0.9665 ∓ 0.0260	0.9722 ∓ 0.0260
	Recall	1.0000 ∓ 0.0000	1.0000 ∓ 0.0000
	Specificity	0.9056 ∓ 0.0820	0.9056 ∓ 0.0820
	MCC	0.9378 ∓ 0.0551	0.9378 ∓ 0.0551
Biochemical-IHC	Accuracy	0.9777 ∓ 0.0208	0.9664 ∓ 0.0135
	Precision	0.9722 ∓ 0.0260	0.9721 ∓ 0.0259
	Recall	1.0000 ∓ 0.0000	0.9858 ∓ 0.0100
	Specificity	0.9056 ∓ 0.0820	0.9056 ∓ 0.0820
	MCC	0.9378 ∓ 0.0551	0.9040 ∓ 0.0350
Molecular-biochemical-IHC	Accuracy	0.9777 ∓ 0.0208	0.9777 ∓ 0.0208
	Precision	0.9722 ∓ 0.0260	0.9722 ∓ 0.0260
	Recall	1.0000 ∓ 0.0000	1.0000 ∓ 0.0000
	Specificity	0.9056 ∓ 0.0820	0.9056 ∓ 0.0820
	MCC	0.9378 ∓ 0.0551	0.9378 ∓ 0.0551

Results are shown in the format (average accuracy ∓ variance) for all the k-folds used to cross-validate the models. We used *k* = 3 for this study. Note that recall is also the sensitivity metric. SVM, support vector machines; SFS, sequential feature selection; MCC, matthews correlation coefficient.

Using each data type alone (Molecular, Biochemical, IHC) in both the complete and reduced models, and generally speaking for the different classifiers, the molecular model gave the best MCC values, while the worst performance came from the biochemical data for the LR and kNN classifiers, and from the IHC data for the NN, RF, and SVM classifiers. For the reduced models, the MCC values for the molecular models were close to those from the complete models, and they were better than those of the complete models for the biochemical and IHC data for all the classifiers except the SVM classifier.

Integrating two or all the data types in one model for the case of the complete models, generally, degraded the performance compared to the best accuracy by any of the single-type models. On the other hand, adding the molecular data to either the biochemical data or the IHC data enhanced the performance compared to either of their individual cases. A few cases of integrating more than one data type demonstrated being slightly superior to the performance of the best individual data type such as combining the molecular and biochemical or molecular and IHC data types with the NN classifier.

In most cases, the reduced models combining the molecular data type showed consistent performance comparable to the reduced molecular model, because the greedy SFS approach favored the important molecular features over those from the biochemical and IHC features, emphasizing the stronger relationship between the phenotype and the genotypic features.

**FIGURE 6:**
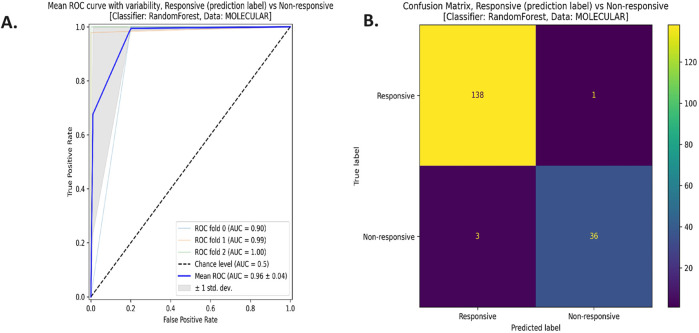
**(A)** The Receiver Operating Characteristics (ROC) curve, and **(B)** the confusion matrix for the reduced model with the random forest (RF) classifier using the molecular data type.

Figure 6 shows the receiver operating characteristics (ROC) curve (Figure 2A) and the confusion matrix (Figure 2B) for the reduced model using the RF classifier and the molecular features. The average area under the ROC curve (AUC) is 0.96 ∓ 0.04 coming from three cross-validation iterations. Moreover, this model successfully predicted 138 responsive (positive) samples out of 139 samples (true positives) and 36 non-responsive (negative) samples out of 39 samples (true negatives). All the ROC curves and confusion matrices for all the models can be found in the Supplementary Figures S6, S7 in Supplementary 1.

### 5.2 Potential biomarkers identification

Tables 13–15 show the hypothesis testing results for the molecular, biochemical, and IHC data types, respectively, comparing the individual features of each data type against the control and against the disease model. These tables do not show the results for all the features, but the results for features with positive or negative promise to be biomarkers. Complete results can be found in the Supplementary Files S7–S12. To summarize the number of occurrences for each treatment drug as either an effective or ineffective biomarker, Table 16 shows these numbers across the three data types. Generally speaking, increasing the dose enhances the chance for a drug to be effective.

**TABLE 13:** Molecular data: Statistically assessing the difference between values of a feature post-treatment against the control and against the disease model. Only statistically insignificant differences are shown. Similar behavior to the control case is a positive sign, while similarity to the disease model is not favorable. q-values are after using the false discovery rate (FDR) approach to correct for multiple hypotheses.

Case	Gene	Drug	Statistics, *P*-value, q-value
Drug vs. Control (similar populations: q > α)	lncRNA-RP11-513I15.6	Hesperidin-200	1.252823, 0.210270, 0.270347
		Cyan-10	−1.120947, 0.262311, 0.295099
		Cyan-20	1.384699, 0.166145, 0.249217
		Pantoprazole-100	0.989071, 0.322629, 0.322629
	miR-125b	Hesperidin-50	−1.945172, 0.051754, 0.051754
		Pantoprazole-25	−1.945172, 0.051754, 0.051754
	LNC-RP11-583F2.2	Hesperidin-100	−1.252823, 0.210270, 0.236554
		pantoprazole-50	−0.659380, 0.509651, 0.509651
	lncRNA-MALAT	hesperidin-200	−1.582513, 0.113532, 0.127724
		Cyan-30	0.065938, 0.947427, 0.947427
	RAB11 mRNA	Hesperidin-50	−1.813296, 0.069786, 0.069786
	miR-106b	cyan -30	0.989071, 0.322629, 0.322629
	TUBG mRNA	cyan -30	−1.846265, 0.064854, 0.064854
Drug vs. HPCL (similar populations: q > α)	BAX mRNA	Hesperidin-50	−1.905256, 0.056747, 0.056747

**TABLE 14:** Biochemical data: Statistically assessing the difference between values of a feature post-treatment against the control and against the disease model. Only statistically insignificant differences are shown. Similar behavior to the control case is a positive sign, while similarity to the disease model is not favorable. q-values are after using the false discovery rate (FDR) approach to correct for multiple hypotheses.

Case	Gene	Drug	Statistic, *p*_value, q_value
Drug vs. Control (similar populations: q > α)	AST	Pantoprazole-100	−1.780327, 0.075022, 0.075022
	ALP	Pantoprazole-100	−0.197814, 0.843190, 0.843190
	Albumin	Hesperidin-200	0.065938, 0.947427, 0.947427
		Cyan-30	−1.384699, 0.166145, 0.186913
		Pantoprazole-100	−1.714389, 0.086457, 0.111159
	TC	Hesperidin-200	−1.087978, 0.276605, 0.276605
		Pantoprazole-100	−1.714389, 0.086457, 0.097264
	TG	Hesperidin-200	−0.989071, 0.322629, 0.414808
		Cyan-30	−0.791257, 0.428794, 0.428794
		Pantoprazole-100	−0.857195, 0.391337, 0.428794
	HDL-C	Hesperidin-50	1.978141, 0.047913, 0.061602
		Cyan-10	1.186885, 0.235273, 0.264682
		Pantoprazole-25	1.055009, 0.291421, 0.291421
	LDL-C	Pantoprazole-100	−0.923133, 0.355938, 0.355938
Drug vs. HPCL (similar populations: q > α)	AST	Cyan-10	1.847521, 0.064672, 0.072756
		Pantoprazole-25	1.645448, 0.099877, 0.099877
	HDL-C	Hesperidin-200	−0.635085, 0.525373, 0.525373
	LDL-C	Hesperidin-50	0.057735, 0.953960, 0.953960
		Cyan-10	−0.346410, 0.729034, 0.820164
		Pantoprazole-25	−0.404145, 0.686106, 0.820164

**TABLE 15:** IHC data: Statistically assessing the difference between values of a feature post-treatment against the control and against the disease model. Only statistically insignificant differences are shown. Similar behavior to the control case is a positive sign, while similarity to the disease model is not favorable. q-values are after using the false discovery rate (FDR) approach to correct for multiple hypotheses.

Case	Gene	Drug	Statistic, *p*_value, q_value
Drug vs. Control (similar populations: q > α)	TNF	Pantoprazole-100	−1.681420, 0.092681, 0.092681
Drug vs. HPCL (similar populations: q > α)	None	None	None

**TABLE 16:** Summary for how many times a treatment drug showed potential for being a treatment response biomarker and how many times it showed incapability. Numbers are displayed in the format (molecular, biochemical, IHC).

Drug	Potentially effective	Potentially ineffective
Hesperidin-50	3: (2, 1, 0)	2: (1, 1, 0)
Hesperidin-100	1: (1, 0, 0)	0: (0, 0, 0)
Hesperidin-200	5: (2, 3, 0)	1: (0, 1, 0)
Cyan-10	2: (1, 1, 0)	2: (0, 2, 0)
Cyan-20	1: (1, 0, 0)	0: (0, 0, 0)
Cyan-30	5: (3, 2, 0)	0: (0, 0, 0)
Pantoprazole-25	2: (1, 1, 0)	2: (0, 2, 0)
Pantoprazole-50	3: (1, 0, 0)	0: (0, 0, 0)
Pantoprazole-100	8: (1, 6, 1)	0: (0, 0, 0)

## 6 Discussion

The field of precision medicine has been enriched by recent advancements in pharmacogenomics, providing a wealth of diverse data types. Analyzing these data has aided in identifying unique cellular sensitivity and resistance patterns of different targets to numerous chemical compounds. One of the key goals of cancer research is to uncover the genomic and molecular characteristics responsible for specific clinical outcomes (Aubrecht et al., 2013). Predictive machine learning models with gene expression features offer an opportunity to explore the impact of molecular features on treatment response, enhancing our understanding of cancer vulnerabilities and enabling the development of predictive models for drug responsiveness. In this study, we employed bioinformatics and machine learning techniques to select robust features from a set of 14 molecular, 12 biochemical, and 3 IHC markers to predict the response to three different drugs (Hesperidin, Cyan, and Pantoprazole) at varying dosages in an HCC rat model.

In our study, we chose three drugs (hesperidin, Pantoprazole, and cyan) due to their established hepatoprotective properties, which include anti-inflammatory and antiapoptotic effects. Hesperidin exhibits anti-cancer effects by inducing cell death in hepatocellular carcinoma HepG2 cells through caspase-independent pathways, primarily by activating the ERK1/2 pathway (Yumnam et al., 2014). Moreover, it induces apoptosis by increasing the accumulation of reactive oxygen species (ROS) and activating the apoptosis signal-regulating kinase 1/Jun N-terminal kinase (ASK1/JNK) pathway (Xia et al., 2018). Additionally, hesperidin inhibits calcium/calmodulin-dependent protein kinase IV (*CAMKIV*), thereby activating the caspase-3-dependent intrinsic pathway and upregulating the pro-apoptotic protein *Bax*, which contributes to its anti-apoptotic and anticancer properties (Naz et al., 2019). Recent evidence suggests that hesperidin induces apoptosis in HeLa cells by involving the endoplasmic reticulum stress pathway and arresting the cell cycle at the G0/G1 phase through the downregulation of *cyclin D1, cyclin E1*, and cyclin-dependent kinase 2 (*Cdk2*) at the protein level (Wang et al., 2015). Cyan effectively inhibits hepatic gluconeogenesis by reducing the expression of gluconeogenic genes. This inhibition is achieved through the phosphorylation and inactivation of coactivators *CRTC2* and *HDAC5* by AMPK. Although Cyan does not directly interact with AMPK, it activates AMPK via the adiponectin receptor signaling pathway, as evidenced by experiments involving the knockdown of adiponectin receptor genes. Furthermore, Cyan increases cellular AMP levels in hepatocytes, and oral administration of Cyan in mice leads to elevated plasma adiponectin concentrations, collectively contributing to AMPK activation (Jia et al., 2022). Additionally, Cyan exhibits potent antioxidant properties and induces cellular senescence and apoptosis in hepatocarcinoma cells under oxidative stress conditions. Cyan enhances the expression of senescence-associated β-galactosidase and key markers of cellular senescence, namely *P16*, *P21*, and *P53*. Tan et al. (2020) found that Cyan exerted a protective effect on HepG2 cells against oxidative damage induced by H_2_O_2_ by reducing reactive radicals. Additionally, Cyan enhanced the expression of important antioxidant enzymes such as SOD, CAT, and GPx. Moreover, Cyan demonstrates a regulatory effect on cell survival and apoptosis by decreasing the levels of the pro-apoptotic protein Bax and increasing the levels of the anti-apoptotic protein Bcl-2 in HepG2 cells (Panritdum et al., 2024). In experiments using LX-2 cells and mouse hepatic stellate cells (pHSCs), Lu et al. demonstrated that pantoprazole dose-dependently notably alleviated liver damage, reduced collagen buildup and inflammation, and inhibited the expression of fibrosis-related genes like *Col1a1*, *Acta2*, *Tgfβ1*, and *Mmp-2*. Through transcriptome analysis and subsequent validation in pantoprazole-treated LX-2 cells, they found that pantoprazole hindered the expression of Yes-associated protein (YAP) and its downstream targets, such as *CTGF*, *ID1*, *survivin*, *CYR61*, and *GLI2*. By manipulating *YAP* expression levels, they demonstrated that PPZ downregulated hepatic fibrogenic gene expression via YAP. Furthermore, pantoprazole facilitated the degradation and ubiquitination of YAP via the proteasome, inhibiting HSC activation. PPZ also disrupted the interaction between the deubiquitinating enzyme *OTUB2* and *YAP*, leading to *YAP* destabilization and impeding hepatic fibrosis progression (Lu et al., 2021).

Additionally, these drugs have demonstrated efficacy in regulating exosome production and autophagy in HCC. Exosomes are now acknowledged as vital facilitators of communication between cells, and their miRNAs, which are involved in cell proliferation and metastasis, play essential roles in the progression of HCC (Mathieu et al., 2019; Zhang et al., 2021). Exosomes stimulate intercellular autophagy to eliminate the negative effects caused by cancer-causing agents, while autophagy, in turn, regulates both the biogenesis and degradation of exosomes (Bunggulawa et al., 2018). Previous research conducted by our team has shown that hesperidin treatment induces the formation of multiple hepatic vacuoles at various stages of autophagosome development. Our findings revealed the gradual emergence of multilamellar bodies, which are specific autophagic vacuoles composed of concentric membrane layers enclosing cellular organelles. Hesperidin has been found to enhance the activity of autophagy in hepatocytes. Additionally, it downregulates exosomal *RAB11A* mRNA and upregulates exosomal miR-1298, resulting in protective effects on the liver in rat models (Hasanin et al., 2020). Pantoprazole primarily targets gastric H+/K+ ATPases and V-ATPases (Spugnini et al., 2015). Its anti-neoplastic effects are attributed to the inhibition of V-ATPase activity, leading to cytosolic acidification and alkalization of endosomal and lysosomal compartments (De Milito et al., 2012). Our previous study proved that Pantoprazole administration reduces the expression of GST-P and PCNA, significantly decreases exosomal *RAB11A* mRNA levels, along with downregulating exosomal Lnc-RNA-RP11-513I15.6, which were found to be elevated in the HCC animal model (Matboli et al., 2019). The beneficial effects of Anthocyanins, including cyan, involve various signaling pathways such as MAPK, NF-κB, AMPK, and Wnt/β-catenin, as well as critical cellular processes including apoptosis and autophagy (Li et al., 2017). Previous research has demonstrated that cyan exhibits a protective effect against high glucose-induced podocyte dysfunction by improving autophagy, reducing apoptosis, and suppressing epithelial-mesenchymal transition through the activation of the SIRT1/AMPK pathway (Wang et al., 2021). Moreover, cyan-enhanced autophagy reduces apoptosis in primary human dermal fibroblasts by mitigating oxidative stress, suggesting a potential mechanism for protection against UVA light-induced damage (Wu et al., 2019).

Most cases of HCC arise from prolonged inflammation in the liver, leading to disruptions in various cellular signaling pathways. These changes in the liver environment disrupt the equilibrium between cell proliferation and cell death, ultimately increasing the likelihood of malignant transformation. Over 90% of HCC cases arise in the presence of hepatic injury and inflammation. The risk factors associated with HCC trigger an unresolved inflammatory response characterized by the infiltration of macrophages and immature myeloid cells, as well as dysregulated cytokine production, and this perpetuates a wound-healing response, leading to the progressive development of fibrosis, cirrhosis, and ultimately HCC (Keenan et al., 2019). During the premalignant stage of hepatocarcinogenesis, chronic activation of inflammatory signaling pathways generates reactive oxygen species (ROS) and reactive nitrogen species (NOS) (Refolo et al., 2020). This chronic inflammation, characterized by the infiltration of macrophages and immature myeloid cells and dysregulated cytokine production, is considered a primary trigger for the development and progression of HCC. In the premalignant environment, inflammatory cells, including stromal cells, produce various molecules such as cytokines, growth factors, chemokines, prostaglandins, and proangiogenic factors (Yu et al., 2018). Autophagy has a significant relationship with inflammation in HCC (Yu et al., 2017). In the early stages of hepatocarcinogenesis, autophagy helps alleviate oxidative stress, prevents genomic instability, and limits uncontrolled inflammation, thereby playing a crucial role in tumor suppression, however, as HCC progresses and autophagy increases, it becomes relevant to poor prognosis and is associated with tumor cell survival under conditions that typically induce cell death, such as hypoxia and nutrient deprivation (Sun et al., 2013). In addition, autophagy deficiency in macrophages can lead to the production of inflammatory and fibrogenic factors (Sun et al., 2021). The depletion of Kupffer cells has been shown to rescue the tumor-promoting effect of autophagy deficiency during the preneoplastic stage. This suggests that autophagy regulates inflammation and fibrosis-promoting effects, potentially through the modulation of NF-κB-associated pathways and the production of cytokines such as IL1α/β (Sun et al., 2017). Therefore, the interplay between autophagy and inflammation is a critical aspect of HCC progression and may offer new insights for targeted therapeutic strategies.

*ATG16-L1*, an essential protein in autophagy, plays a crucial role in HCC, its deficiency leads to decreased bacterial clearance and abnormal interleukin-1β production, fostering inflammation and carcinogenesis (Peantum et al., 2018). The *ATG16-L1* p.T300A polymorphism emerged as a significant risk factor for HCC in cirrhotic patients (Reuken et al., 2019). Studies using a mouse model showed that increased ATG16L1 activation is required to prevent the progression of steatohepatitis and the onset of HCC. Furthermore, the overexpression of *ATG16-L1* suppressed NF-κB and IL6 signaling pathways, suggesting its potential role in mitigating HCC development (Okada et al., 2017). *RAB11A* serves as a target molecule involved in various microRNA-mediated tumor suppression processes. Zhang et al. proved that *RAB11A is* involved in HCC progression by regulating the expression of MMP2 by activating the PI3K/AKT signaling pathway (Zhang et al., 2020). The overexpression of cyclin E in HCC is believed to play a crucial role in promoting cell proliferation and survival (Zhou et al., 2003). *Cyclin E1* and *cyclin E2*, which interact with cyclin-dependent kinase 2 (*Cdk2*), are known to stimulate cell cycle progression and initiation of HCC (Sonntag et al., 2018). Chen et al. provided evidence showing that *TUBG1* exhibited substantial upregulation in both non-alcoholic fatty liver disease (NAFLD) and HCC tissues. This suggests that *TUBG1* serves as a carcinogenic factor contributing to the development of NAFLD and HCC (Chen et al., 2022). Apoptosis induction triggered by oncogene activation, DNA damage, and senescence is a recognized mechanism during chronic liver damage, crucial for cancer prevention (Zhang et al., 2016). The proteins *BAX* and *BAK* control the choice between cell survival and mitochondrial apoptosis. Thus, assessing the regulation of BAX/BAK can anticipate cellular predisposition to apoptosis (Funk et al., 2020). p53 plays an essential role in promoting growth arrest and inducing cell death. The inactivation or loss of functional p53 is a necessary condition for oncogenesis, as it allows for uncontrolled growth and proliferation. This is a prevalent abnormality observed in various human cancers, including HCC (Luo et al., 2021).

Furthermore, our investigation revealed dysregulated expression levels of various mRNA fragments in the HCC rat model. Notably, *Cyclin E* and *TUBG* mRNAs showed increased expression, while *BAX*, *ATG16-L1*, *P53*, and *RAB11* mRNA exhibited downregulation. The administration of the drugs resulted in the modulation of these expression disturbances. Importantly, previous research, including our own, has highlighted the significance of these biomarkers in HCC pathogenesis. For instance, *RAB11A* and *ATG16L1* are core genes involved in autophagy, and their interaction suggests a novel model for autophagosome biogenesis. The *BAX* mRNA serves as a central regulator of cell death and mitochondrial dysfunction. The loss of tumor suppressor p53, often observed in tumors, leads to defects in the cell cycle and hampers the ability to respond to DNA damage or oncogene dysregulation, ultimately inducing apoptosis or cellular senescence. *Cyclin E* contributes to increased proliferative drive favoring hepatocarcinogenesis. Moreover, *cyclin E* and p53 have been reported to inversely regulate each other’s expression, possibly mediated by miR-34 (Pok et al., 2013). The overexpression of *γ-TUBG* has been identified as a characteristic feature of thyroid, breast, and liver cancers it also can induce carcinogenesis (Niu et al., 2009; Hořejší et al., 2012).

Abnormal expression of non-coding RNAs (such as miRNAs, lncRNAs, and cicRNA) has been detected in HCC regulation and linked to critical aspects of cell invasion, metastasis, and drug resistance. In our study, we observed significant disruptions in the expression of non-coding RNAs following the induction of HCC. Notably, circ_0001345 showed a pronounced decrease in expression, accompanied by an upregulation of miR-106b, while other miRNAs (miR-1262, miR-125b, and miR-1289) exhibited downregulation. Additionally, all examined lncRNAs (lncRNA-MALAT, lncRNA-RP11-513I15.6, and lncRNA-RP11-583F2.2) displayed downregulation. Interestingly, the administration of the drugs resulted in the restoration of these expression disruptions.

Regarding the biochemical analysis, we observed in the HPCL considerable elevation in liver destructive markers, indicating liver damage. Additionally, the serum lipid profile displayed significant alterations, particularly a significant decrease in HDLC levels. The AFP, a well-established biomarker used for HCC screening, diagnosis, prognostication, and therapeutic evaluation, (Hu et al., 2022) showed a striking increase. However, following drug administration, we observed enhanced biochemical and AFP levels. This was accompanied by a decrease in fibrotic and inflammatory states, as well as a significant reduction in the percentage area of GSTP foci, PCNA expression, and TNF levels, particularly with higher drug doses.

Machine learning algorithms a branch of AI are increasingly being employed for personalized predictions of drug responses (Vadapalli et al., 2022). These algorithms facilitate the integration of data from diverse sources in a statistically meaningful manner, allowing for the identification of predictive biomarkers (Yamanishi et al., 2012; Rampášek et al., 2019). A critical aspect of this process is how data from multiple sources are harmoniously integrated to enhance the overall prediction performance of drug responses. In alignment with this concept, various approaches have been developed for drug response prediction by leveraging prior knowledge based on genomic and molecular profiles (Stanfield et al., 2017; Cichonska et al., 2018). These approaches aim to exploit the wealth of information embedded in biological systems to refine predictions. This is vital for aiding clinicians in determining the most efficient and least harmful therapeutic choices, facilitating a more intelligent selection and monitoring of patients participating in clinical trials, and contributing to the ongoing evolution of personalized medicine (Simon, 2013; Relling and Evans, 2015; Aronson and Rehm, 2015). In this context, we built predictive models using three different types of signatures, encompassing molecular, biochemical, and IHC features. One of the primary objectives was to assess which signature type might be predictive of treatment response in ML models. In addition to the complete models, we constructed several reduced models by employing the greedy forward sequential feature selection (SFS) approach with random forests as the estimator, we aimed to identify the smallest set of features that best represent each model. The selected molecular features were miR-1289 and TUBG mRNA, while the biochemical features included ALT and TG, and the IHC features comprised GSTP.

Our study showed that the molecular model consistently achieved the highest MCC values when considering each data type individually—Molecular, Biochemical, and IHC—in both complete and reduced models across various classifiers. In reduced models, MCC values for molecular models closely resemble those of complete models and outperform them in the case of biochemical and IHC data across all classifiers except SVM. Integrating multiple data types in complete models generally diminishes performance compared to single-type models. However, integrating molecular data with either biochemical or IHC data enhanced performance compared to individual cases, and in some instances, combining multiple data types slightly improves performance over the best individual data type, such as with the NN classifier. Reduced models combining molecular data consistently perform well, as the greedy SFS approach favored the important molecular features over IHC and biochemical features. Also, the ROC curve and confusion matrix for the reduced model employing the RF classifier with molecular features showed an average AUC of 0.96 ± 0.04 from three cross-validation iterations. Additionally, this model accurately predicts 138 responsive and 36 non-responsive samples out of the total samples highlighting its robustness.

Furthermore, we employed a rigorous analysis approach to select potential biomarkers that exhibit promising treatment responses, closely resembling the normal response. We focused on features with *p*-values exceeding the predetermined alpha threshold when comparing individual features of each data type against the control group, meaning that the control samples and the feature samples might come from the same population. These features were considered highly promising due to their ability to bring the treatment response closer to that of the normal response. The results revealed a total of seven highly promising molecular responses within our drug spectrum that exhibited statistically insignificant results when compared to the control model. Among these responses, three were lncRNAs, two were miRNAs, and two were mRNAs. Specifically, lncRNA-RP11-513I15.6 demonstrated its potential as a marker for Hesperidin-200, Cyan (at 10 and 20 mg doses), and Pantoprazole-100. MiR-125b showcased promise for Hesperidin-50 and Pantoprazole-25, while LNC-RP11-583F2.2 exhibited potential for Hesperidin-100 and pantoprazole-50. Furthermore, lncRNA-MALAT displayed significant associations with Hesperidin-200 and Cyan-30, miR-125b was linked to Hesperidin-50 and Pantoprazole-25, miR-106b showed potential with Cyan-30, and *TUBG* mRNA displayed promise with Cyan-30. Notably, *RAB11* mRNA exhibited an interesting response when treated with Hesperidin-50.

In addition to these molecular markers, seven biochemical markers showed as highly predictive candidates (AST, ALP, Albumin, TC, TG, HDL-C, LDL-C) and TNF as important IHC biomarkers. These findings highlight the potential significance of a comprehensive approach that combines multiple types of markers to better understand drug response. A particularly noteworthy finding is that increasing the dosage of the administered drug enhances the likelihood of achieving effective results.

Prior studies have examined the connections between genomic profiles and drug response (Barretina et al., 2012; Garnett et al., 2012). Numerous algorithms for predicting drug sensitivity have been put forth like logistic regression that recognized as a comparative method in multiple studies in drug sensitivity predictions (Daemen et al., 2013; Menden et al., 2013; Costello et al., 2014; Kim et al., 2012). Zeng et al. (2022) developed artificial intelligence models utilizing digital histological images that demonstrate the capability to predict the activation of multiple immune and inflammatory gene signatures with an AUC > 0.8. Riddick et al. (2011) developed a multistep algorithm to predict *in vitro* drug sensitivity using gene expression data from the NCI60 panel. Their approach, tested on 19 breast cancer cell lines, involved feature selection, elimination of outlying cell lines, and training random forest regression models. The authors concluded that their algorithm surpassed existing techniques in predicting drug sensitivity. Daemen et al. (2013) drug sensitivity data for 138 drugs and molecular profiles from 70 breast cancer cell lines. This data was utilized to train models that classify samples into two categories: those responding well to treatment and those responding poorly. Subsequently, the models were validated using independent patient-derived data, focusing on two drugs commonly employed in breast cancer treatment tamoxifen and valproic acid. In ovarian cancer, Chen et al. (2015) demonstrated the possible significance of a 61-transcript expression pattern in anticipating a patient’s reaction to platinum-taxane chemotherapy. Importantly, when this expression pattern was integrated with the BRCA1/2 mutation status, a conventional prognostic marker in this context, it resulted in improved patient stratification based on clinical response.

While current models cannot fully replicate the complexities of human disease, significant progress has been made in developing HCC animal models (Fornari et al., 2022). These models are essential for foundational research, enabling more convenient and effective studies of disease mechanisms, therapeutic targets, and drug screening (Liao et al., 2024). The creation of models capable of monitoring the onset, progression, and reversibility of drug-induced toxicity remains a crucial objective, as they are invaluable for designing safer and more effective drugs (Blidisel et al., 2021).

This study has limitations due to the small sample size, and our investigation concentrated on a restricted selection of drugs, potentially not capturing the full array of therapeutic options for HCC. Future inquiries encompassing a wider variety of medications and treatment approaches could offer a more thorough insight into effective treatment methodologies for this intricate condition. Further studies are imperative to evaluate the efficacy of the chosen mRNA-miRNA-LncRNA-Circ-RNA in HCC with diverse etiologies. Since we solely examined these molecular markers in animal specimens, it’s essential to corroborate these findings through multicenter clinical trials involving human subjects. Expanding our research to include human samples will significantly enhance the relevance and applicability of our findings. We recommend more studies to be held in this context to significantly enhance the relevance and applicability of our findings. Training our current machine learning models on preclinical datasets and validating them using human clinical data will allow us to assess and refine the models’ predictive performance in a real-world setting.

## 7 Conclusion

In conclusion, our study introduces a machine-learning algorithm capable of accurately predicting the treatment response to three distinct drugs: Pantoprazole, Cyan, and Hesperidin. Our approach involved a comprehensive panel that incorporated traditional HCC biochemical, molecular, and IHC features. This integration of multiple markers not only enhanced the reliability and accuracy of the panel but also ensured cost-effectiveness and speed by utilizing RT-PCR in addition to conventional testing methods instead of more expensive high-profile approaches (Cohen et al., 2018).

## Data Availability

The datasets presented in this study can be found in online repositories. The names of the repository/repositories and accession number(s) can be found in the article/Supplementary Material.
