# Supplementary material for: Integrating molecular, biochemical, and immunohistochemical features as predictors of hepatocellular carcinoma drug response using machine-learning algorithms
Source: Front Mol Biosci. 2024 Oct 16;11:1430794. doi: 10.3389/fmolb.2024.1430794 (PMC11521808; doi:10.3389/fmolb.2024.1430794)

**Table S2. Details of the datasets retrieved from the GEO database.**

| Accession number | Platform | Experiment Title | Organism | Experimental Design | Reference |
| --- | --- | --- | --- | --- | --- |
| GSE141090 | GPL1355[Rat230_2] Affymetrix Rat Genome 230 2.0 Array | The expression landscape of hepatocarcinogeneis in a rat model | Rattus norvegicus | Microarray expression profiling on 6 liver tissues including 3 tissues from the HCC group, and 3 tissues from the normal mock group. | Chen Z, Li S, Shen M, Lu X, Bao C, Chen D, Ding J, Wang Q, Huang S, Cong W, Han L. The mutational and transcriptional landscapes of hepatocarcinogenesis in a rat model. Iscience. 2020 Nov 20;23(11). |
| GSE24600 | GPL7292 Agilent-013328 Rat Oligo Microarray (V2) G4130B (Probe Name version) | Expression profiles dysplastic and neoplastic rat liver lesions | Rattus norvegicus | Gene expression profiles were determined by microarray analysis and validated by quantitative RT-PCR and Western blot. Cluster analysis revealed two distinctive gene expression patterns, the first of which included normal liver of F344 and BN rats and BN rat nodules, and the second one F344 nodules and HCC of both strains. |  |
| GSE49515 | GPL6246 [MoGene-1_0-st] Affymetrix Mouse Gene 1.0 ST Array [transcript (gene) version] | Expression profiling of PBMC from patients with hepatocellular carcinoma | Homo sapiens | Peripheral blood mononuclear cell (PBMC) from healthy individuals, patients with pancreatic carcinoma, gastric carcinoma and HCC were isolated and total RNA was extracted for Affymetrix gene microarray analysis (10 healthy control+10 HCC samples) in Singapore. | Shi M, Chen MS, Sekar K, Tan CK et al. A blood-based three-gene signature for the non-invasive detection of early human hepatocellular carcinoma. Eur J Cancer 2014 Mar;50(5):928-36. PMID: 24332572 |
| GSE38199 | GPL6246 [MoGene-1_0-st] Affymetrix Mouse Gene 1.0 ST Array [transcript (gene) version] | Induction of hepatocellular carcinoma through activation of stromal cells in Pdgf-c transgenic mice | Mus musculus | Two strains of mice, C57BL/6 and C57/BL6 Pdgf-c transgenic, were analyzed to see if liver stromal cells play an essential role in tumorigenesis (8 normal control and 8 HCC) | Wright JH, Johnson MM, Shimizu-Albergine M, Bauer RL et al. Paracrine activation of hepatic stellate cells in platelet-derived growth factor C transgenic mice: evidence for stromal induction of hepatocellular carcinoma. Int J Cancer 2014 Feb 15;134(4):778-88. PMID: 23929039 |
| GSE41804 | GPL570 [HG-U133_Plus_2] Affymetrix Human Genome U133 Plus 2.0 Array | Hepatic gene expression of HCV related Hepatocellular carcinoma and non-cancerous tissue with Il28B rs8099917 TT genotype and TG/GG genotype | Homo sapiens | The molecular feature that was associated with the IL2B genotype was attempted to be clarified by comparing Hepatic gene expression of HCV-related Hepatocellular carcinoma and non-cancerous tissue with Il28B rs8099917 TT genotype and TG/GG genotype. Ten HCC patients were selected from each IL28B genotype, and their gene expression was analyzed using Affymetrix genechip analysis (10 normal control and 30 HCC samples) in Japan. | Hodo Y, Honda M, Tanaka A, Nomura Y et al. Association of interleukin-28B genotype and hepatocellular carcinoma recurrence in patients with chronic hepatitis C. Clin Cancer Res 2013 Apr 1;19(7):1827-37. PMID: 23426277 |

**Supplementary Figures**

**Figure S1. The proof of the expression of the selected genes in the HCC by Expression ATLAS database (available at** [**https://www.ebi.ac.uk/gxa/home**](https://www.ebi.ac.uk/gxa/home)**) and The Cancer Proteome Atlas (TCPA) (available at** [**https://www.tcpaportal.org/**](https://www.tcpaportal.org/)**).**


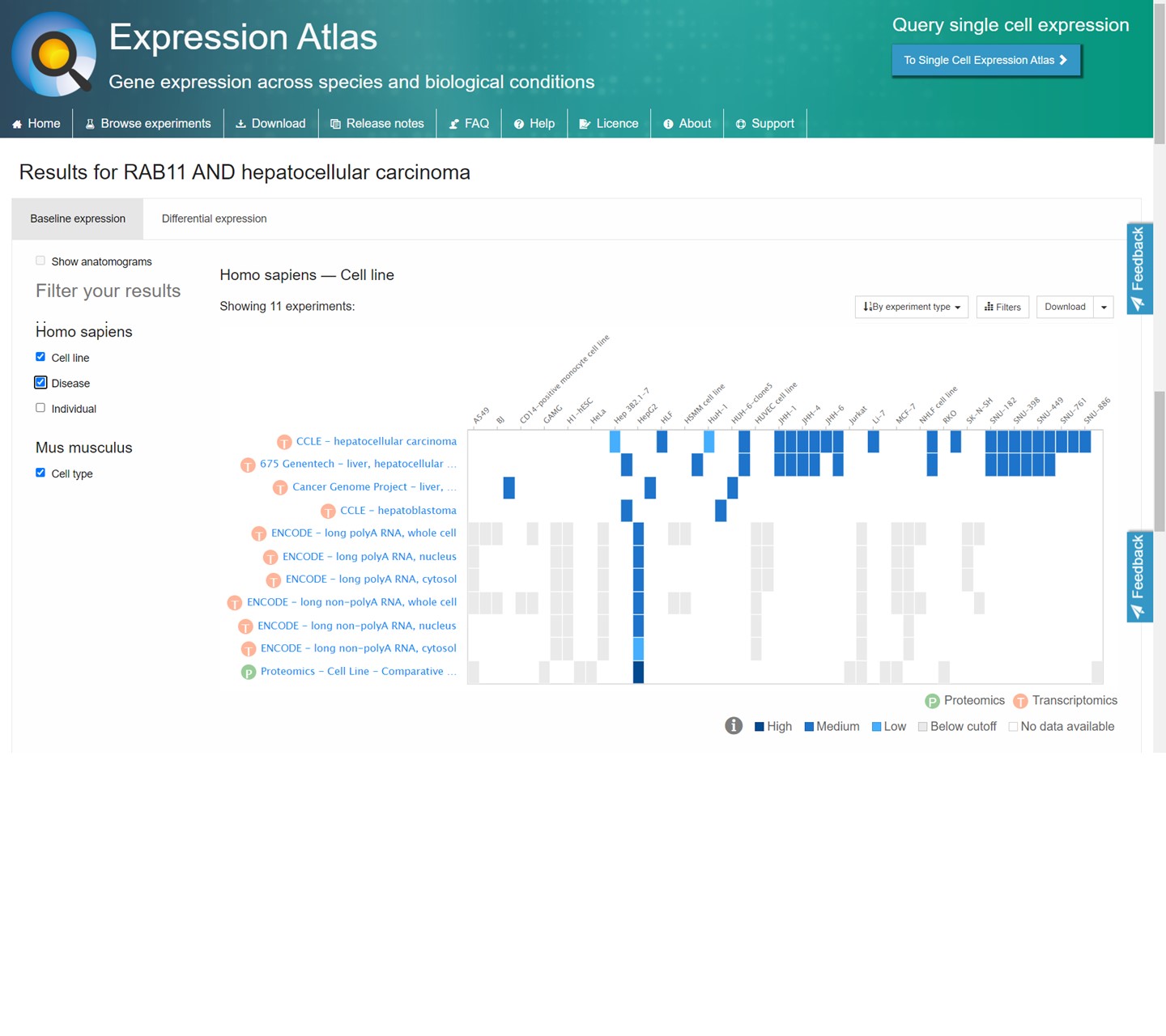


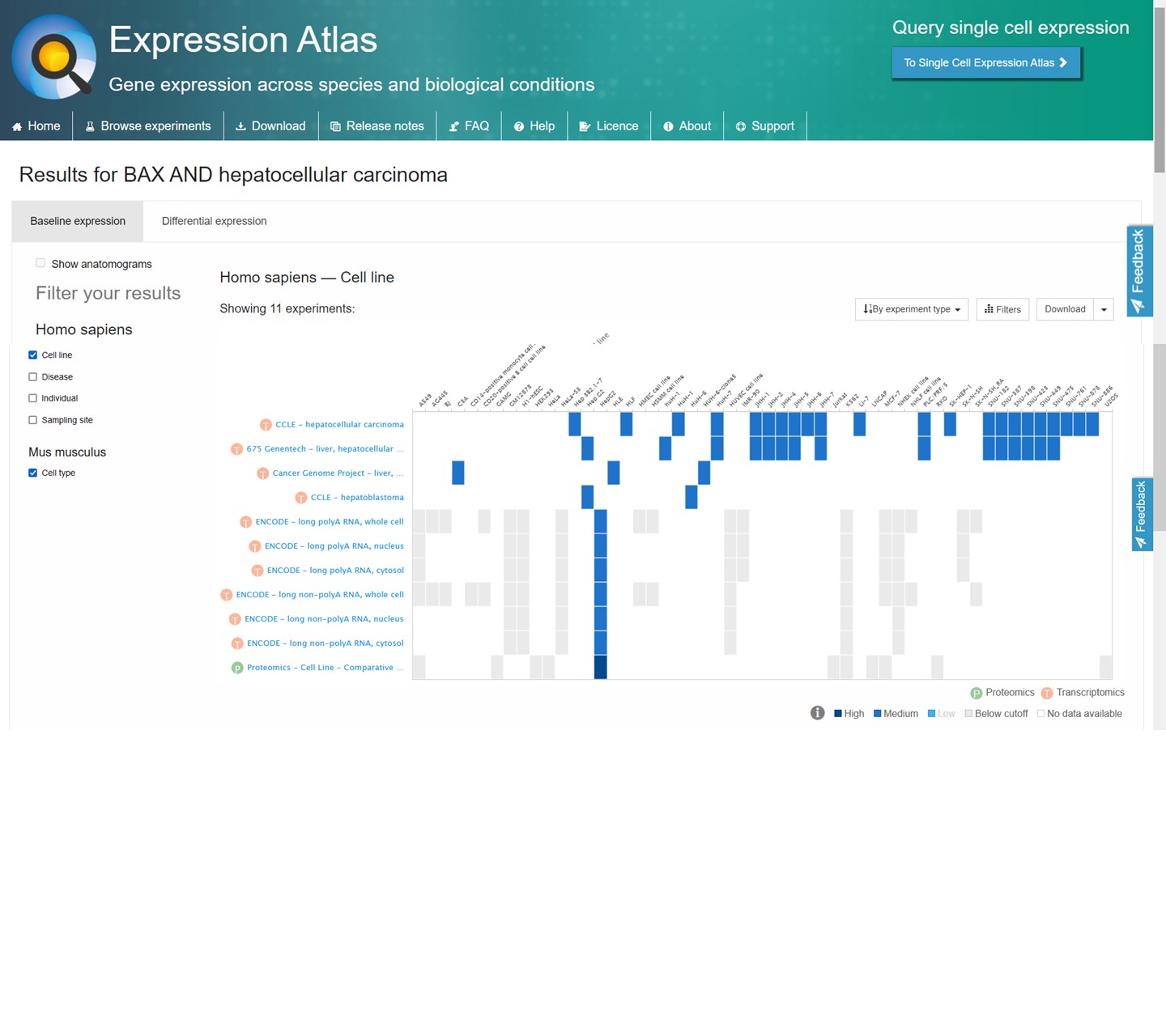


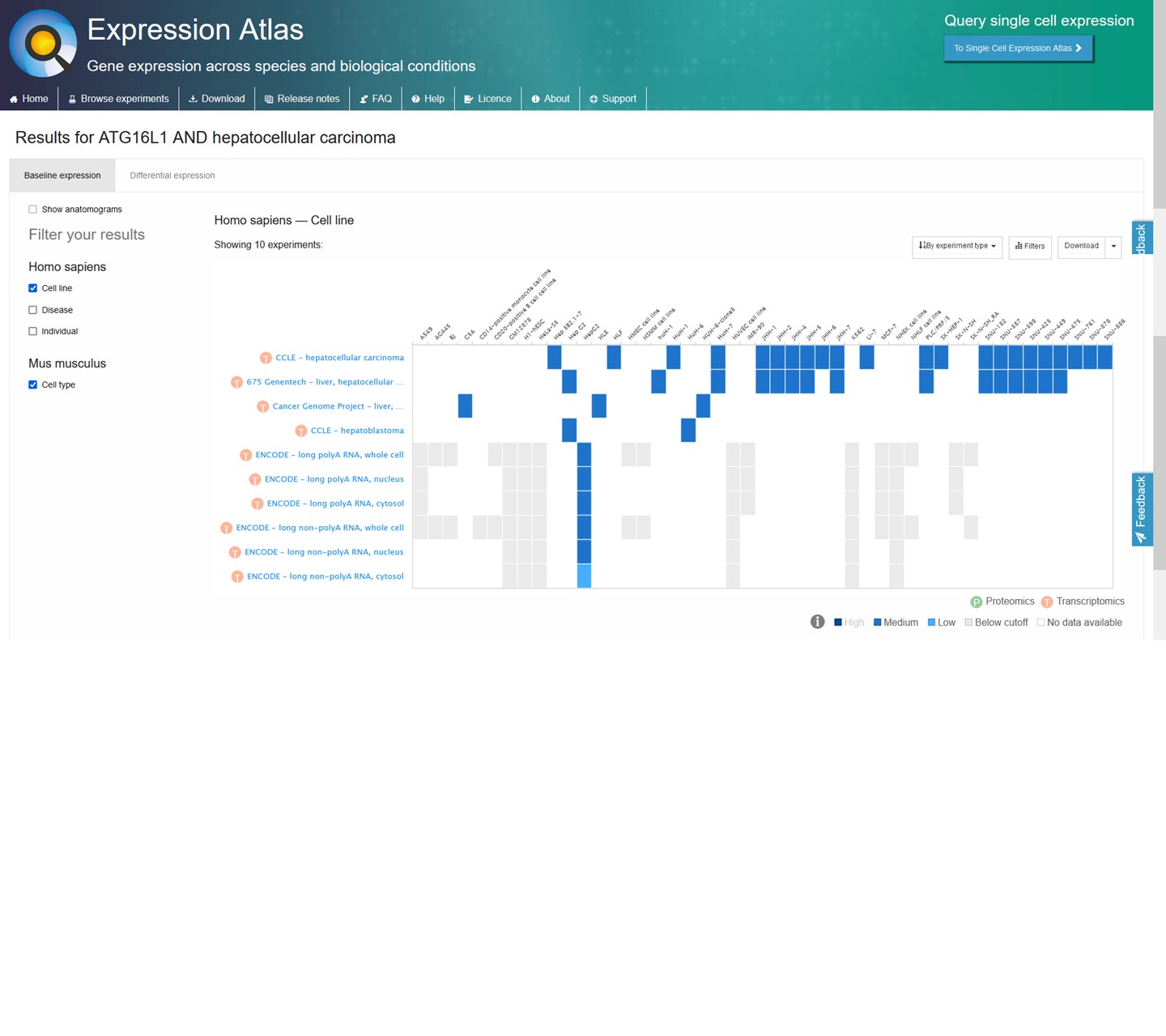


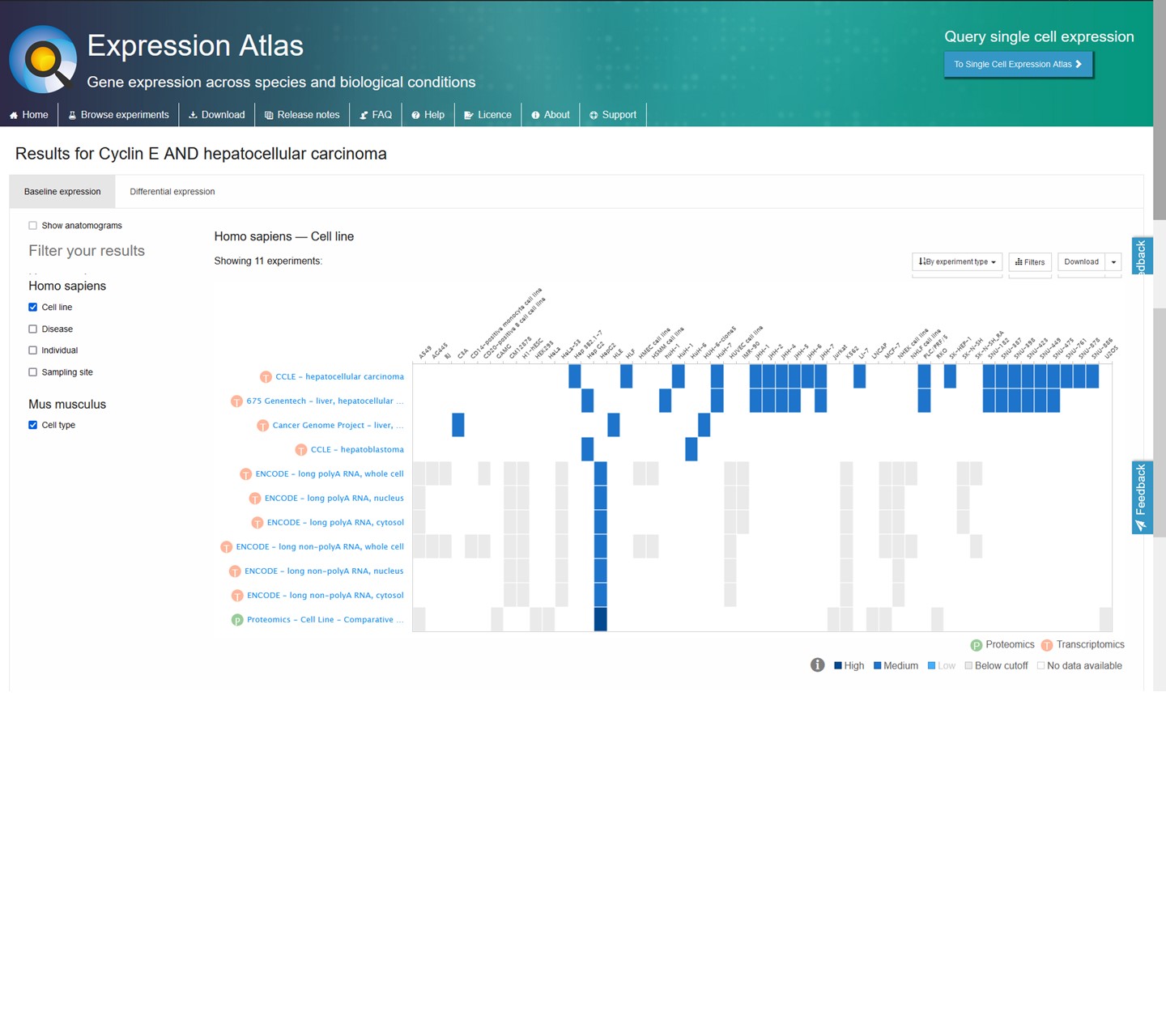


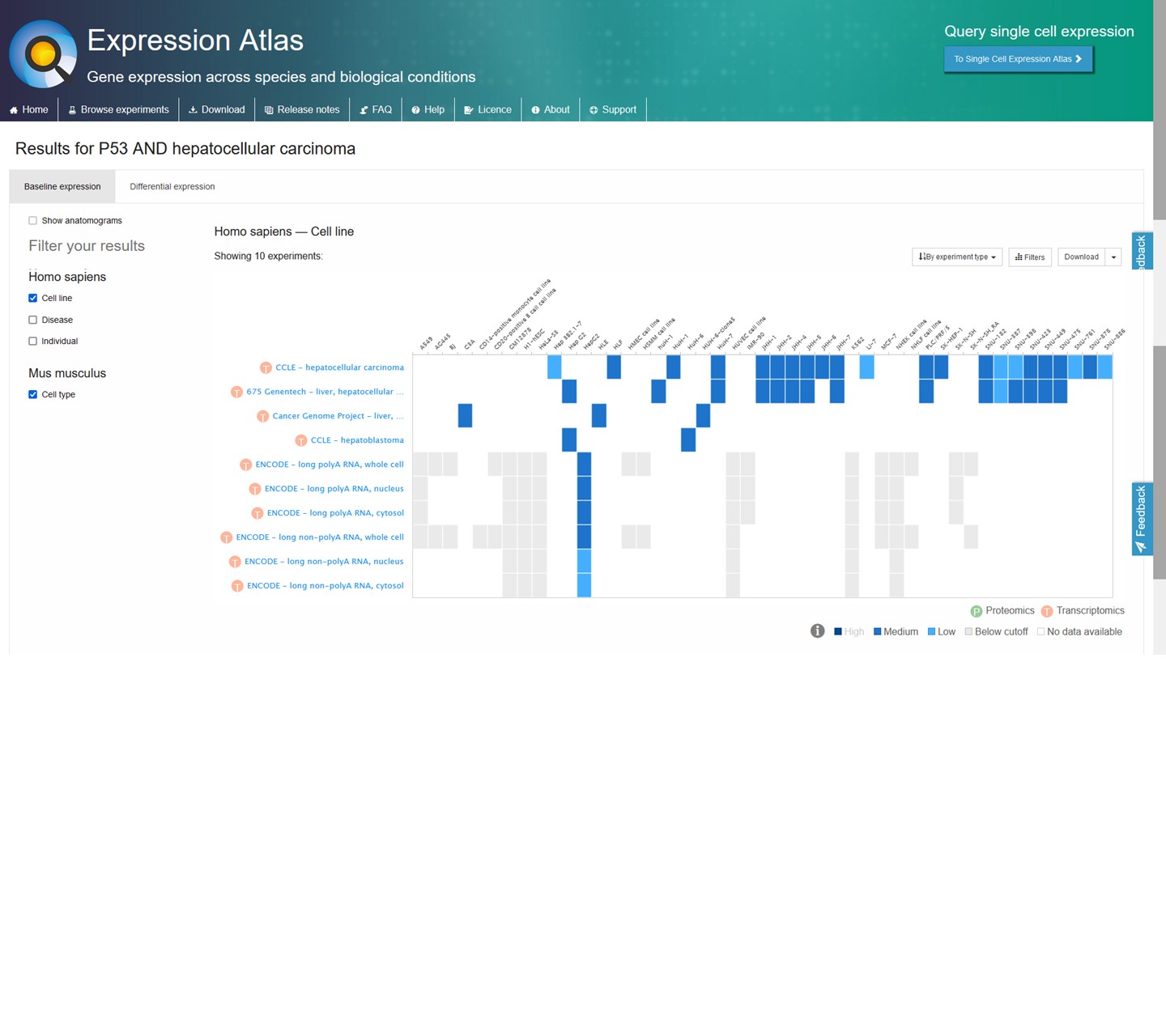


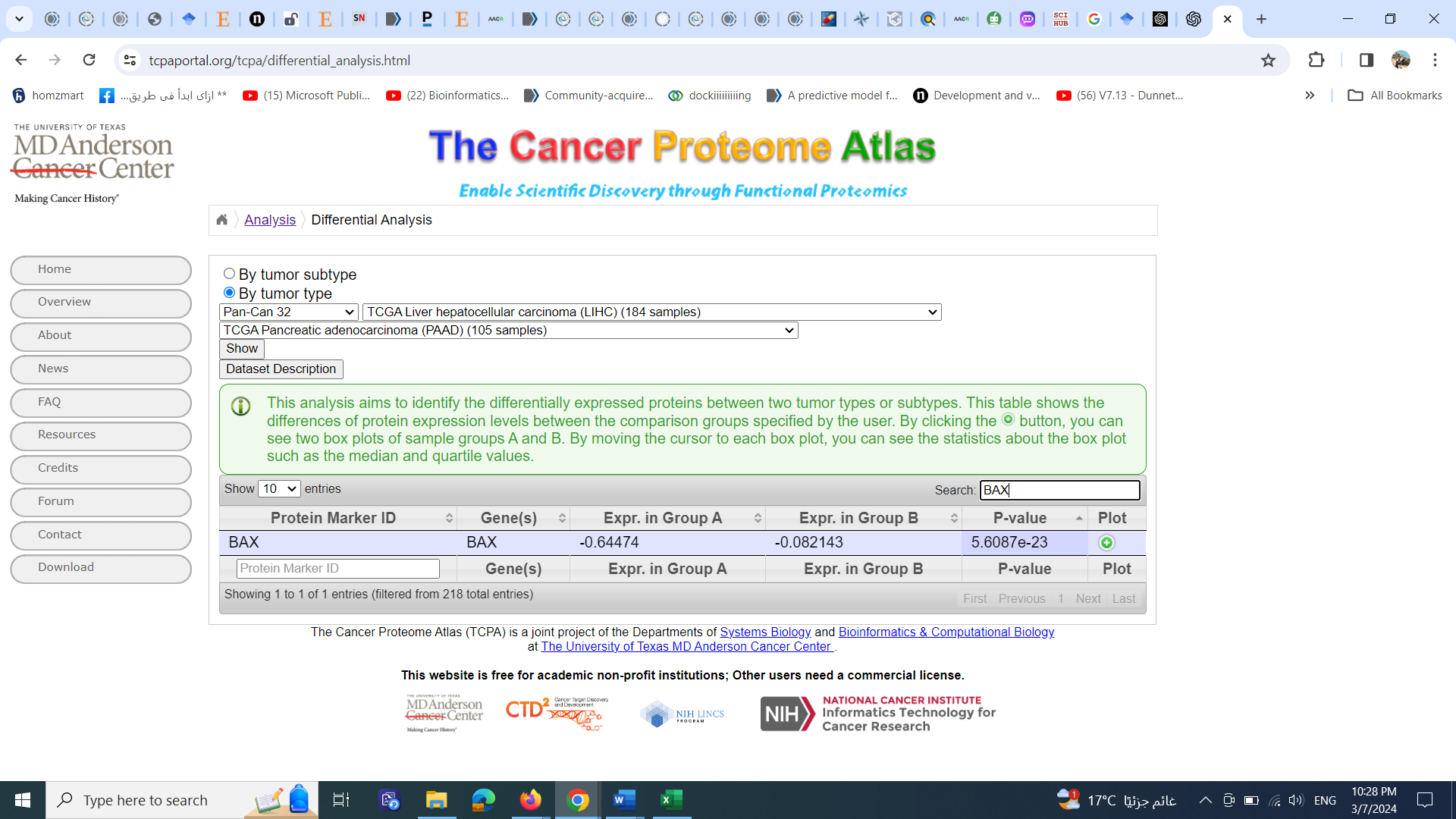


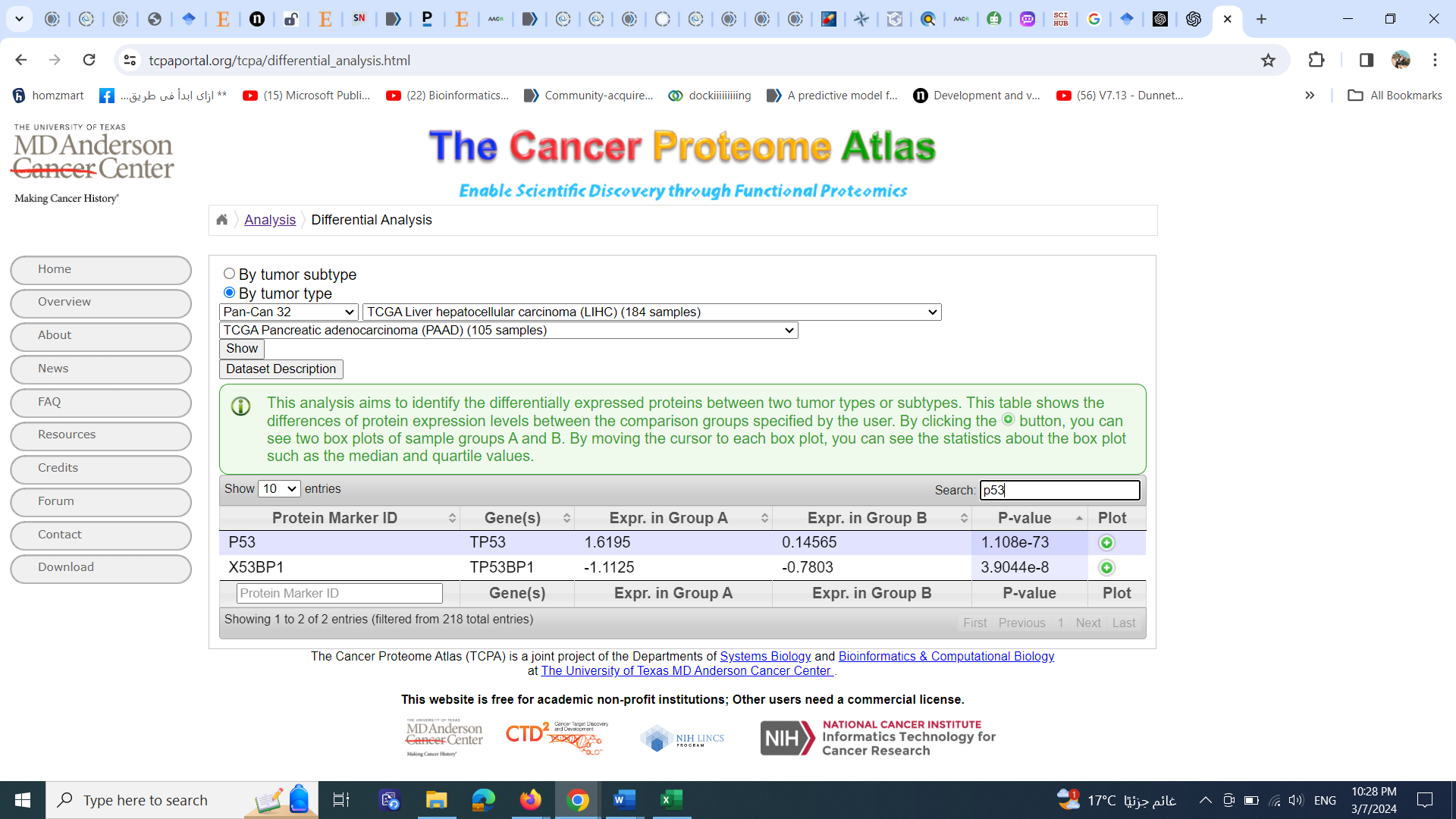


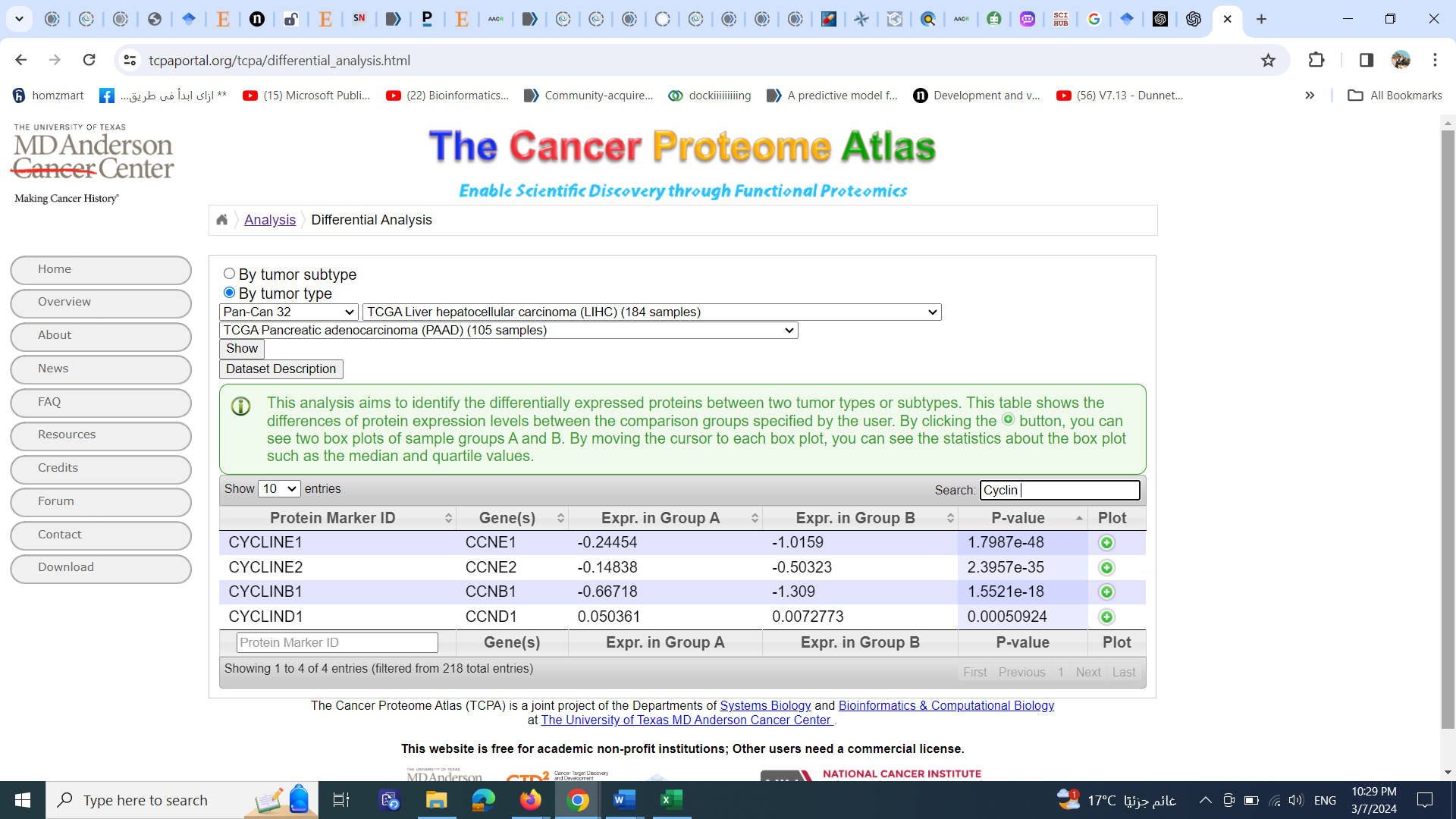


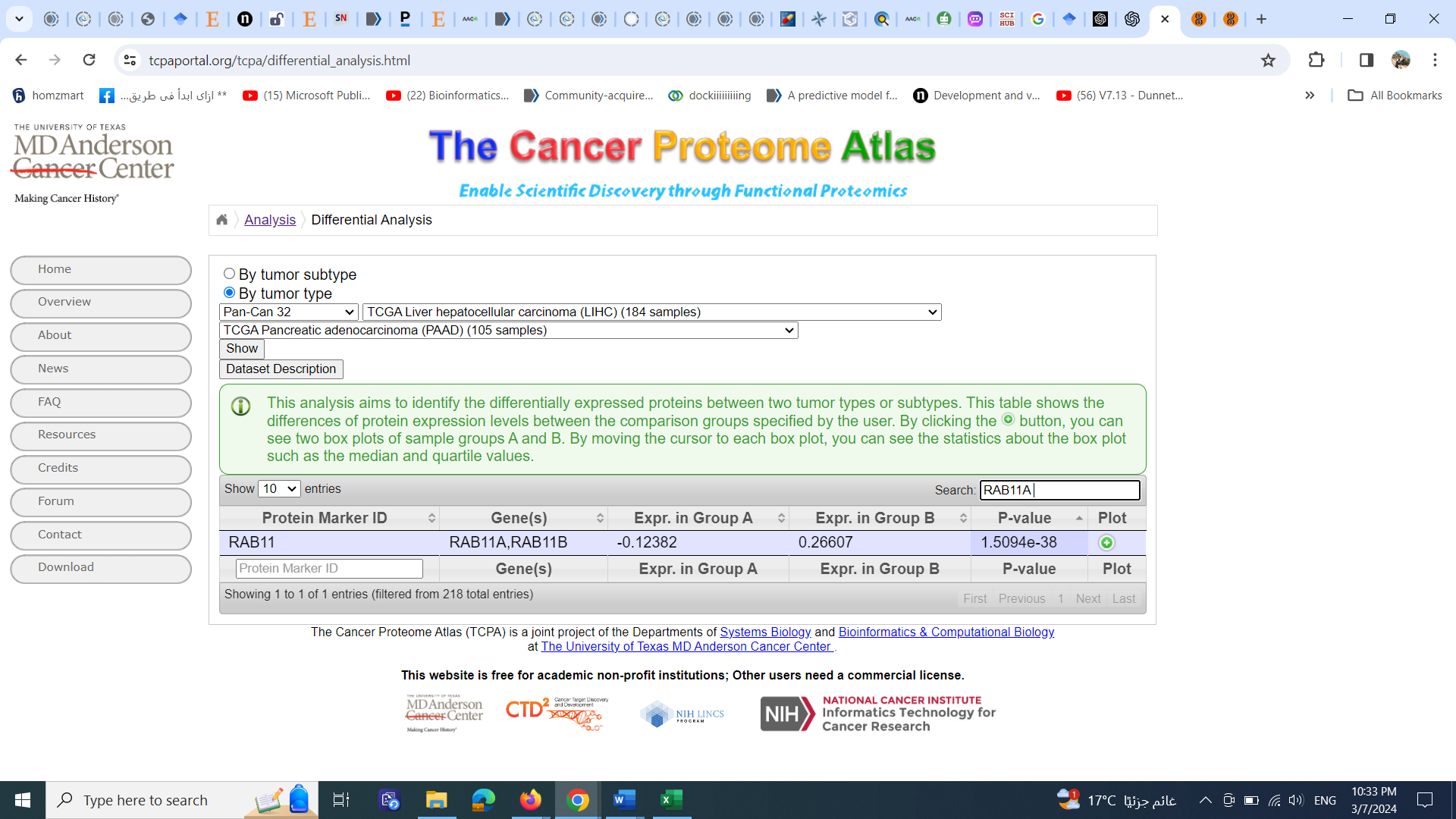


**Figure S2.** **The bar chart of the top 10 enriched KEGG and Reactome pathways for the *BAX*, *P53*,** ***ATG16-L1, TUBG1, Cyclin E*, and *RAB11* network is displayed based on the -log10(p-value), with the actual *P*-value shown next to each term using the Enricher database (**[**https://maayanlab.cloud/Enrichr/**](https://maayanlab.cloud/Enrichr/)**).**


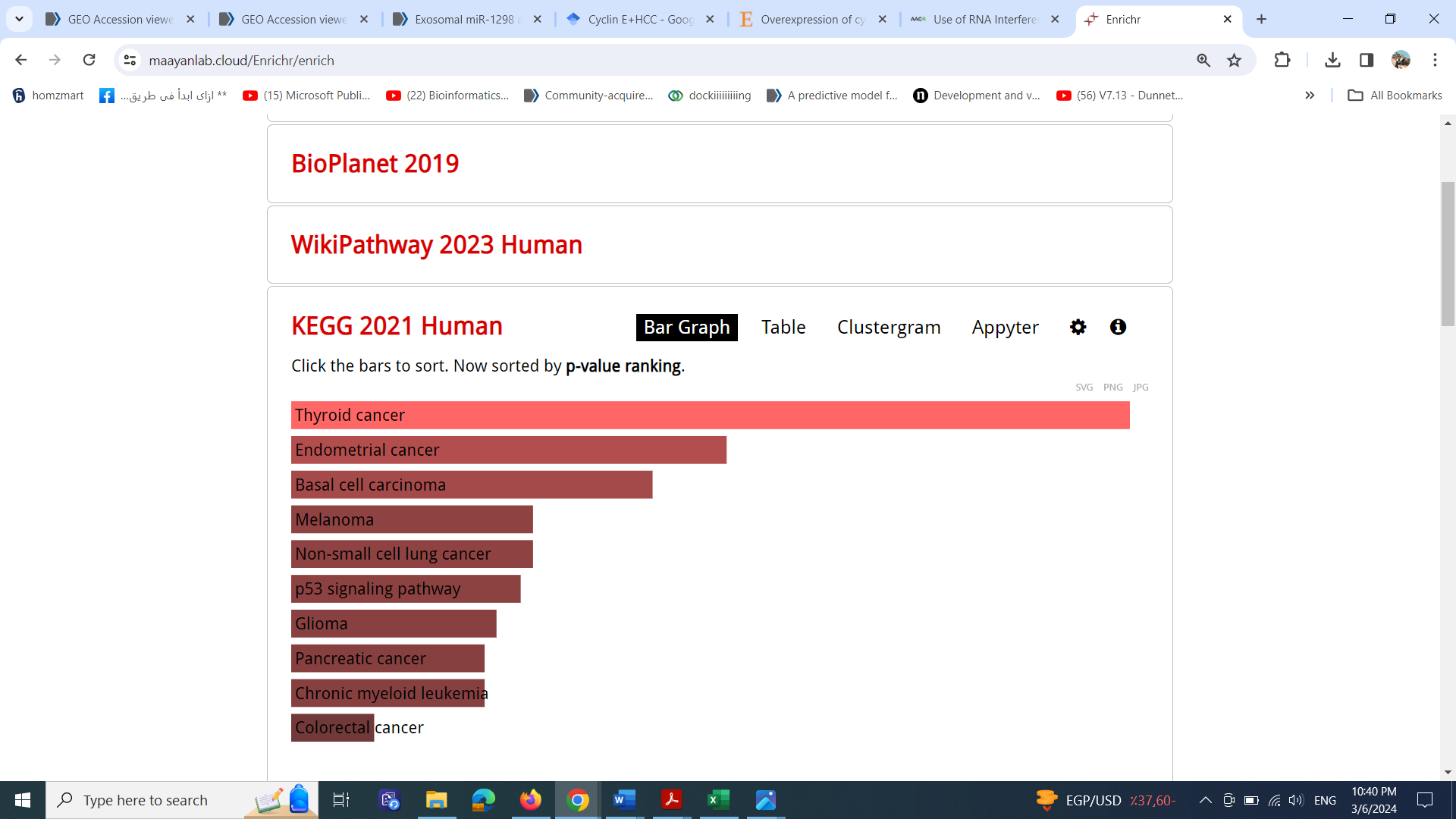


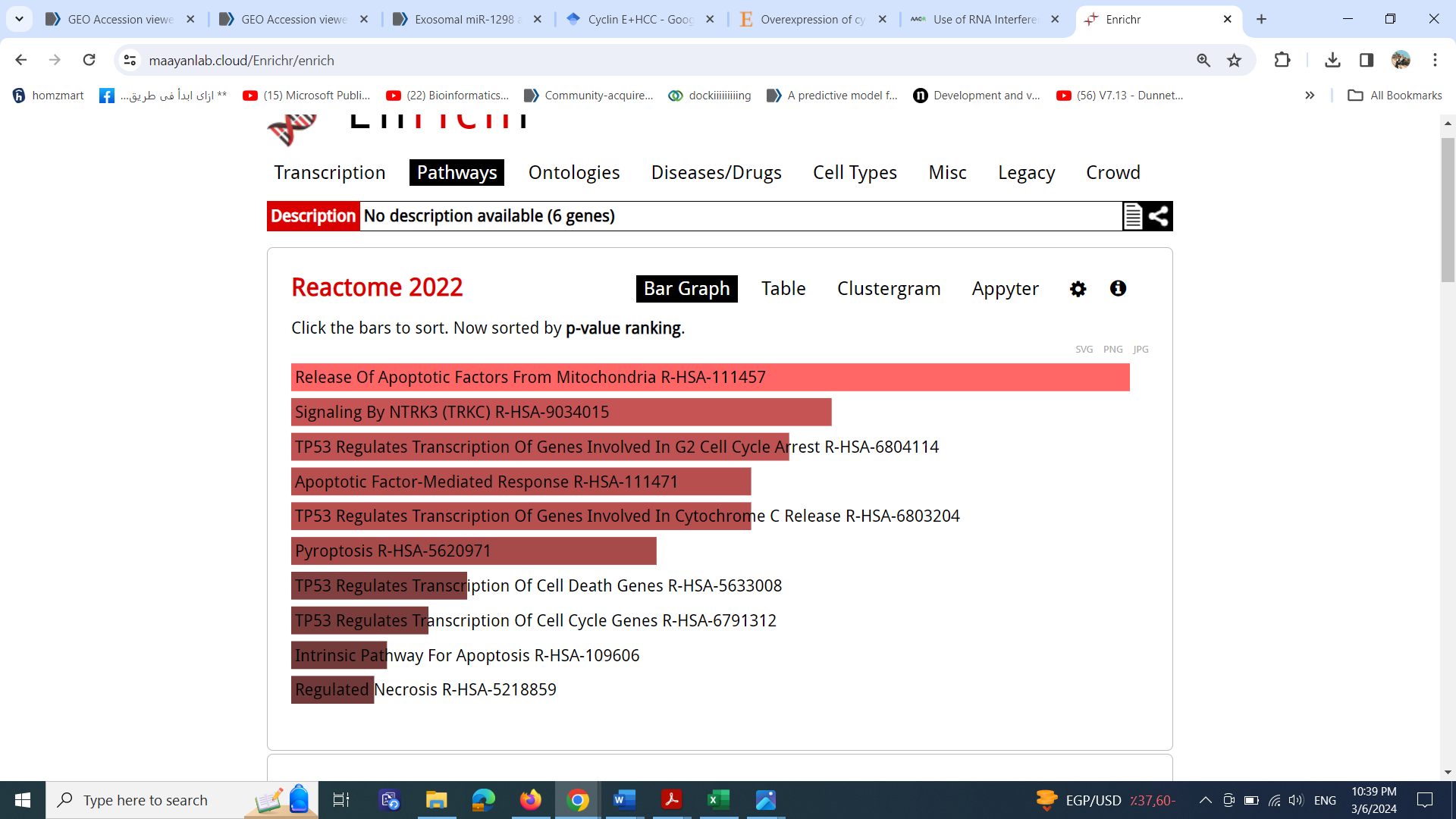


**Figure S3. Represents the interaction between the selected DEGs and miRNAs using the mirwalk database (available at** [**http://mirwalk.umm.uni-heidelberg.de/**](http://mirwalk.umm.uni-heidelberg.de/)**) and the RNA22 database (available at** [**https://cm.jefferson.edu/rna22/Interactive/**](https://cm.jefferson.edu/rna22/Interactive/)**).**


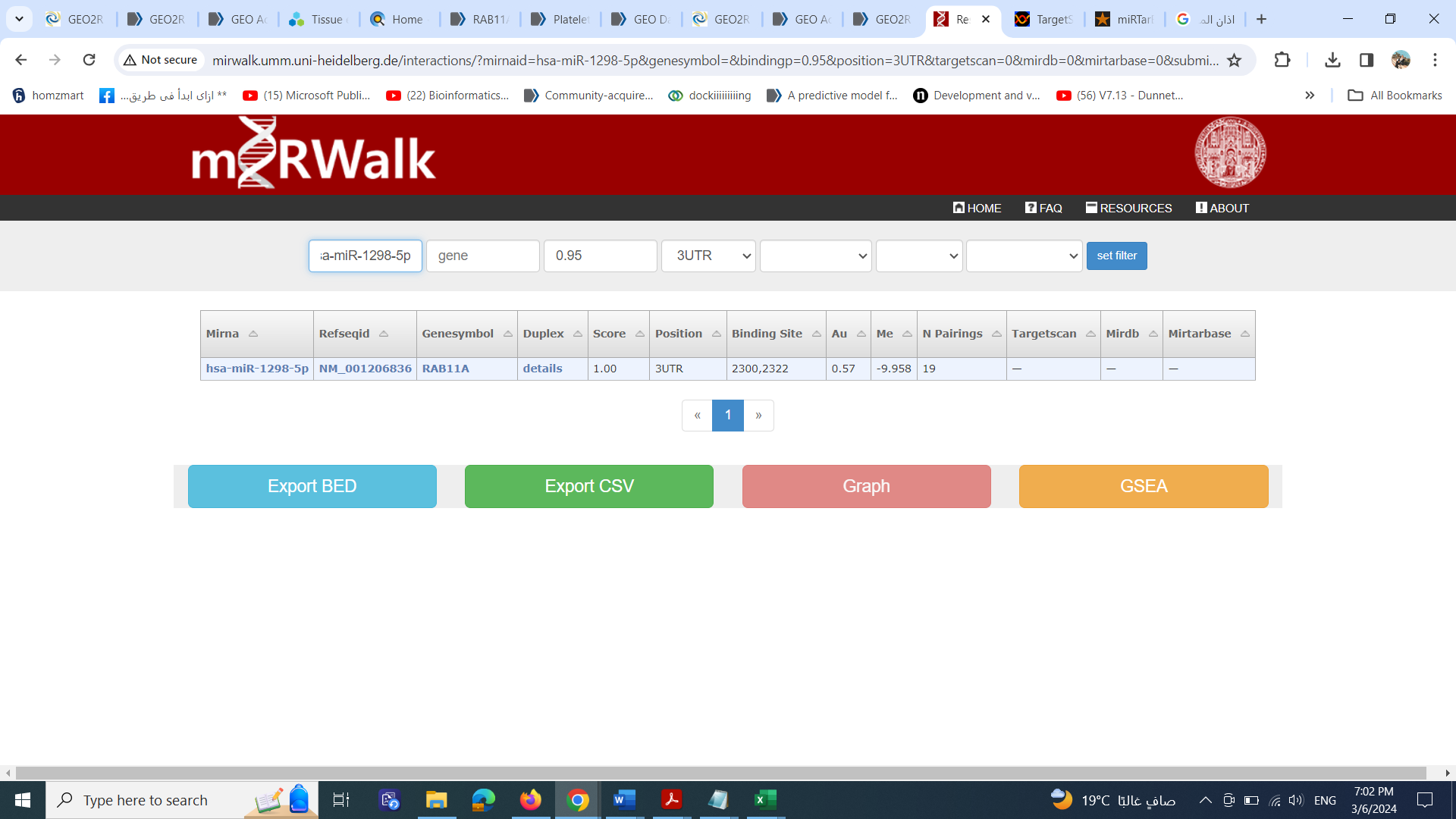


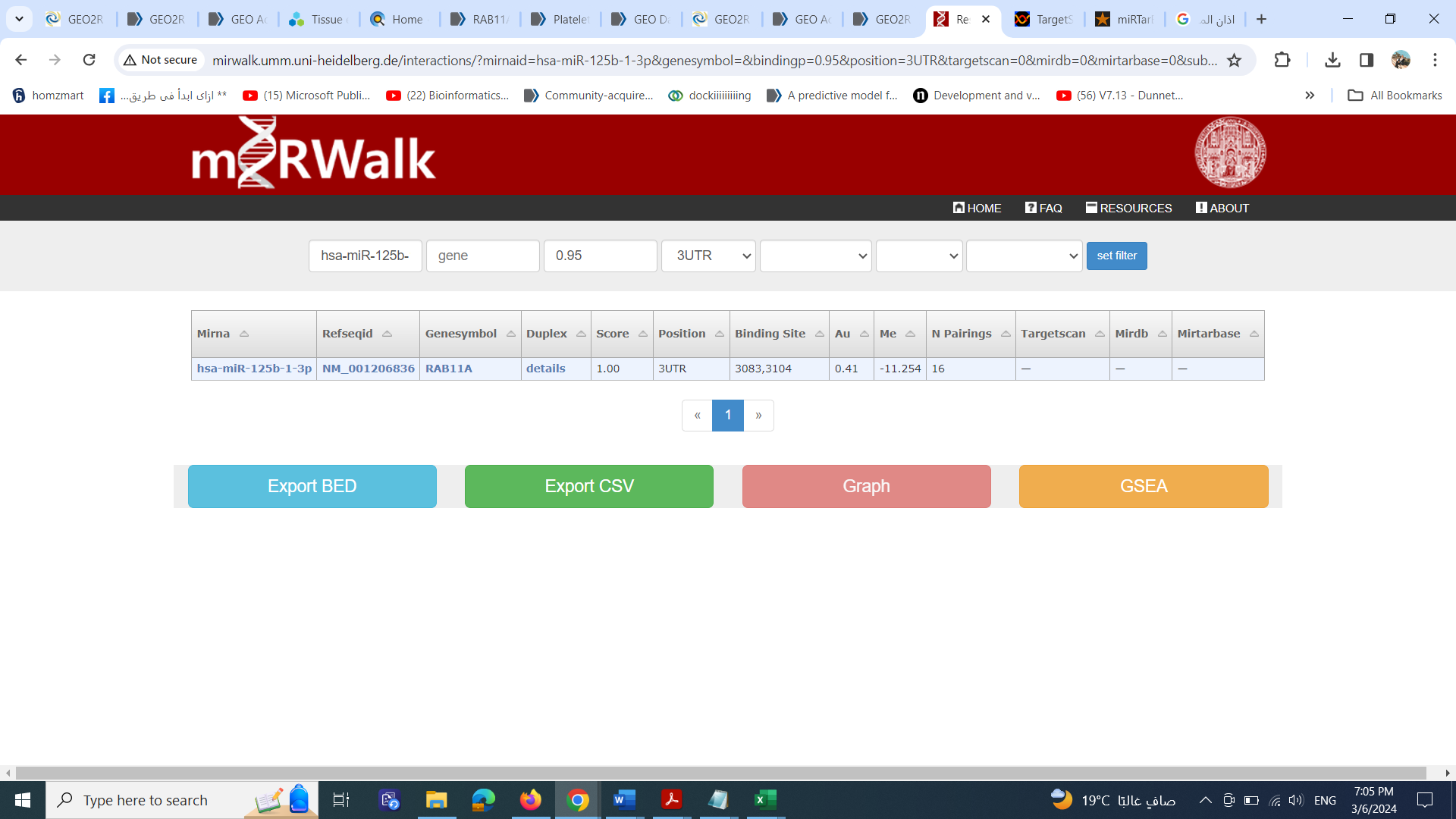


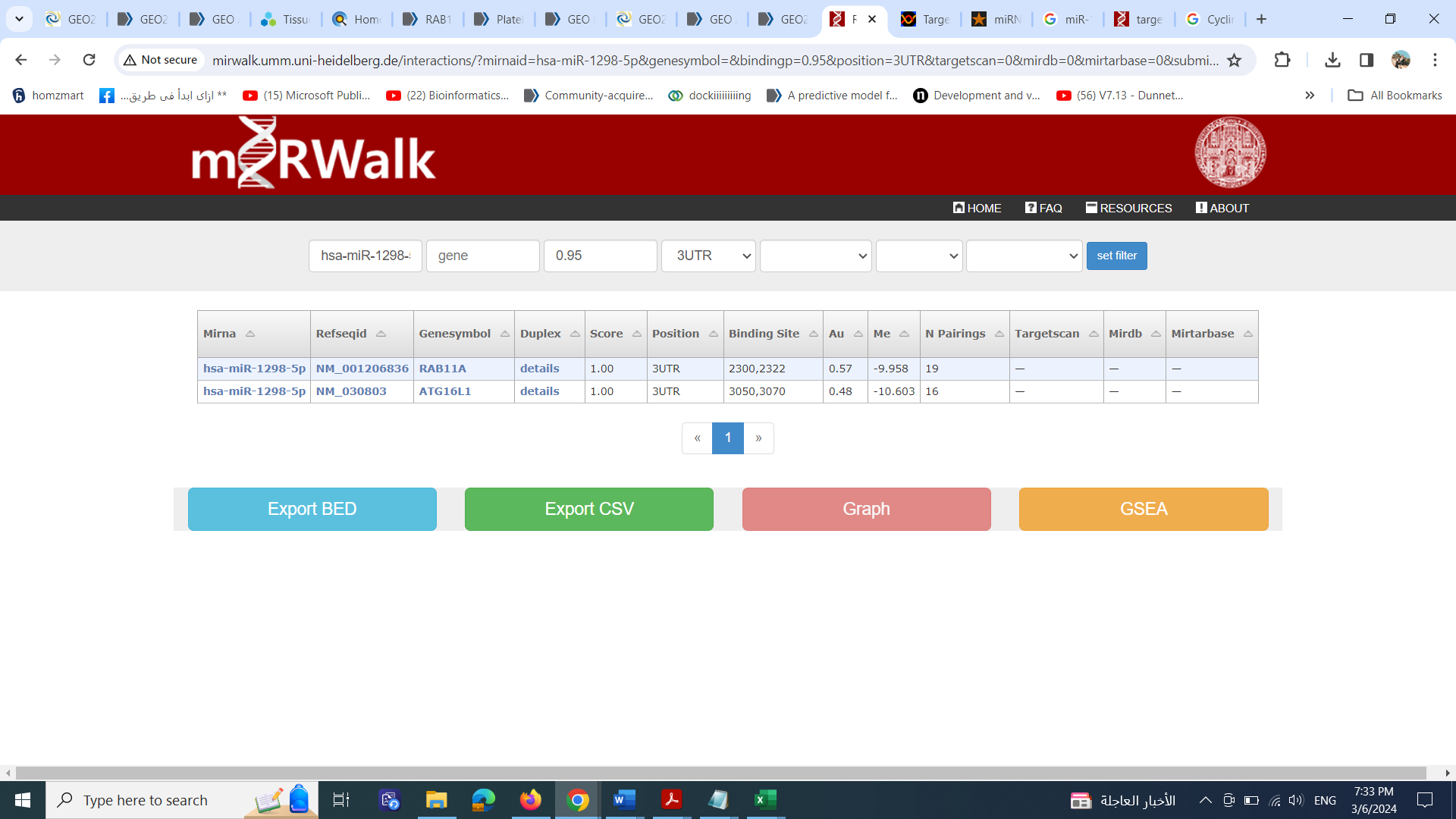


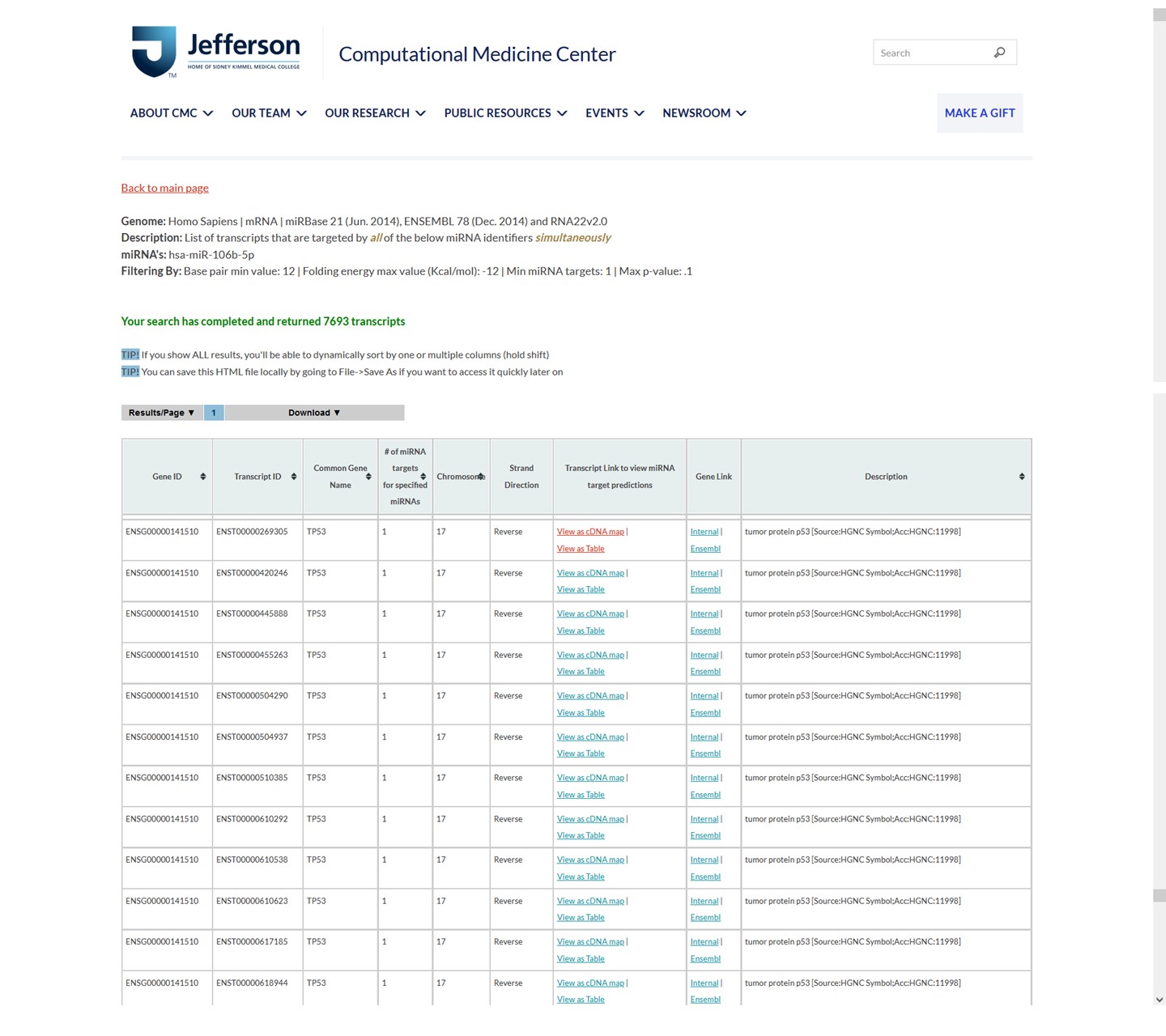


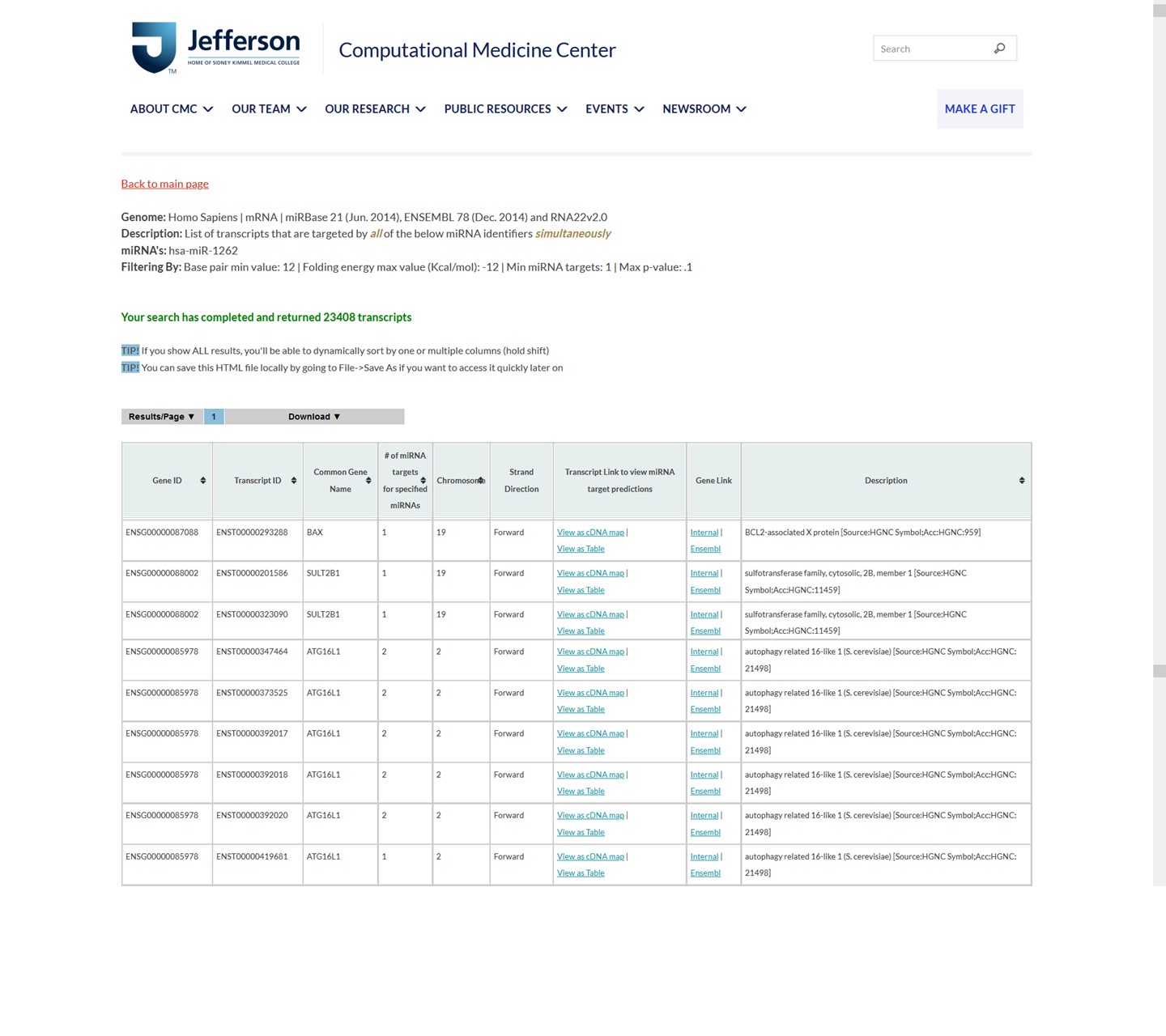


**Figure S4.** **The interaction between the retrieved LncRNAs (MALAT and RP11-513I15.6) and miR‐1298 using the RNA22 database (available at** [**https://cm.jefferson.edu/rna22/Interactive/**](https://cm.jefferson.edu/rna22/Interactive/)**).**


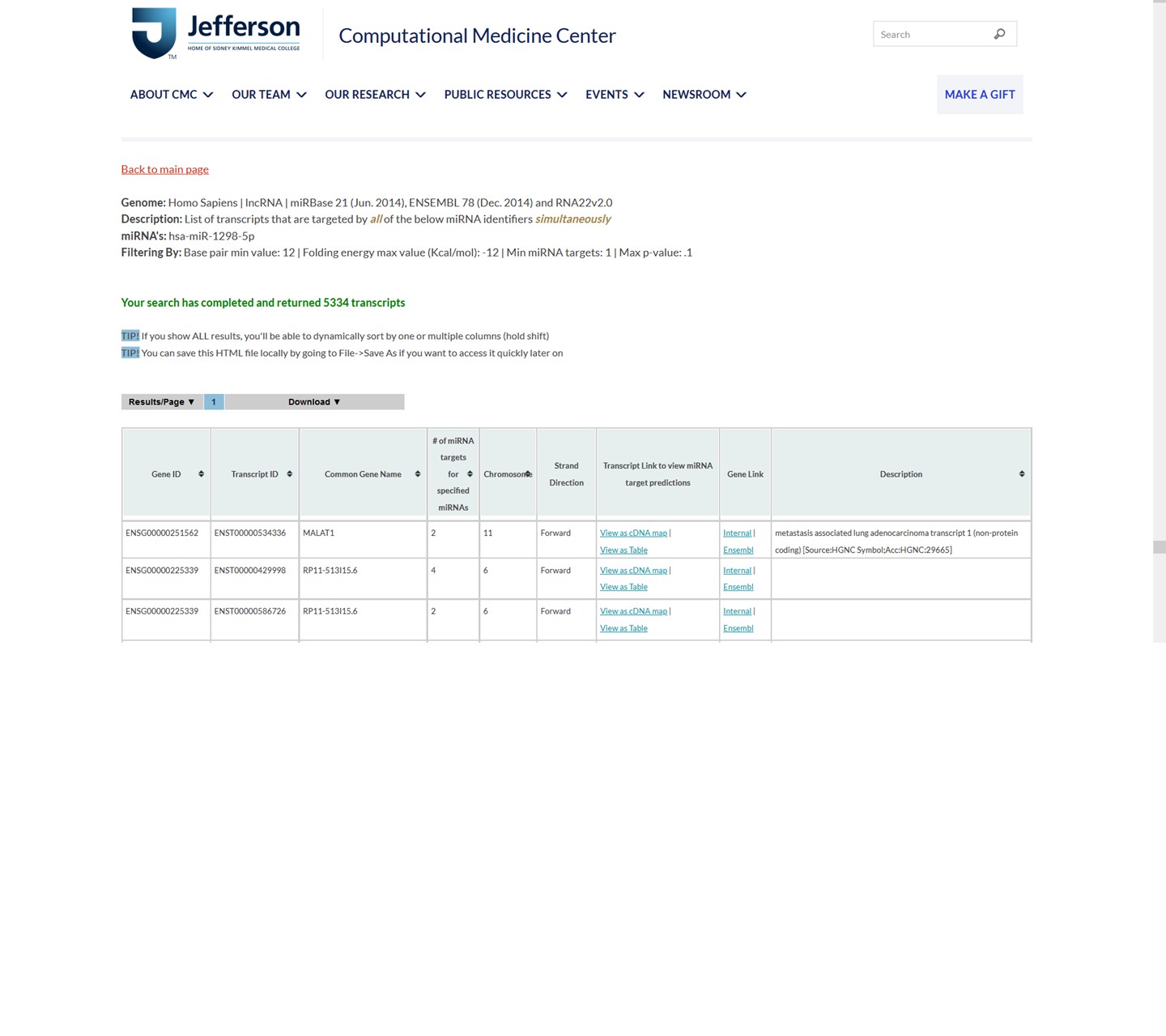


**Figure S5. The validation of the expression of lncRNA-RP11-583F2.2 and** **lncRNA-MALAT in HCC cell lines using the TANTRIC database (available at** [**https://www.tanric.org/**](https://www.tanric.org/)**)**


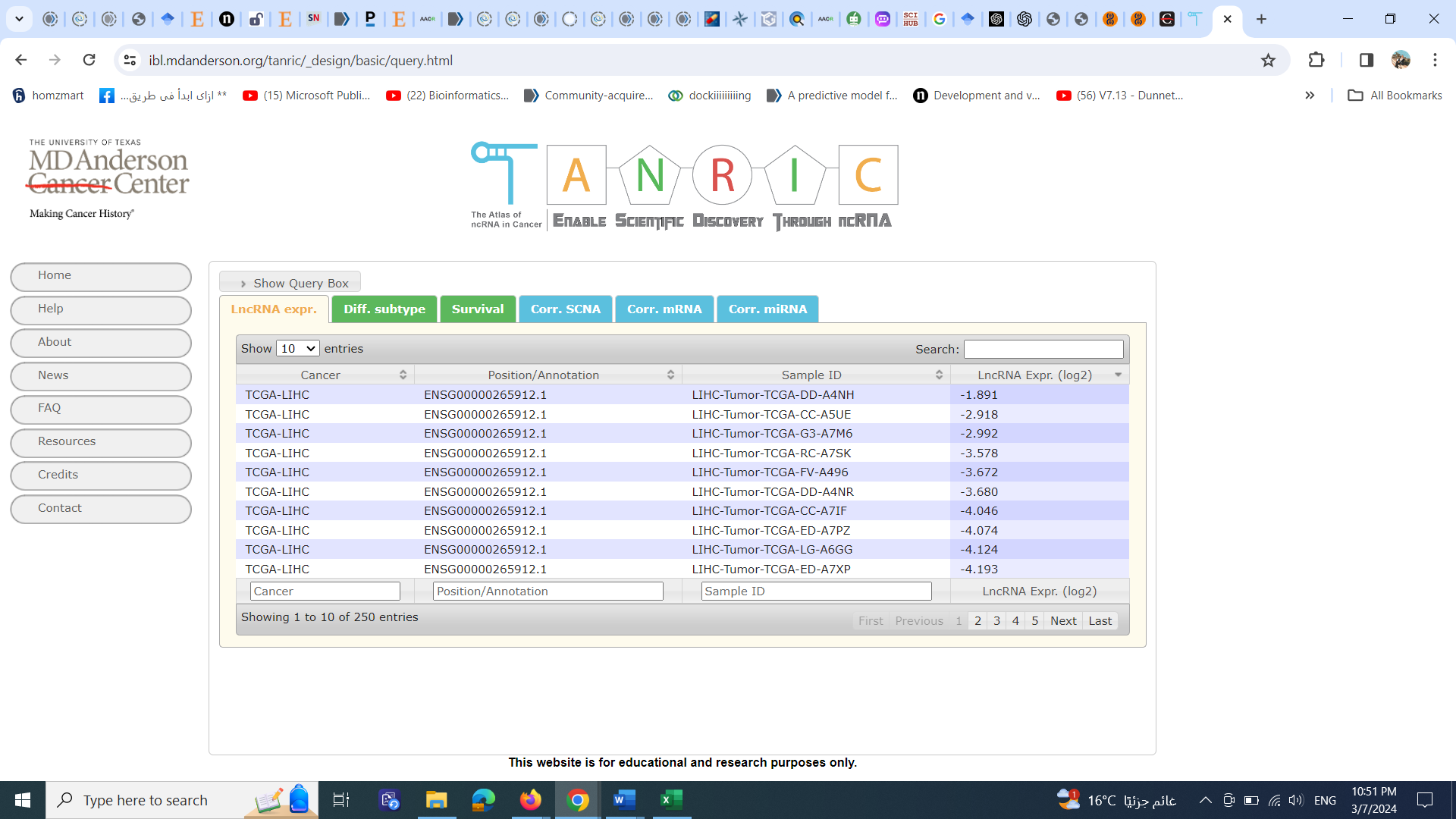


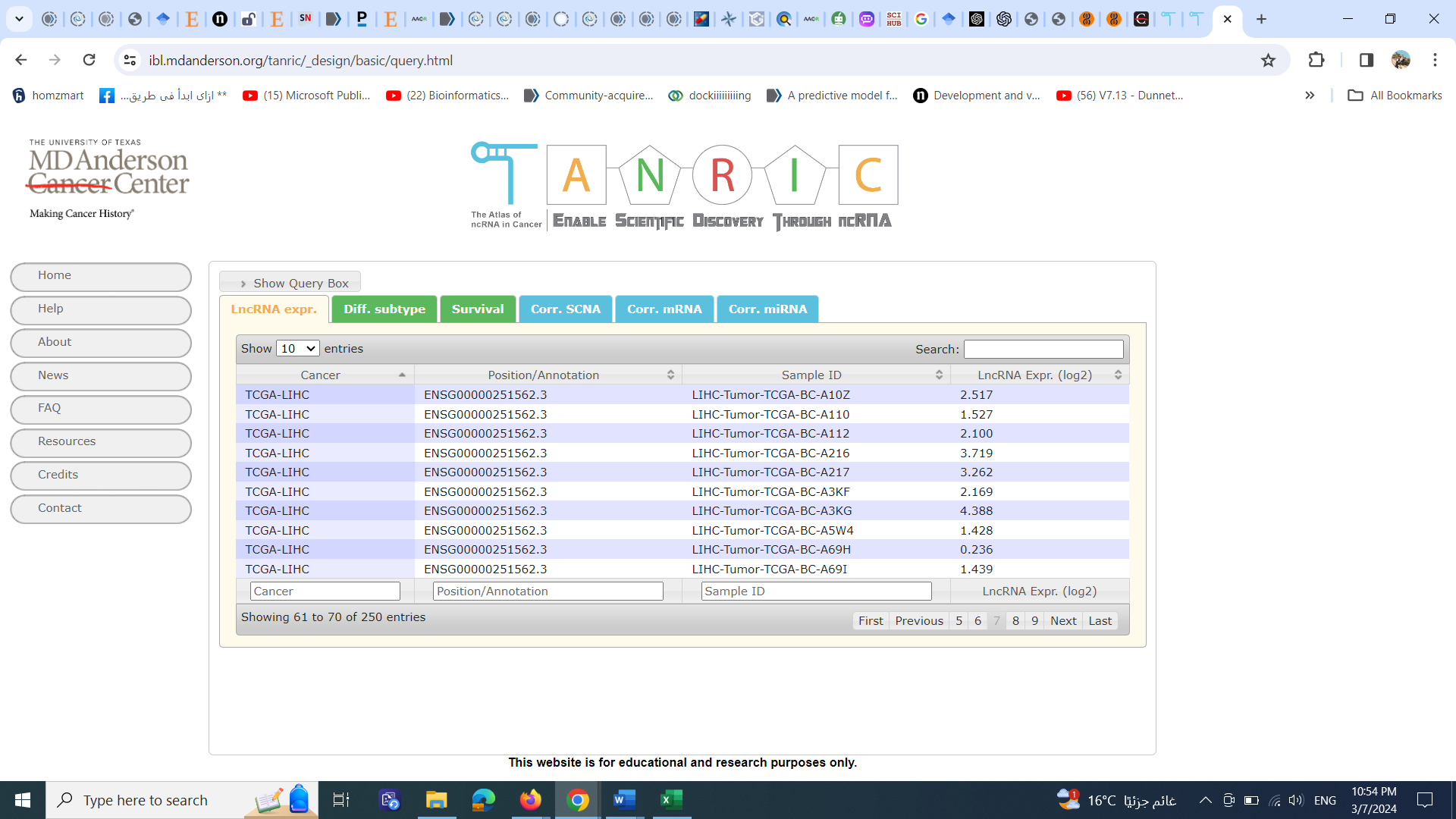


**Figure S6. Confusion Matrices**


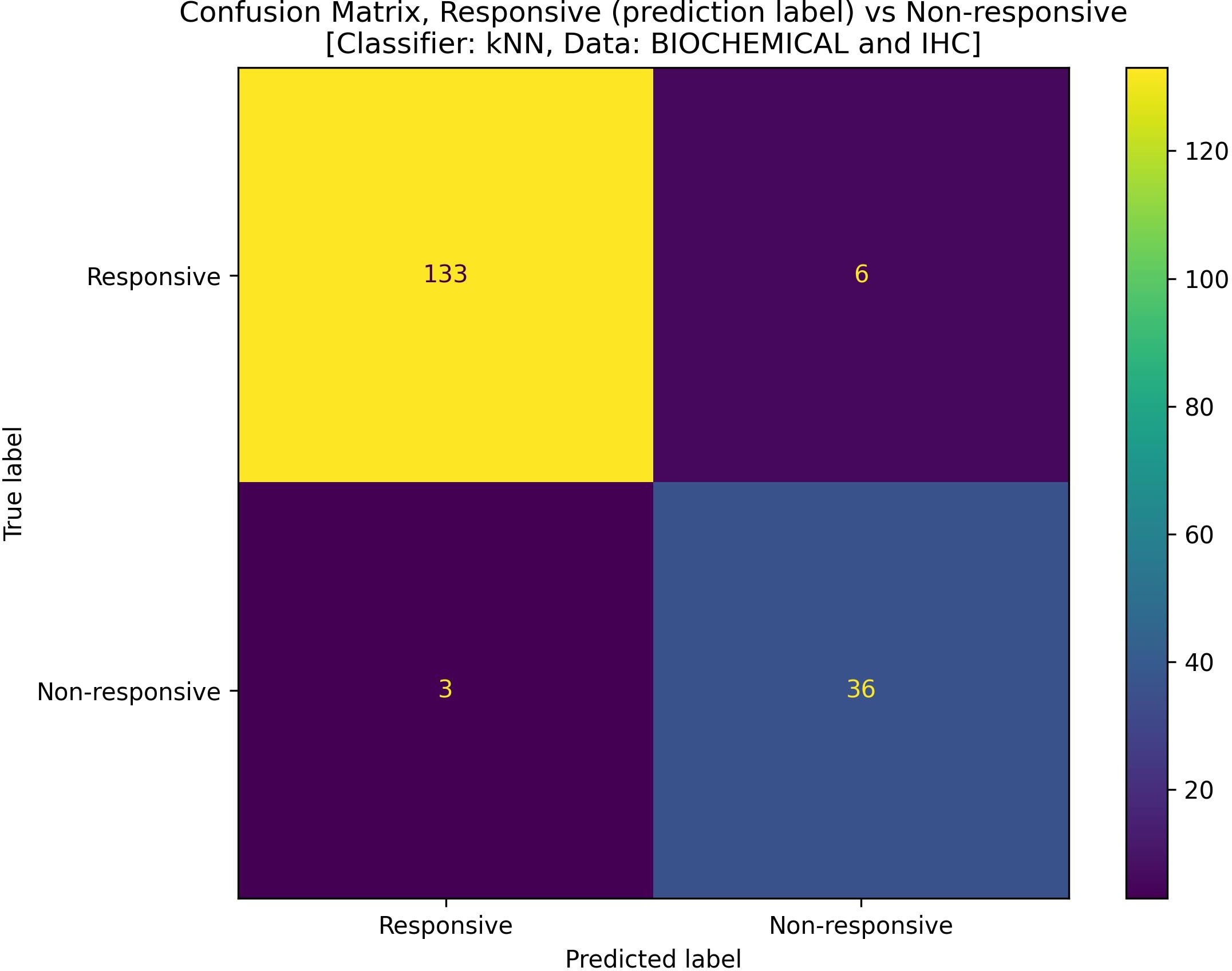


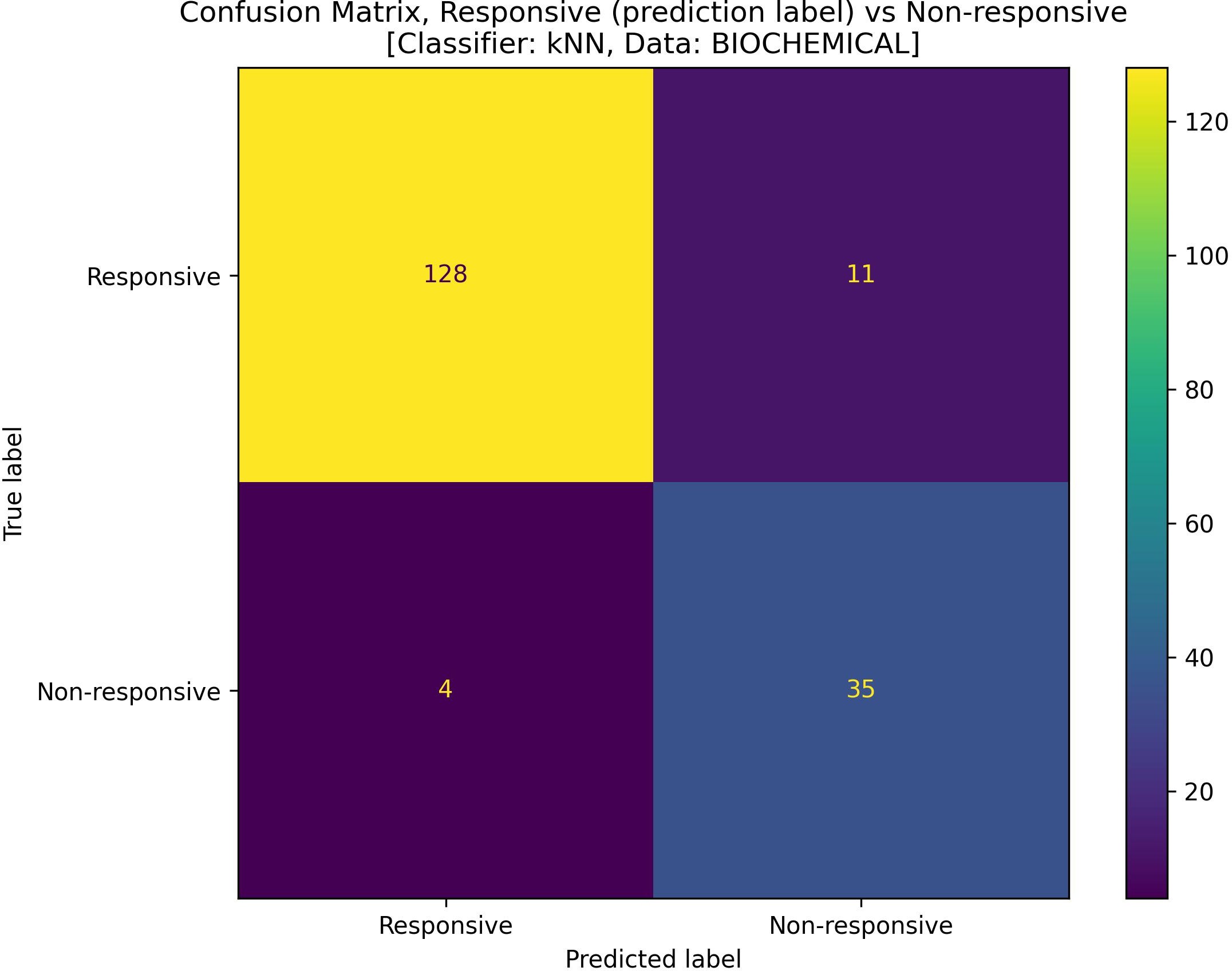


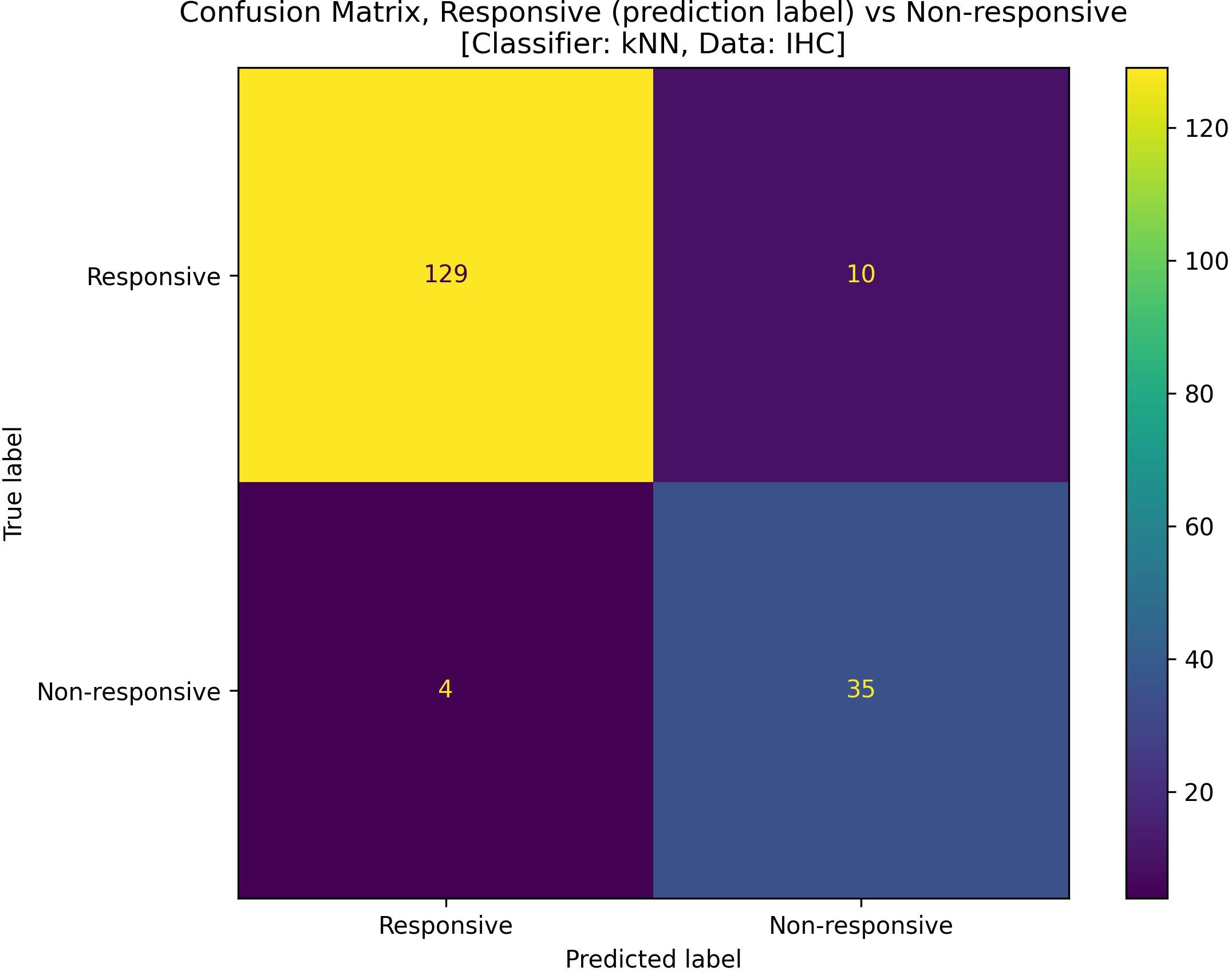


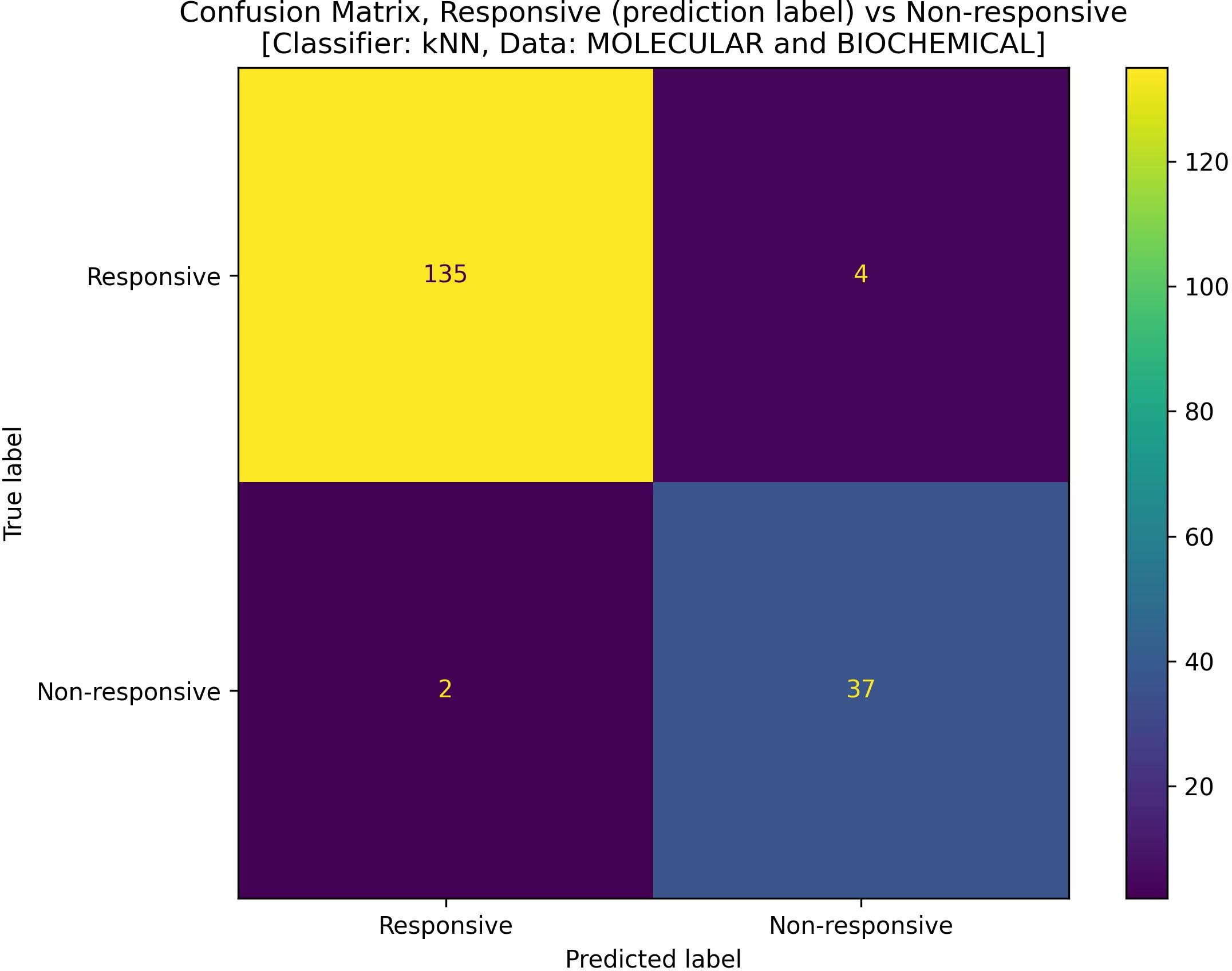


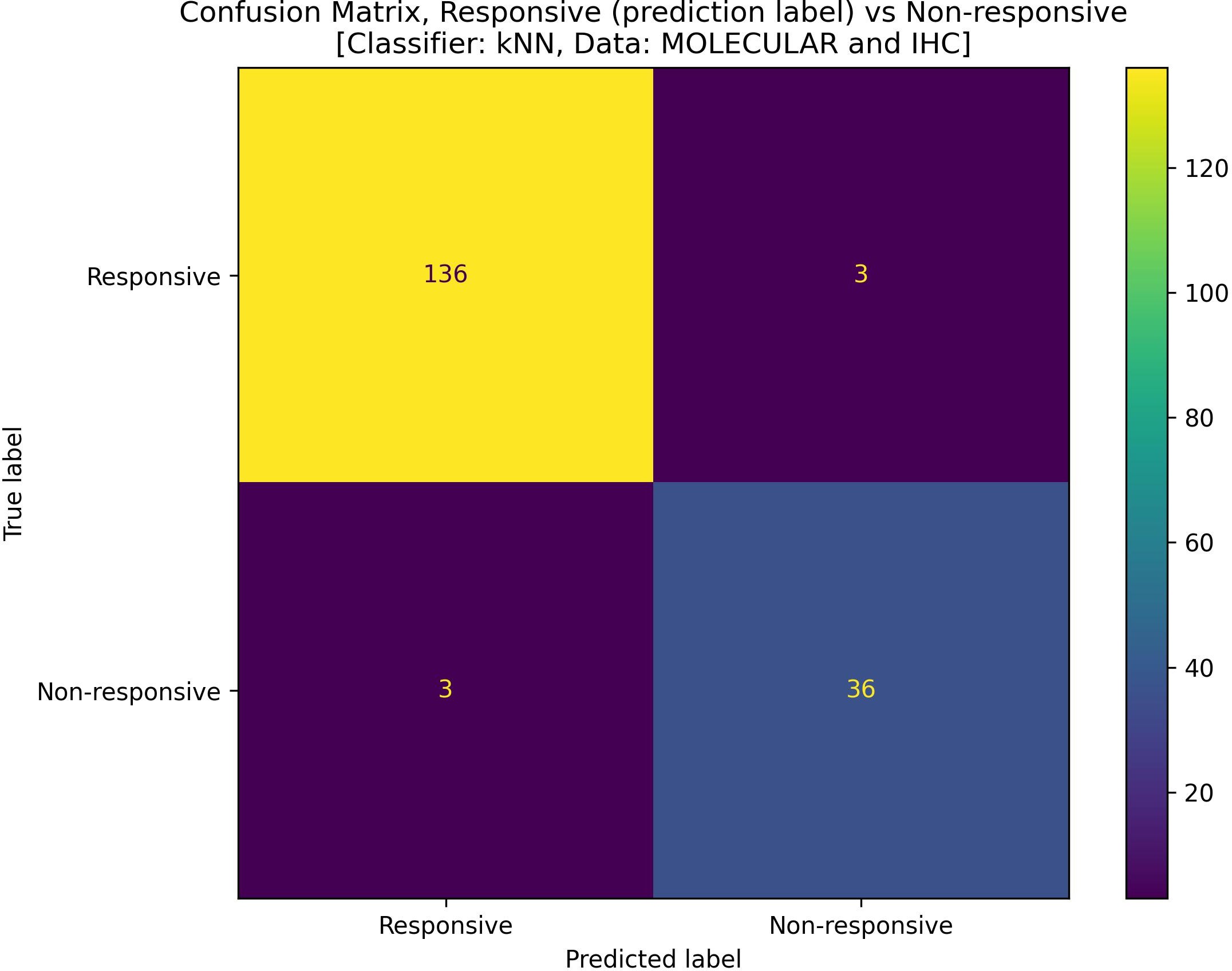


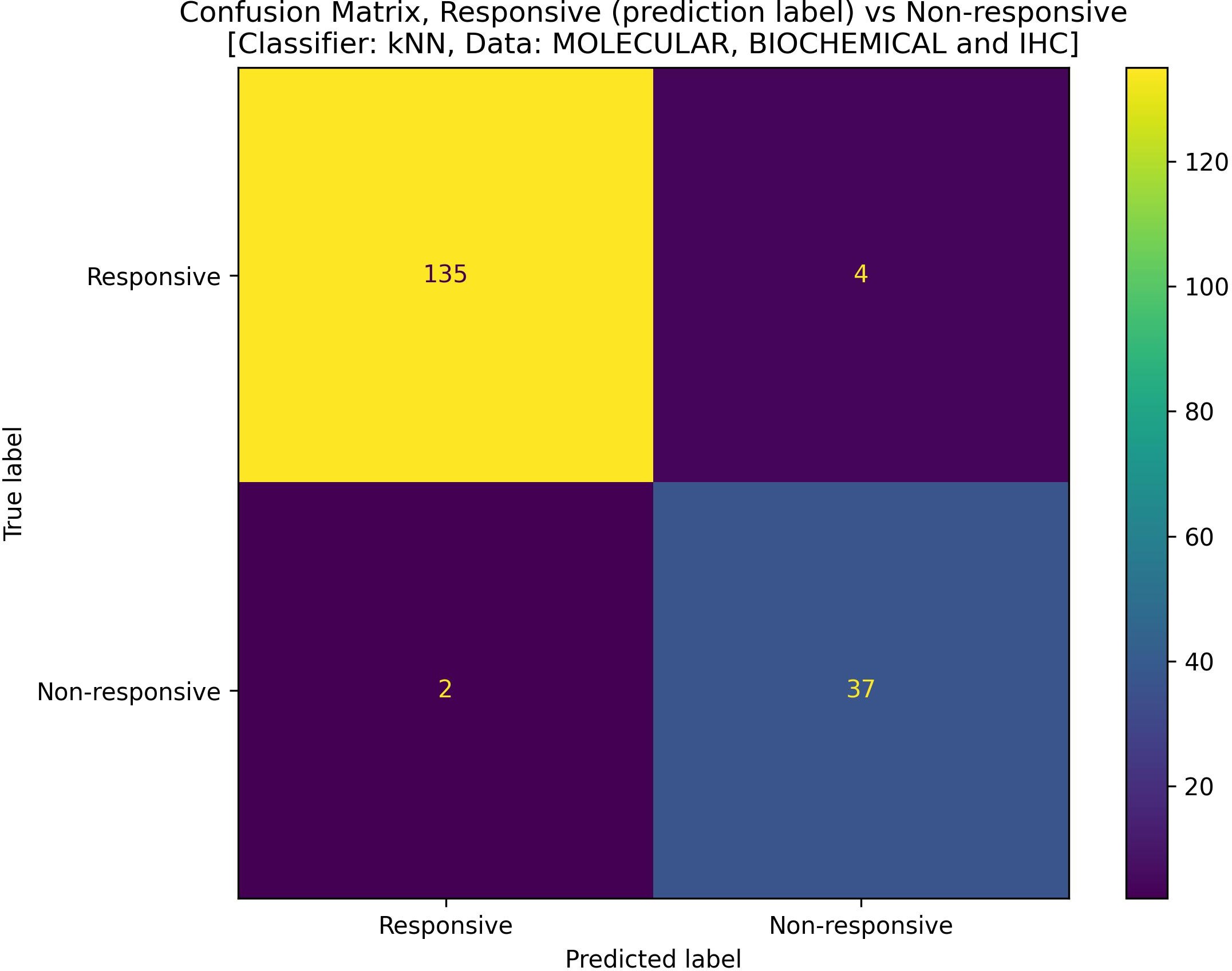


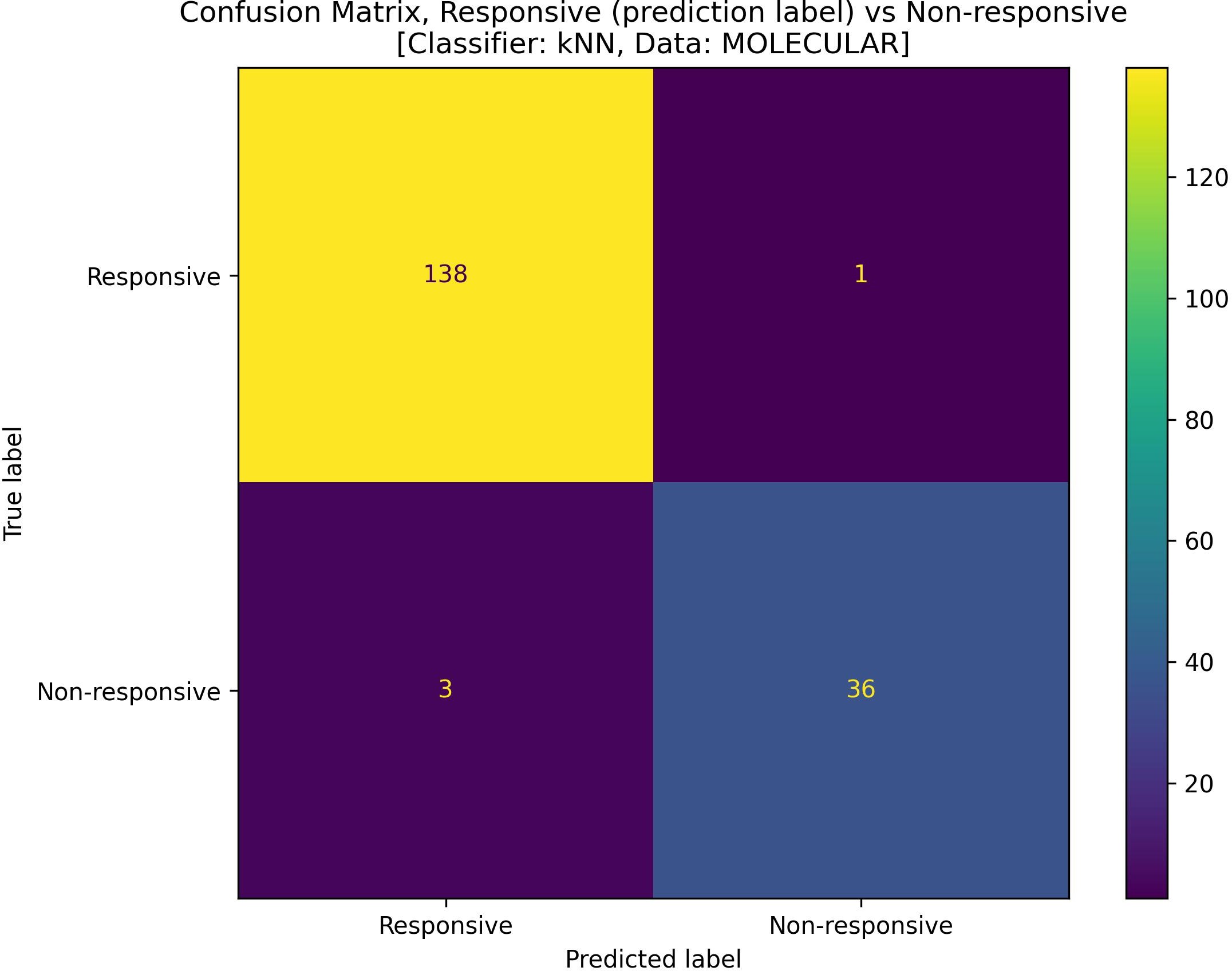


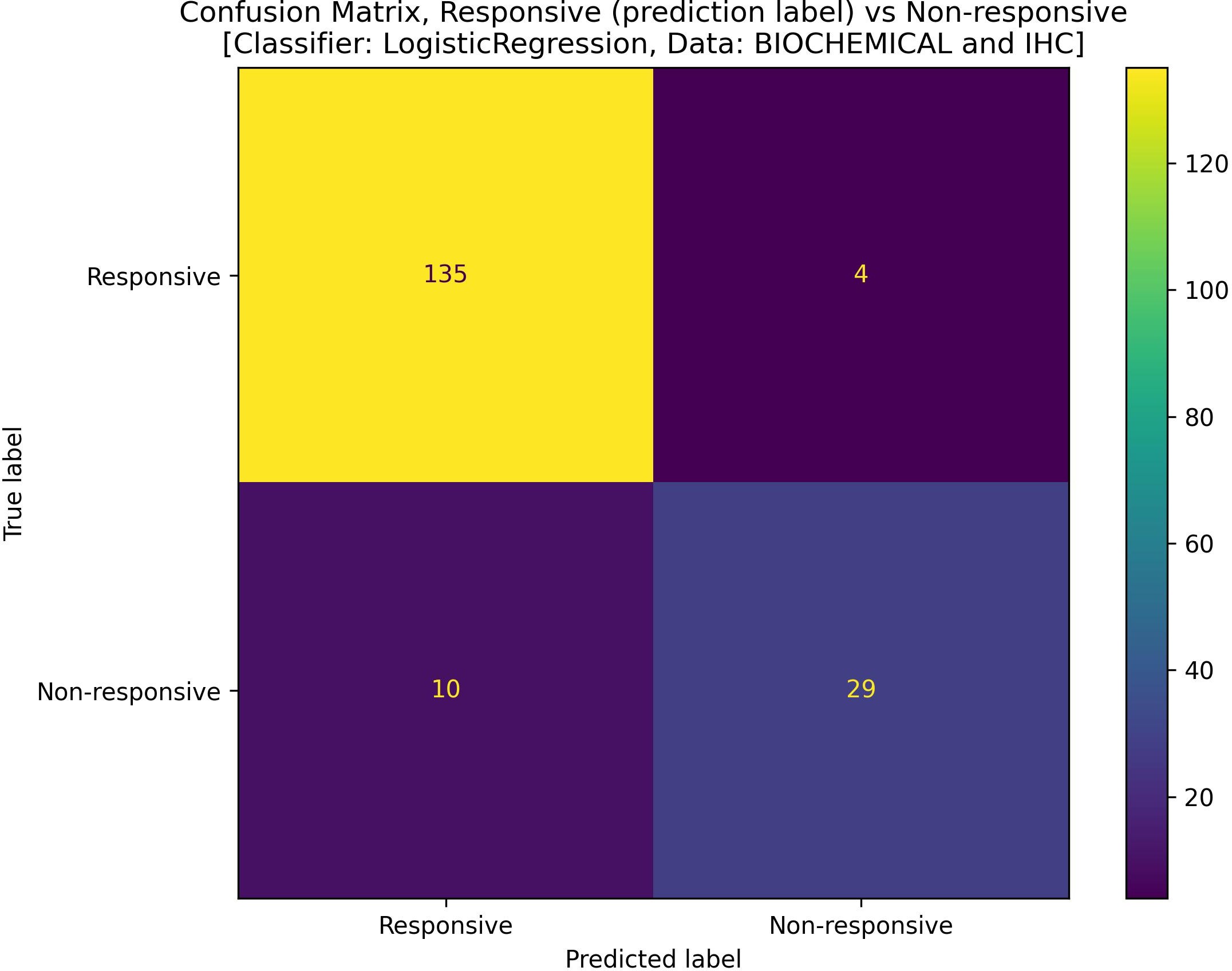


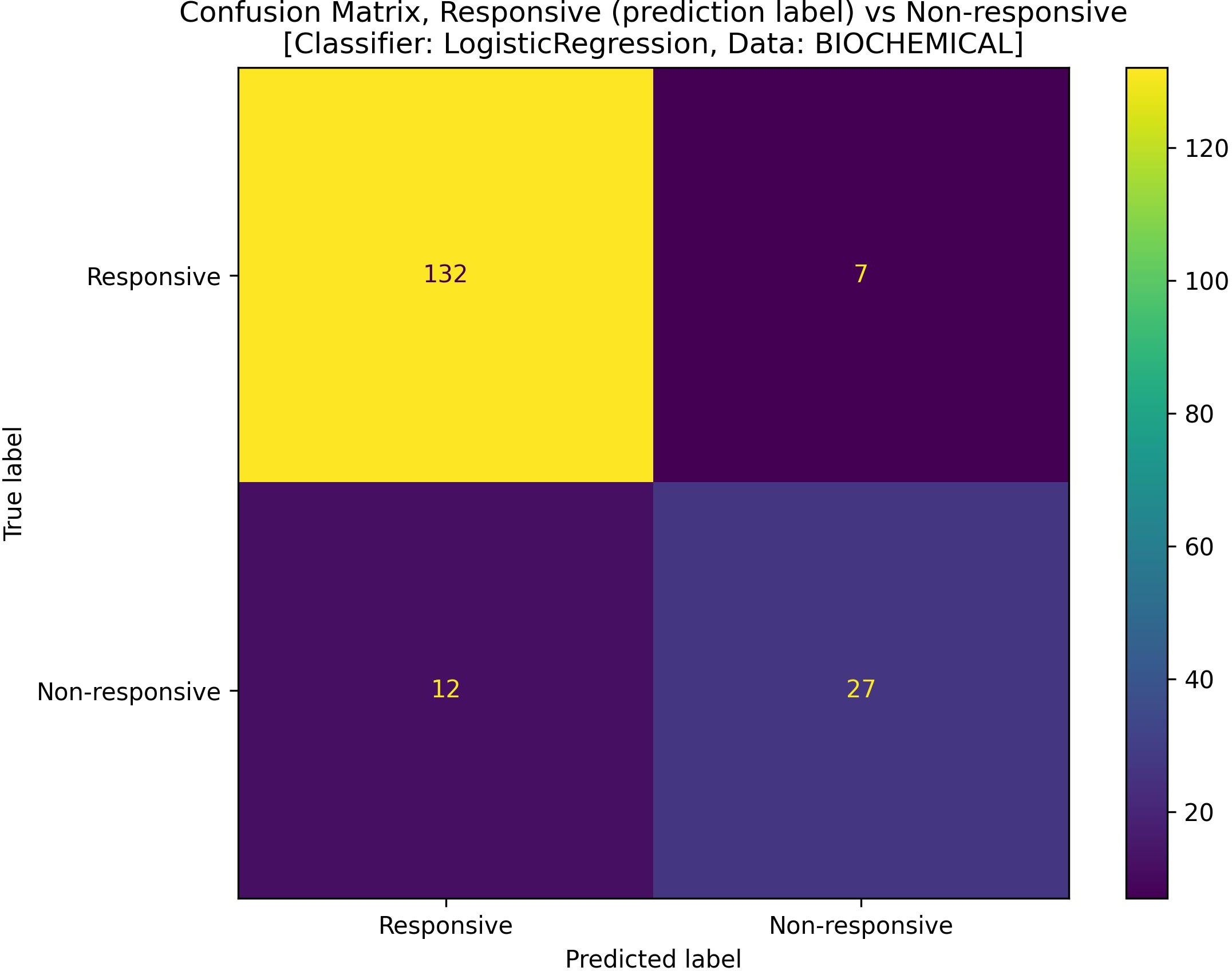


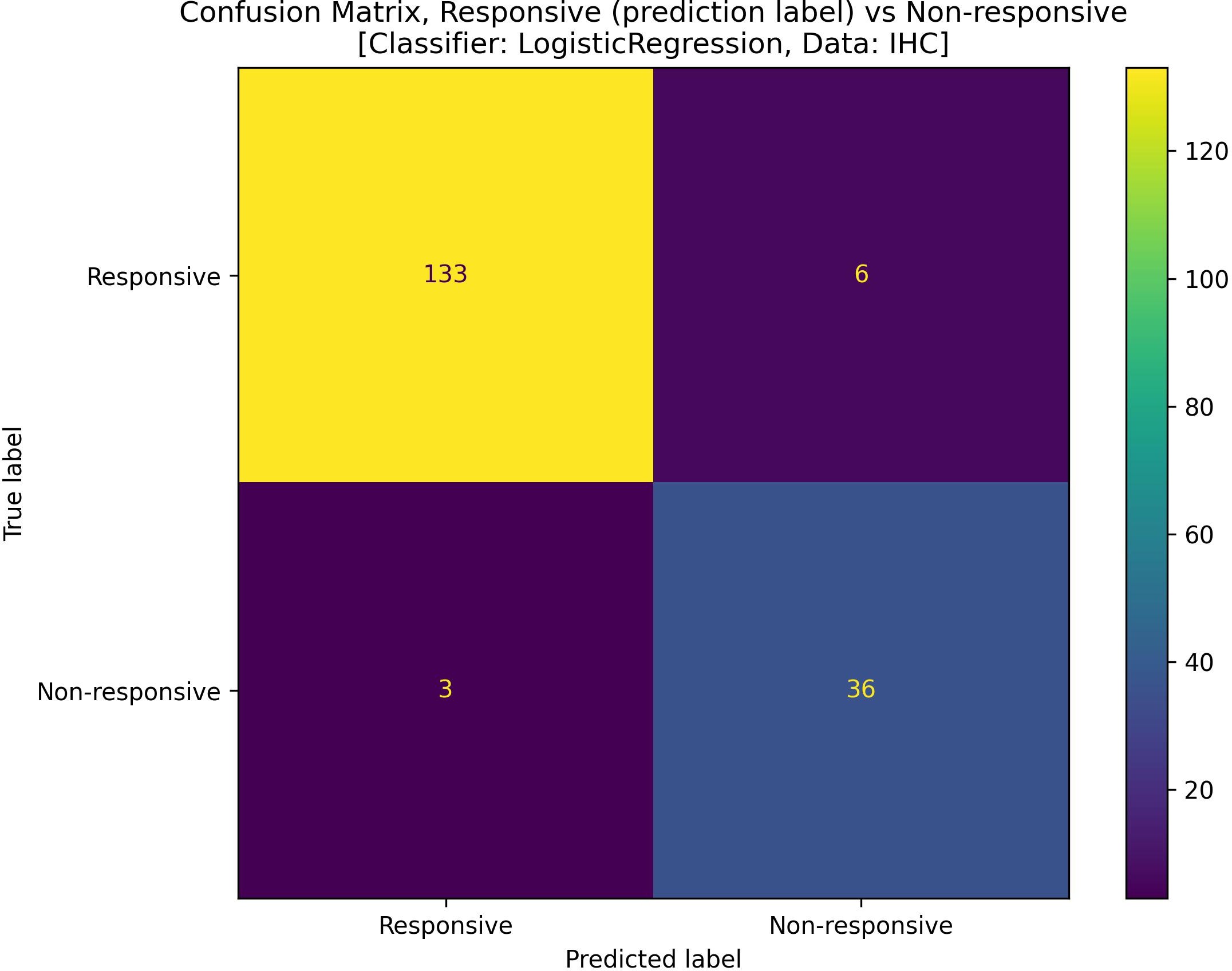


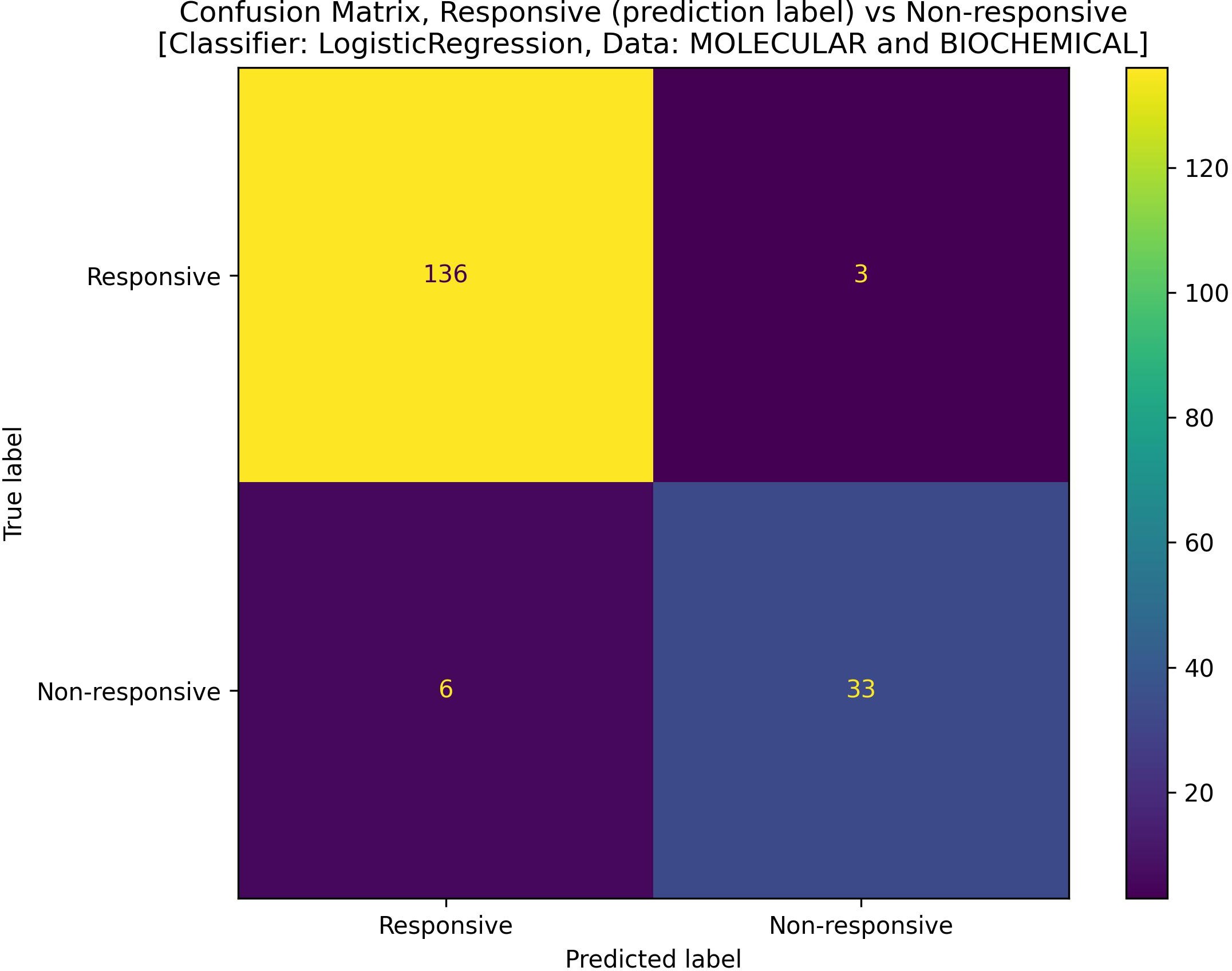


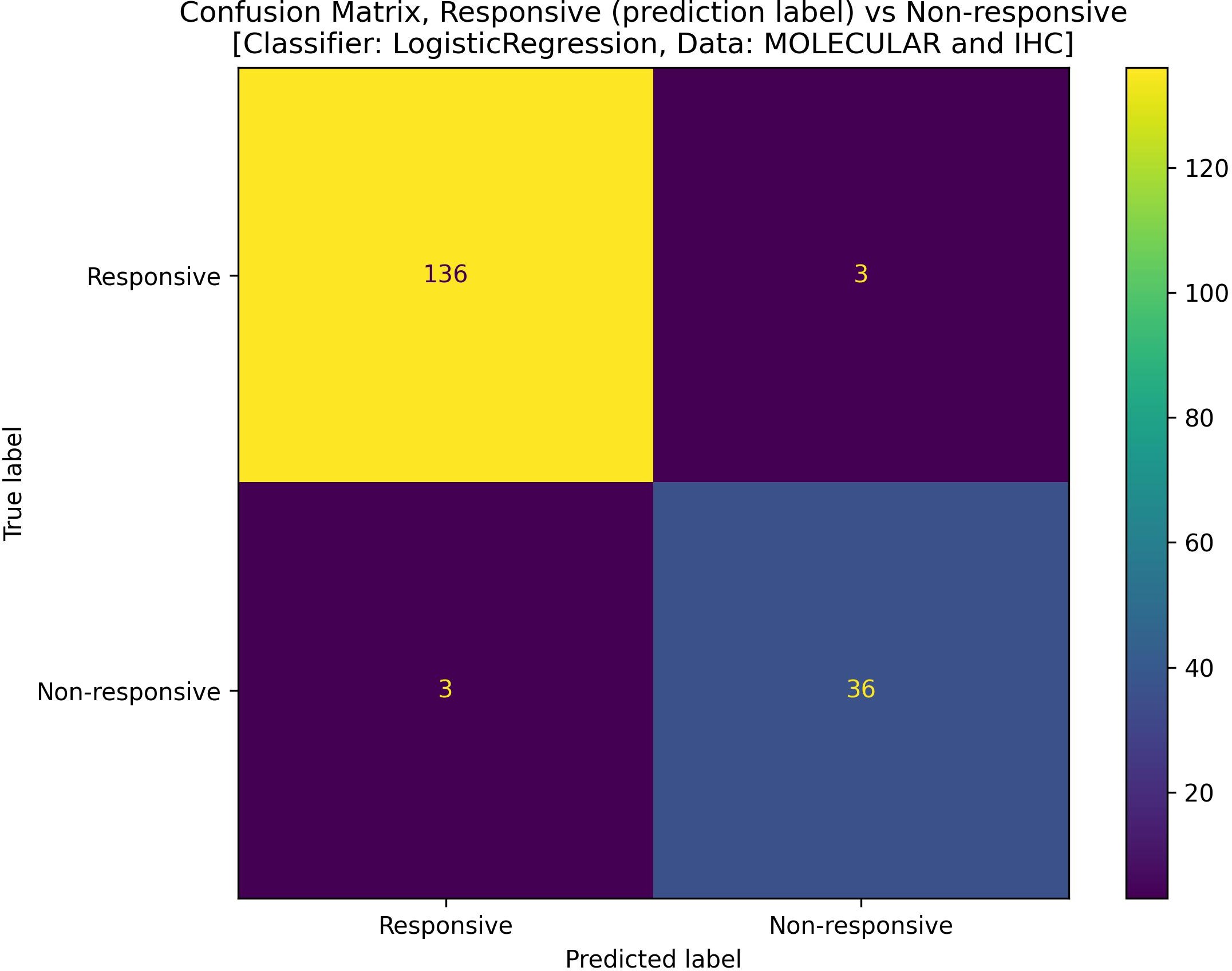


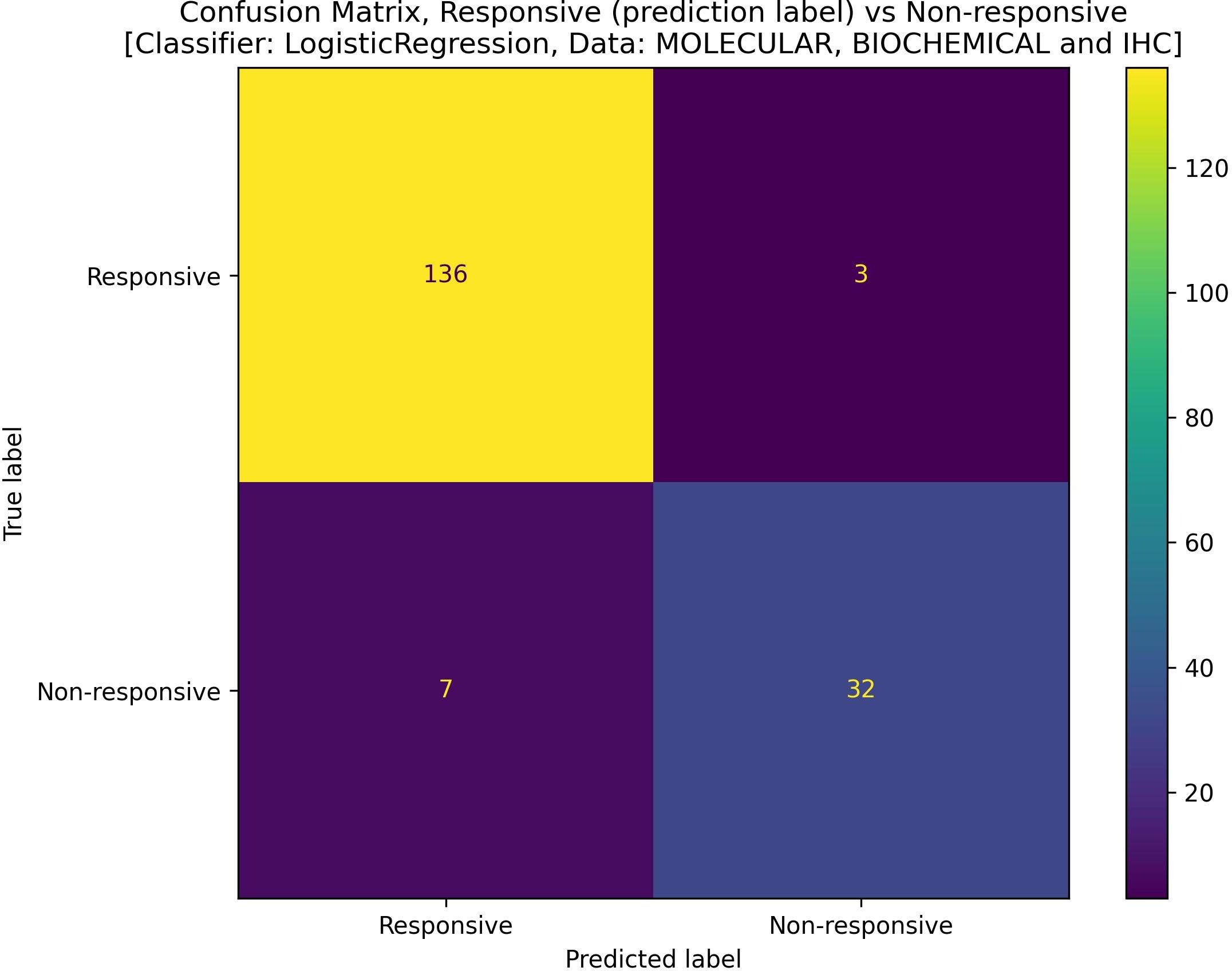


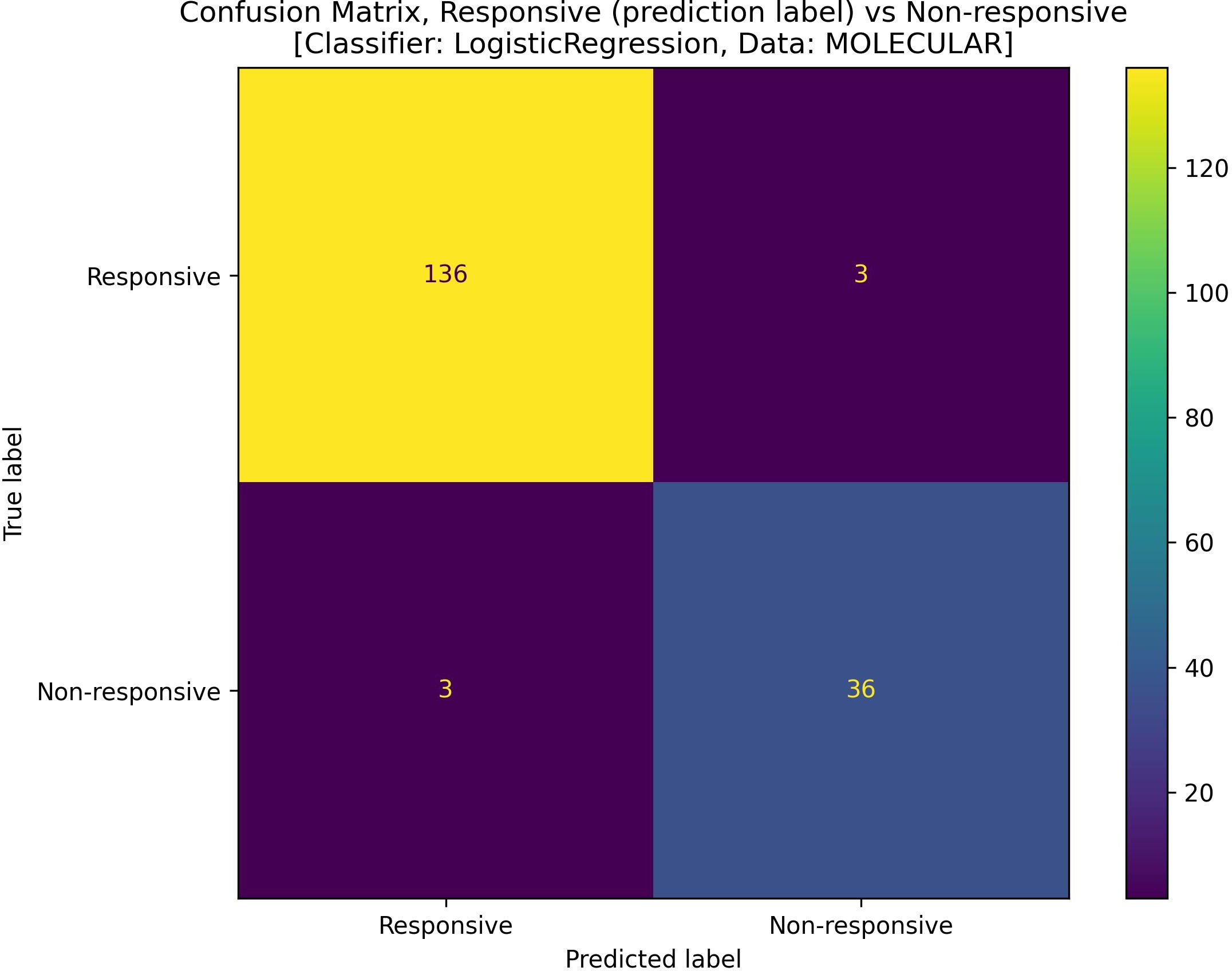


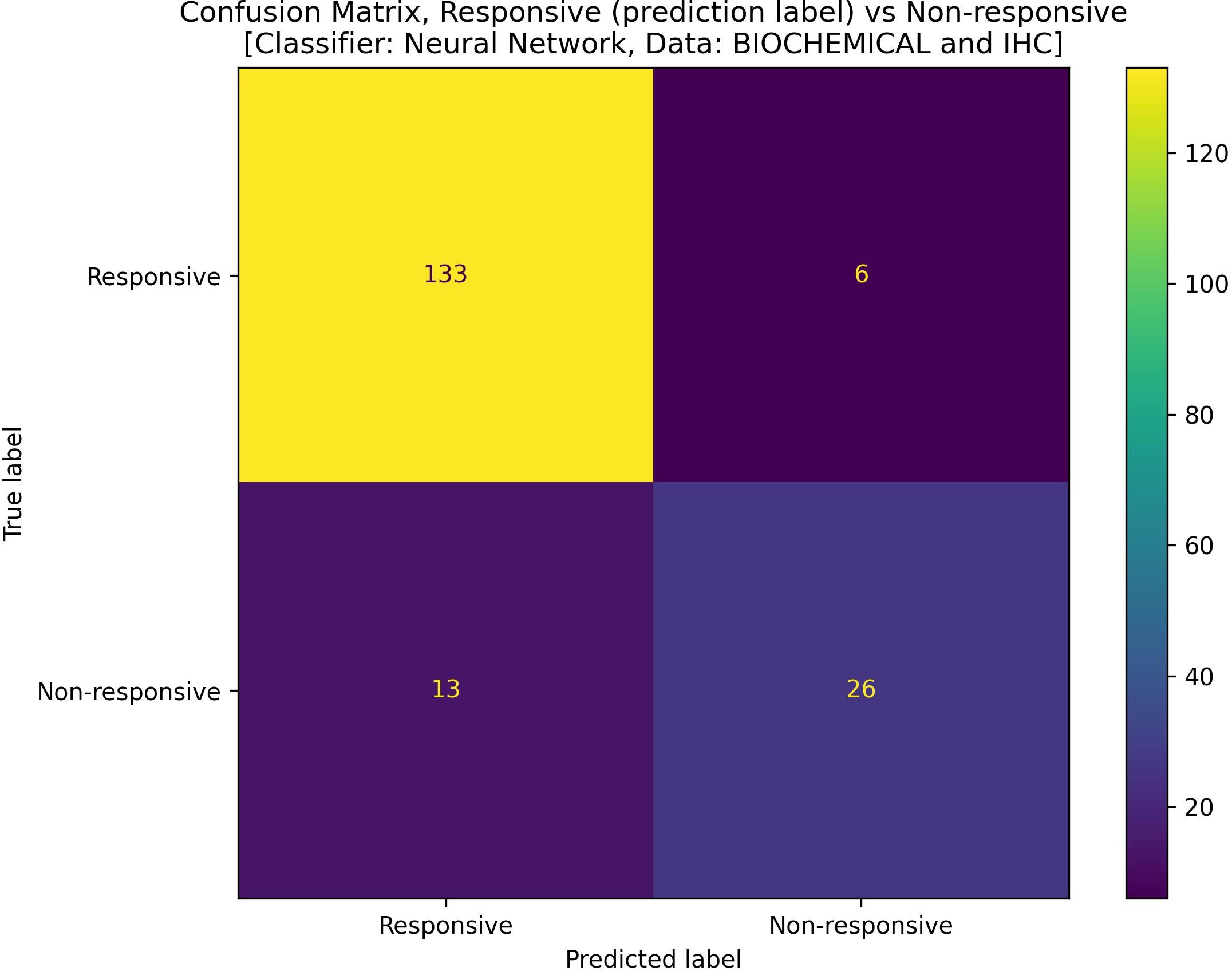


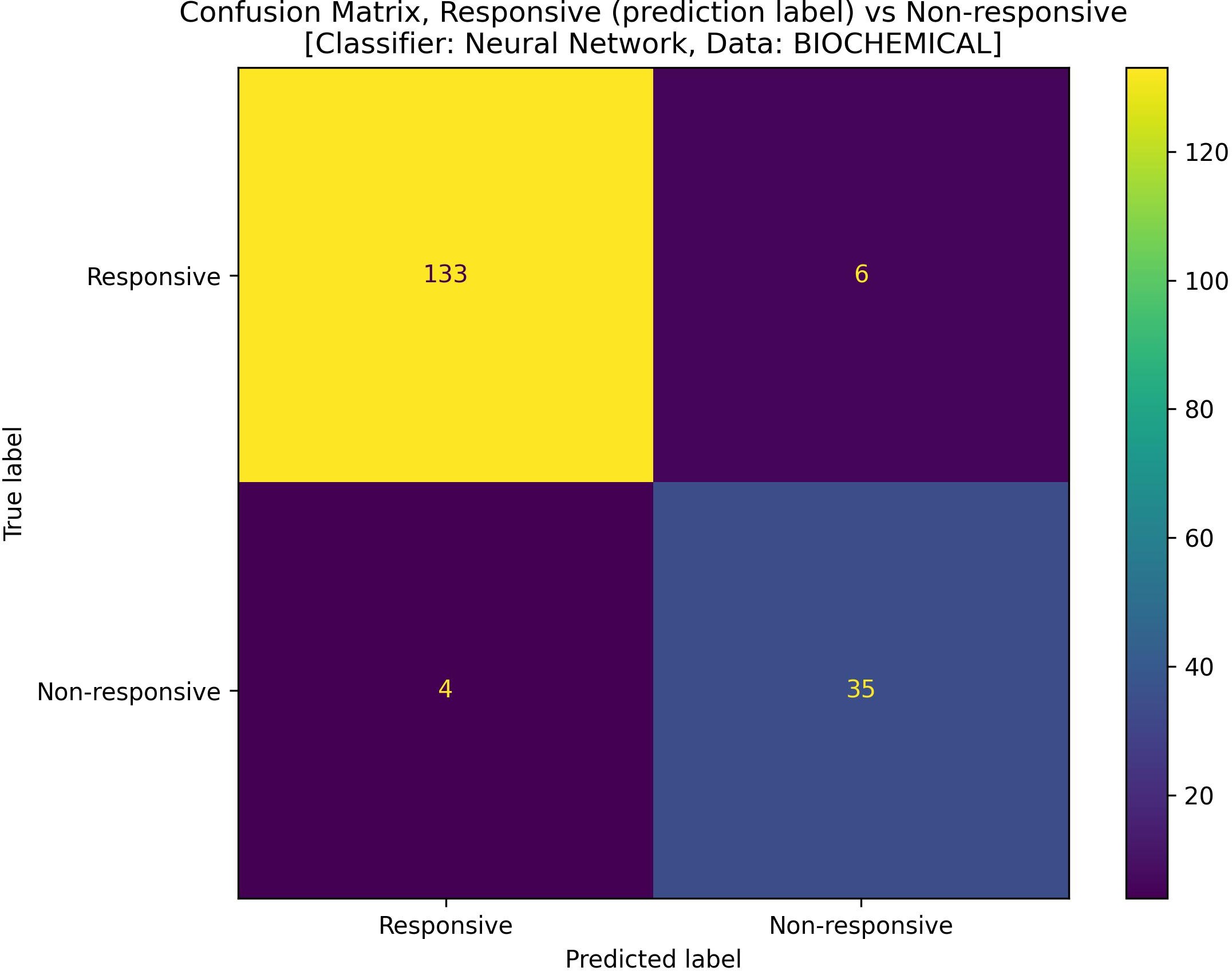


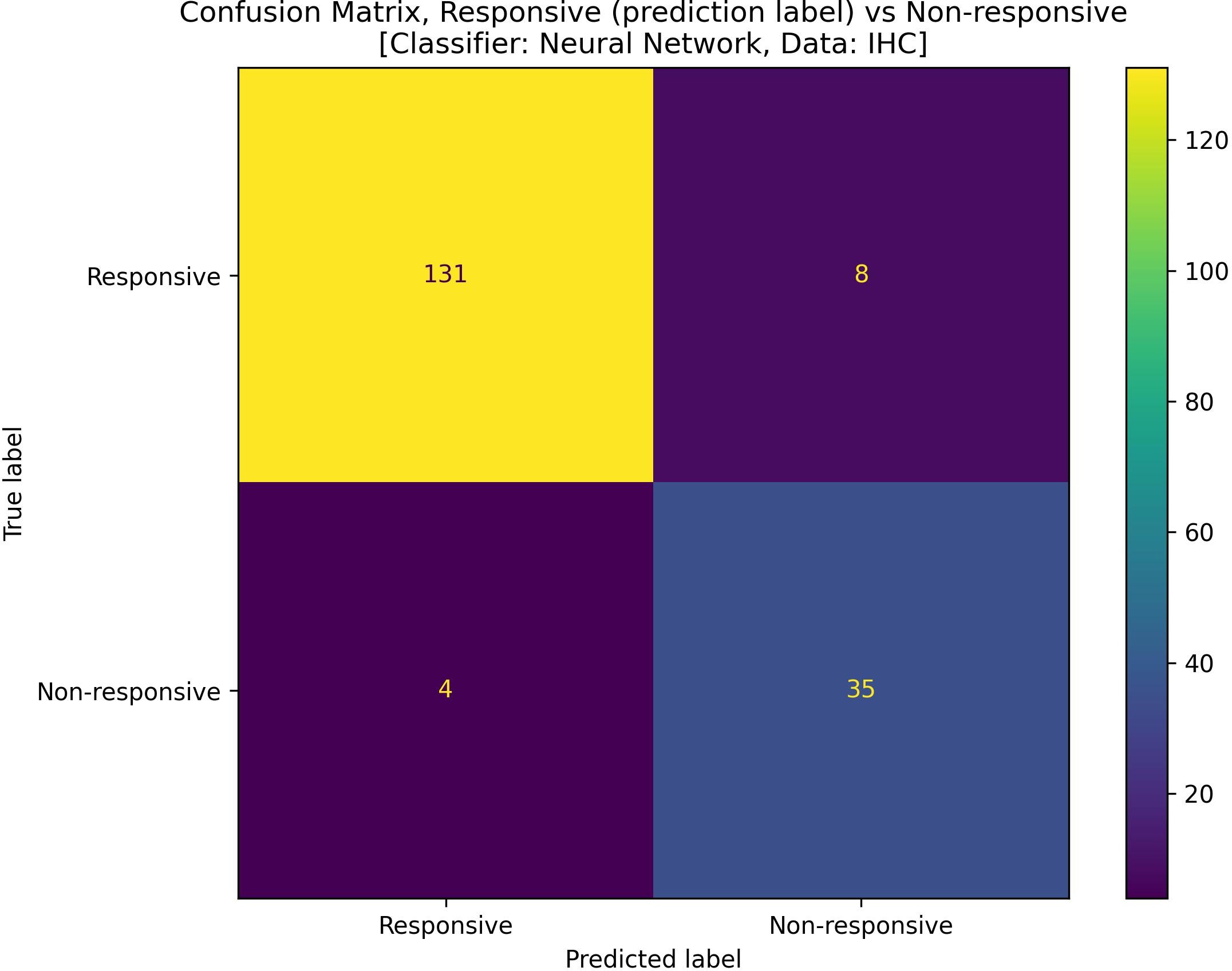


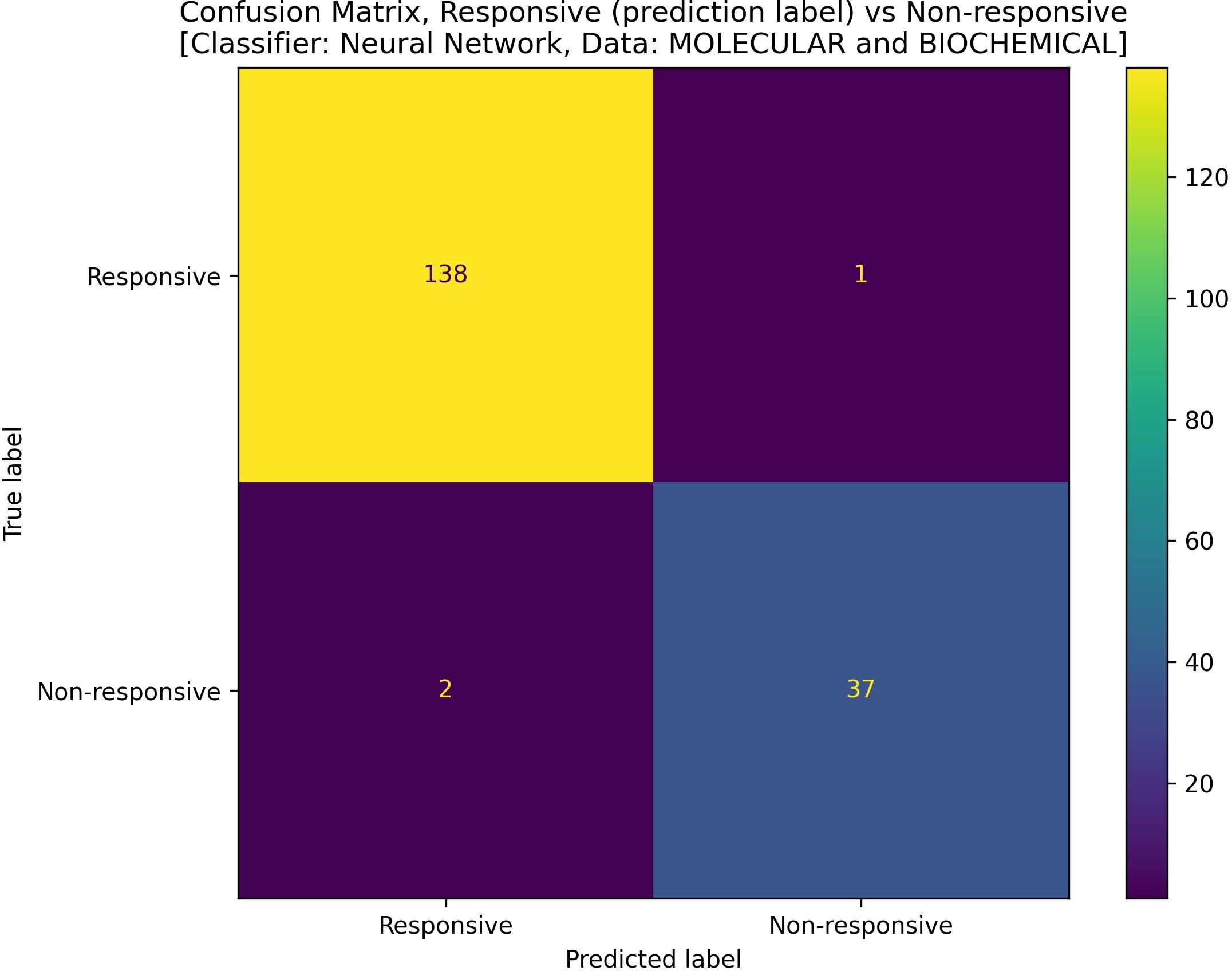


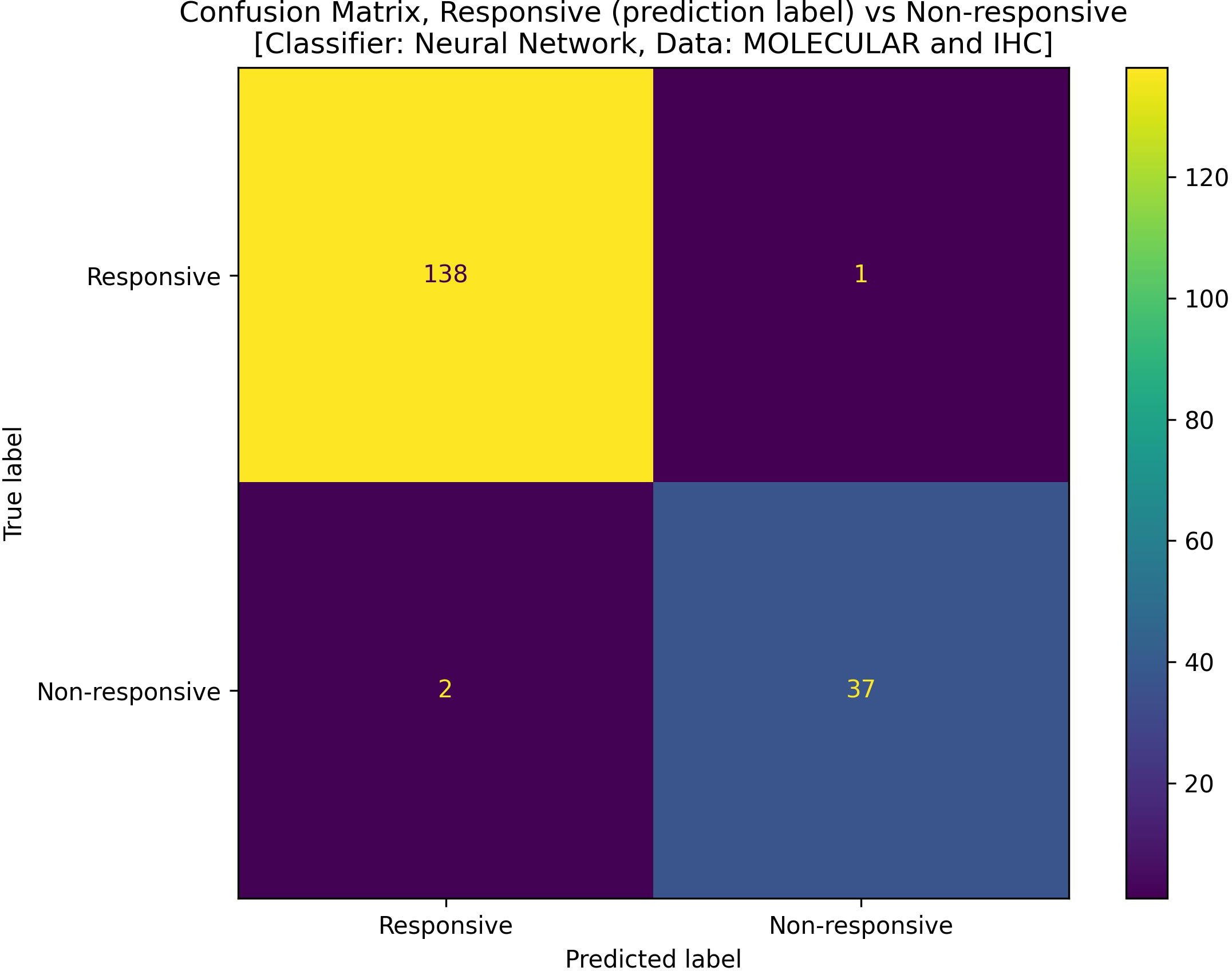


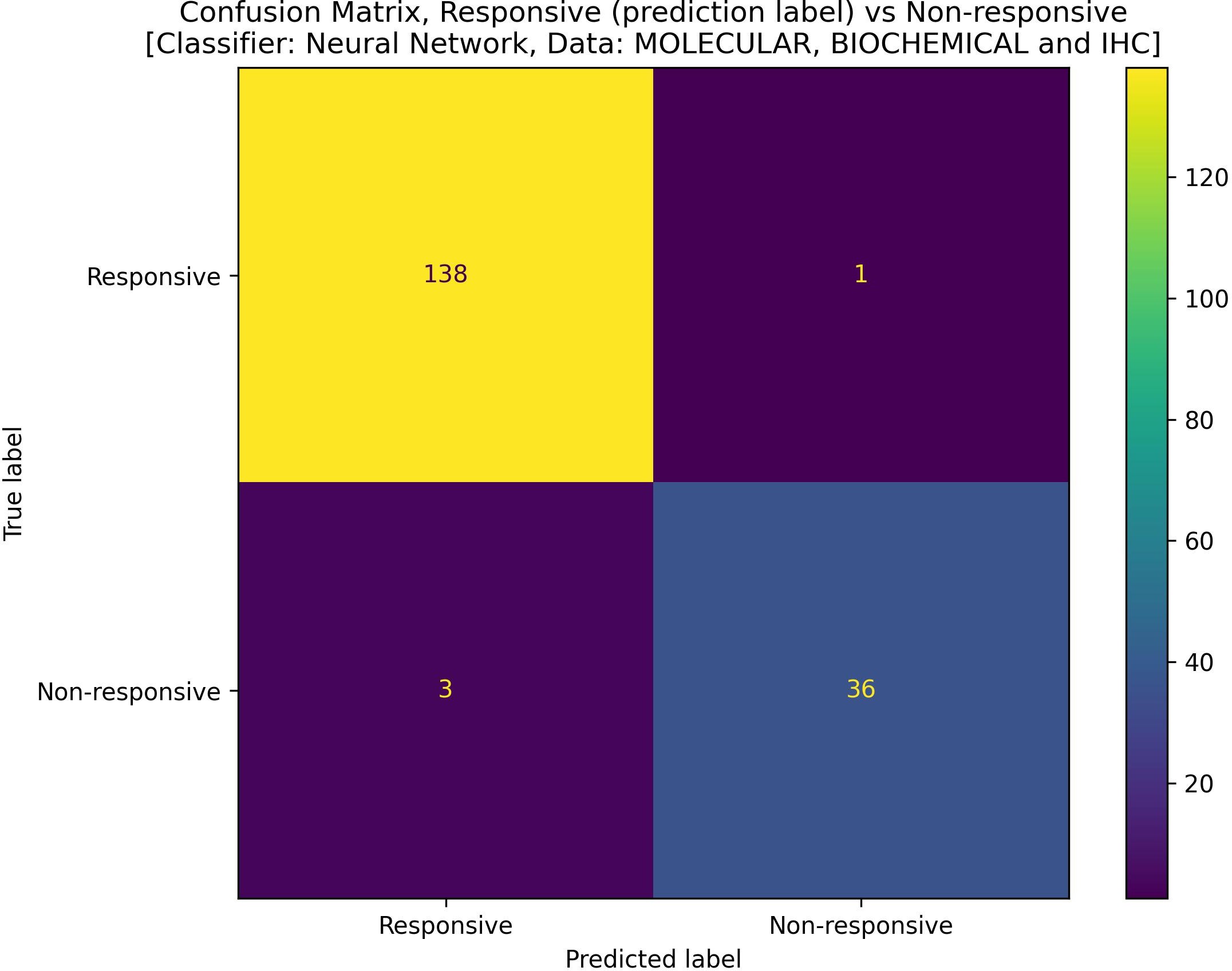


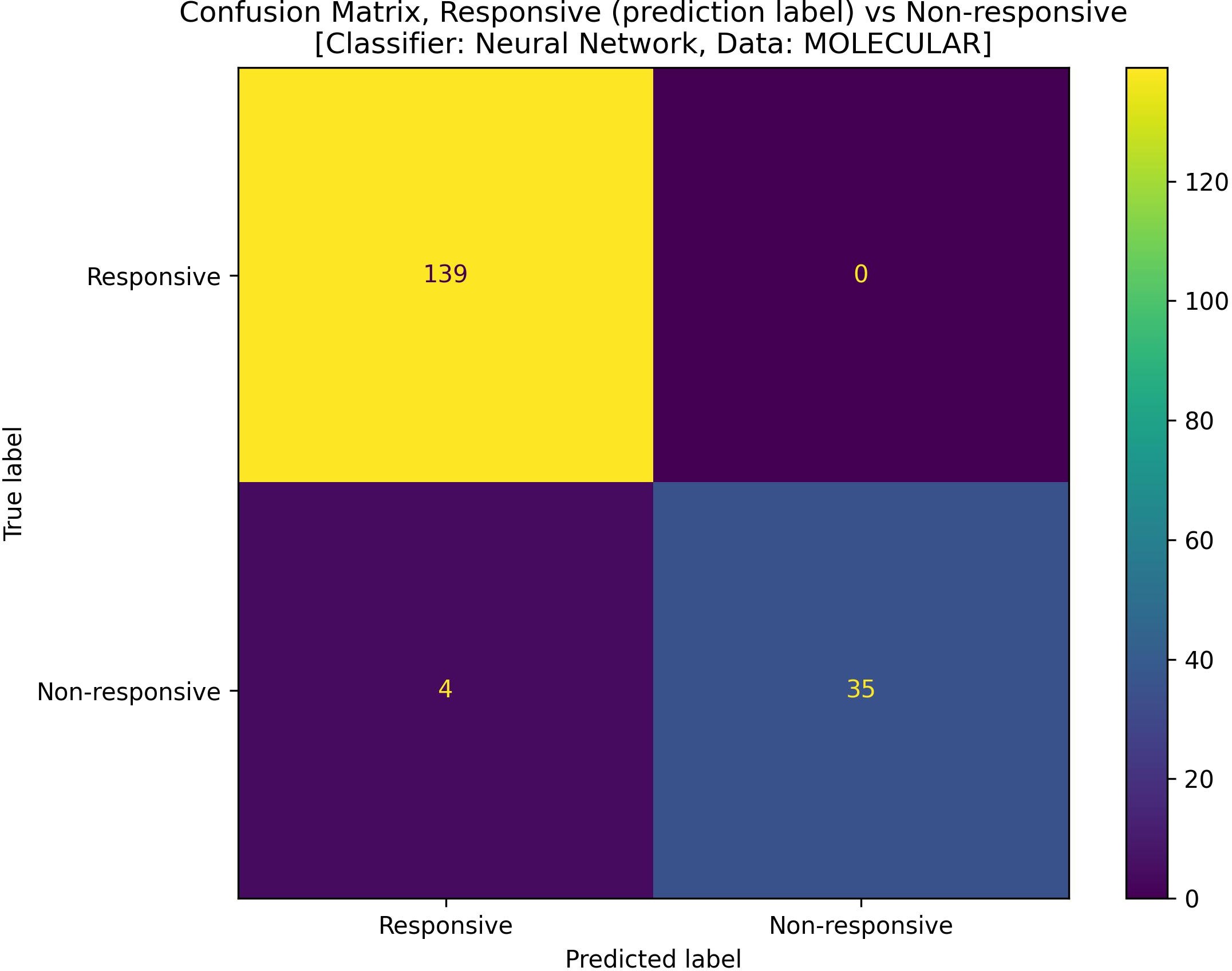


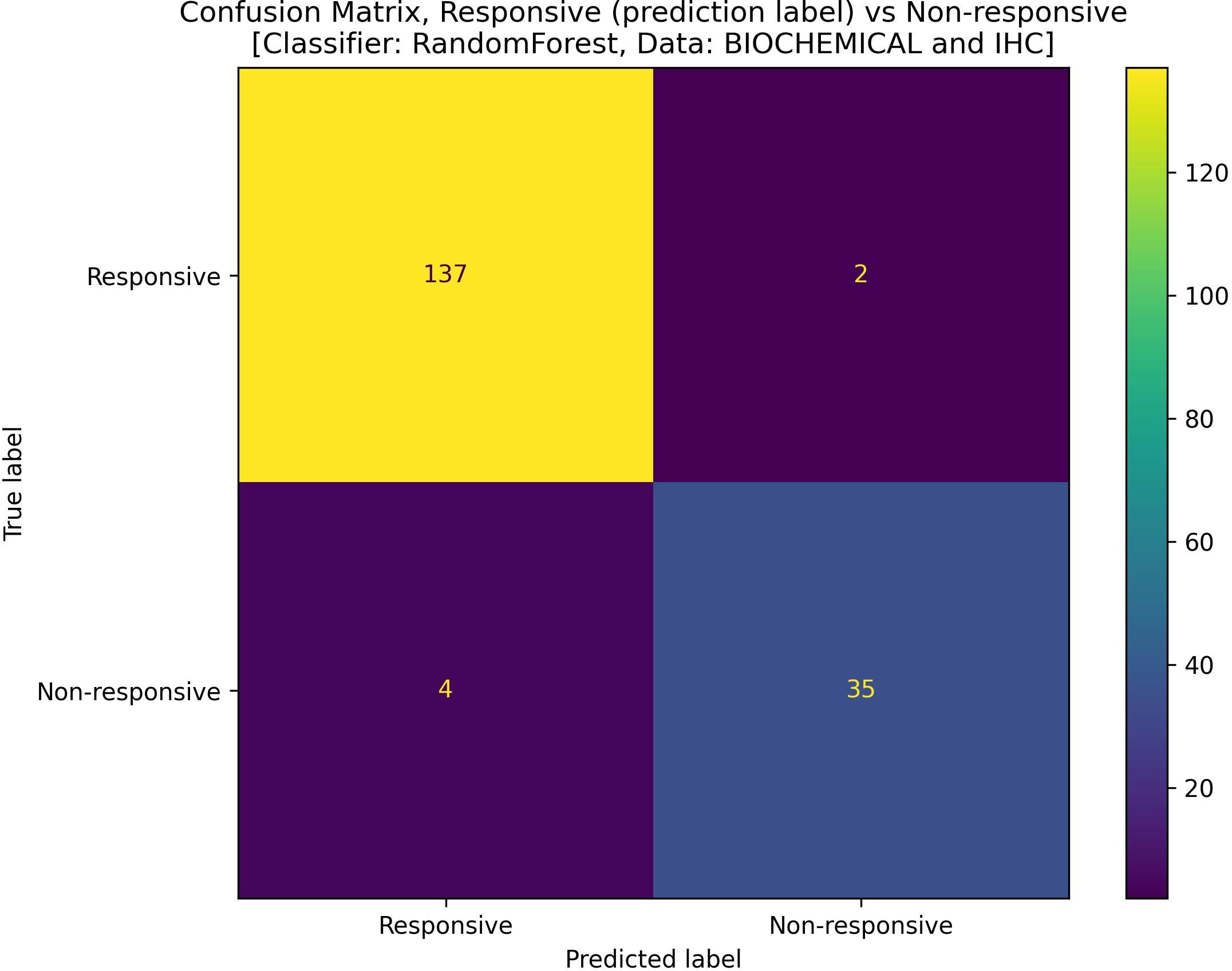


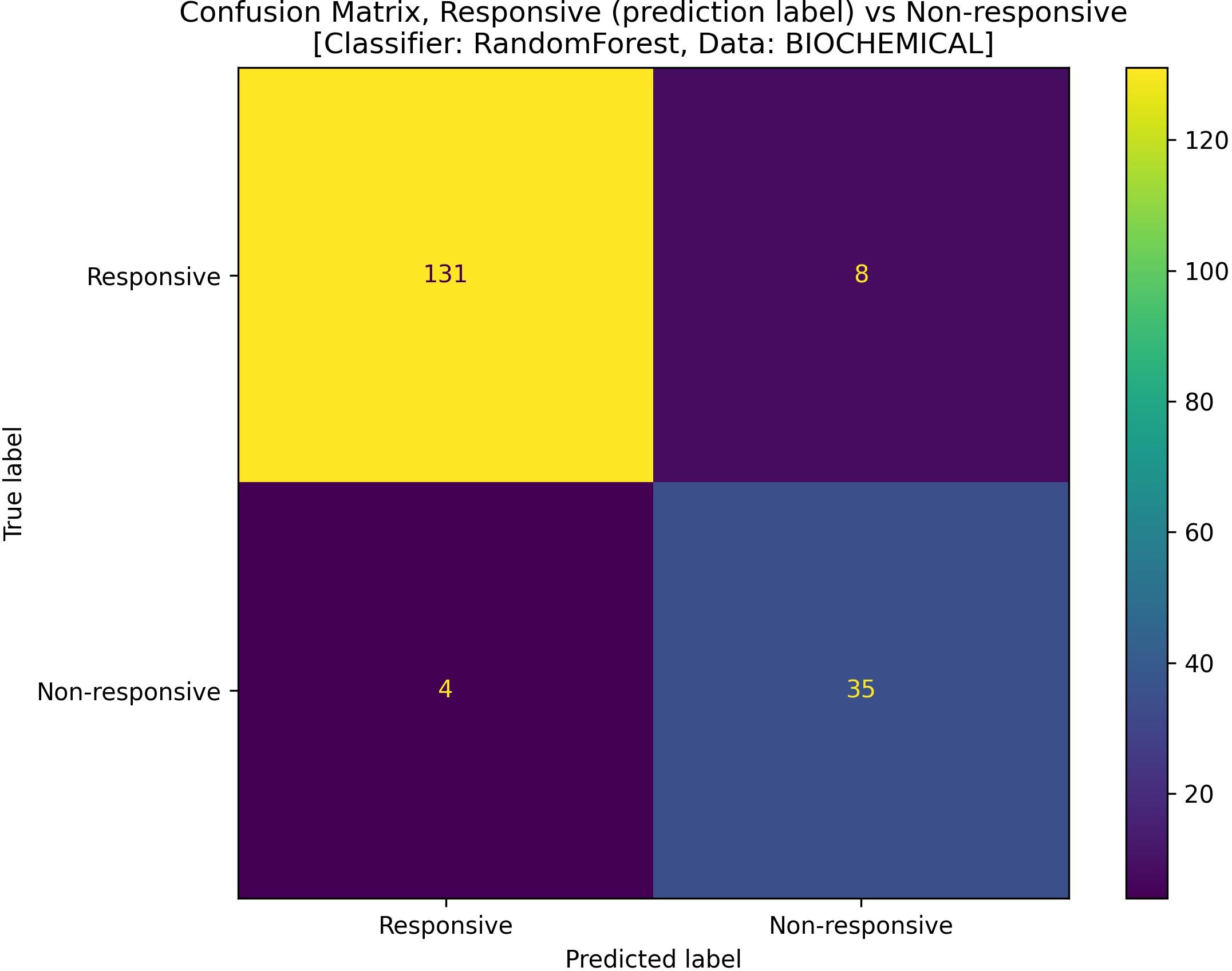


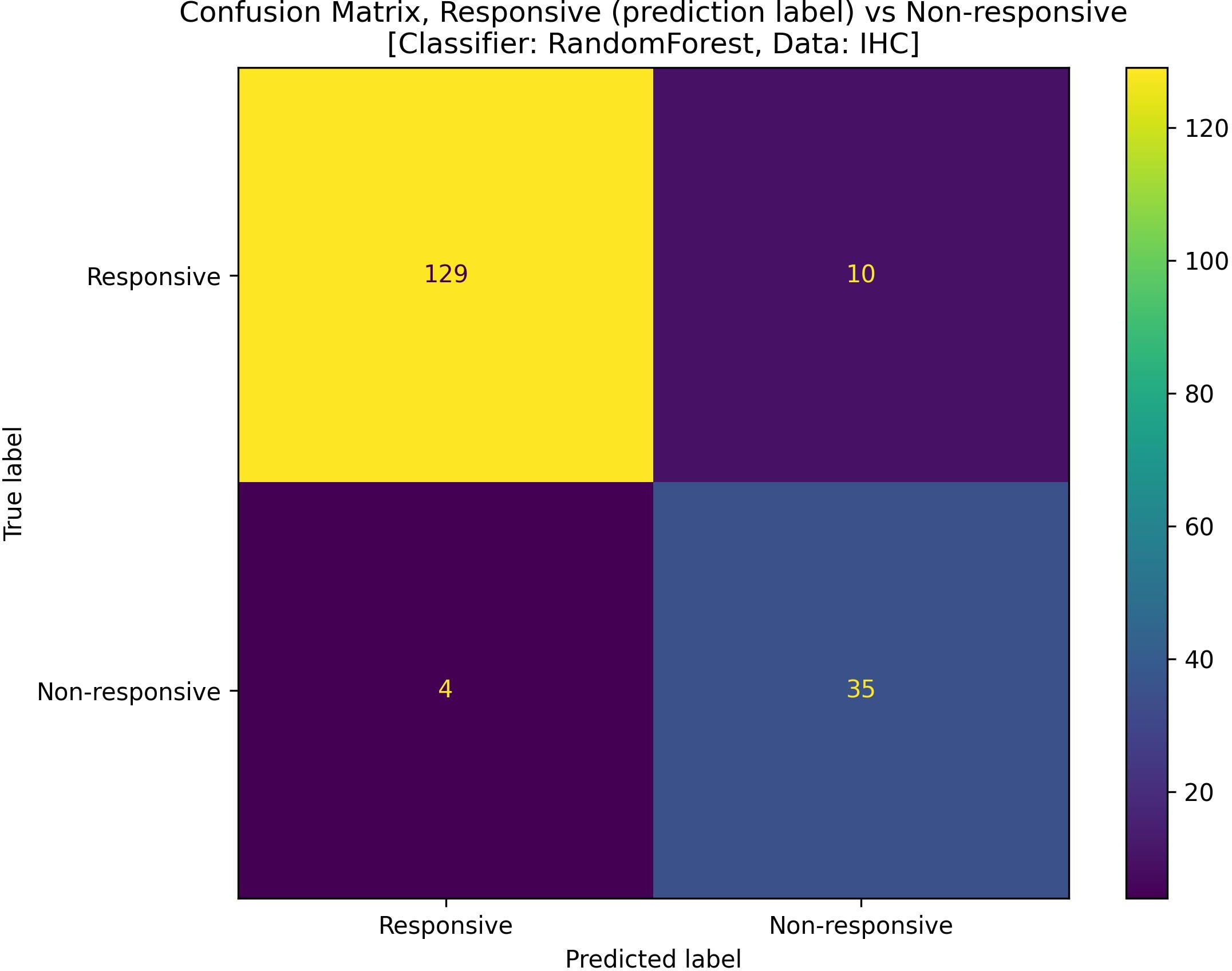


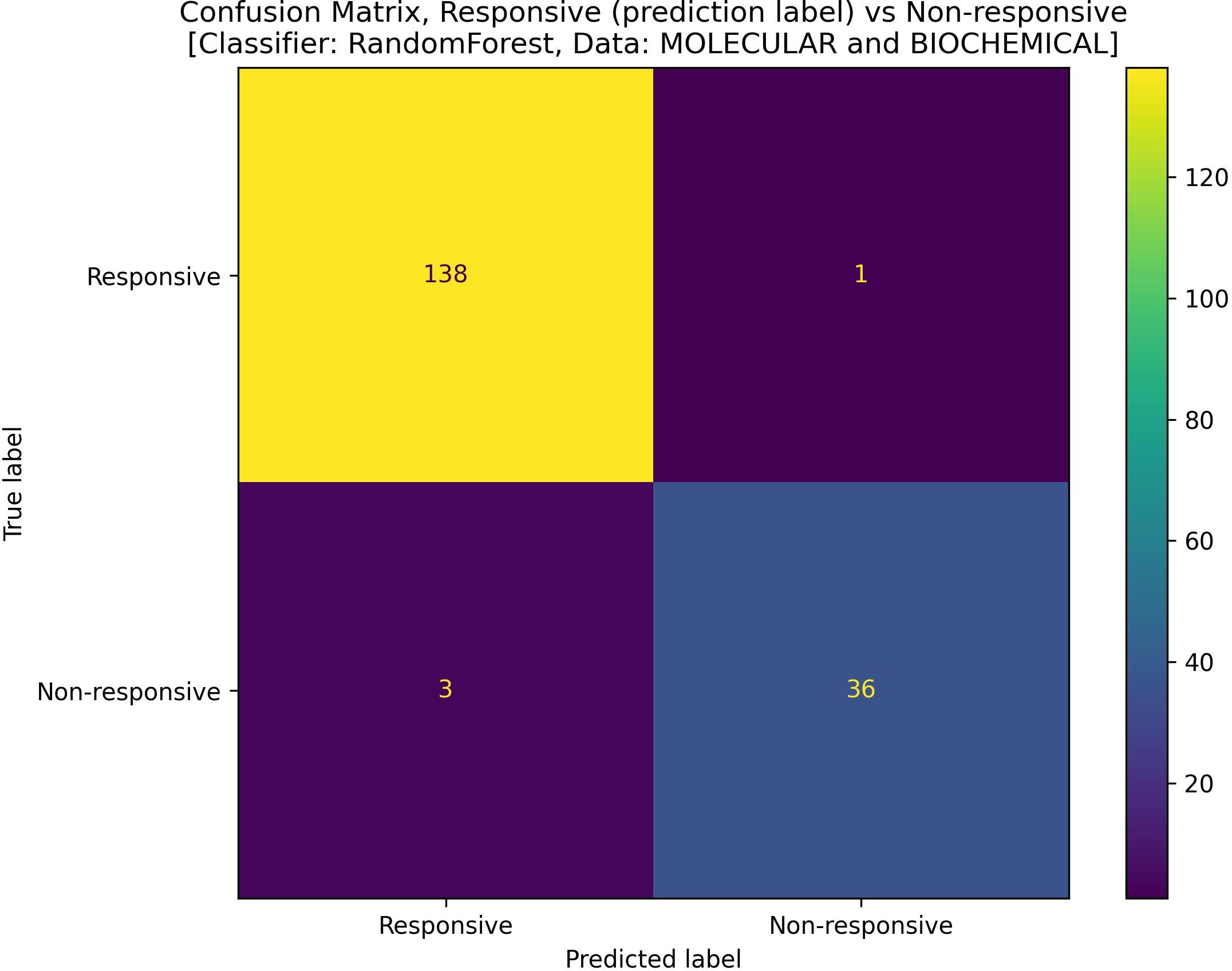


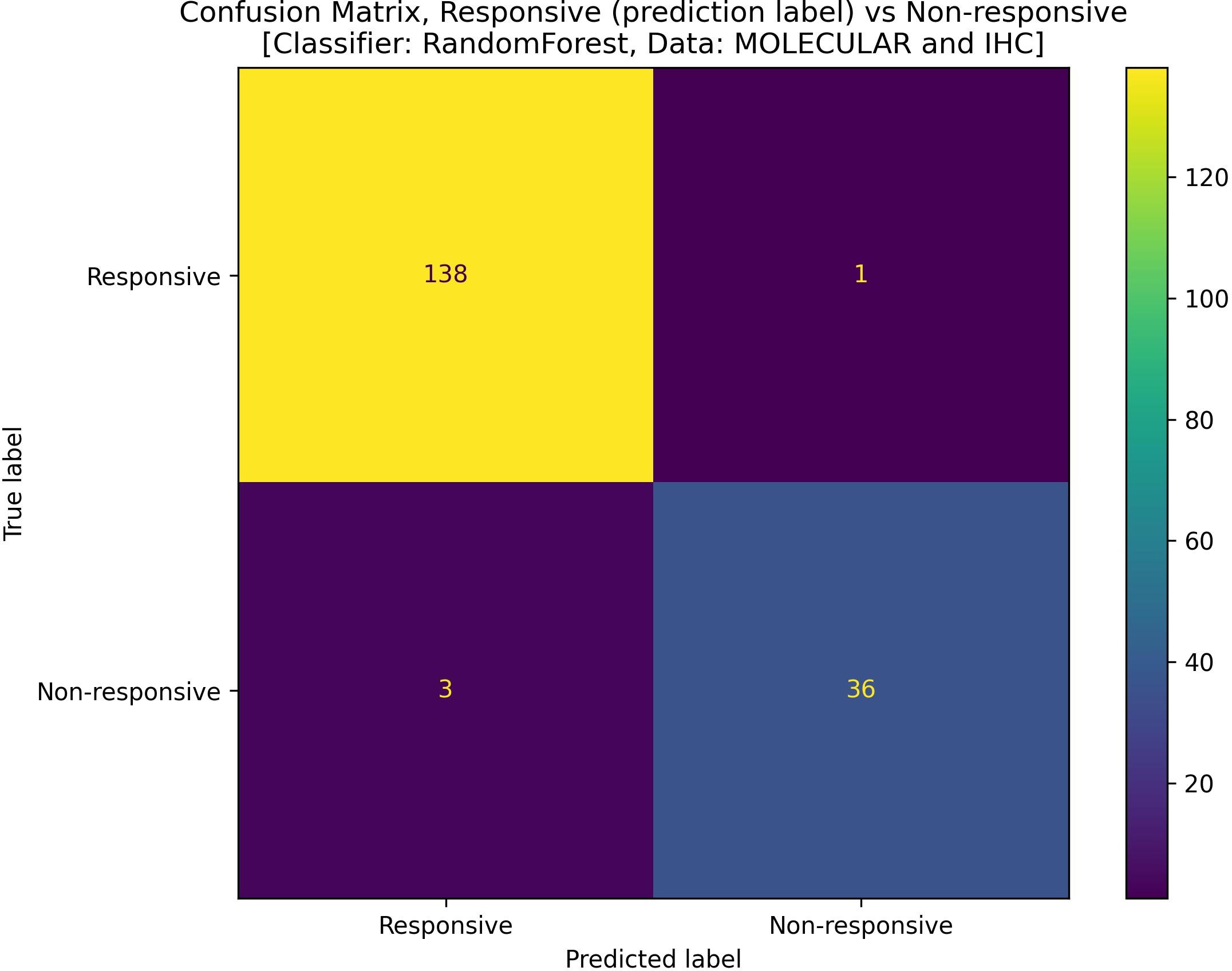


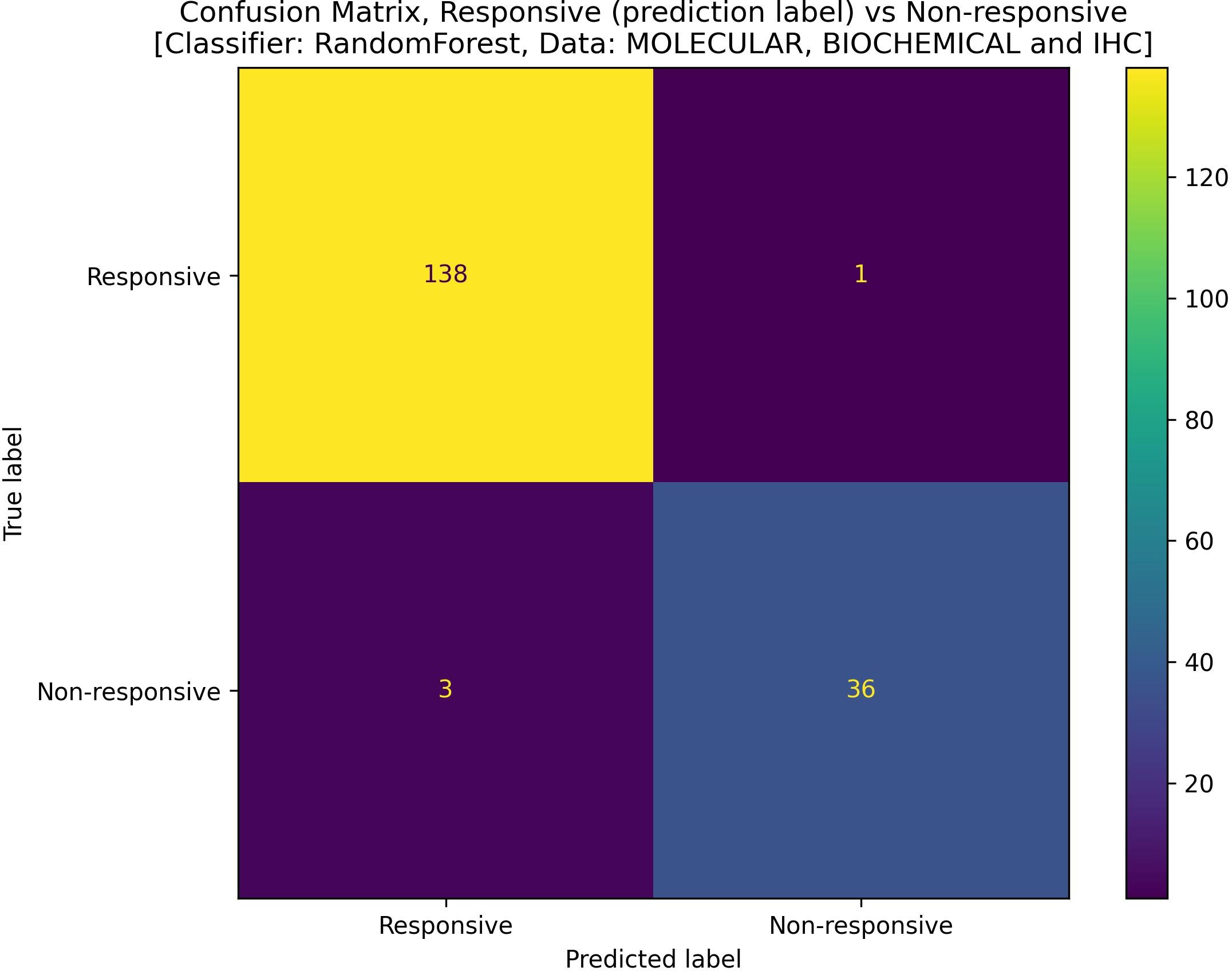


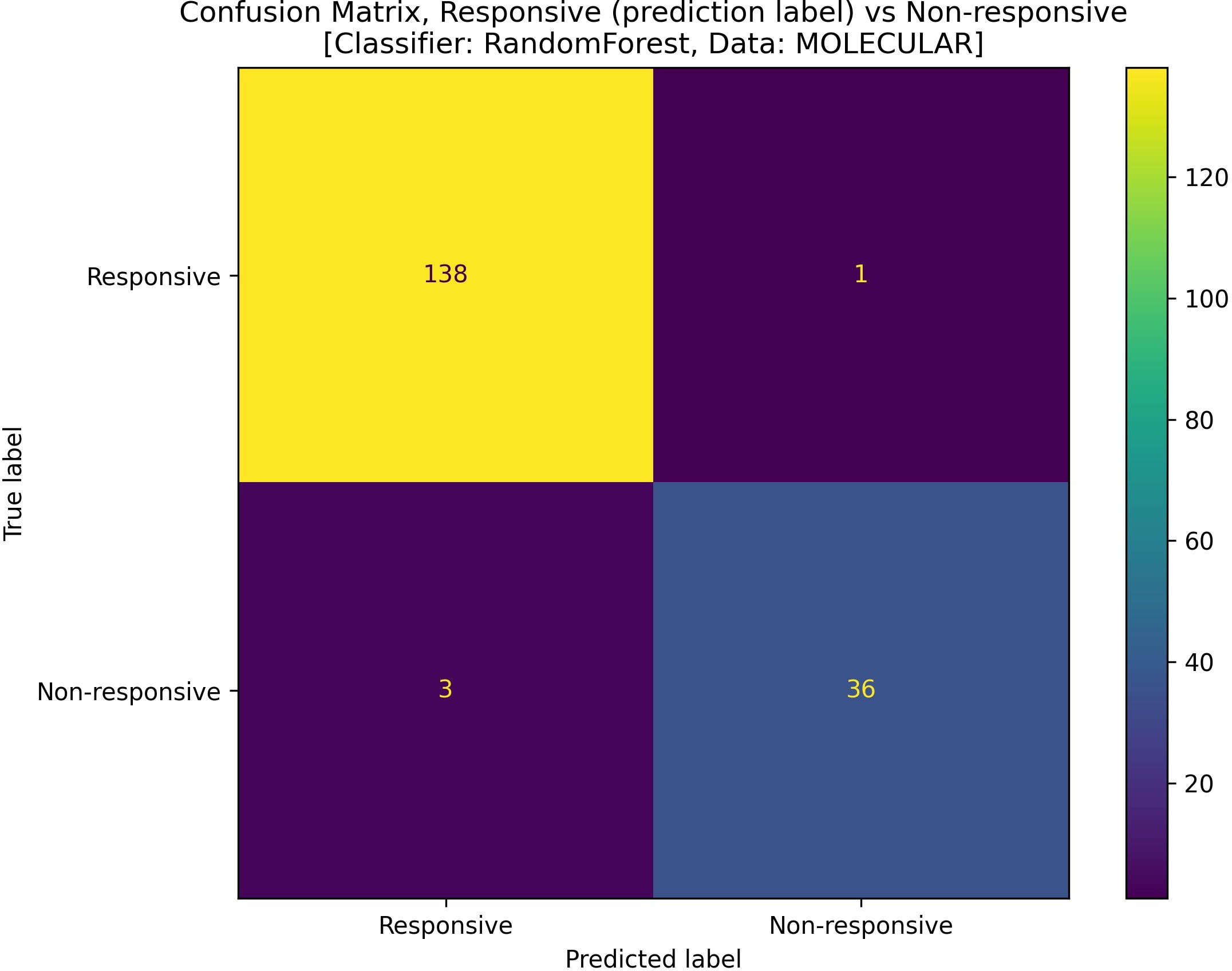


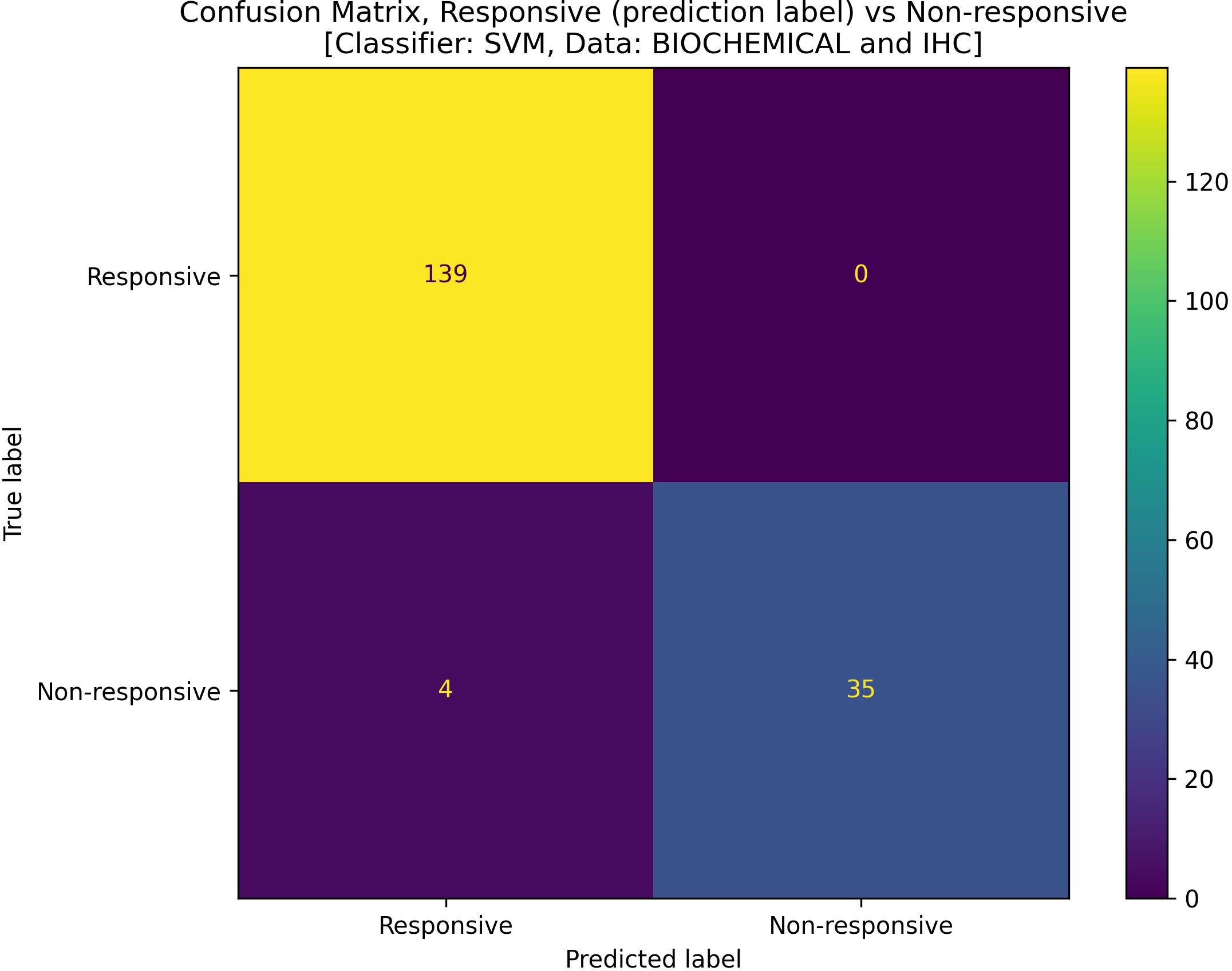


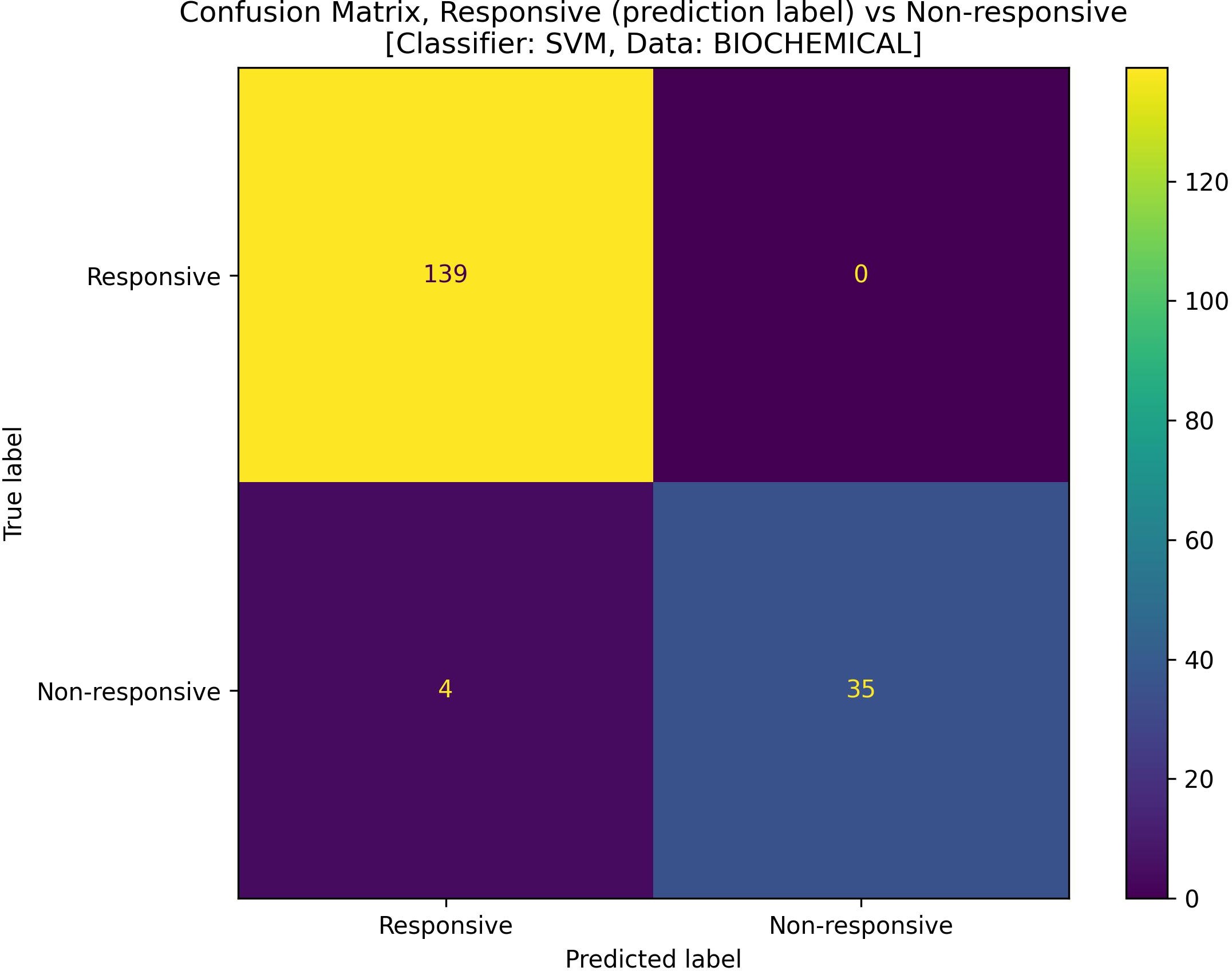


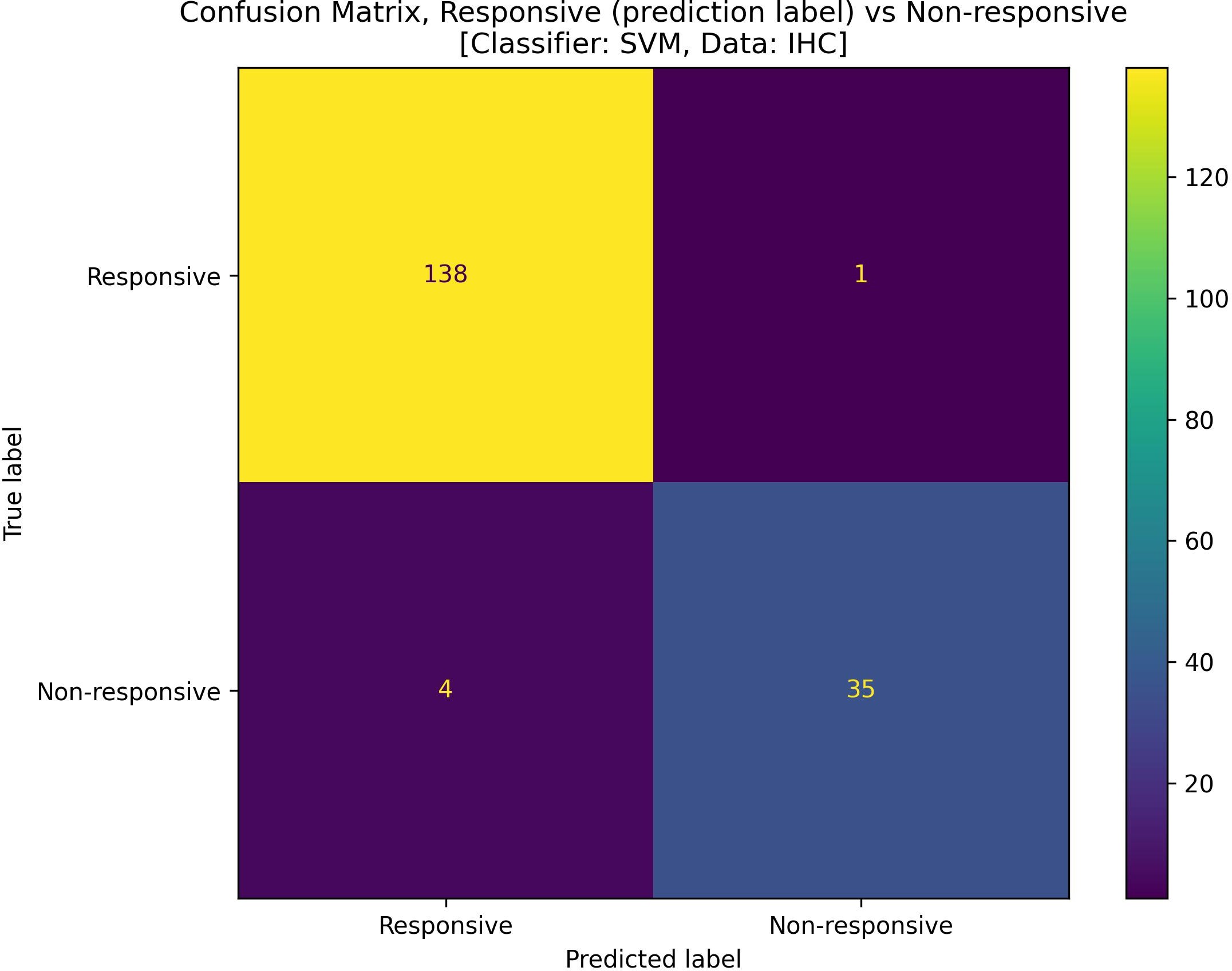


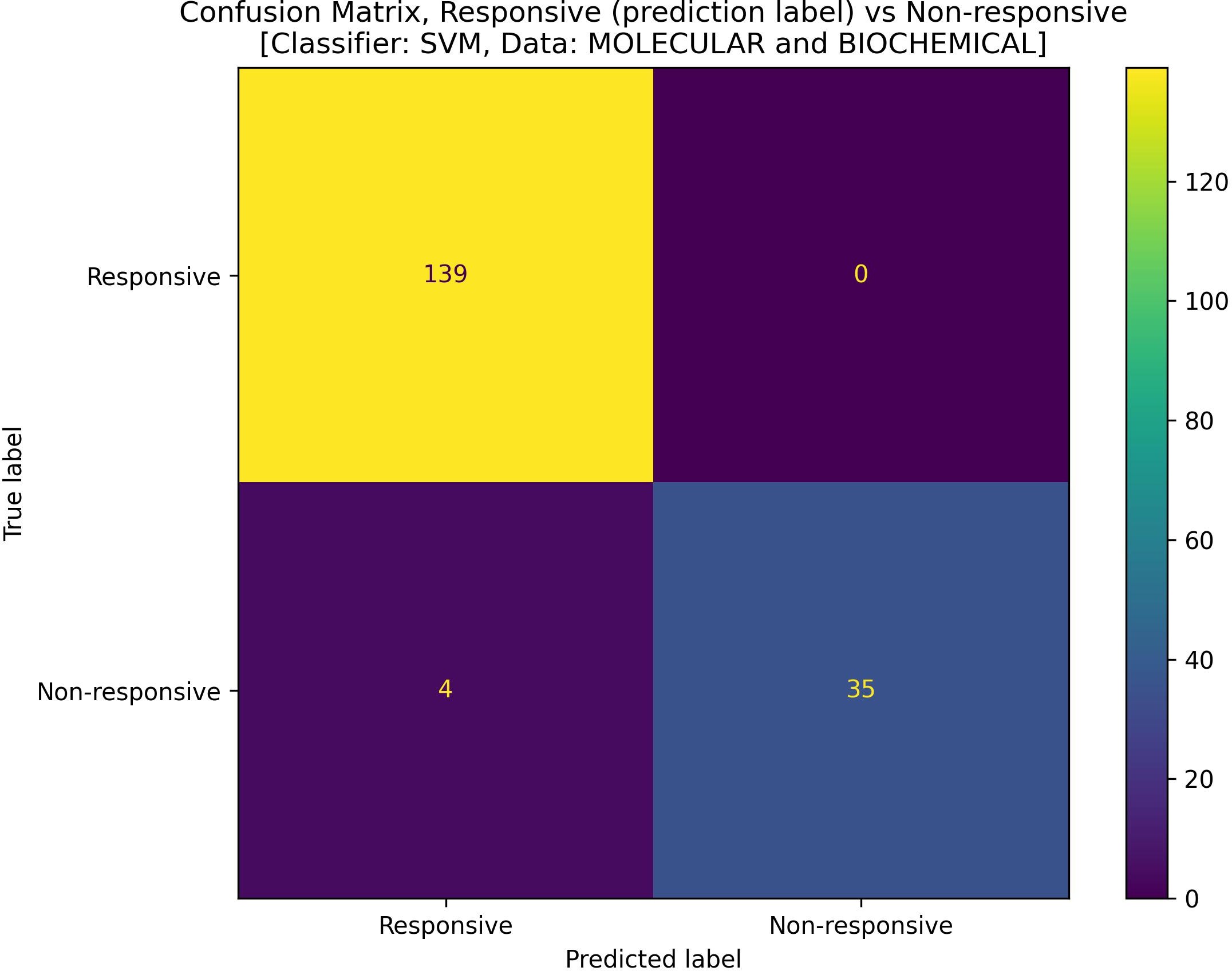


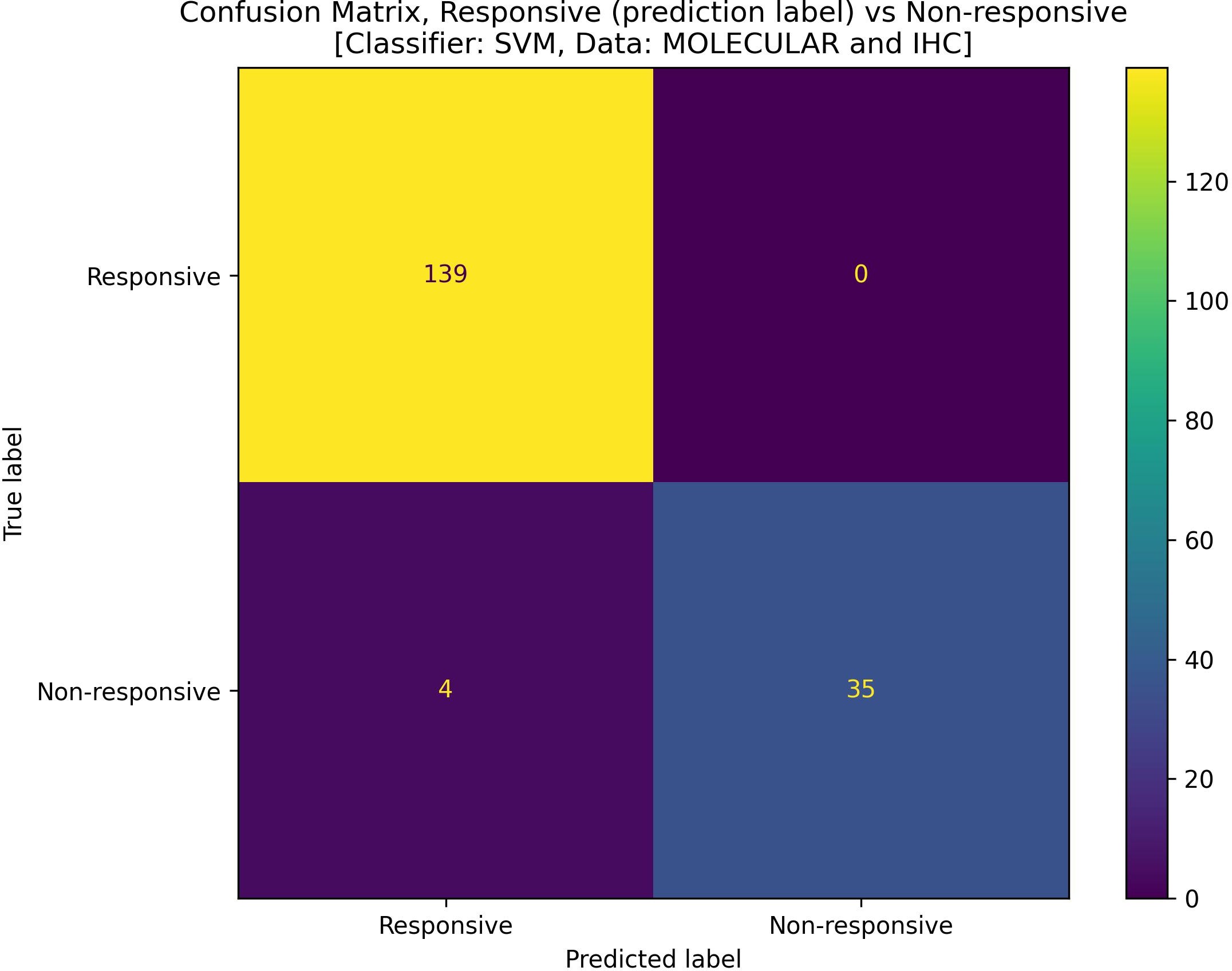


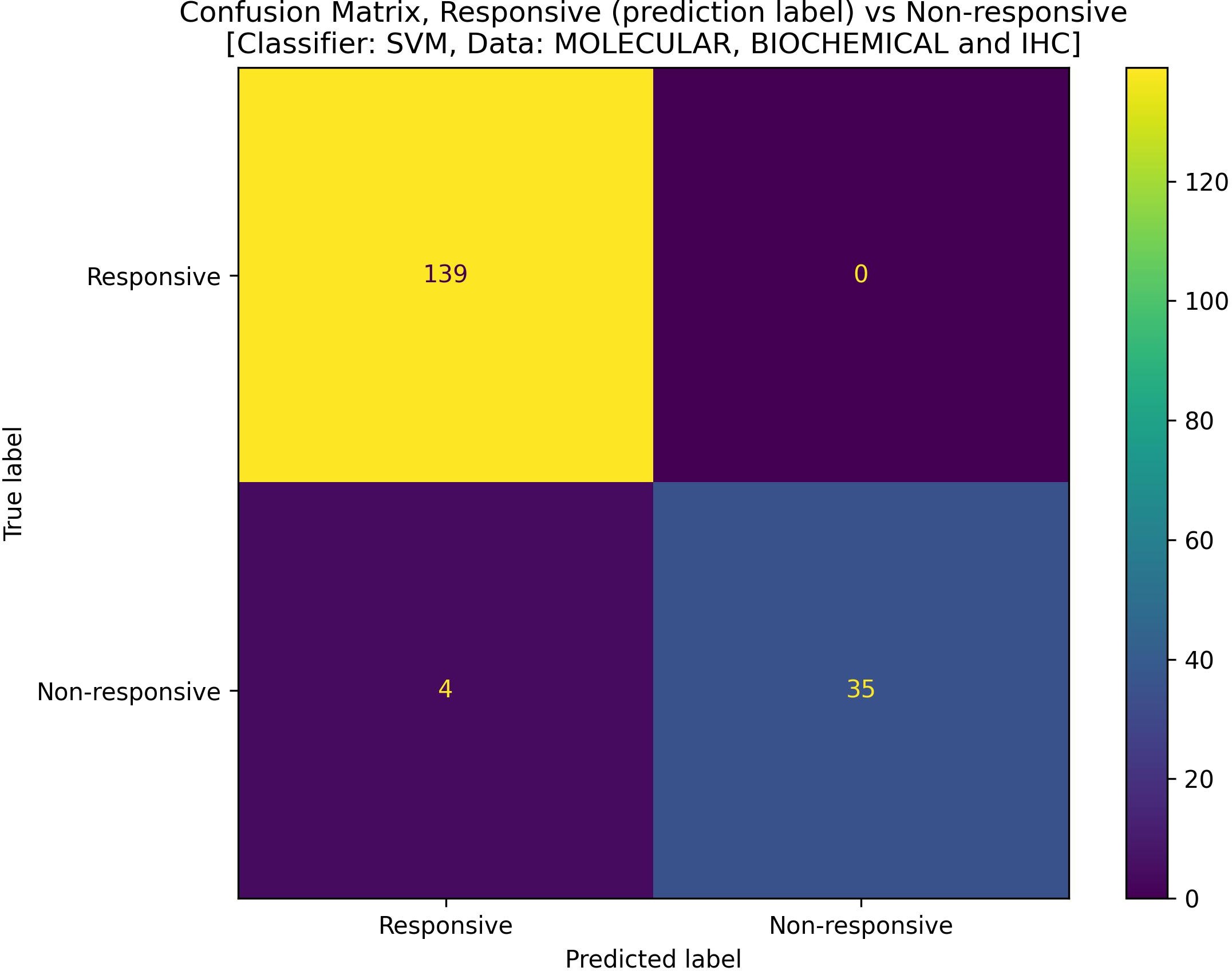


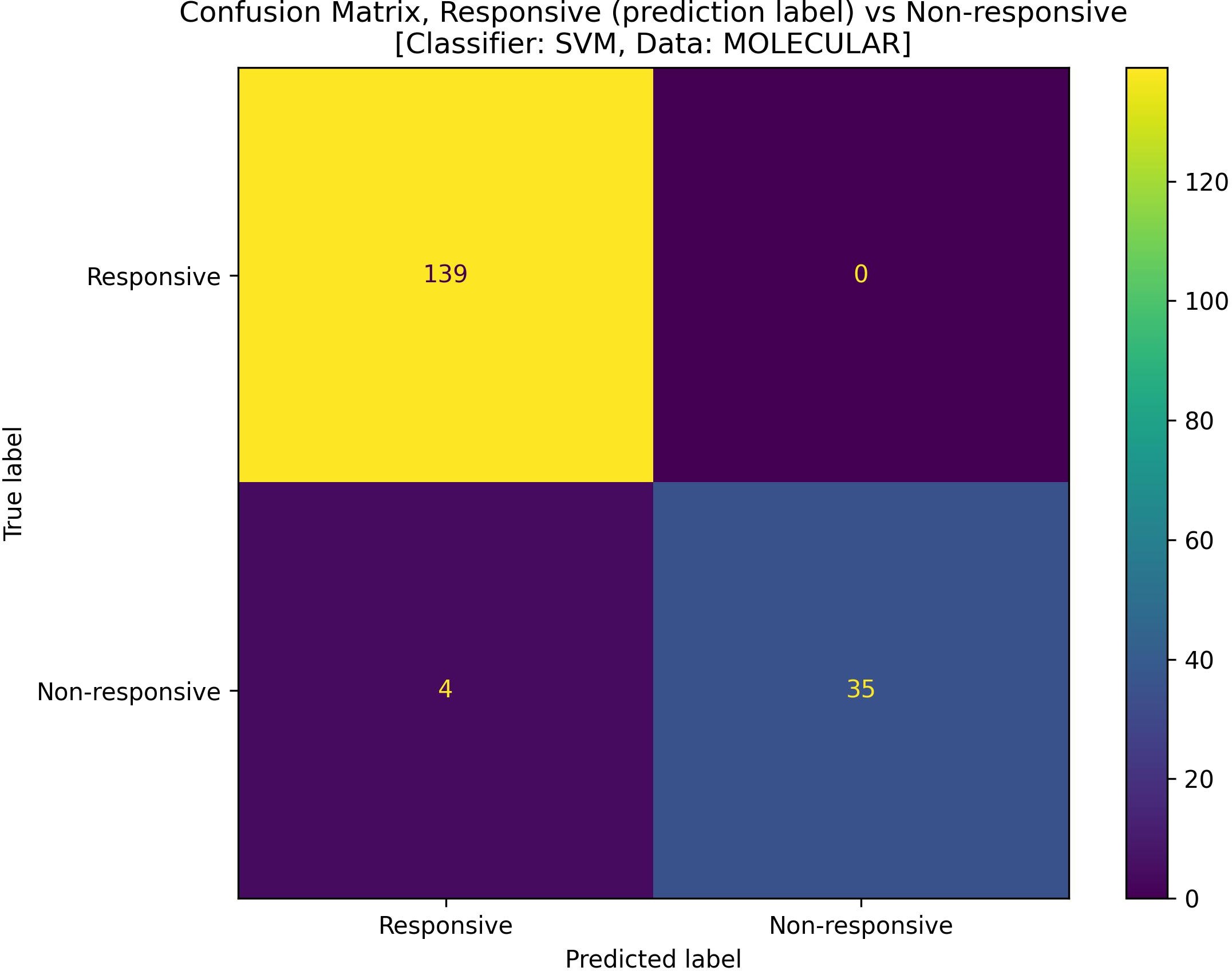


**Figures S7. ROC curves.**


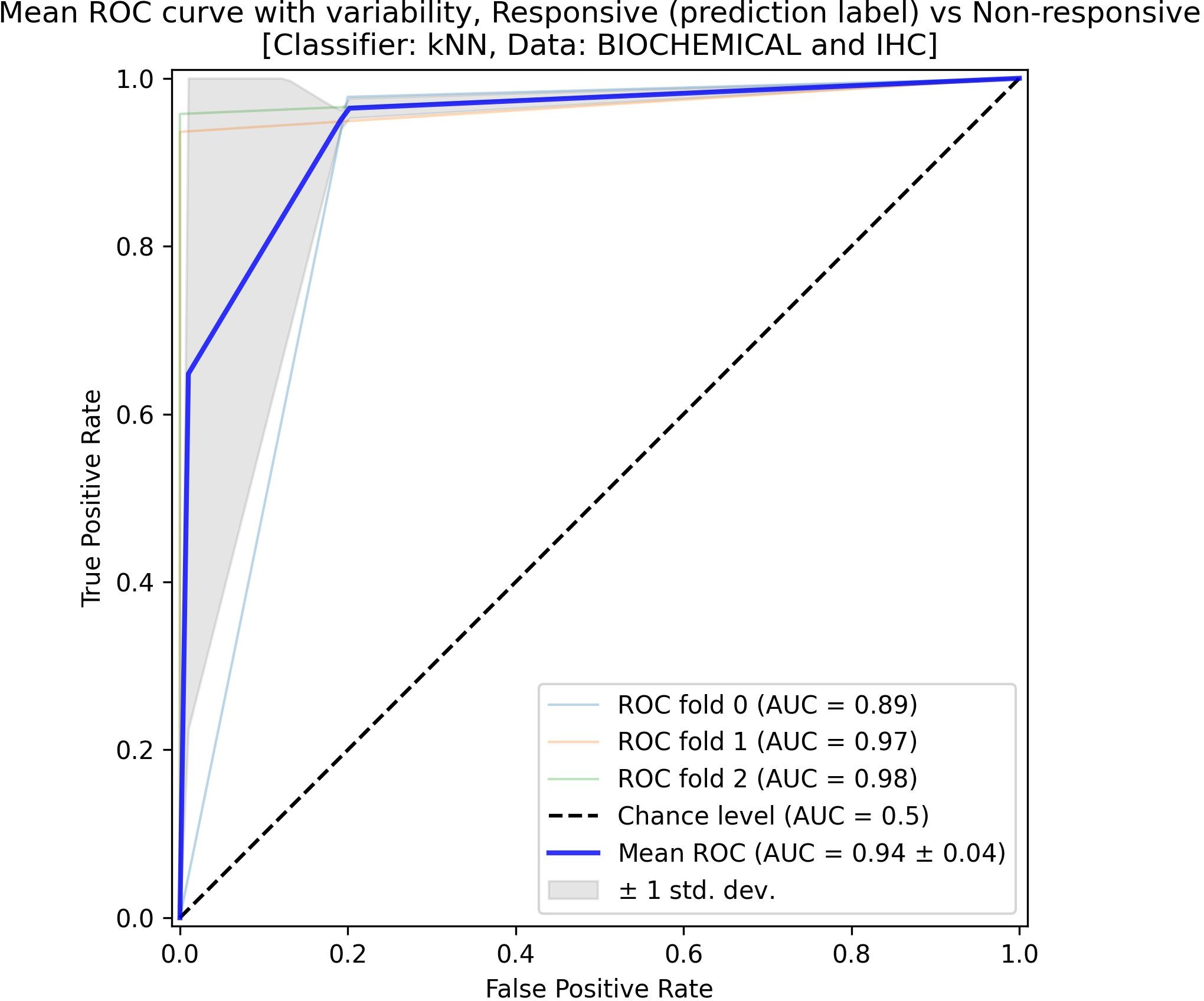


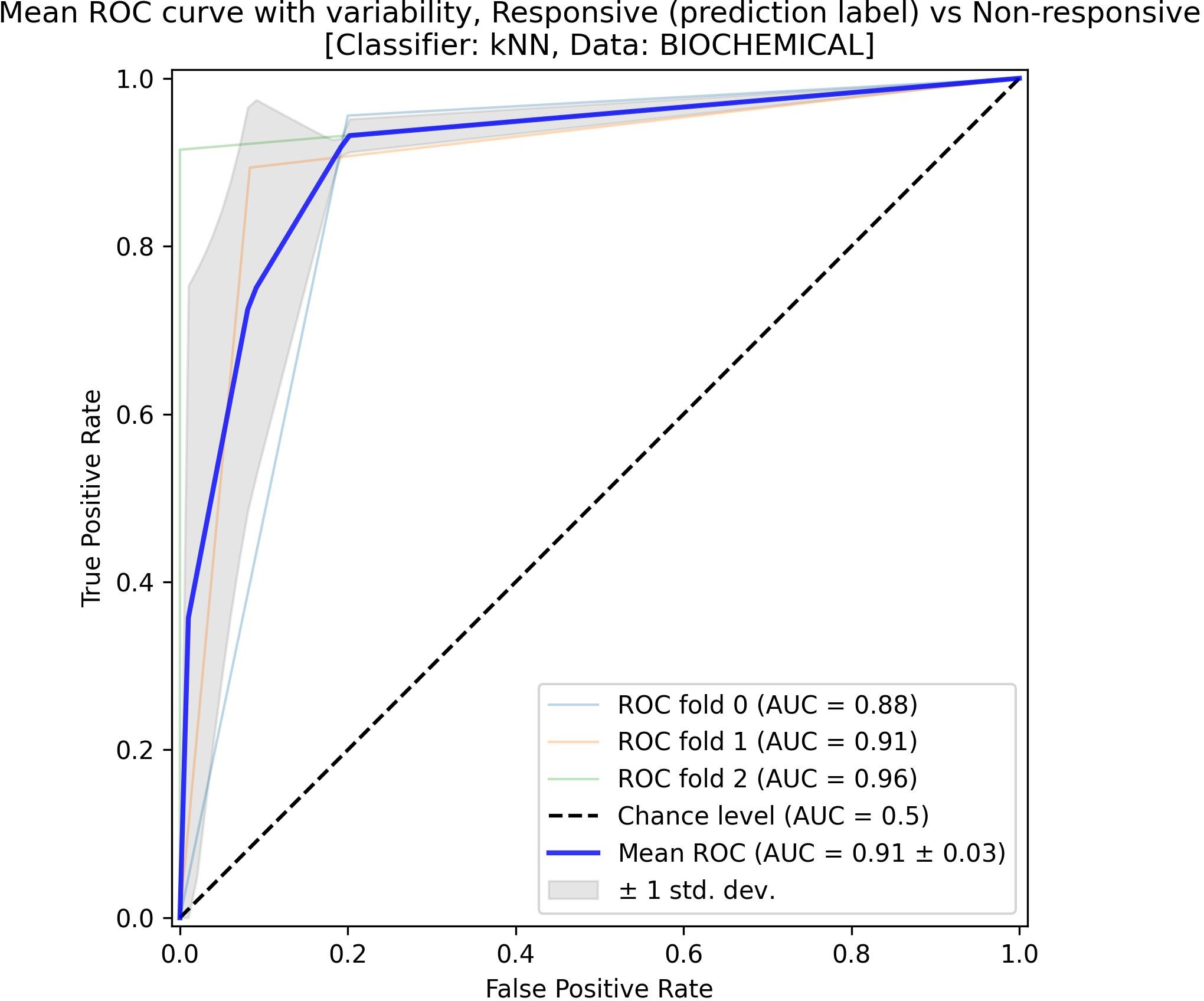


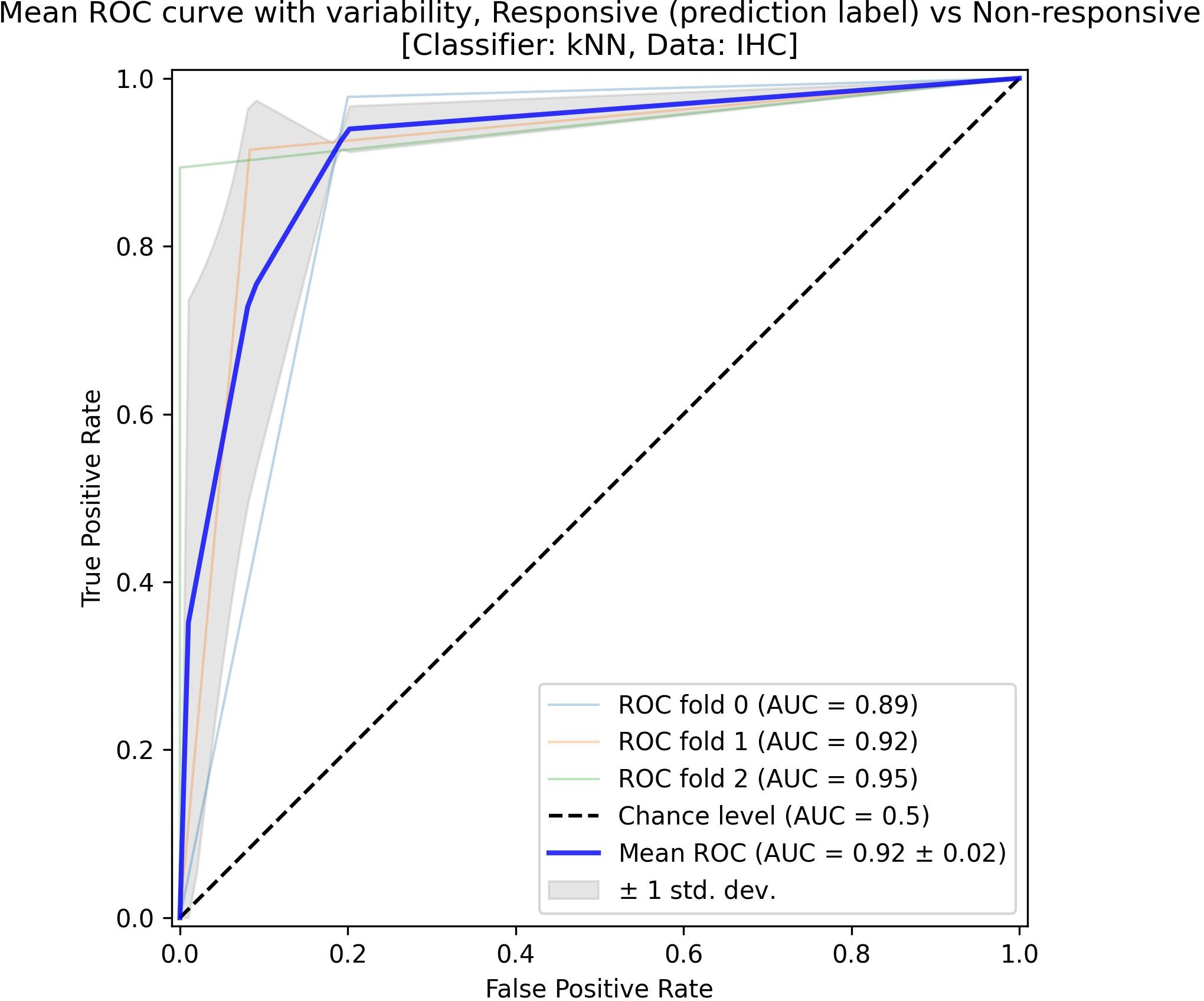


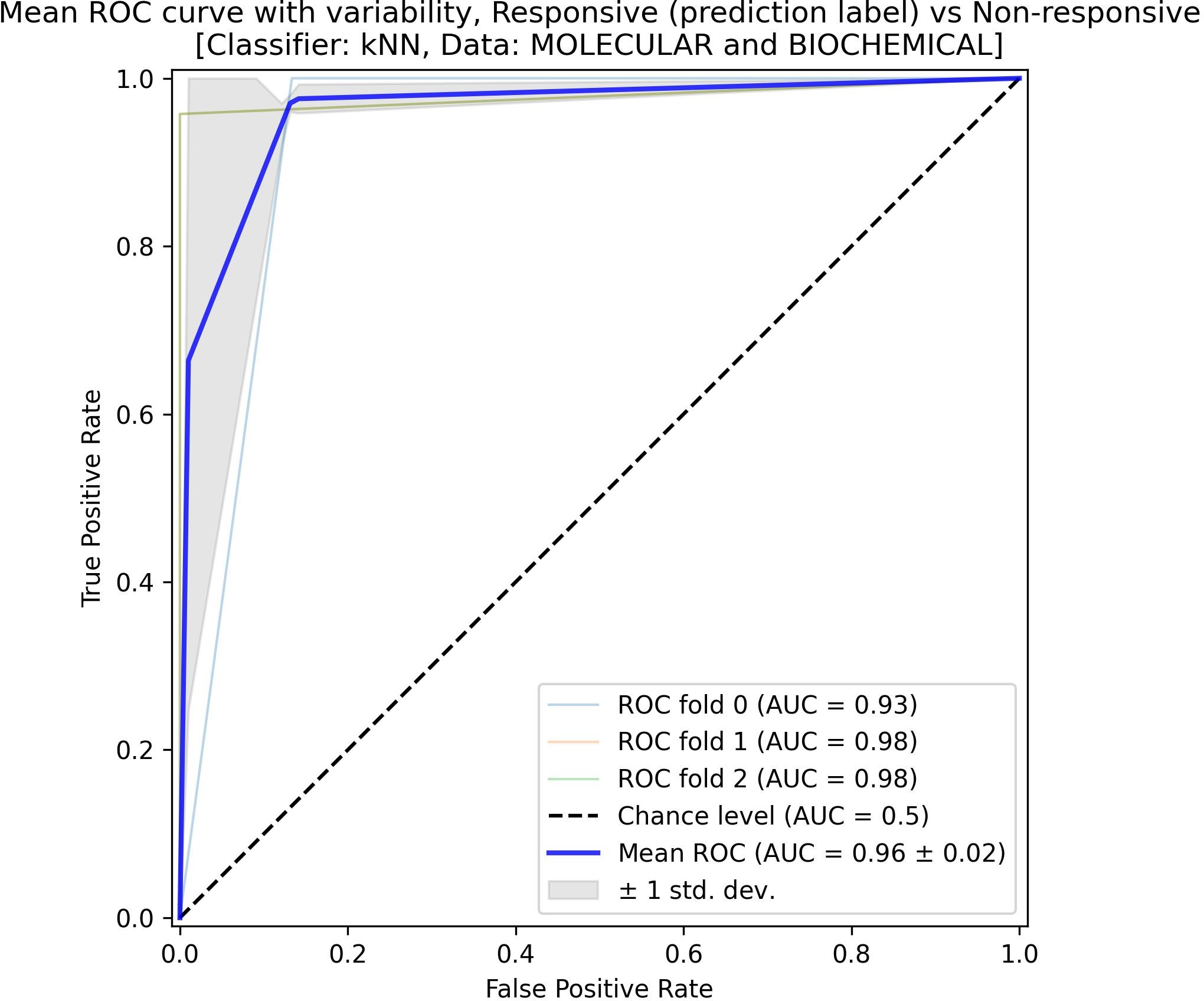


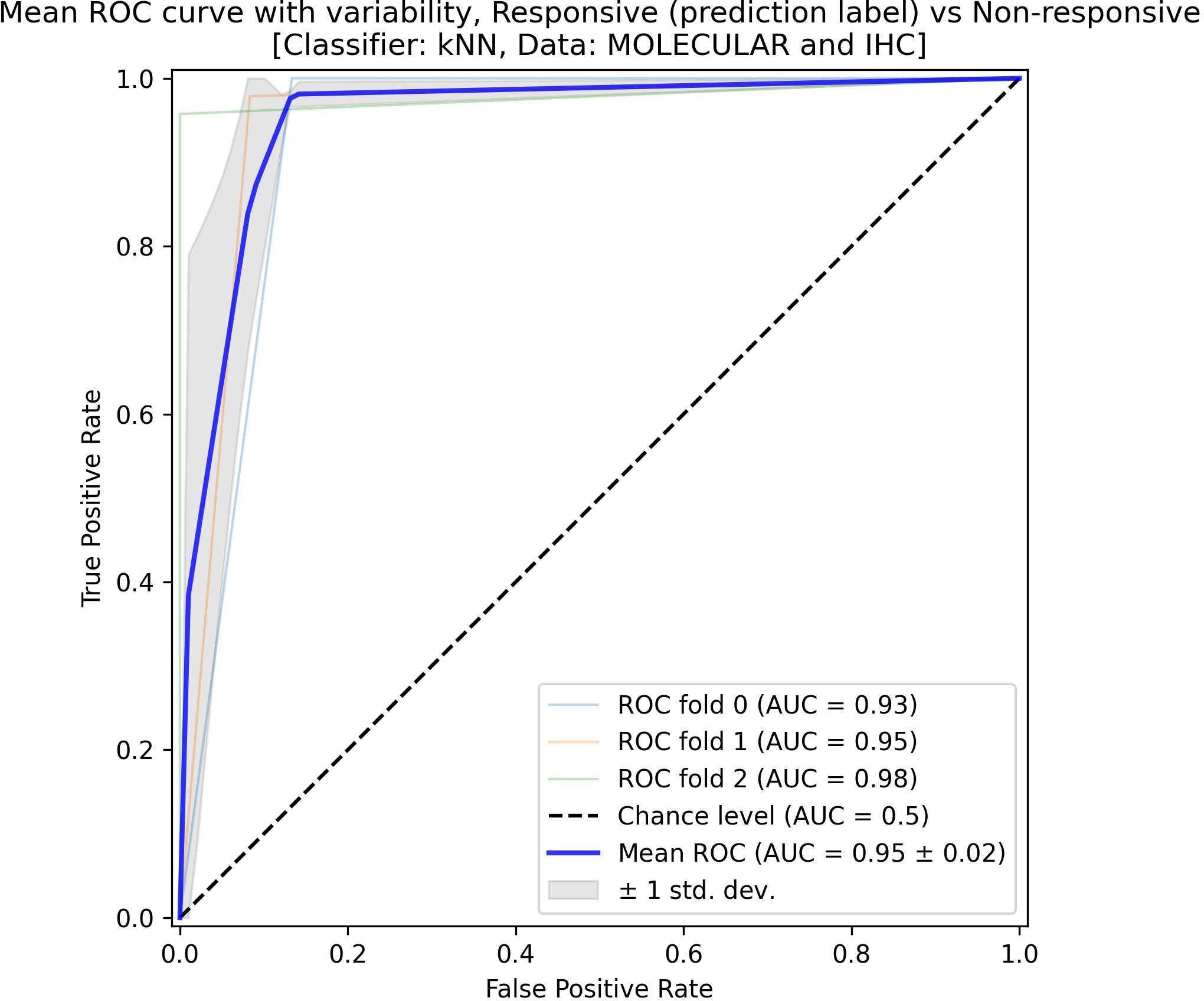


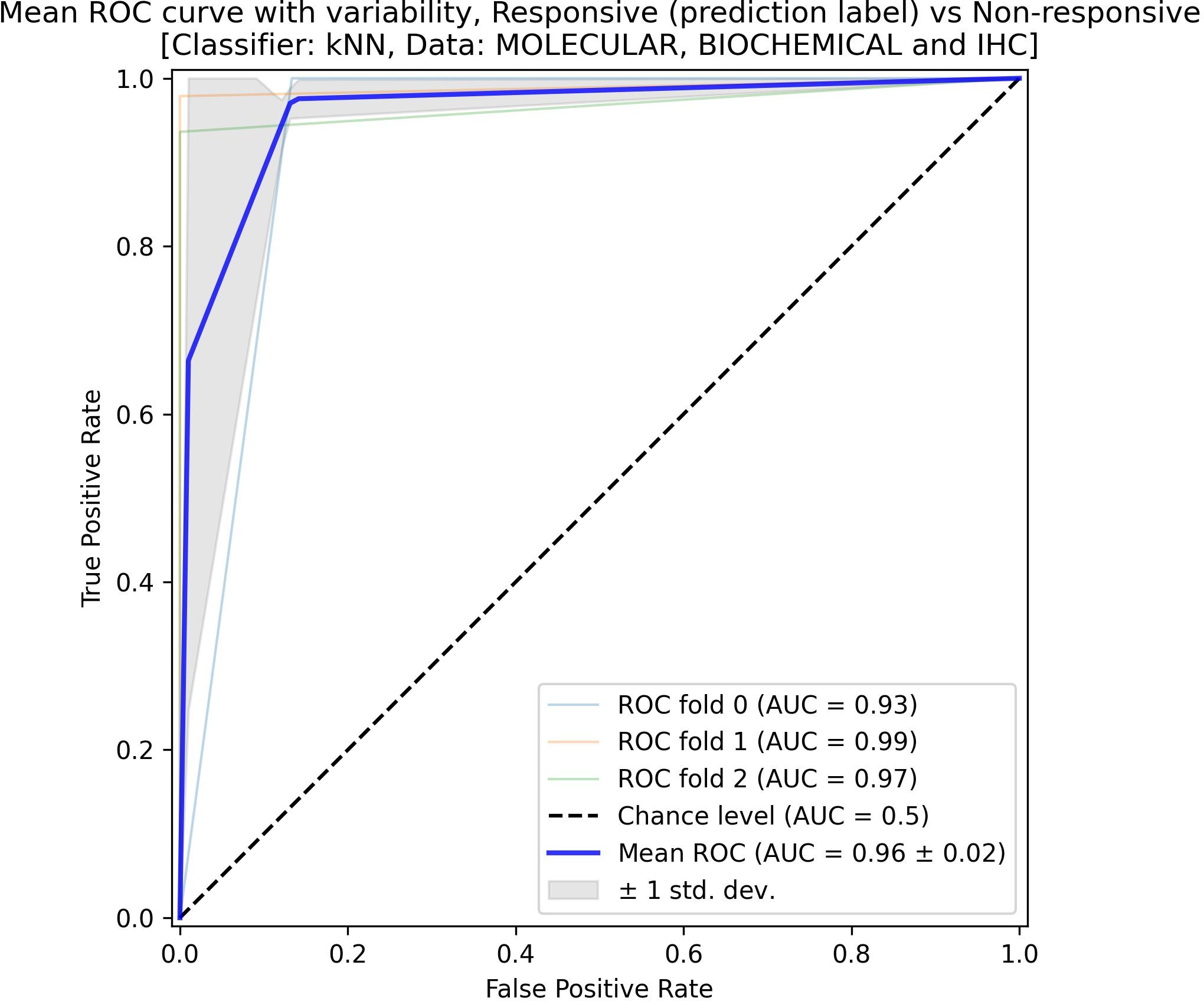


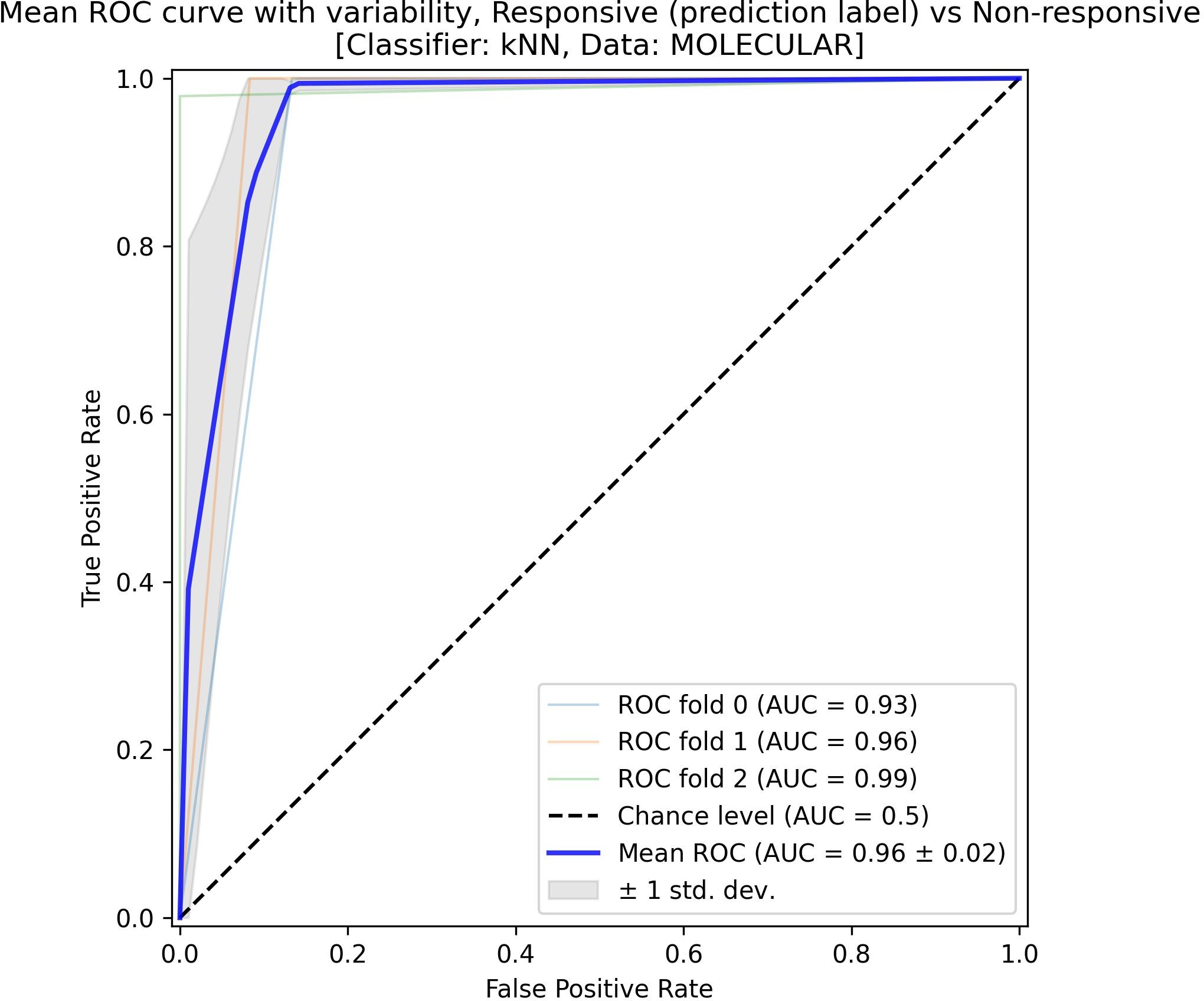


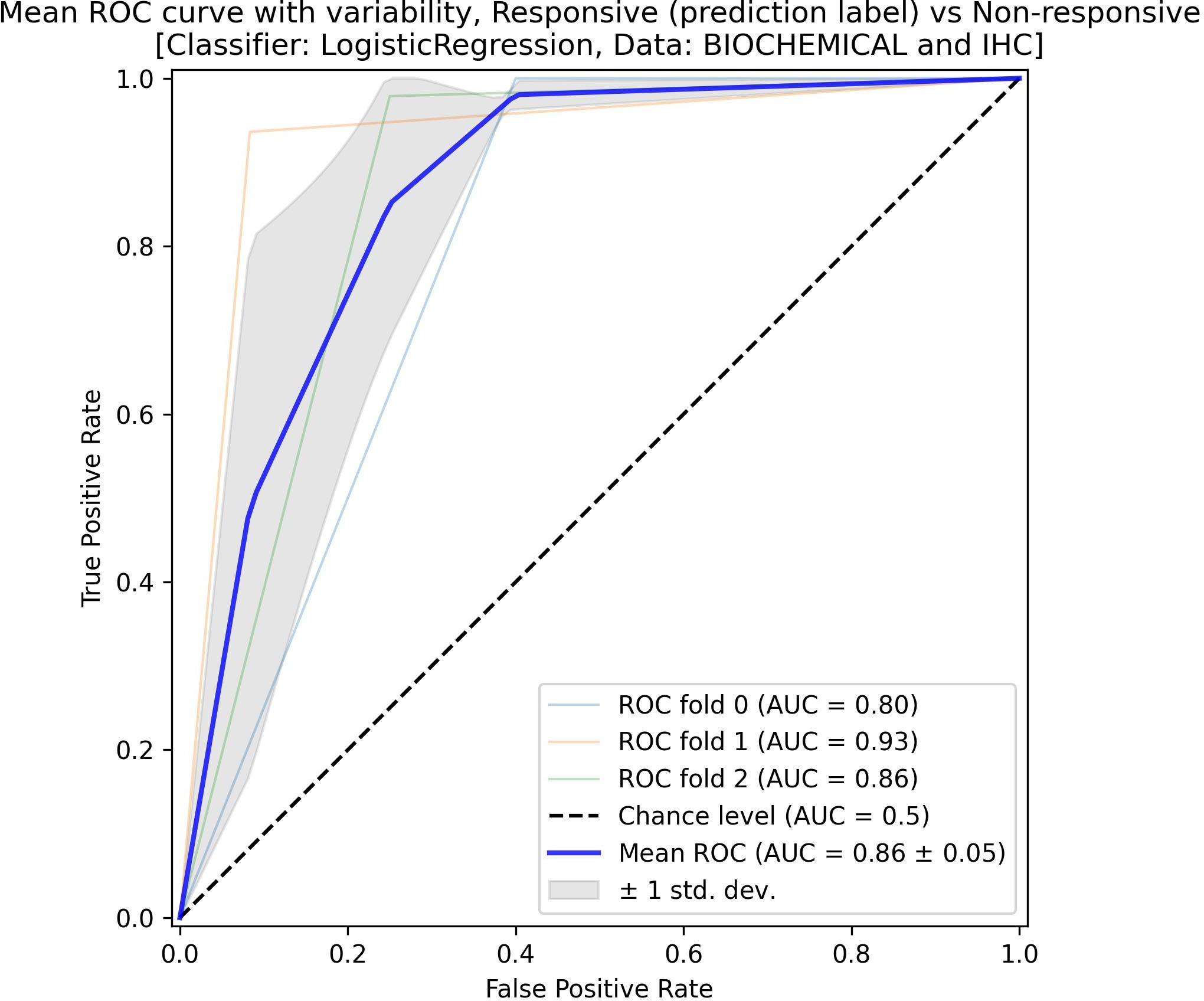


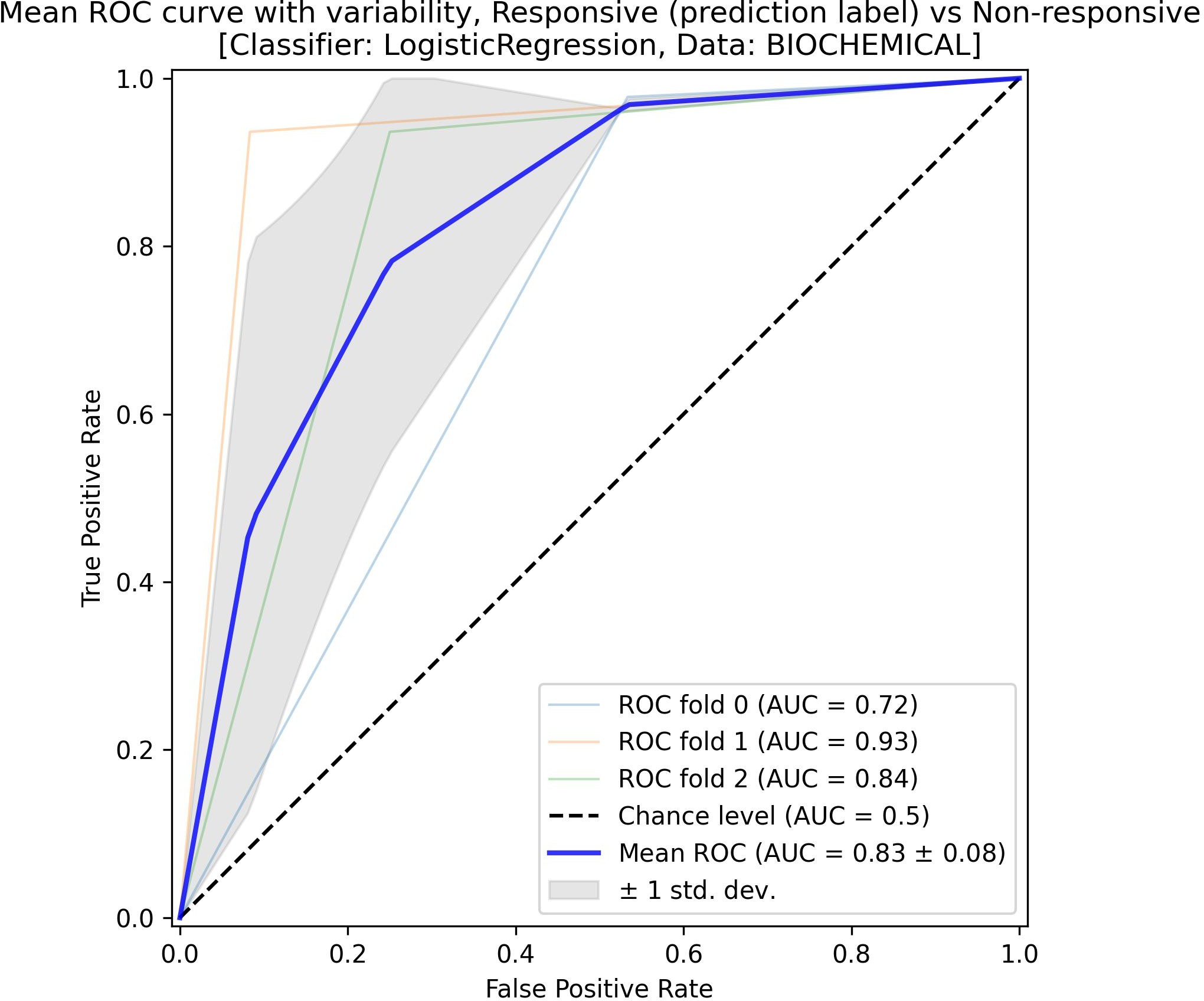


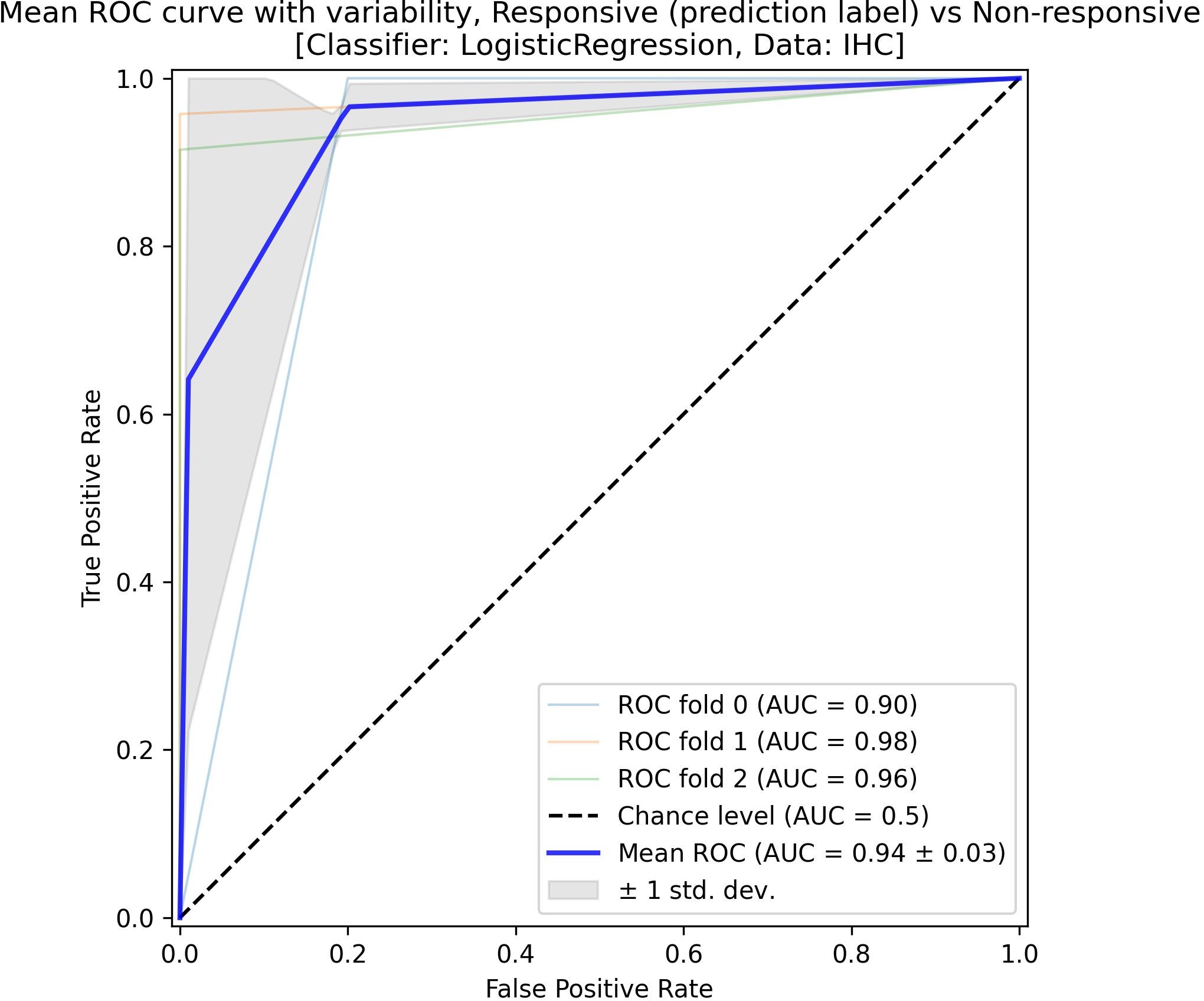


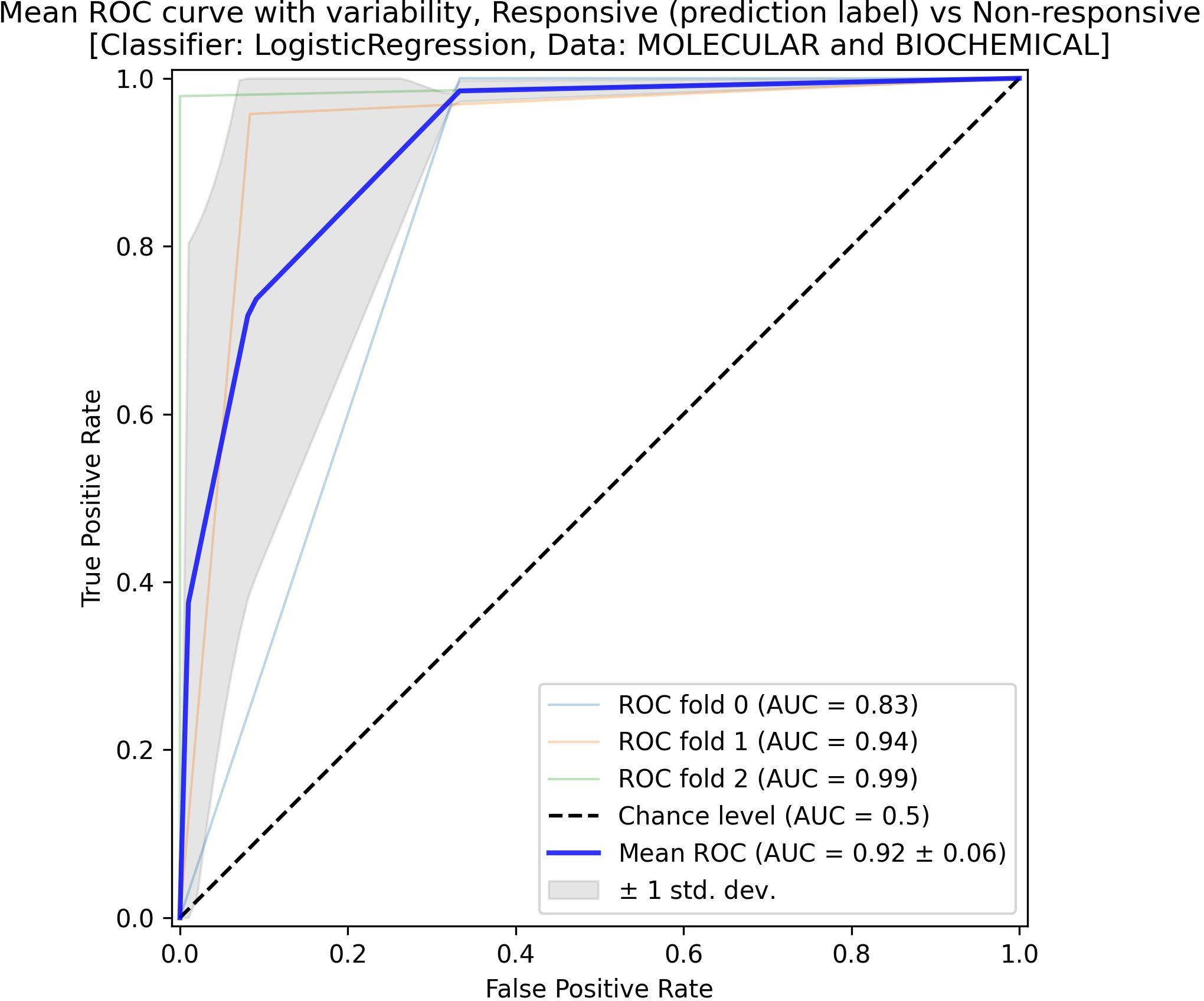


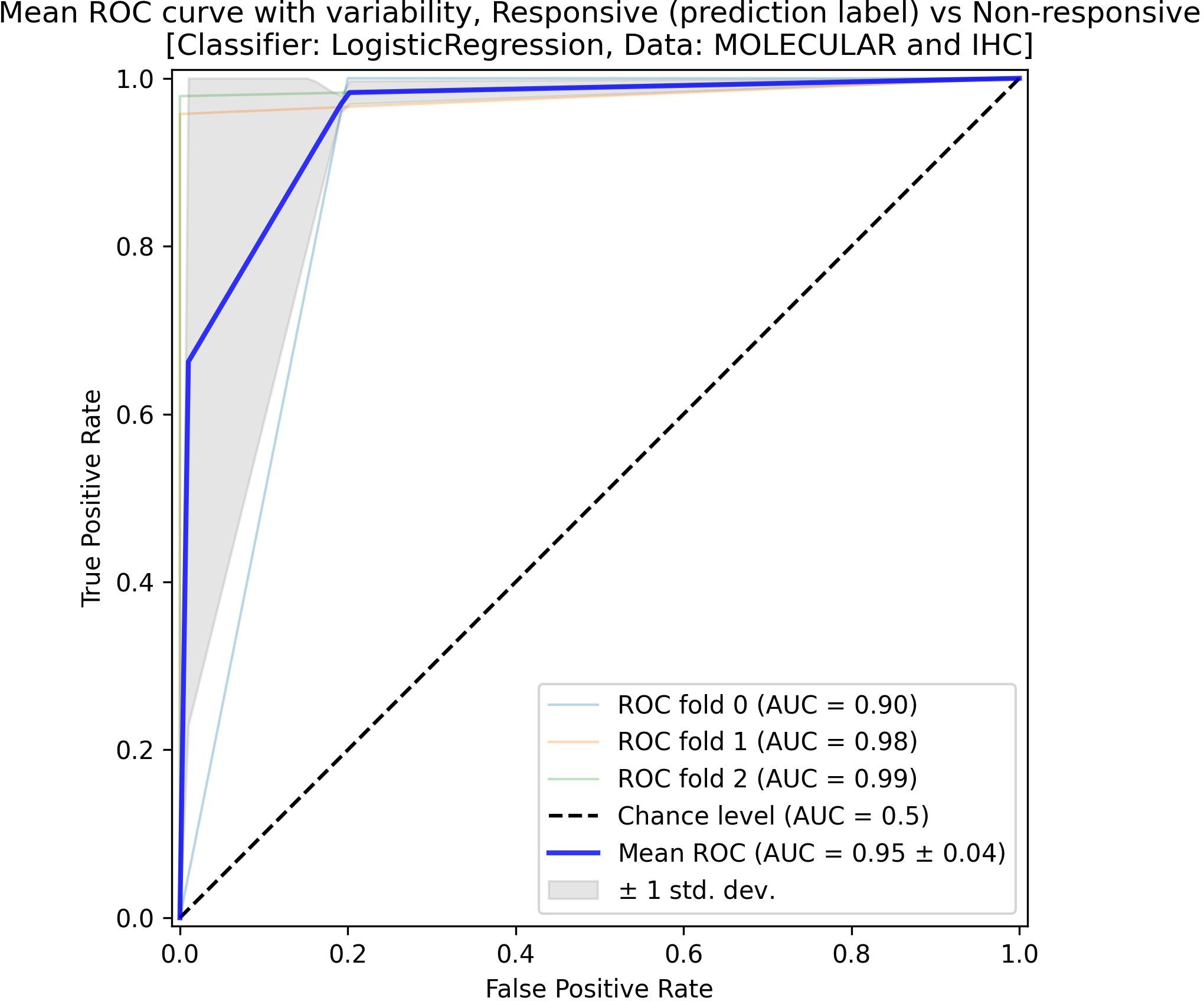


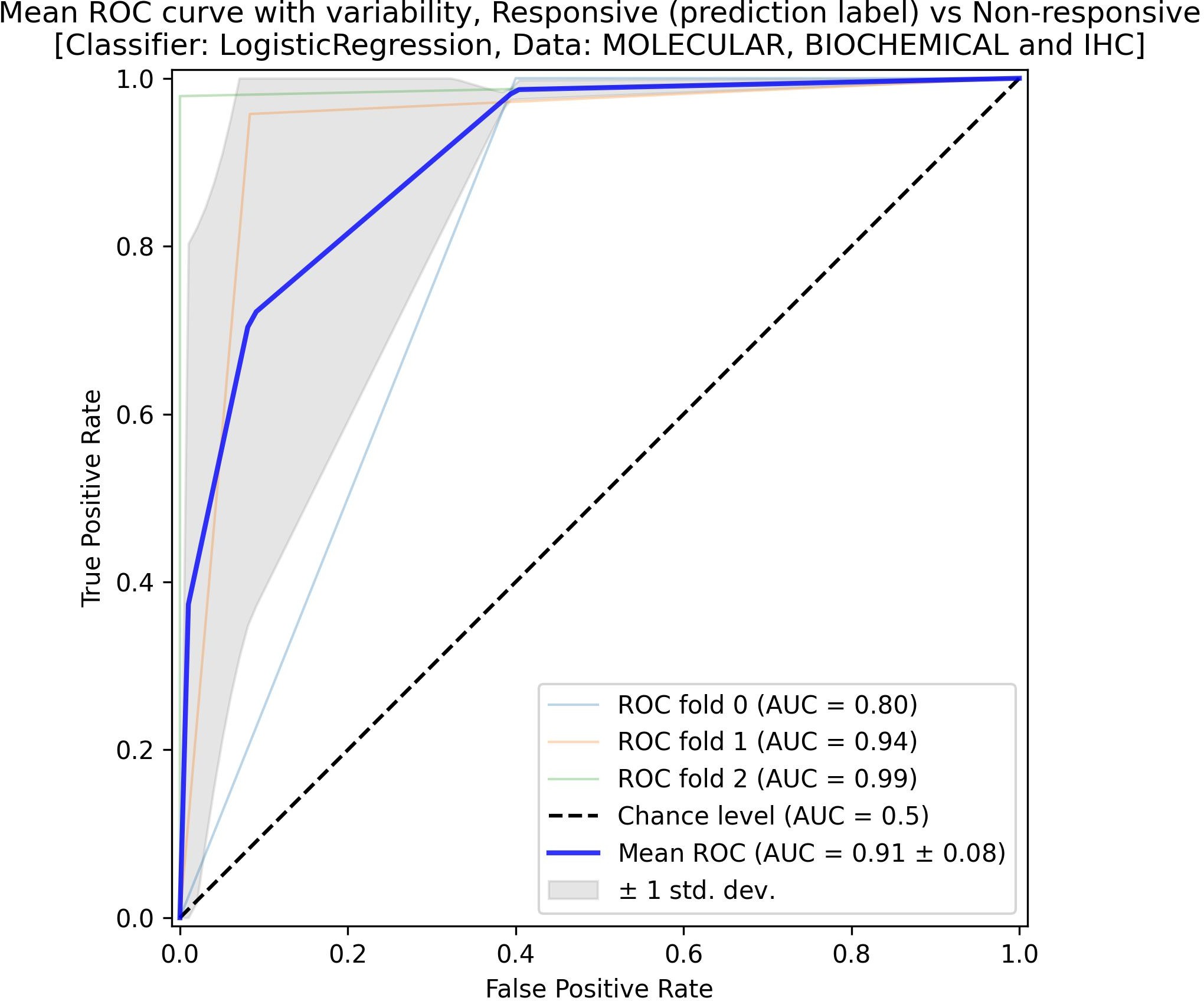


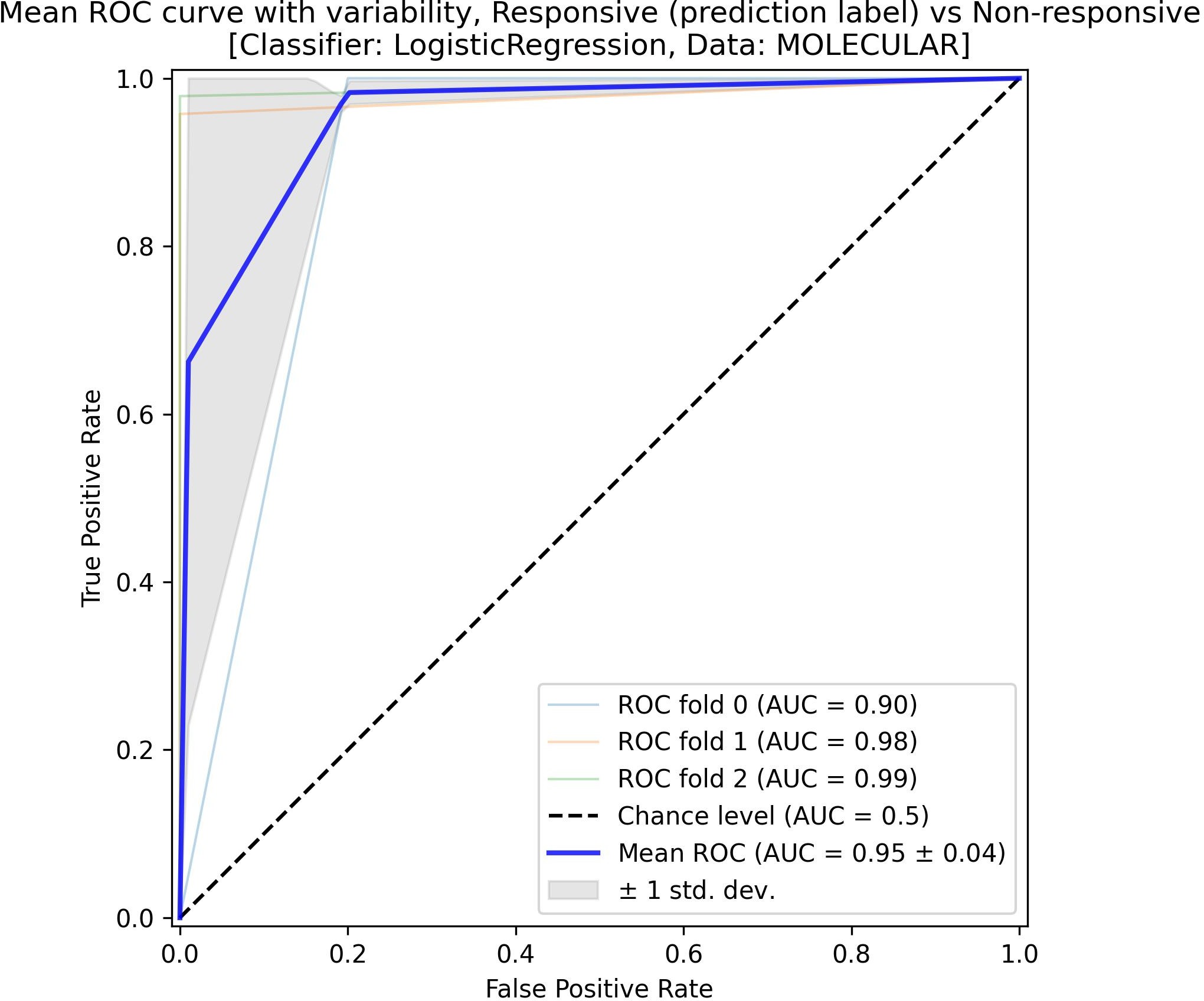


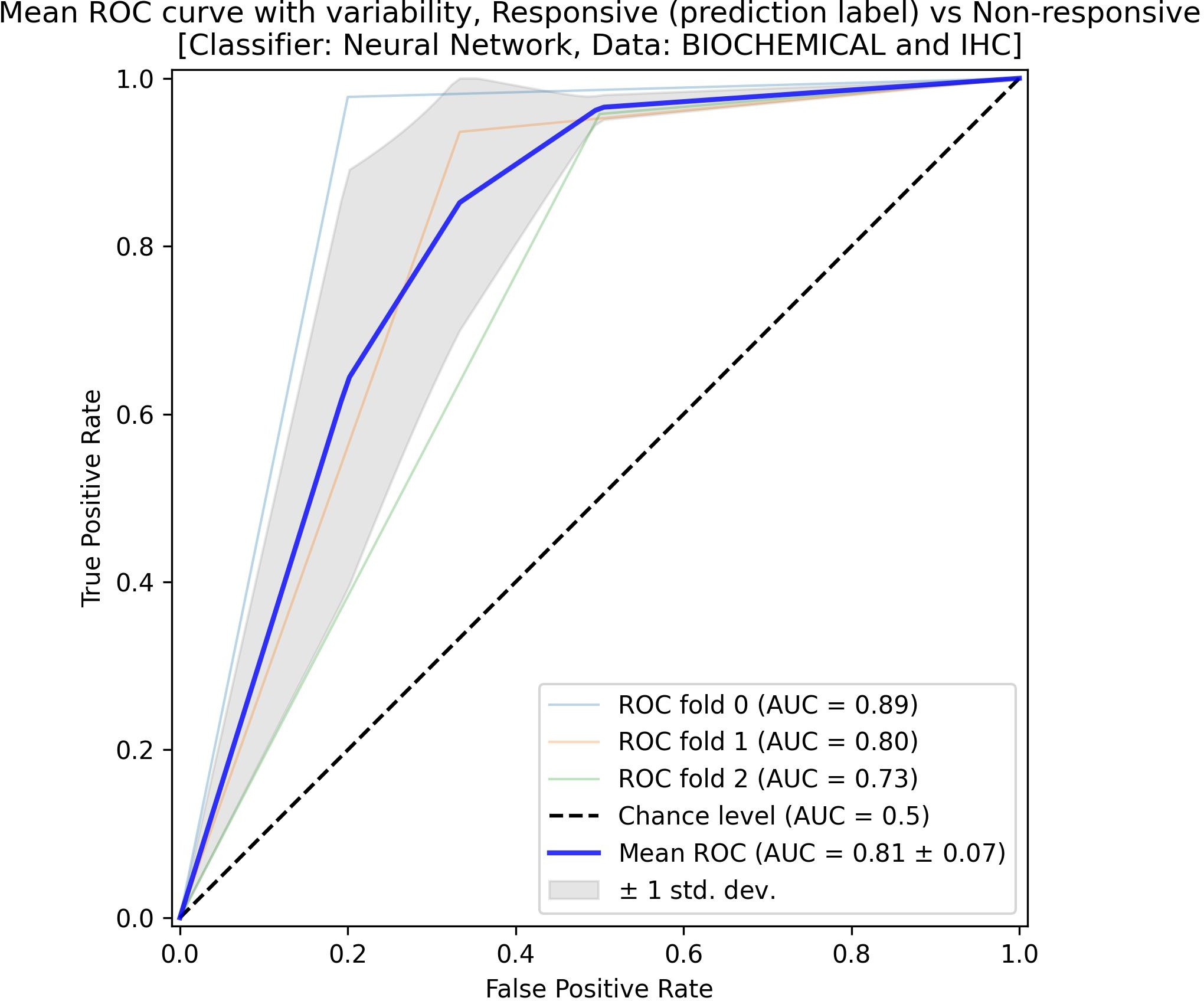


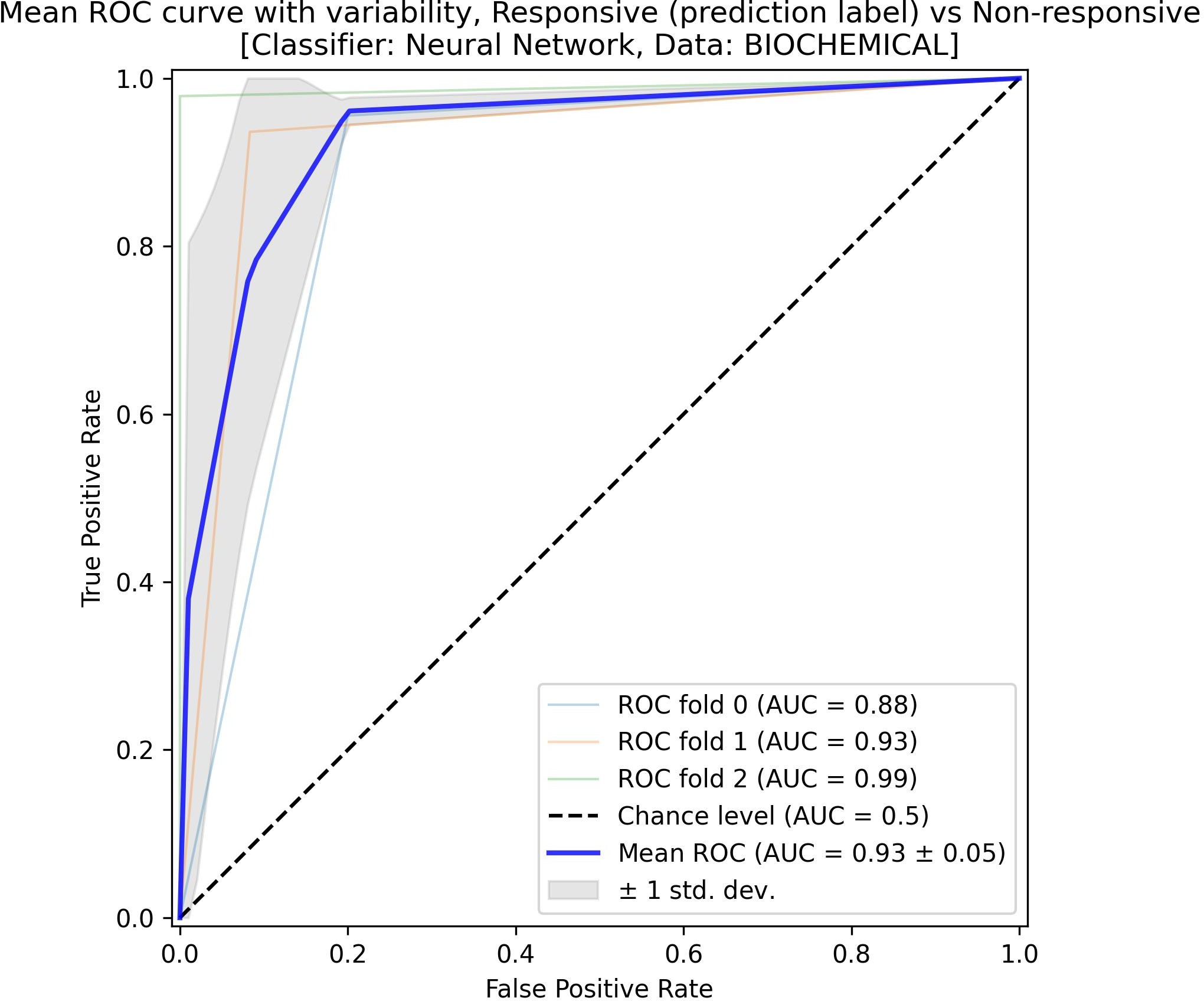


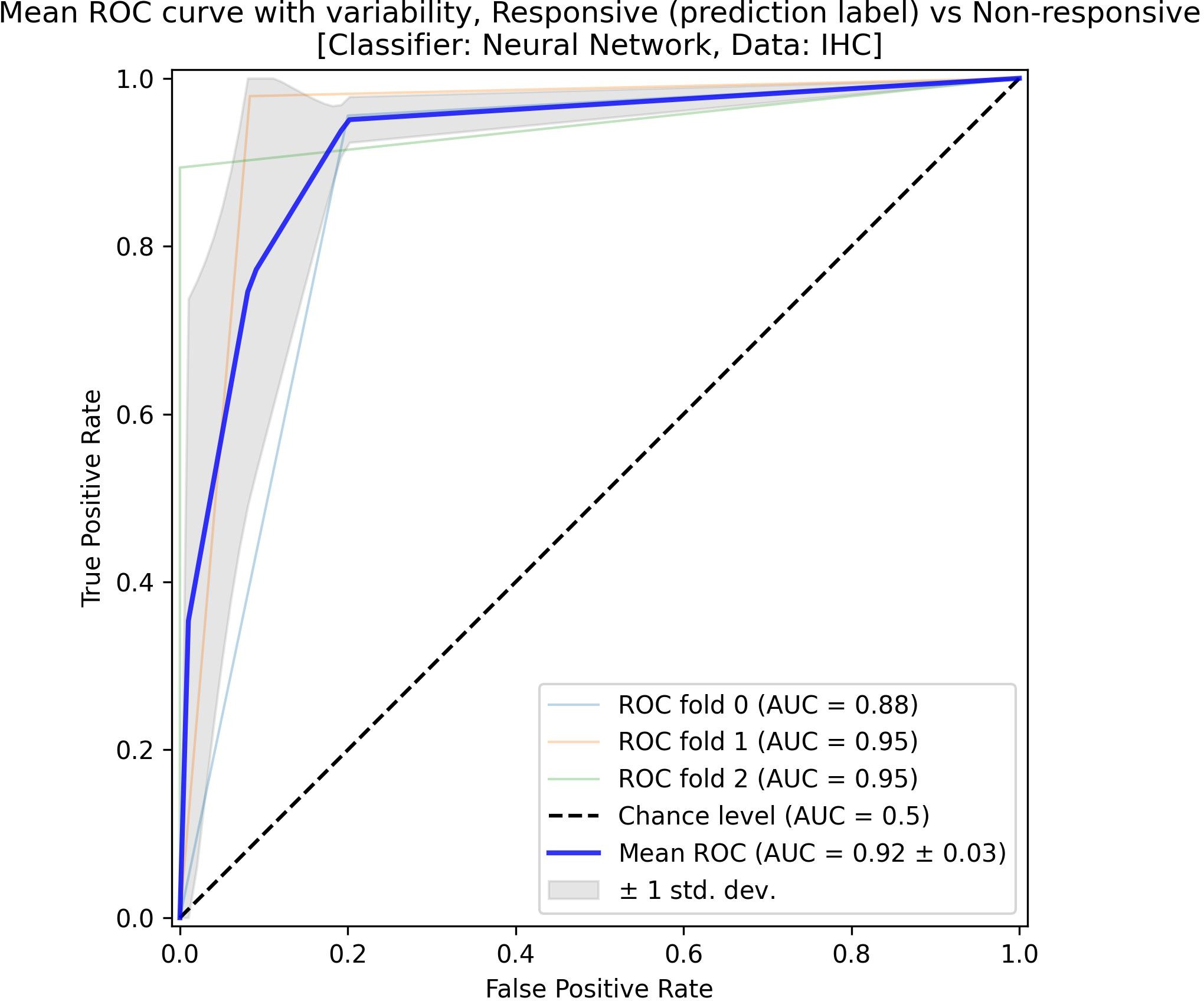


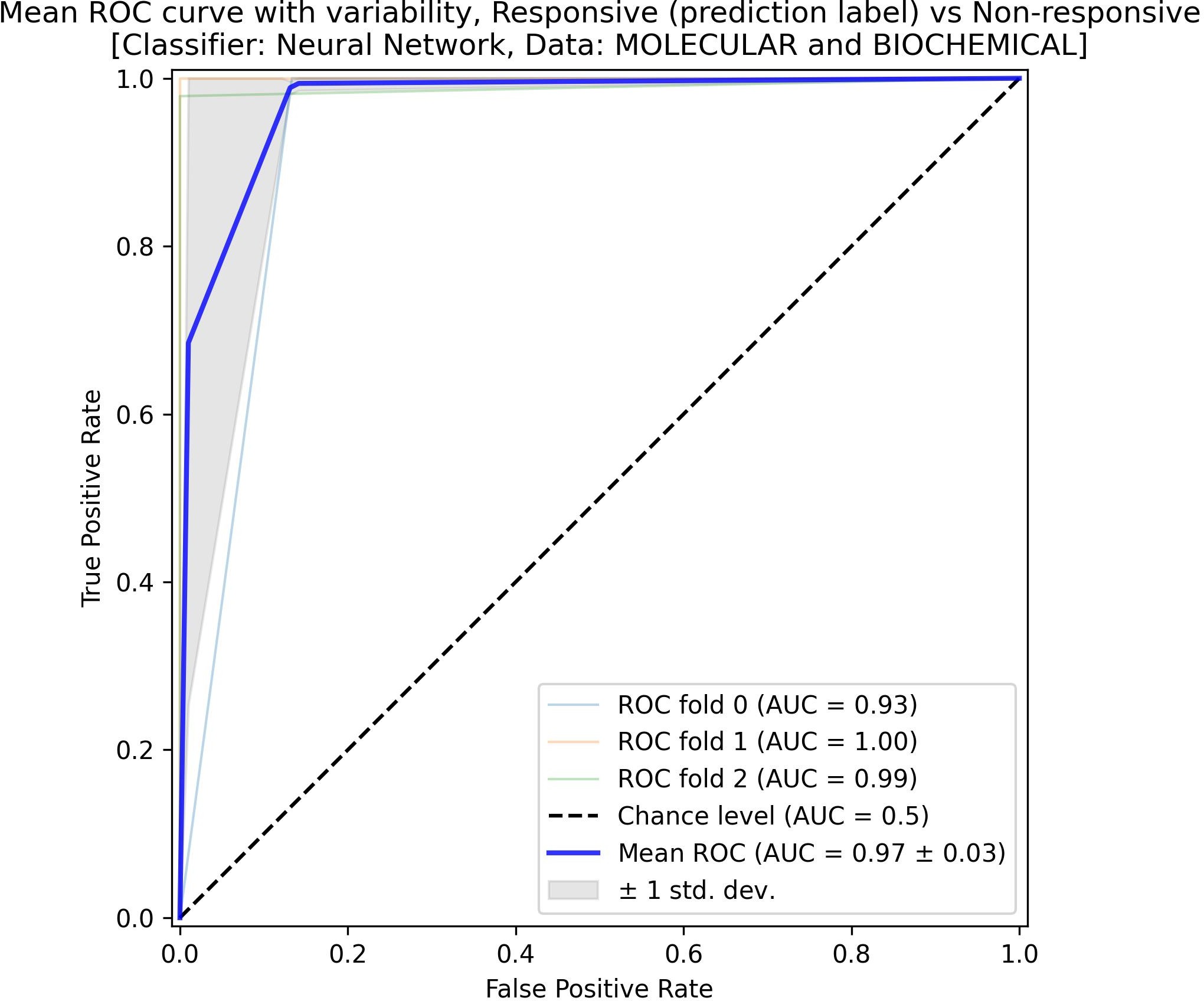


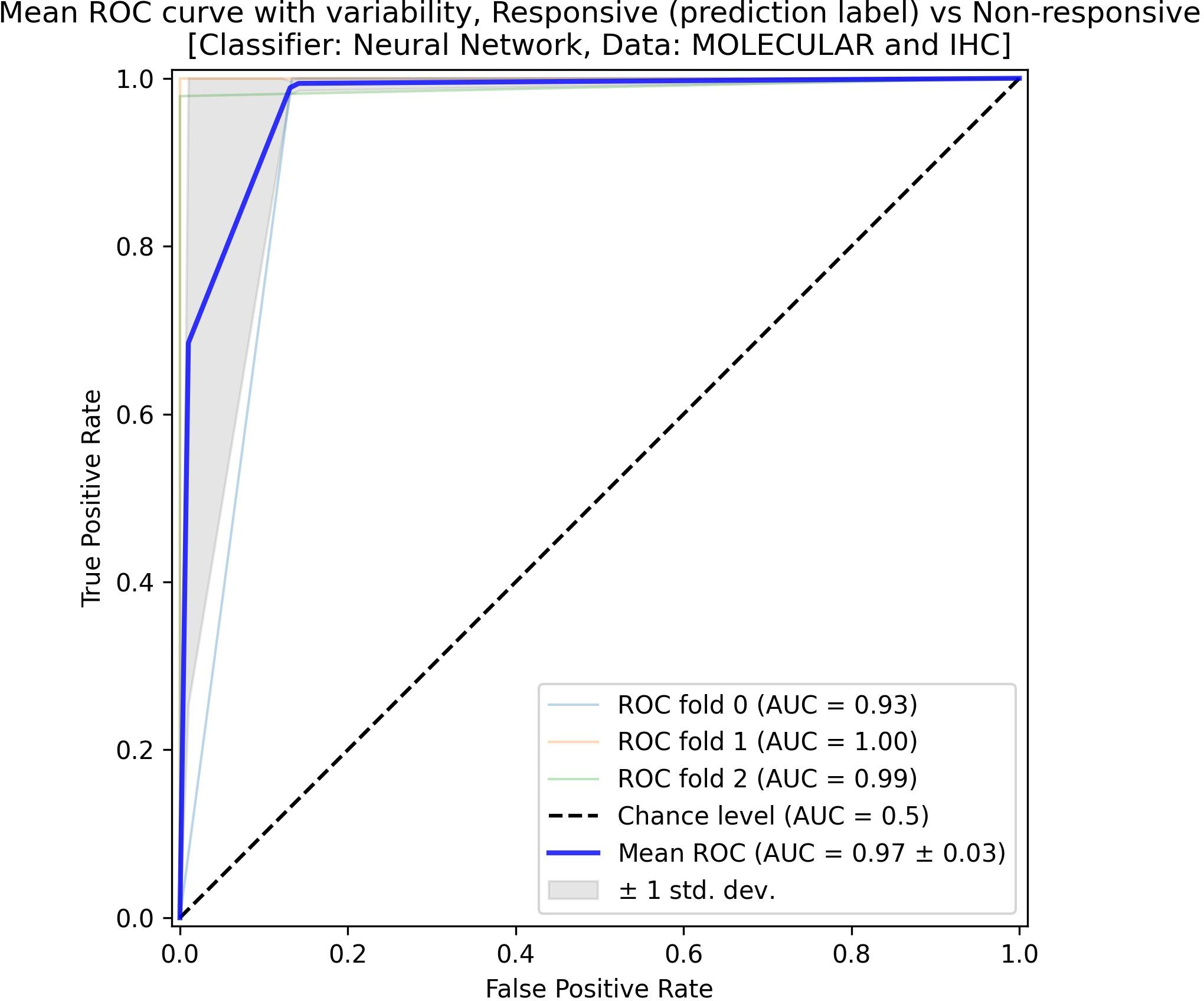


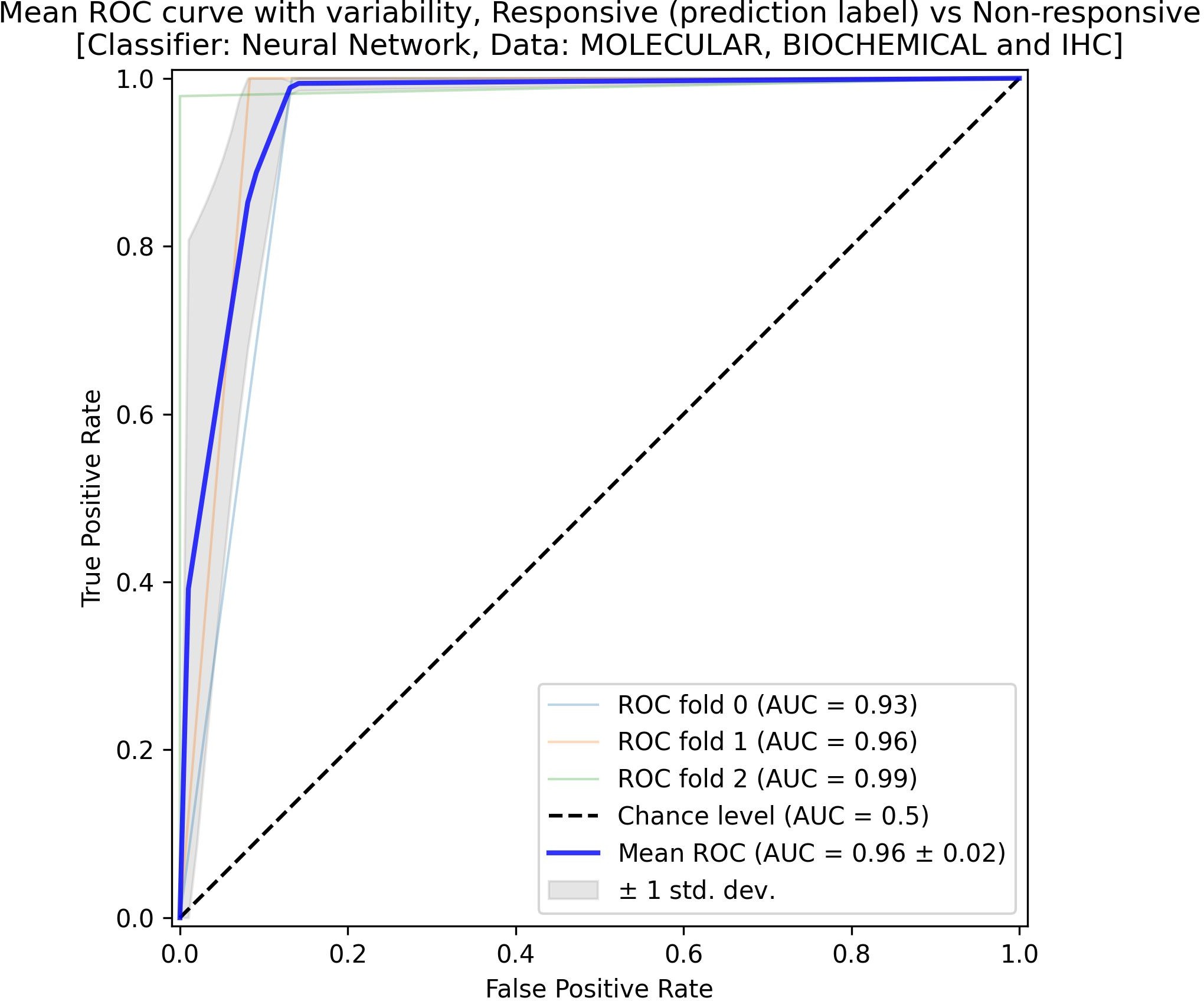


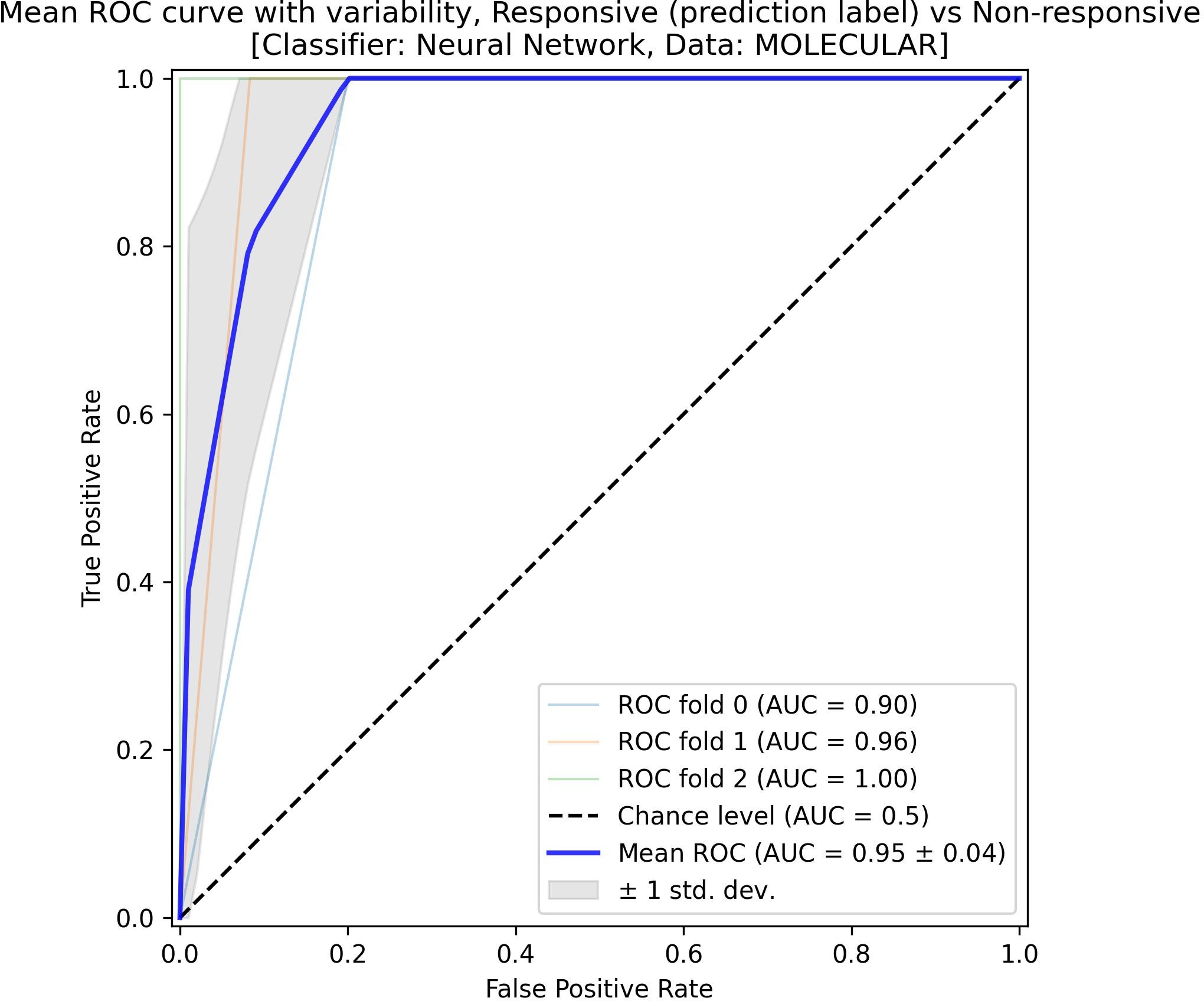


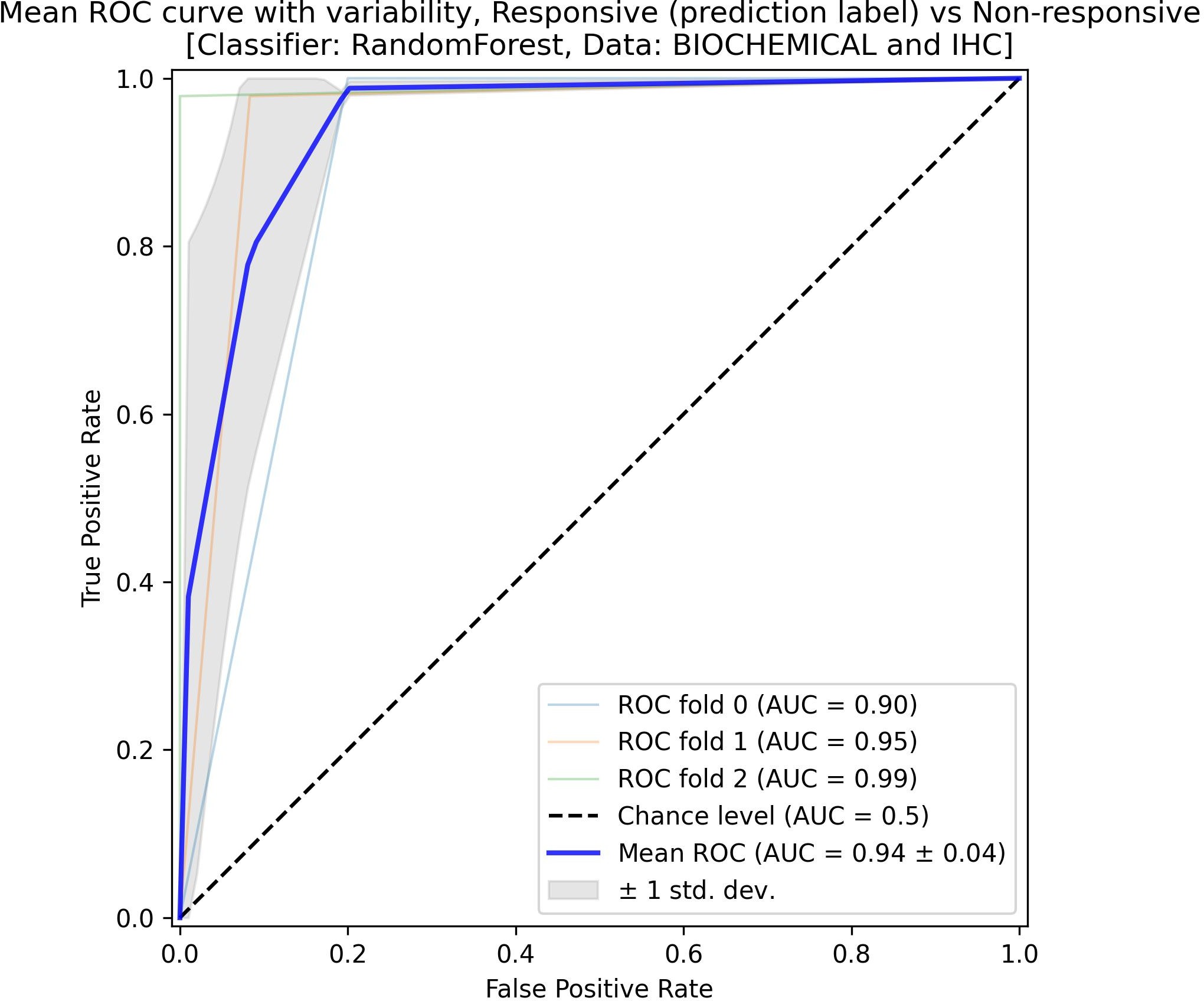


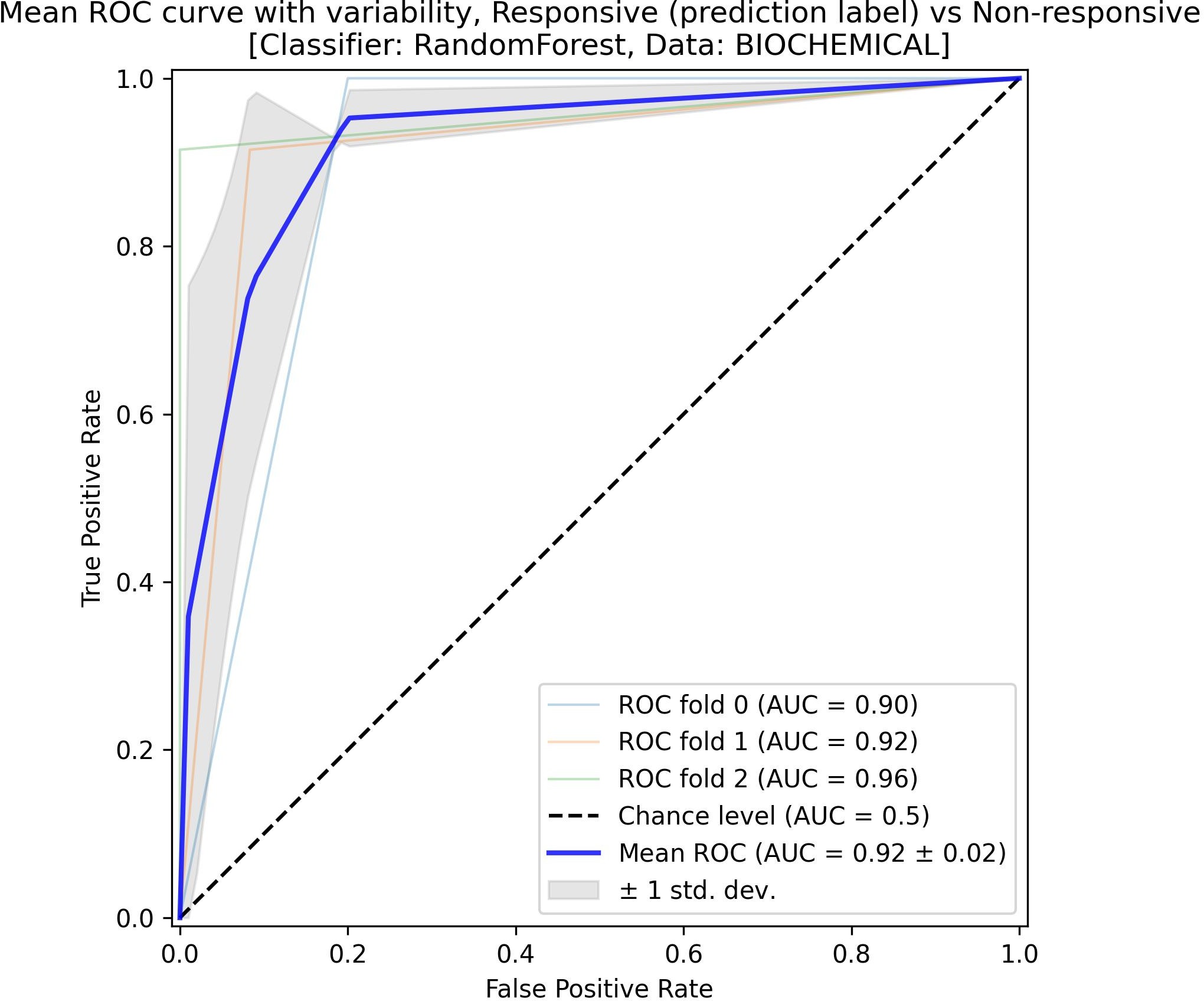


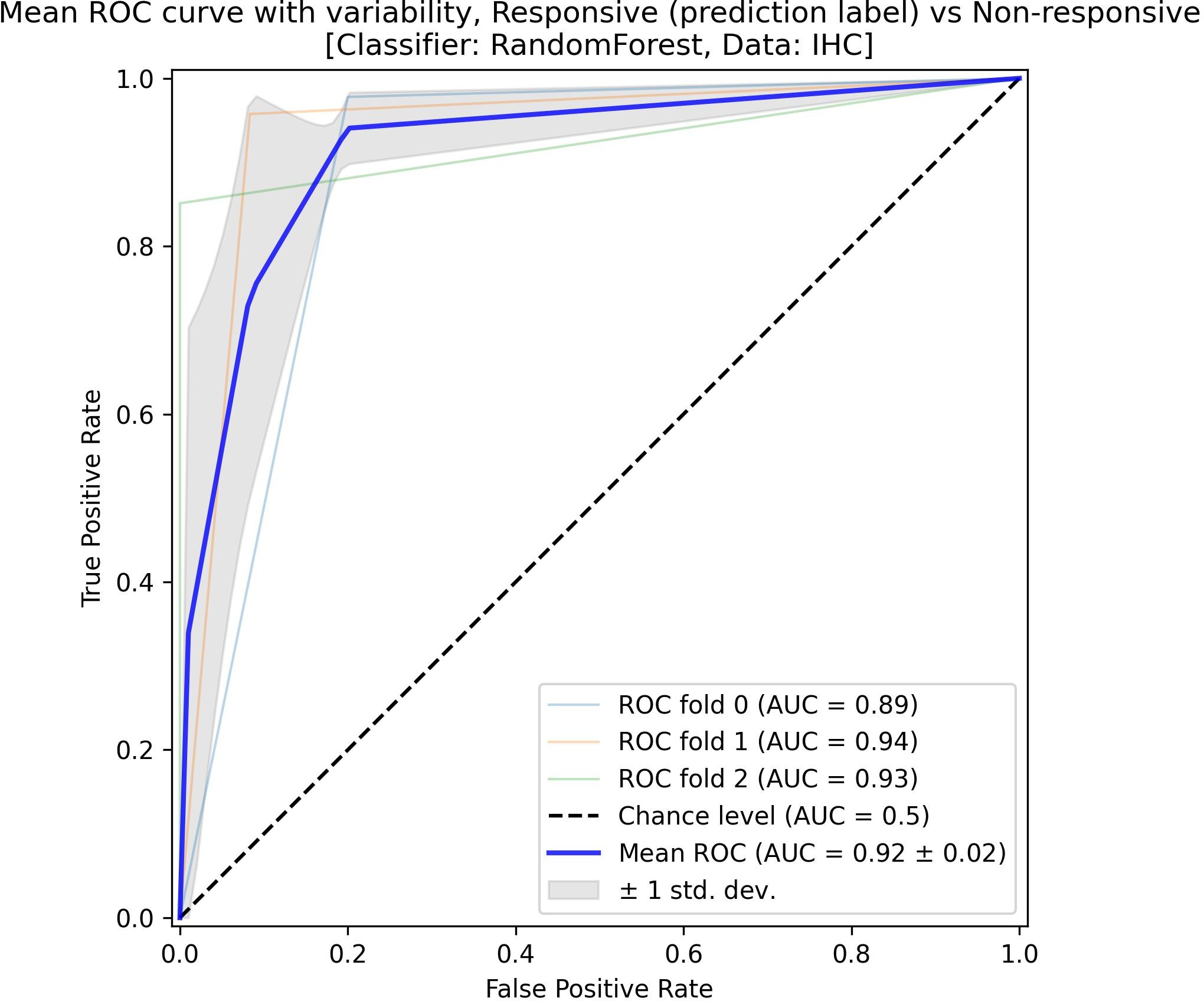


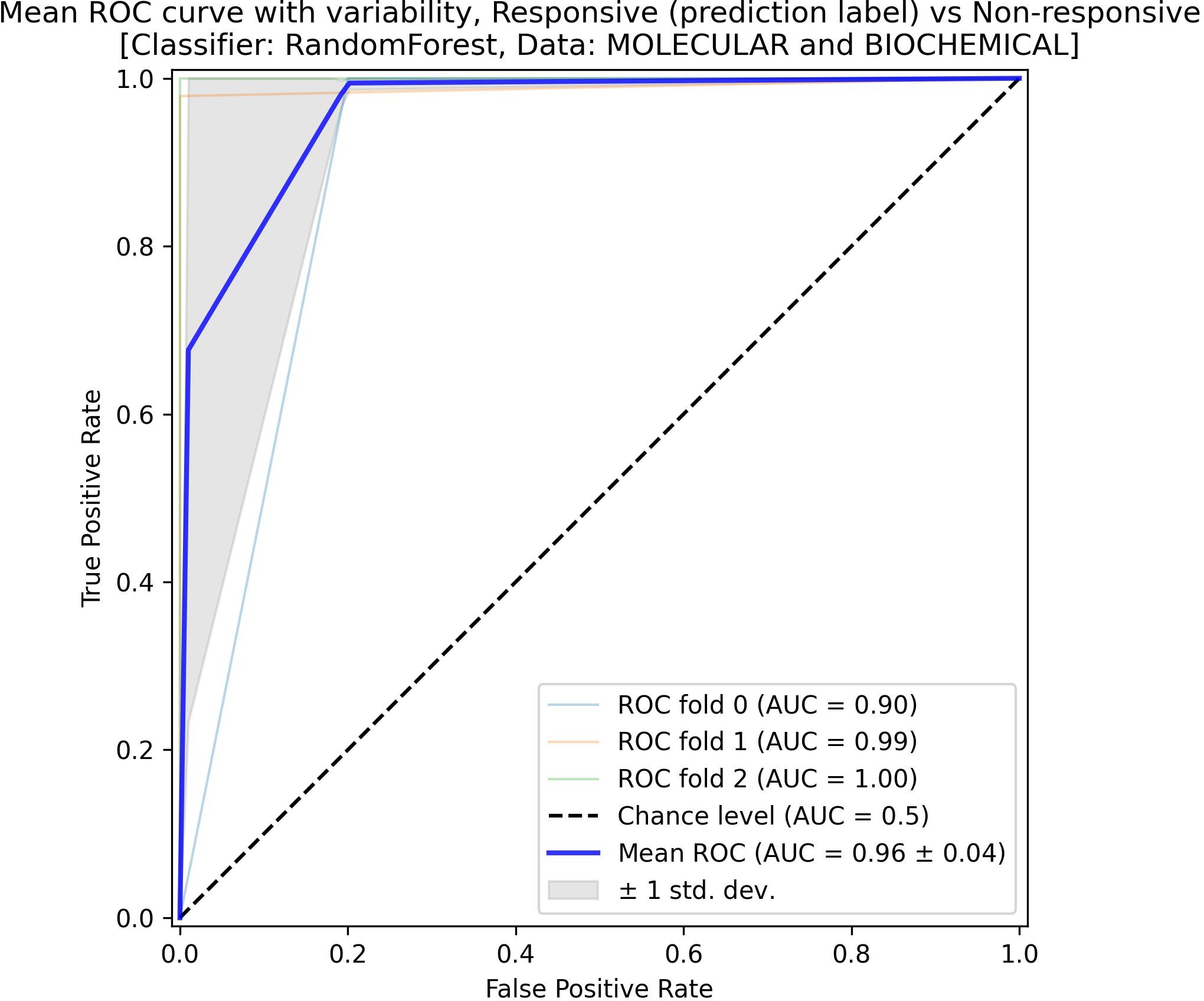


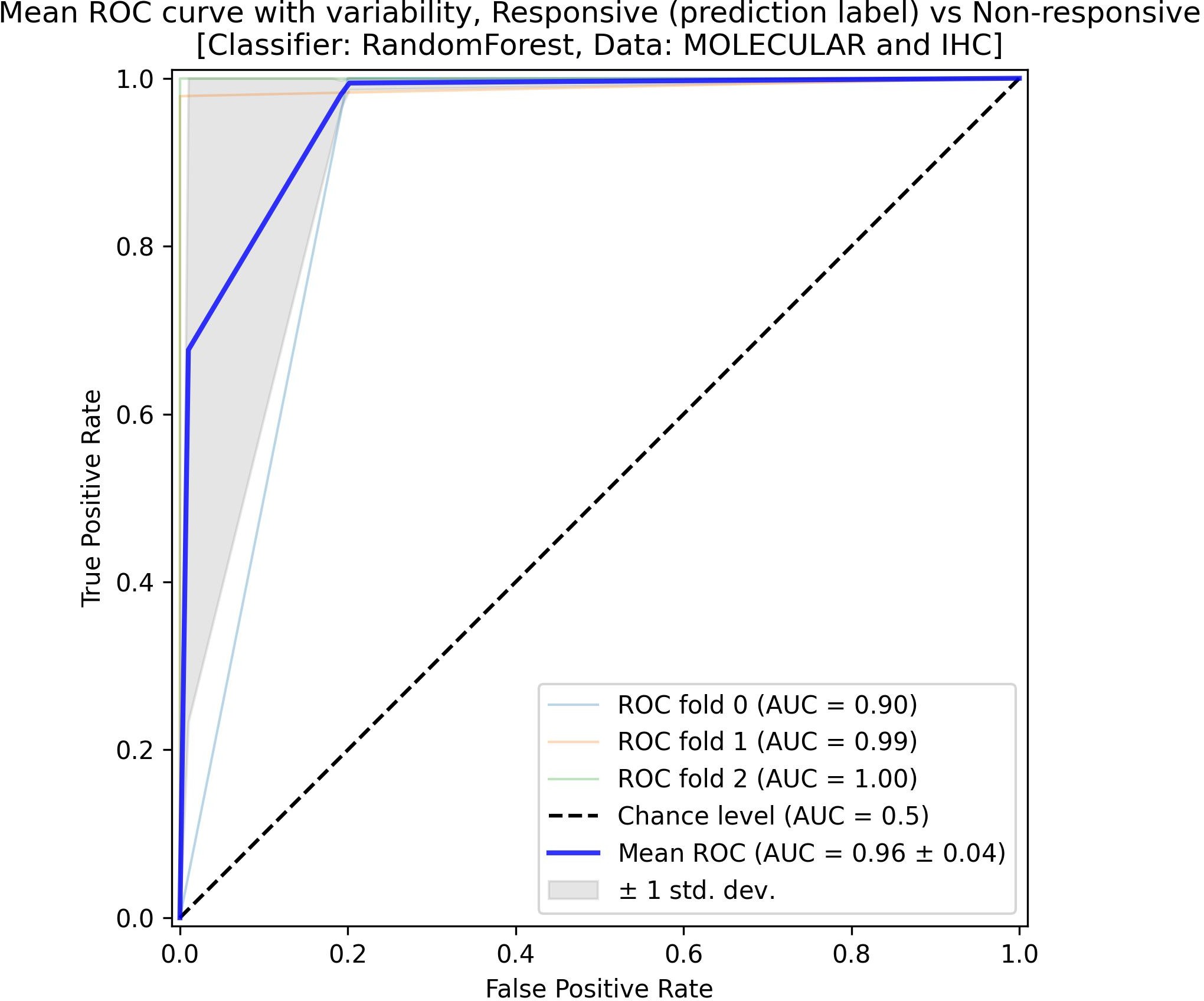


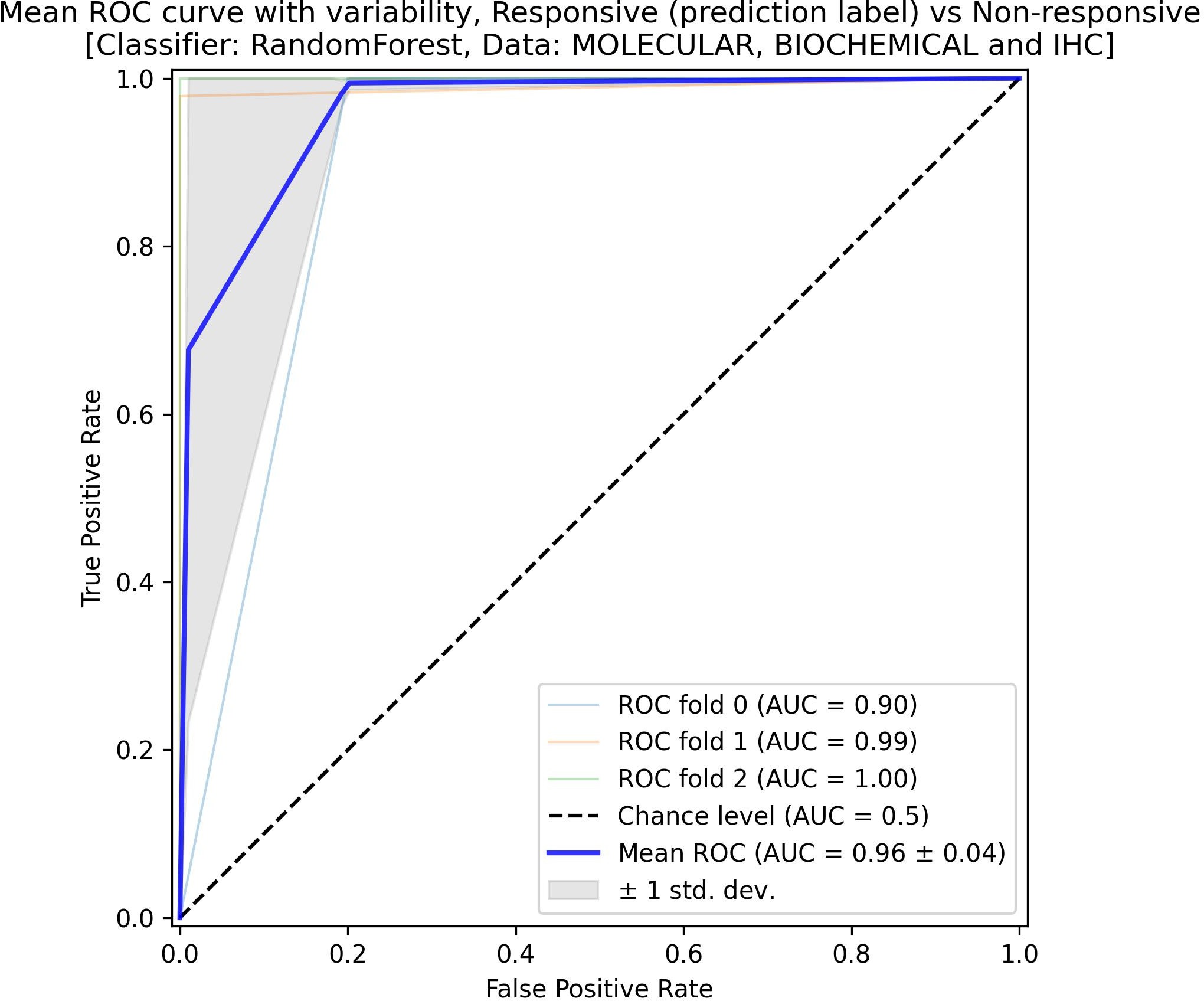


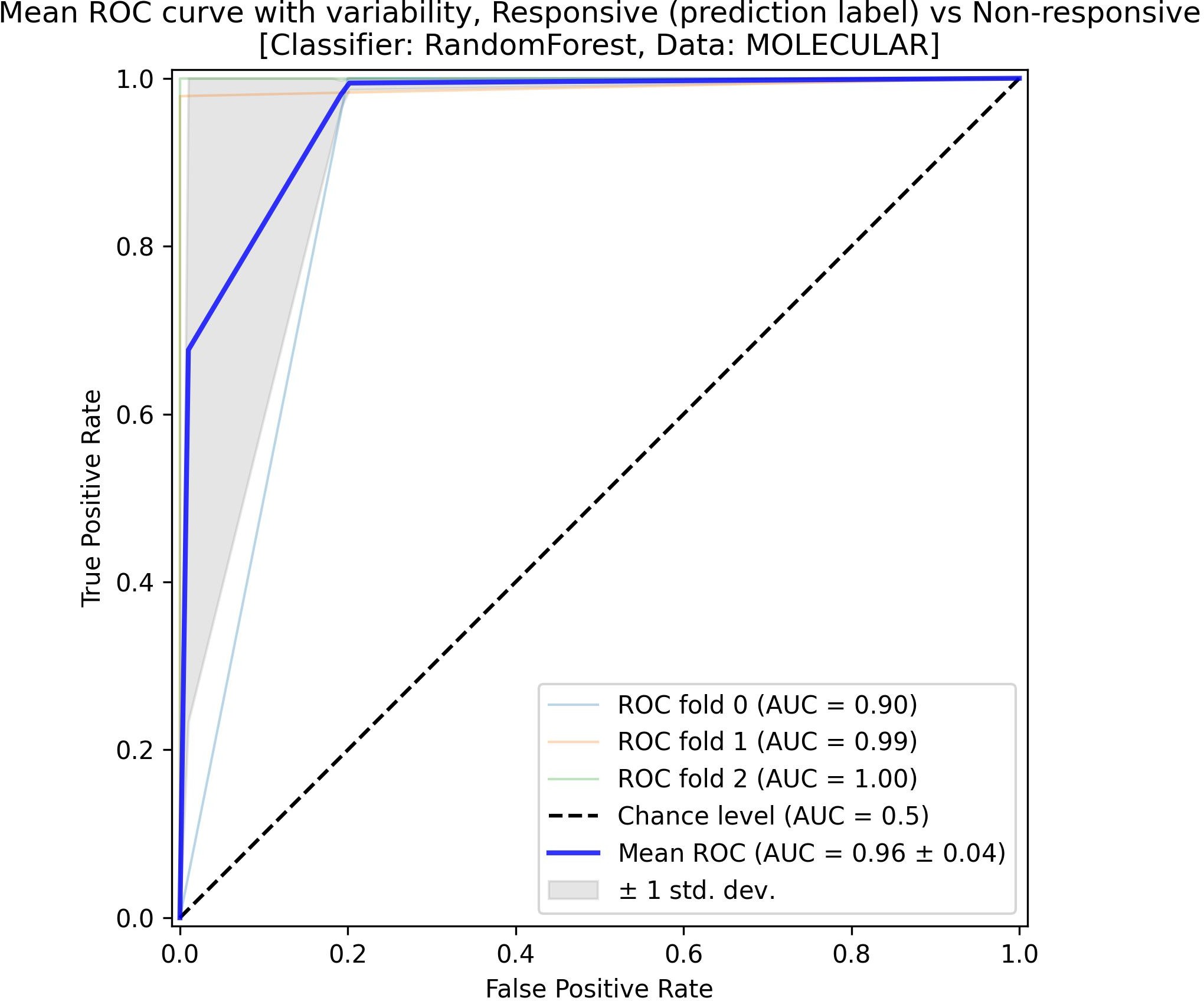


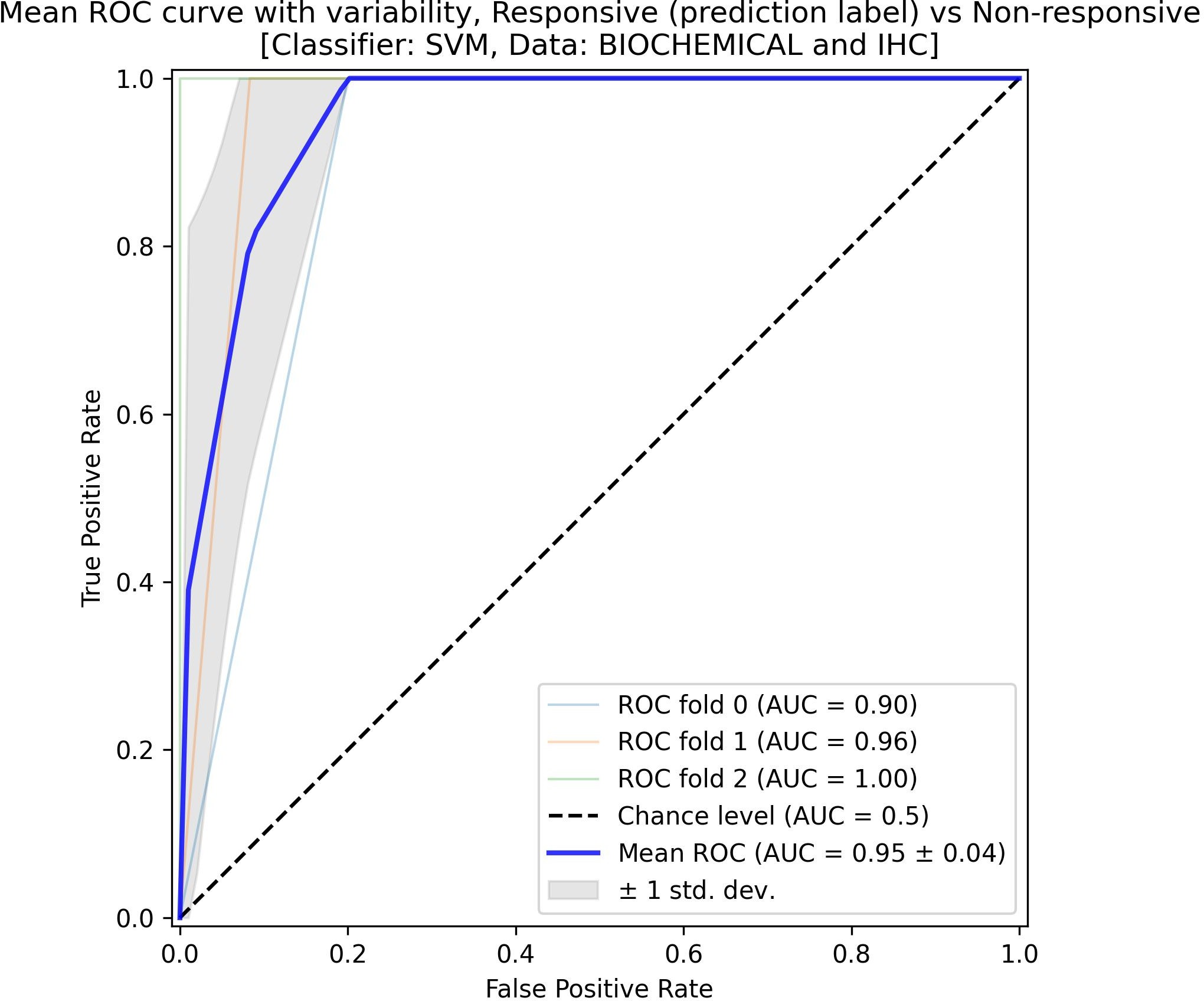


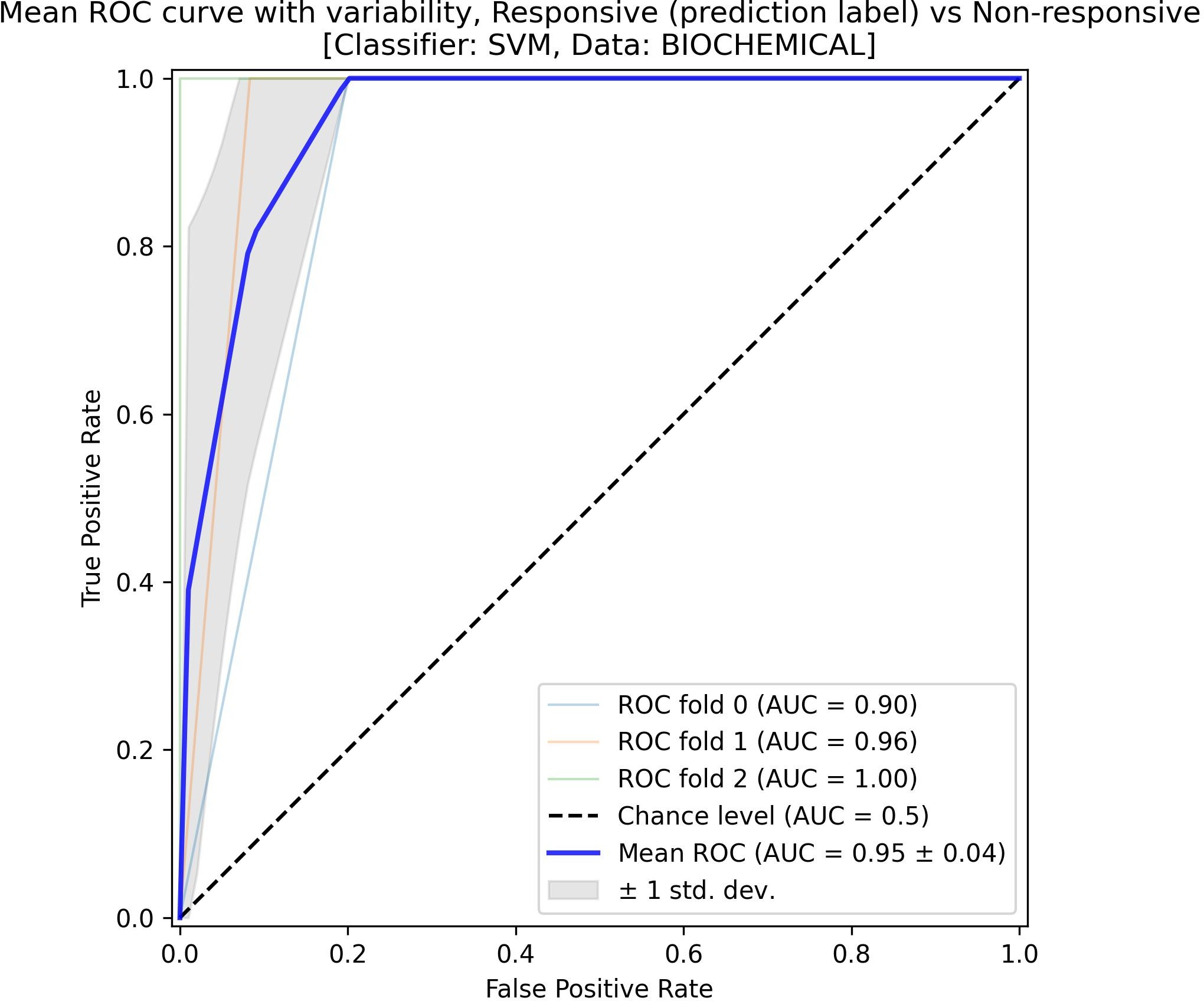


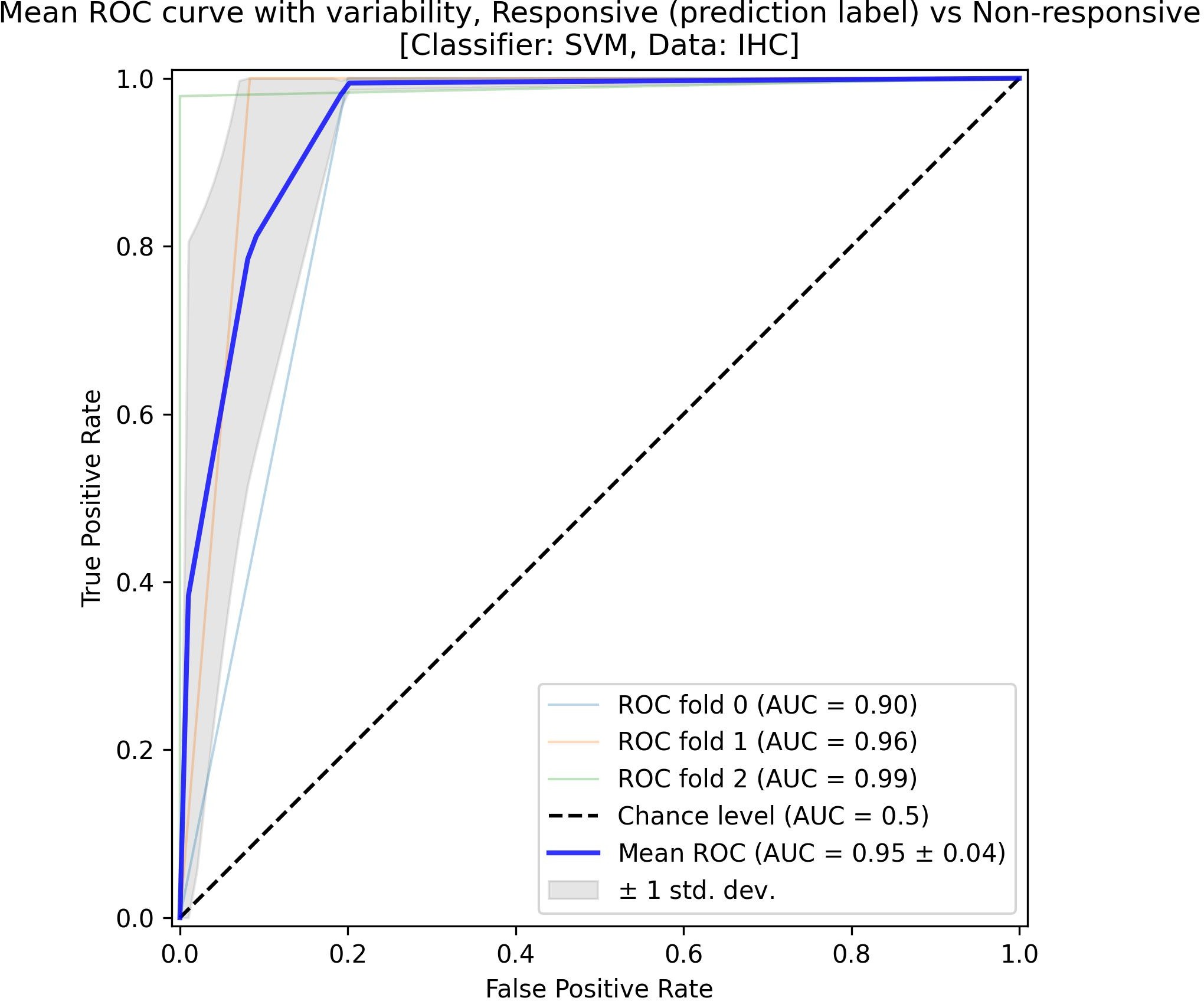


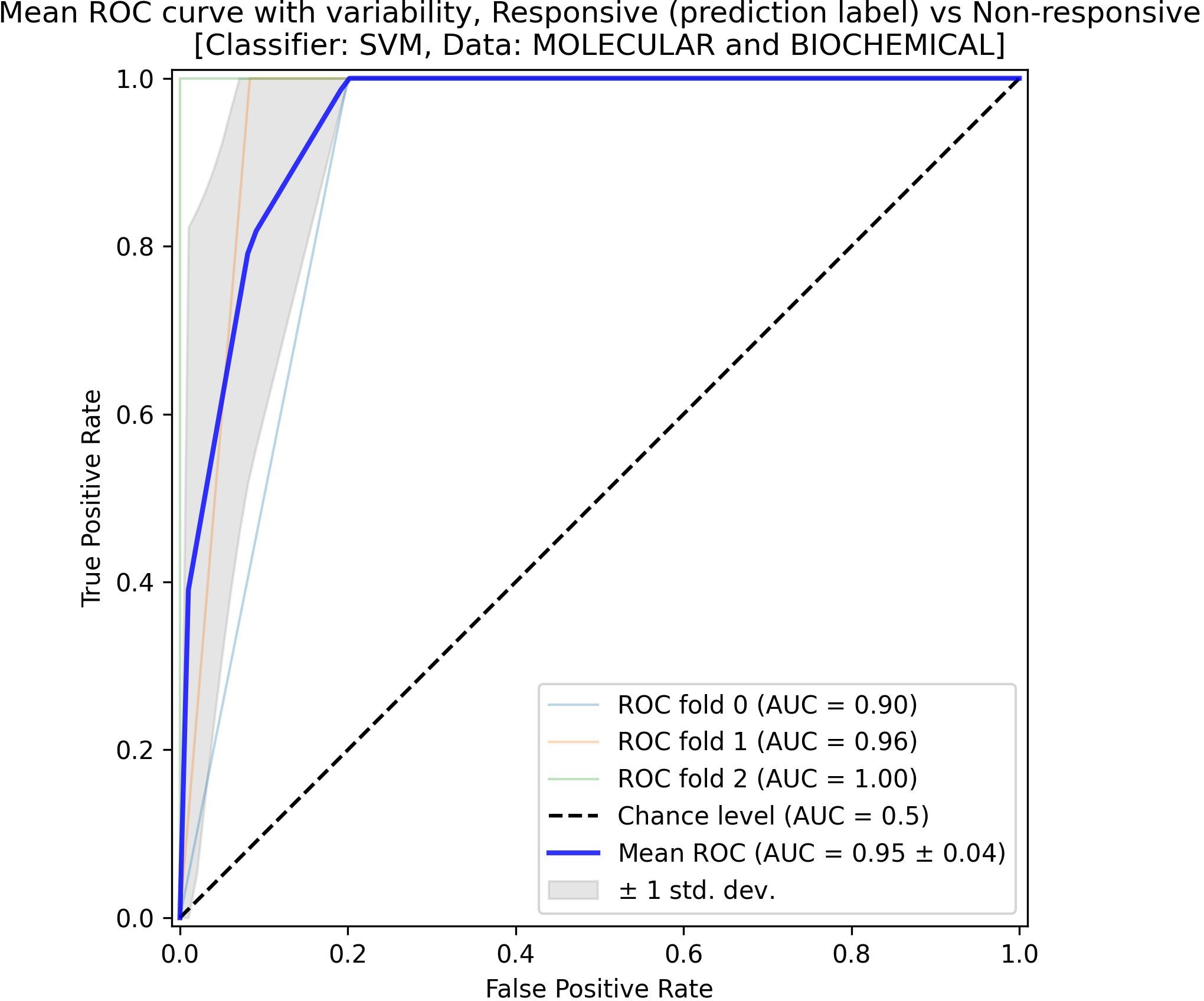


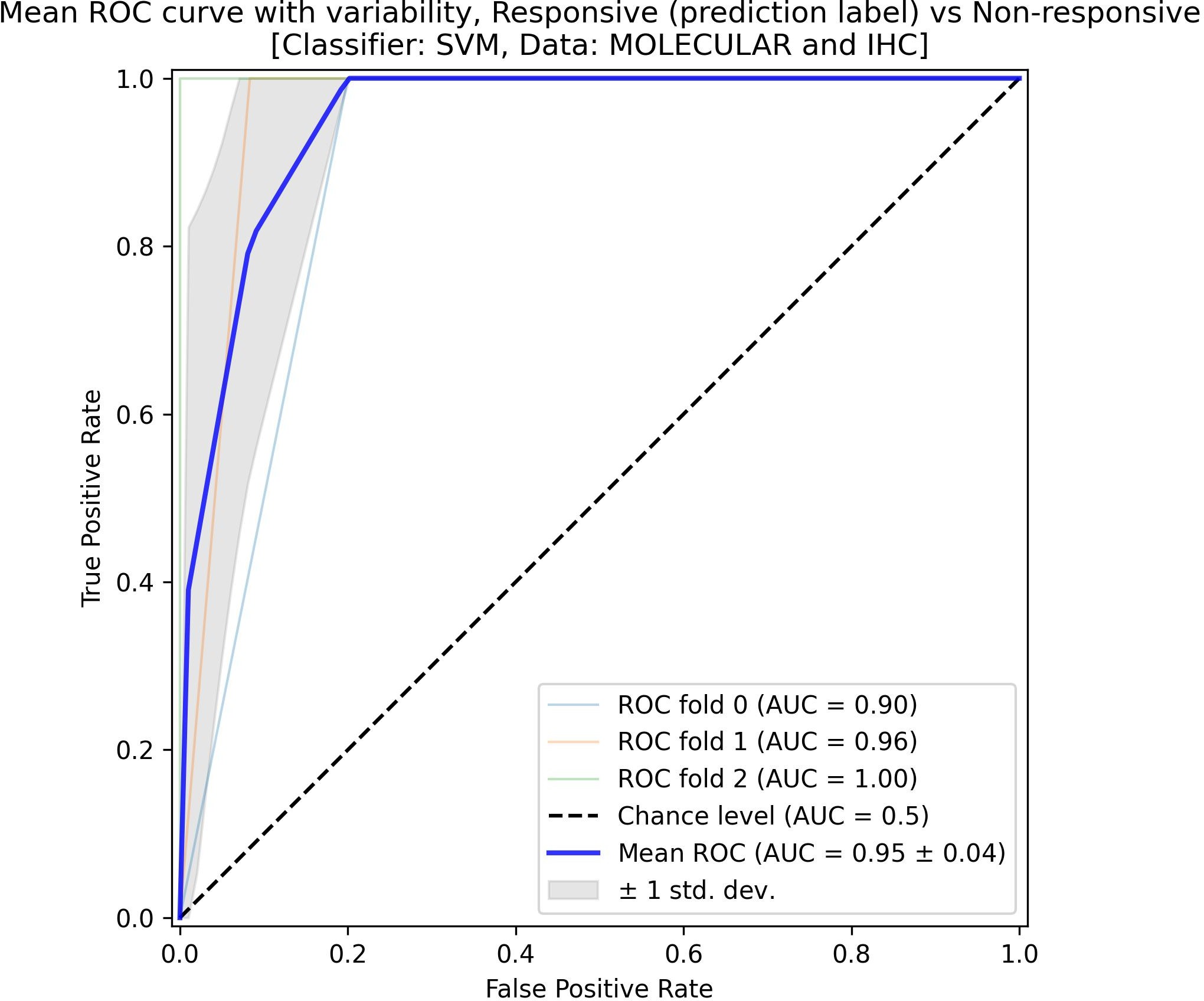


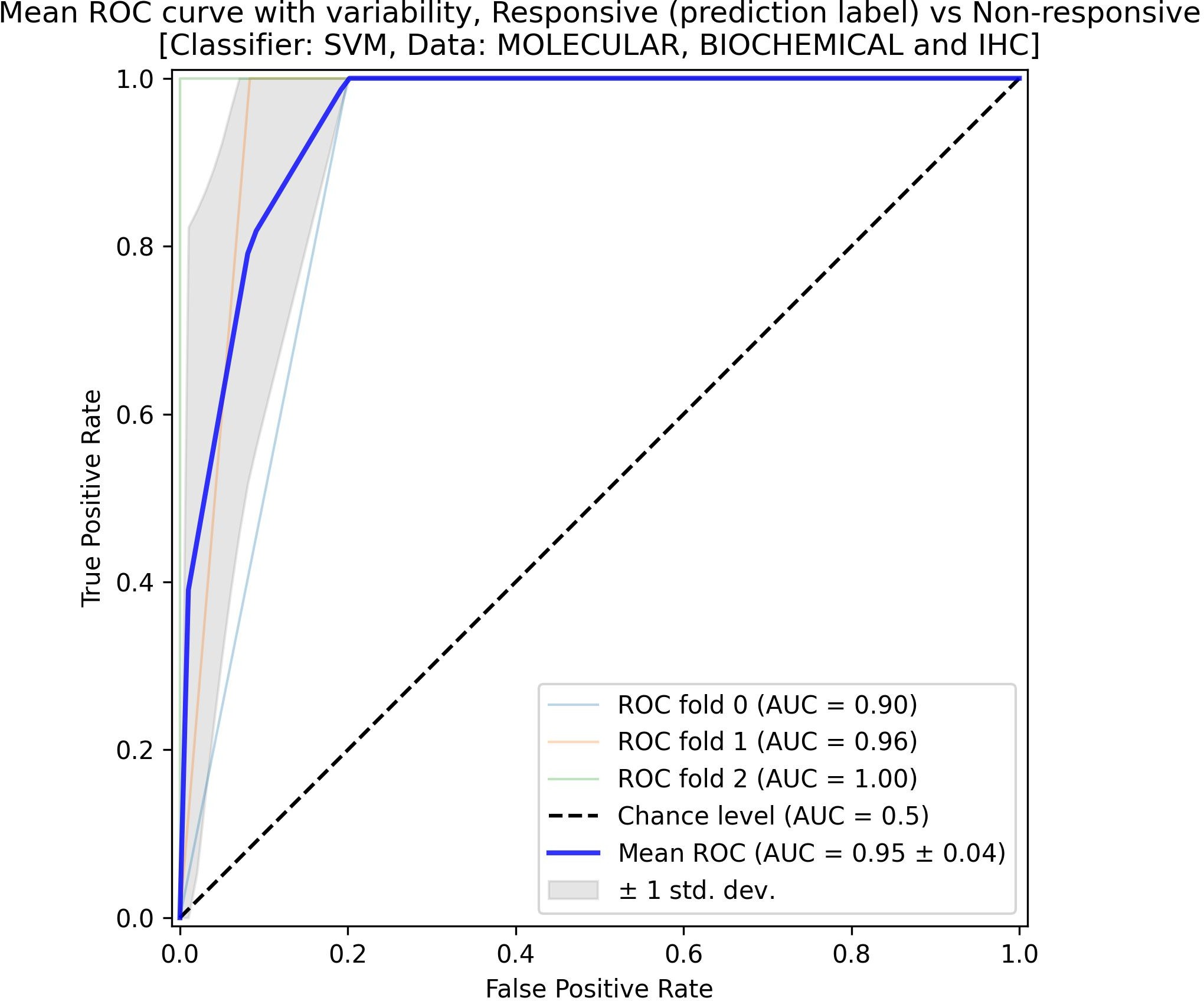


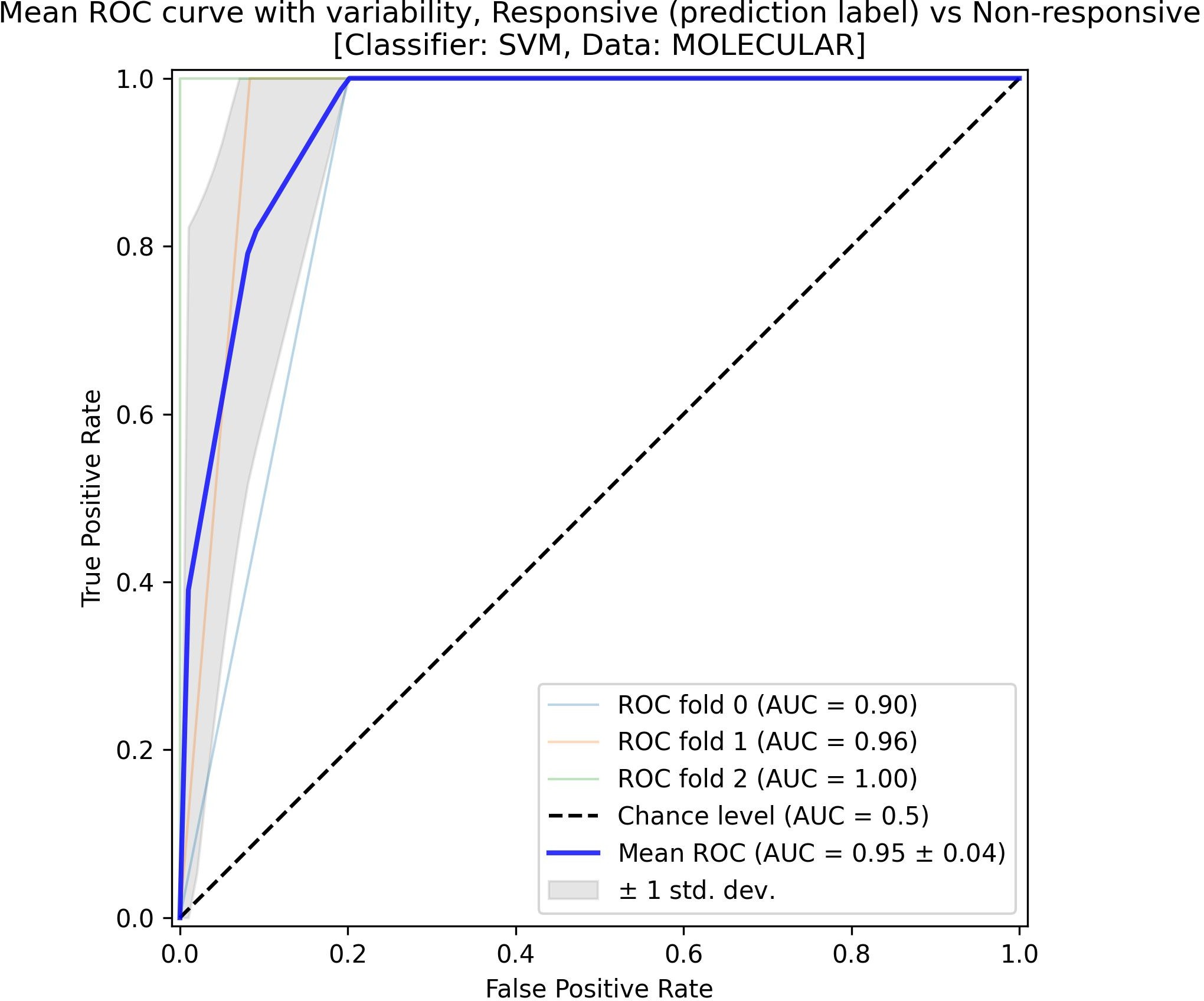

Supplement: Supplementary file 1 [file DataSheet1.zip › Supplementary File 1.DOCX]
