# Supplementary material for: Integrating molecular, biochemical, and immunohistochemical features as predictors of hepatocellular carcinoma drug response using machine-learning algorithms
Source: Front Mol Biosci. 2024 Oct 16;11:1430794. doi: 10.3389/fmolb.2024.1430794 (PMC11521808; doi:10.3389/fmolb.2024.1430794)
Supplement: Supplementary file 1 [file DataSheet1.zip › Supplementary File 12.PDF]

GSTP:
Hesperidin-50: 4.156922, 0.000032, 0.000032.
Hesperidin-100: 4.156922, 0.000032, 0.000032.
Hesperidin-200: 4.156922, 0.000032, 0.000032.
Cyan-10: 4.156922, 0.000032, 0.000032.
Cyan-20: 4.156922, 0.000032, 0.000032.
Cyan-30: 4.156922, 0.000032, 0.000032.
Pentoperazole-25: 4.156922, 0.000032, 0.000032.
Pentoperazole-50: 4.156922, 0.000032, 0.000032.
Pentoperazole-100: 4.156922, 0.000032, 0.000032.

PCNA:
Hesperidin-50: 3.348632, 0.000812, 0.000812.
Hesperidin-100: 4.156922, 0.000032, 0.000036.
Hesperidin-200: 4.156922, 0.000032, 0.000036.
Cyan-10: 4.156922, 0.000032, 0.000036.
Cyan-20: 4.156922, 0.000032, 0.000036.
Cyan-30: 4.156922, 0.000032, 0.000036.
Pentoperazole-25: 4.156922, 0.000032, 0.000036.
Pentoperazole-50: 4.156922, 0.000032, 0.000036.
Pentoperazole-100: 4.156922, 0.000032, 0.000036.

TNF:
Hesperidin-50: 3.262029, 0.001106, 0.001106.
Hesperidin-100: 4.156922, 0.000032, 0.000036.
Hesperidin-200: 4.156922, 0.000032, 0.000036.
Cyan-10: 4.156922, 0.000032, 0.000036.
Cyan-20: 4.156922, 0.000032, 0.000036.
Cyan-30: 4.156922, 0.000032, 0.000036.
Pentoperazole-25: 4.156922, 0.000032, 0.000036.
Pentoperazole-50: 4.156922, 0.000032, 0.000036.
Pentoperazole-100: 4.156922, 0.000032, 0.000036.
