# Supplementary material for: Integrating molecular, biochemical, and immunohistochemical features as predictors of hepatocellular carcinoma drug response using machine-learning algorithms
Source: Front Mol Biosci. 2024 Oct 16;11:1430794. doi: 10.3389/fmolb.2024.1430794 (PMC11521808; doi:10.3389/fmolb.2024.1430794)
Supplement: Supplementary file 1 [file DataSheet1.zip › Supplementary File 11.PDF]

GSTP:
Hesperidin-50: -3.956283, 0.000076, 0.000098.
Hesperidin-100: -3.956283, 0.000076, 0.000098.
Hesperidin-200: -3.956283, 0.000076, 0.000098.
Cyan-10: -3.956283, 0.000076, 0.000098.
Cyan-20: -3.956283, 0.000076, 0.000098.
Cyan-30: -3.758469, 0.000171, 0.000192.
Pentoperazole-25: -3.956283, 0.000076, 0.000098.
Pentoperazole-50: -3.956283, 0.000076, 0.000098.
Pentoperazole-100: -2.670491, 0.007574, 0.007574.

PCNA:
Hesperidin-50: -3.956283, 0.000076, 0.000086.
Hesperidin-100: -3.956283, 0.000076, 0.000086.
Hesperidin-200: -3.956283, 0.000076, 0.000086.
Cyan-10: -3.956283, 0.000076, 0.000086.
Cyan-20: -3.956283, 0.000076, 0.000086.
Cyan-30: -3.956283, 0.000076, 0.000086.
Pentoperazole-25: -3.956283, 0.000076, 0.000086.
Pentoperazole-50: -3.956283, 0.000076, 0.000086.
Pentoperazole-100: -1.978141, 0.047913, 0.047913.

TNF:
Hesperidin-50: -3.956283, 0.000076, 0.000171.
Hesperidin-100: -3.956283, 0.000076, 0.000171.
Hesperidin-200: -2.175956, 0.029559, 0.033253.
Cyan-10: -2.835336, 0.004578, 0.006867.
Cyan-20: -3.296902, 0.000978, 0.001760.
Cyan-30: -3.956283, 0.000076, 0.000171.
Pentoperazole-25: -2.175956, 0.029559, 0.033253.
Pentoperazole-50: -3.956283, 0.000076, 0.000171.
Pentoperazole-100: -1.681420, 0.092681, 0.092681.
