# Supplementary material for: Integrating molecular, biochemical, and immunohistochemical features as predictors of hepatocellular carcinoma drug response using machine-learning algorithms
Source: Front Mol Biosci. 2024 Oct 16;11:1430794. doi: 10.3389/fmolb.2024.1430794 (PMC11521808; doi:10.3389/fmolb.2024.1430794)
Supplement: Supplementary file 1 [file DataSheet1.zip › Supplementary File 10.PDF]

ALT:
Hesperidin-50: 4.156922, 0.000032, 0.000032.
Hesperidin-100: 4.156922, 0.000032, 0.000032.
Hesperidin-200: 4.156922, 0.000032, 0.000032.
Cyan-10: 4.156922, 0.000032, 0.000032.
Cyan-20: 4.156922, 0.000032, 0.000032.
Cyan-30: 4.156922, 0.000032, 0.000032.
Pentoperazole-25: 4.156922, 0.000032, 0.000032.
Pentoperazole-50: 4.156922, 0.000032, 0.000032.
Pentoperazole-100: 4.156922, 0.000032, 0.000032.

AST:
Hesperidin-50: 3.319764, 0.000901, 0.001158.
Hesperidin-100: 4.156922, 0.000032, 0.000048.
Hesperidin-200: 4.156922, 0.000032, 0.000048.
Cyan-10: 1.847521, 0.064672, 0.072756.
Cyan-20: 4.156922, 0.000032, 0.000048.
Cyan-30: 4.156922, 0.000032, 0.000048.
Pentoperazole-25: 1.645448, 0.099877, 0.099877.
Pentoperazole-50: 4.156922, 0.000032, 0.000048.
Pentoperazole-100: 4.156922, 0.000032, 0.000048.

ALP:
Hesperidin-50: 3.550704, 0.000384, 0.000494.
Hesperidin-100: 4.156922, 0.000032, 0.000048.
Hesperidin-200: 4.156922, 0.000032, 0.000048.
Cyan-10: 2.598076, 0.009375, 0.010547.
Cyan-20: 4.156922, 0.000032, 0.000048.
Cyan-30: 4.156922, 0.000032, 0.000048.
Pentoperazole-25: 2.424871, 0.015314, 0.015314.
Pentoperazole-50: 4.156922, 0.000032, 0.000048.
Pentoperazole-100: 4.156922, 0.000032, 0.000048.

GGT:
Hesperidin-50: 3.868247, 0.000110, 0.000141.
Hesperidin-100: 4.156922, 0.000032, 0.000048.
Hesperidin-200: 4.156922, 0.000032, 0.000048.
Cyan-10: 3.406367, 0.000658, 0.000741.
Cyan-20: 4.156922, 0.000032, 0.000048.
Cyan-30: 4.156922, 0.000032, 0.000048.
Pentoperazole-25: 3.059956, 0.002214, 0.002214.
Pentoperazole-50: 4.156922, 0.000032, 0.000048.
Pentoperazole-100: 4.156922, 0.000032, 0.000048.

T.Bilirubin:
Hesperidin-50: 3.233162, 0.001224, 0.001574.
Hesperidin-100: 4.156922, 0.000032, 0.000048.
Hesperidin-200: 4.156922, 0.000032, 0.000048.
Cyan-10: 2.742414, 0.006099, 0.006861.
Cyan-20: 4.156922, 0.000032, 0.000048.
Cyan-30: 4.156922, 0.000032, 0.000048.
Pentoperazole-25: 2.626944, 0.008616, 0.008616.
Pentoperazole-50: 4.156922, 0.000032, 0.000048.
Pentoperazole-100: 4.156922, 0.000032, 0.000048.

D.Bilirubin:
Hesperidin-50: 3.695042, 0.000220, 0.000247.
Hesperidin-100: 4.156922, 0.000032, 0.000048.
Hesperidin-200: 4.156922, 0.000032, 0.000048.
Cyan-10: 3.781644, 0.000156, 0.000200.
Cyan-20: 4.156922, 0.000032, 0.000048.
Cyan-30: 4.156922, 0.000032, 0.000048.
Pentoperazole-25: 3.319764, 0.000901, 0.000901.
Pentoperazole-50: 4.156922, 0.000032, 0.000048.
Pentoperazole-100: 4.156922, 0.000032, 0.000048.

AFP:
Hesperidin-50: 4.041452, 0.000053, 0.000068.
Hesperidin-100: 4.156922, 0.000032, 0.000048.
Hesperidin-200: 4.156922, 0.000032, 0.000048.
Cyan-10: 3.810512, 0.000139, 0.000139.
Cyan-20: 4.156922, 0.000032, 0.000048.
Cyan-30: 4.156922, 0.000032, 0.000048.
Pentoperazole-25: 3.810512, 0.000139, 0.000139.
Pentoperazole-50: 4.156922, 0.000032, 0.000048.
Pentoperazole-100: 4.156922, 0.000032, 0.000048.

Albumin:
Hesperidin-50: -4.156922, 0.000032, 0.000032.
Hesperidin-100: -4.156922, 0.000032, 0.000032.
Hesperidin-200: -4.156922, 0.000032, 0.000032.
Cyan-10: -4.156922, 0.000032, 0.000032.
Cyan-20: -4.156922, 0.000032, 0.000032.
Cyan-30: -4.156922, 0.000032, 0.000032.
Pentoperazole-25: -4.156922, 0.000032, 0.000032.
Pentoperazole-50: -4.156922, 0.000032, 0.000032.
Pentoperazole-100: -4.156922, 0.000032, 0.000032.

TC:
Hesperidin-50: 3.637307, 0.000276, 0.000354.
Hesperidin-100: 4.156922, 0.000032, 0.000048.
Hesperidin-200: 4.156922, 0.000032, 0.000048.
Cyan-10: 3.002221, 0.002680, 0.002680.
Cyan-20: 4.156922, 0.000032, 0.000048.
Cyan-30: 4.156922, 0.000032, 0.000048.
Pentoperazole-25: 3.002221, 0.002680, 0.002680.
Pentoperazole-50: 4.156922, 0.000032, 0.000048.
Pentoperazole-100: 4.156922, 0.000032, 0.000048.

TG:
Hesperidin-50: 3.117691, 0.001823, 0.002344.
Hesperidin-100: 4.156922, 0.000032, 0.000048.
Hesperidin-200: 4.156922, 0.000032, 0.000048.
Cyan-10: 2.540341, 0.011074, 0.012459.
Cyan-20: 4.156922, 0.000032, 0.000048.
Cyan-30: 4.156922, 0.000032, 0.000048.
Pentoperazole-25: 2.424871, 0.015314, 0.015314.
Pentoperazole-50: 4.156922, 0.000032, 0.000048.
Pentoperazole-100: 4.156922, 0.000032, 0.000048.

HDL-C:
Hesperidin-50: -4.012584, 0.000060, 0.000180.
Hesperidin-100: -3.088824, 0.002010, 0.003014.
Hesperidin-200: -0.635085, 0.525373, 0.525373.
Cyan-10: -4.156922, 0.000032, 0.000145.
Cyan-20: -3.637307, 0.000276, 0.000496.
Cyan-30: -2.020726, 0.043308, 0.048722.
Pentoperazole-25: -4.156922, 0.000032, 0.000145.
Pentoperazole-50: -3.868247, 0.000110, 0.000247.
Pentoperazole-100: -2.078461, 0.037667, 0.048429.

LDL-C:
Hesperidin-50: 0.057735, 0.953960, 0.953960.
Hesperidin-100: 3.695042, 0.000220, 0.000396.
Hesperidin-200: 4.156922, 0.000032, 0.000097.
Cyan-10: -0.346410, 0.729034, 0.820164.
Cyan-20: 3.695042, 0.000220, 0.000396.
Cyan-30: 4.156922, 0.000032, 0.000097.
Pentoperazole-25: -0.404145, 0.686106, 0.820164.
Pentoperazole-50: 3.637307, 0.000276, 0.000413.
Pentoperazole-100: 4.156922, 0.000032, 0.000097.
