# Supplementary material for: Integrating molecular, biochemical, and immunohistochemical features as predictors of hepatocellular carcinoma drug response using machine-learning algorithms
Source: Front Mol Biosci. 2024 Oct 16;11:1430794. doi: 10.3389/fmolb.2024.1430794 (PMC11521808; doi:10.3389/fmolb.2024.1430794)
Supplement: Supplementary file 1 [file DataSheet1.zip › Supplementary File 7.PDF]

lncRNA-RP11-513I15.6:
Hesperidin-50: -3.956283, 0.000076, 0.000685.
Hesperidin-100: -3.165026, 0.001551, 0.003489.
Hesperidin-200: 1.252823, 0.210270, 0.270347.
Cyan-10: -1.120947, 0.262311, 0.295099.
Cyan-20: 1.384699, 0.166145, 0.249217.
cyan -30: 2.373770, 0.017608, 0.031694.
Pentoperazole-25: -3.560655, 0.000370, 0.001665.
pentoperazole-50: -3.428778, 0.000606, 0.001819.
Pentoperazole-100: 0.989071, 0.322629, 0.322629.

miR-125b:
Hesperidin-50: -1.945172, 0.051754, 0.051754.
Hesperidin-100: -2.835336, 0.004578, 0.005886.
Hesperidin-200: -3.890345, 0.000100, 0.000300.
Cyan-10: -3.395809, 0.000684, 0.001232.
Cyan-20: -3.956283, 0.000076, 0.000300.
cyan -30: -3.956283, 0.000076, 0.000300.
Pentoperazole-25: -1.945172, 0.051754, 0.051754.
pentoperazole-50: -2.835336, 0.004578, 0.005886.
Pentoperazole-100: -3.758469, 0.000171, 0.000385.

miR-1289:
Hesperidin-50: -2.835336, 0.004578, 0.004578.
Hesperidin-100: -3.560655, 0.000370, 0.000555.
Hesperidin-200: -3.956283, 0.000076, 0.000137.
Cyan-10: -3.956283, 0.000076, 0.000137.
Cyan-20: -3.956283, 0.000076, 0.000137.
cyan -30: -3.956283, 0.000076, 0.000137.
Pentoperazole-25: -2.835336, 0.004578, 0.004578.
pentoperazole-50: -3.428778, 0.000606, 0.000780.
Pentoperazole-100: -3.956283, 0.000076, 0.000137.

lncRNA-RP11-583F2.2:
Hesperidin-50: -2.901274, 0.003716, 0.005575.
Hesperidin-100: -1.252823, 0.210270, 0.236554.
Hesperidin-200: 3.692531, 0.000222, 0.000862.
Cyan-10: 3.165026, 0.001551, 0.003489.
Cyan-20: 3.626593, 0.000287, 0.000862.
cyan -30: 3.956283, 0.000076, 0.000685.
Pentoperazole-25: -2.901274, 0.003716, 0.005575.
pentoperazole-50: -0.659380, 0.509651, 0.509651.
Pentoperazole-100: 2.637522, 0.008351, 0.010738.

miR-1262:
Hesperidin-50: -3.165026, 0.001551, 0.001551.
Hesperidin-100: -3.956283, 0.000076, 0.000169.
Hesperidin-200: -3.890345, 0.000100, 0.000169.
Cyan-10: -3.824407, 0.000131, 0.000169.
Cyan-20: -3.956283, 0.000076, 0.000169.
cyan -30: -3.956283, 0.000076, 0.000169.
Pentoperazole-25: -3.428778, 0.000606, 0.000682.
pentoperazole-50: -3.824407, 0.000131, 0.000169.
Pentoperazole-100: -3.956283, 0.000076, 0.000169.

BAX mRNA:
Hesperidin-50: 3.956283, 0.000076, 0.000076.
Hesperidin-100: -3.956283, 0.000076, 0.000076.
Hesperidin-200: -3.956283, 0.000076, 0.000076.
Cyan-10: -3.956283, 0.000076, 0.000076.
Cyan-20: -3.956283, 0.000076, 0.000076.
cyan -30: -3.956283, 0.000076, 0.000076.
Pentoperazole-25: -3.956283, 0.000076, 0.000076.
pentoperazole-50: -3.956283, 0.000076, 0.000076.
Pentoperazole-100: -3.956283, 0.000076, 0.000076.

Cyclin E mRNA:
Hesperidin-50: -3.956283, 0.000076, 0.000076.
Hesperidin-100: -3.956283, 0.000076, 0.000076.
Hesperidin-200: -3.956283, 0.000076, 0.000076.
Cyan-10: -3.956283, 0.000076, 0.000076.
Cyan-20: -3.956283, 0.000076, 0.000076.
cyan -30: -3.956283, 0.000076, 0.000076.
Pentoperazole-25: -3.956283, 0.000076, 0.000076.
pentoperazole-50: -3.956283, 0.000076, 0.000076.
Pentoperazole-100: -3.956283, 0.000076, 0.000076.

ATG16-L1:
Hesperidin-50: -3.626593, 0.000287, 0.000369.
Hesperidin-100: -3.956283, 0.000076, 0.000137.
Hesperidin-200: -3.956283, 0.000076, 0.000137.
Cyan-10: -3.296902, 0.000978, 0.001099.
Cyan-20: -3.956283, 0.000076, 0.000137.
cyan -30: -3.956283, 0.000076, 0.000137.
Pentoperazole-25: -3.263933, 0.001099, 0.001099.
pentoperazole-50: -3.890345, 0.000100, 0.000150.
Pentoperazole-100: -3.956283, 0.000076, 0.000137.

lncRNA-MALAT:
Hesperidin-50: -3.956283, 0.000076, 0.000137.
Hesperidin-100: -3.956283, 0.000076, 0.000137.
Hesperidin-200: -1.582513, 0.113532, 0.127724.
Cyan-10: -3.956283, 0.000076, 0.000137.
Cyan-20: -3.692531, 0.000222, 0.000333.
cyan -30: 0.065938, 0.947427, 0.947427.
Pentoperazole-25: -3.956283, 0.000076, 0.000137.
pentoperazole-50: -3.956283, 0.000076, 0.000137.
Pentoperazole-100: -2.703460, 0.006862, 0.008823.

P53 mRNA:
Hesperidin-50: -3.626593, 0.000287, 0.000287.
Hesperidin-100: -3.956283, 0.000076, 0.000098.
Hesperidin-200: -3.956283, 0.000076, 0.000098.
Cyan-10: -3.956283, 0.000076, 0.000098.
Cyan-20: -3.956283, 0.000076, 0.000098.
cyan -30: -3.956283, 0.000076, 0.000098.
Pentoperazole-25: -3.824407, 0.000131, 0.000147.
pentoperazole-50: -3.956283, 0.000076, 0.000098.
Pentoperazole-100: -3.956283, 0.000076, 0.000098.

RAB11 mRNA:
Hesperidin-50: -1.813296, 0.069786, 0.069786.
Hesperidin-100: -2.571584, 0.010123, 0.013016.
Hesperidin-200: -3.956283, 0.000076, 0.000171.
Cyan-10: -3.890345, 0.000100, 0.000180.
Cyan-20: -3.956283, 0.000076, 0.000171.
cyan -30: -3.956283, 0.000076, 0.000171.
Pentoperazole-25: -2.044079, 0.040946, 0.046064.
pentoperazole-50: -2.571584, 0.010123, 0.013016.
Pentoperazole-100: -3.956283, 0.000076, 0.000171.

miR-106b:
Hesperidin-50: -3.956283, 0.000076, 0.000171.
Hesperidin-100: -3.956283, 0.000076, 0.000171.
Hesperidin-200: -2.637522, 0.008351, 0.009395.
Cyan-10: -3.165026, 0.001551, 0.002791.
Cyan-20: -2.835336, 0.004578, 0.006867.
cyan -30: 0.989071, 0.322629, 0.322629.
Pentoperazole-25: -3.956283, 0.000076, 0.000171.
pentoperazole-50: -3.956283, 0.000076, 0.000171.
Pentoperazole-100: -2.769398, 0.005616, 0.007221.

circ_0001345:
Hesperidin-50: -3.956283, 0.000076, 0.000086.
Hesperidin-100: -3.956283, 0.000076, 0.000086.
Hesperidin-200: -3.956283, 0.000076, 0.000086.
Cyan-10: -3.956283, 0.000076, 0.000086.
Cyan-20: -3.956283, 0.000076, 0.000086.
cyan -30: -3.956283, 0.000076, 0.000086.
Pentoperazole-25: -3.956283, 0.000076, 0.000086.
pentoperazole-50: -3.890345, 0.000100, 0.000100.
Pentoperazole-100: -3.956283, 0.000076, 0.000086.

TUBG mRNA:
Hesperidin-50: -3.956283, 0.000076, 0.000114.
Hesperidin-100: -3.956283, 0.000076, 0.000114.
Hesperidin-200: -3.033150, 0.002420, 0.002723.
Cyan-10: -3.956283, 0.000076, 0.000114.
Cyan-20: -3.956283, 0.000076, 0.000114.
cyan -30: -1.846265, 0.064854, 0.064854.
Pentoperazole-25: -3.956283, 0.000076, 0.000114.
pentoperazole-50: -3.956283, 0.000076, 0.000114.
Pentoperazole-100: -3.165026, 0.001551, 0.001994.
