# Supplementary material for: Integrating molecular, biochemical, and immunohistochemical features as predictors of hepatocellular carcinoma drug response using machine-learning algorithms
Source: Front Mol Biosci. 2024 Oct 16;11:1430794. doi: 10.3389/fmolb.2024.1430794 (PMC11521808; doi:10.3389/fmolb.2024.1430794)
Supplement: Supplementary file 1 [file DataSheet1.zip › Supplementary File 9.PDF]

ALT:
Hesperidin-50: -3.956283, 0.000076, 0.000086.
Hesperidin-100: -3.956283, 0.000076, 0.000086.
Hesperidin-200: -3.956283, 0.000076, 0.000086.
Cyan-10: -3.956283, 0.000076, 0.000086.
Cyan-20: -3.956283, 0.000076, 0.000086.
Cyan-30: -3.956283, 0.000076, 0.000086.
Pentoperazole-25: -3.956283, 0.000076, 0.000086.
Pentoperazole-50: -3.956283, 0.000076, 0.000086.
Pentoperazole-100: -3.560655, 0.000370, 0.000370.

AST:
Hesperidin-50: -3.956283, 0.000076, 0.000086.
Hesperidin-100: -3.956283, 0.000076, 0.000086.
Hesperidin-200: -3.956283, 0.000076, 0.000086.
Cyan-10: -3.956283, 0.000076, 0.000086.
Cyan-20: -3.956283, 0.000076, 0.000086.
Cyan-30: -3.956283, 0.000076, 0.000086.
Pentoperazole-25: -3.956283, 0.000076, 0.000086.
Pentoperazole-50: -3.956283, 0.000076, 0.000086.
Pentoperazole-100: -1.780327, 0.075022, 0.075022.

ALP:
Hesperidin-50: -3.956283, 0.000076, 0.000098.
Hesperidin-100: -3.956283, 0.000076, 0.000098.
Hesperidin-200: -3.857376, 0.000115, 0.000129.
Cyan-10: -3.956283, 0.000076, 0.000098.
Cyan-20: -3.956283, 0.000076, 0.000098.
Cyan-30: -3.956283, 0.000076, 0.000098.
Pentoperazole-25: -3.956283, 0.000076, 0.000098.
Pentoperazole-50: -3.956283, 0.000076, 0.000098.
Pentoperazole-100: -0.197814, 0.843190, 0.843190.

GGT:
Hesperidin-50: -3.956283, 0.000076, 0.000076.
Hesperidin-100: -3.956283, 0.000076, 0.000076.
Hesperidin-200: -3.956283, 0.000076, 0.000076.
Cyan-10: -3.956283, 0.000076, 0.000076.
Cyan-20: -3.956283, 0.000076, 0.000076.
Cyan-30: -3.956283, 0.000076, 0.000076.
Pentoperazole-25: -3.956283, 0.000076, 0.000076.
Pentoperazole-50: -3.956283, 0.000076, 0.000076.
Pentoperazole-100: -3.956283, 0.000076, 0.000076.

T.Bilirubin:
Hesperidin-50: -3.956283, 0.000076, 0.000076.
Hesperidin-100: -3.956283, 0.000076, 0.000076.
Hesperidin-200: -3.956283, 0.000076, 0.000076.
Cyan-10: -3.956283, 0.000076, 0.000076.
Cyan-20: -3.956283, 0.000076, 0.000076.
Cyan-30: -3.956283, 0.000076, 0.000076.
Pentoperazole-25: -3.956283, 0.000076, 0.000076.
Pentoperazole-50: -3.956283, 0.000076, 0.000076.
Pentoperazole-100: -3.956283, 0.000076, 0.000076.

D.Bilirubin:
Hesperidin-50: -3.956283, 0.000076, 0.000076.
Hesperidin-100: -3.956283, 0.000076, 0.000076.
Hesperidin-200: -3.956283, 0.000076, 0.000076.
Cyan-10: -3.956283, 0.000076, 0.000076.
Cyan-20: -3.956283, 0.000076, 0.000076.
Cyan-30: -3.956283, 0.000076, 0.000076.
Pentoperazole-25: -3.956283, 0.000076, 0.000076.
Pentoperazole-50: -3.956283, 0.000076, 0.000076.
Pentoperazole-100: -3.956283, 0.000076, 0.000076.

AFP:
Hesperidin-50: -3.956283, 0.000076, 0.000076.
Hesperidin-100: -3.956283, 0.000076, 0.000076.
Hesperidin-200: -3.956283, 0.000076, 0.000076.
Cyan-10: -3.956283, 0.000076, 0.000076.
Cyan-20: -3.956283, 0.000076, 0.000076.
Cyan-30: -3.956283, 0.000076, 0.000076.
Pentoperazole-25: -3.956283, 0.000076, 0.000076.
Pentoperazole-50: -3.956283, 0.000076, 0.000076.
Pentoperazole-100: -3.956283, 0.000076, 0.000076.

Albumin:
Hesperidin-50: 3.956283, 0.000076, 0.000228.
Hesperidin-100: 3.824407, 0.000131, 0.000295.
Hesperidin-200: 0.065938, 0.947427, 0.947427.
Cyan-10: 3.956283, 0.000076, 0.000228.
Cyan-20: 3.692531, 0.000222, 0.000400.
Cyan-30: -1.384699, 0.166145, 0.186913.
Pentoperazole-25: 3.956283, 0.000076, 0.000228.
Pentoperazole-50: 3.560655, 0.000370, 0.000555.
Pentoperazole-100: -1.714389, 0.086457, 0.111159.

TC:
Hesperidin-50: -3.956283, 0.000076, 0.000228.
Hesperidin-100: -3.230964, 0.001234, 0.001851.
Hesperidin-200: -1.087978, 0.276605, 0.276605.
Cyan-10: -3.956283, 0.000076, 0.000228.
Cyan-20: -3.692531, 0.000222, 0.000400.
Cyan-30: -2.110018, 0.034857, 0.044816.
Pentoperazole-25: -3.956283, 0.000076, 0.000228.
Pentoperazole-50: -3.692531, 0.000222, 0.000400.
Pentoperazole-100: -1.714389, 0.086457, 0.097264.

TG:
Hesperidin-50: -3.956283, 0.000076, 0.000114.
Hesperidin-100: -3.956283, 0.000076, 0.000114.
Hesperidin-200: -0.989071, 0.322629, 0.414808.
Cyan-10: -3.956283, 0.000076, 0.000114.
Cyan-20: -3.956283, 0.000076, 0.000114.
Cyan-30: -0.791257, 0.428794, 0.428794.
Pentoperazole-25: -3.956283, 0.000076, 0.000114.
Pentoperazole-50: -3.956283, 0.000076, 0.000114.
Pentoperazole-100: -0.857195, 0.391337, 0.428794.

HDL-C:
Hesperidin-50: 1.978141, 0.047913, 0.061602.
Hesperidin-100: 3.428778, 0.000606, 0.001364.
Hesperidin-200: 3.956283, 0.000076, 0.000228.
Cyan-10: 1.186885, 0.235273, 0.264682.
Cyan-20: 3.296902, 0.000978, 0.001760.
Cyan-30: 3.956283, 0.000076, 0.000228.
Pentoperazole-25: 1.055009, 0.291421, 0.291421.
Pentoperazole-50: 2.637522, 0.008351, 0.012527.
Pentoperazole-100: 3.956283, 0.000076, 0.000228.

LDL-C:
Hesperidin-50: -3.956283, 0.000076, 0.000114.
Hesperidin-100: -3.956283, 0.000076, 0.000114.
Hesperidin-200: -2.835336, 0.004578, 0.005150.
Cyan-10: -3.956283, 0.000076, 0.000114.
Cyan-20: -3.956283, 0.000076, 0.000114.
Cyan-30: -3.033150, 0.002420, 0.003112.
Pentoperazole-25: -3.956283, 0.000076, 0.000114.
Pentoperazole-50: -3.956283, 0.000076, 0.000114.
Pentoperazole-100: -0.923133, 0.355938, 0.355938.
