# Supplementary material for: Integrating molecular, biochemical, and immunohistochemical features as predictors of hepatocellular carcinoma drug response using machine-learning algorithms
Source: Front Mol Biosci. 2024 Oct 16;11:1430794. doi: 10.3389/fmolb.2024.1430794 (PMC11521808; doi:10.3389/fmolb.2024.1430794)
Supplement: Supplementary file 1 [file DataSheet1.zip › Supplementary File 4.PDF]

MOLECULAR Data:
 Accuracy   : 1) 0.9500, 2) 0.9831, 3) 1.0000. Average: 0.9777, STD: 0.0208.
 Precision : 1) 0.9375, 2) 0.9792, 3) 1.0000. Average: 0.9722, STD: 0.0260.
 Recall   : 1) 1.0000, 2) 1.0000, 3) 1.0000. Average: 1.0000, STD: 0.0000.
 Specificity: 1) 0.8000, 2) 0.9167, 3) 1.0000. Average: 0.9056, STD: 0.0820.
 MCC   : 1) 0.8660, 2) 0.9474, 3) 1.0000. Average: 0.9378, STD: 0.0551.
 Reduced Model with SFS:
   Accuracy   : 1) 0.9833, 2) 0.9661, 3) 0.9661. Average: 0.9718, STD: 0.0081.
   Precision : 1) 0.9783, 2) 1.0000, 3) 1.0000. Average: 0.9928, STD: 0.0102.
   Recall   : 1) 1.0000, 2) 0.9574, 3) 0.9574. Average: 0.9716, STD: 0.0201.
   Specificity: 1) 0.9333, 2) 1.0000, 3) 1.0000. Average: 0.9778, STD: 0.0314.
   MCC   : 1) 0.9555, 2) 0.9059, 3) 0.9059. Average: 0.9224, STD: 0.0234.
   Features in: 2 features
     miR-125b
     TUBG mRNA
   Features out: 12 features
     lncRNA-RP11-513I15.6
     miR-1289
     lncRNA-RP11-583F2.2
     miR-1262
     BAX mRNA
     Cyclin E mRNA
     ATG16-L1
     lncRNA-MALAT
     P53 mRNA
     RAB11 mRNA
     miR-106b
     circ_0001345

BIOCHEMICAL Data:
 Accuracy   : 1) 0.9167, 2) 0.9322, 3) 0.9831. Average: 0.9440, STD: 0.0284.
 Precision : 1) 0.9348, 2) 0.9778, 3) 1.0000. Average: 0.9709, STD: 0.0271.
 Recall   : 1) 0.9556, 2) 0.9362, 3) 0.9787. Average: 0.9568, STD: 0.0174.
 Specificity: 1) 0.8000, 2) 0.9167, 3) 1.0000. Average: 0.9056, STD: 0.0820.
 MCC   : 1) 0.7735, 2) 0.8069, 3) 0.9505. Average: 0.8436, STD: 0.0768.
 Reduced Model with SFS:
   Accuracy   : 1) 0.9500, 2) 0.9831, 3) 0.9831. Average: 0.9720, STD: 0.0156.
   Precision : 1) 0.9375, 2) 0.9792, 3) 1.0000. Average: 0.9722, STD: 0.0260.
   Recall   : 1) 1.0000, 2) 1.0000, 3) 0.9787. Average: 0.9929, STD: 0.0100.
   Specificity: 1) 0.8000, 2) 0.9167, 3) 1.0000. Average: 0.9056, STD: 0.0820.
   MCC   : 1) 0.8660, 2) 0.9474, 3) 0.9505. Average: 0.9213, STD: 0.0391.
   Features in: 2 features
     ALT
     TG
   Features out: 10 features
     AST
     ALP
     GGT
     T.Bilirubin
     D.Bilirubin
     AFP
     Albumin
     TC
     HDL-C
     LDL-C

IHC Data:
Accuracy : 1) 0.9167, 2) 0.9661, 3) 0.9153. Average: 0.9327, STD: 0.0236.
Precision : 1) 0.9348, 2) 0.9787, 3) 1.0000. Average: 0.9712, STD: 0.0272.
Recall : 1) 0.9556, 2) 0.9787, 3) 0.8936. Average: 0.9426, STD: 0.0359.
Specificity: 1) 0.8000, 2) 0.9167, 3) 1.0000. Average: 0.9056, STD: 0.0820.
MCC : 1) 0.7735, 2) 0.8954, 3) 0.7942. Average: 0.8210, STD: 0.0532.
Reduced Model with SFS:
  Accuracy : 1) 0.9500, 2) 0.9661, 3) 0.9831. Average: 0.9664, STD: 0.0135.
  Precision : 1) 0.9375, 2) 0.9787, 3) 1.0000. Average: 0.9721, STD: 0.0259.
  Recall : 1) 1.0000, 2) 0.9787, 3) 0.9787. Average: 0.9858, STD: 0.0100.
  Specificity: 1) 0.8000, 2) 0.9167, 3) 1.0000. Average: 0.9056, STD: 0.0820.
  MCC : 1) 0.8660, 2) 0.8954, 3) 0.9505. Average: 0.9040, STD: 0.0350.
  Features in: 1 features
    GSTP
  Features out: 2 features
    PCNA
    TNF

MOLECULAR-BIOCHEMICAL Data:
Accuracy : 1) 0.9667, 2) 1.0000, 3) 0.9831. Average: 0.9832, STD: 0.0136.
Precision : 1) 0.9574, 2) 1.0000, 3) 1.0000. Average: 0.9858, STD: 0.0201.
Recall : 1) 1.0000, 2) 1.0000, 3) 0.9787. Average: 0.9929, STD: 0.0100.
Specificity: 1) 0.8667, 2) 1.0000, 3) 1.0000. Average: 0.9556, STD: 0.0629.
MCC : 1) 0.9109, 2) 1.0000, 3) 0.9505. Average: 0.9538, STD: 0.0364.
Reduced Model with SFS:
  Accuracy : 1) 0.9833, 2) 0.9661, 3) 0.9661. Average: 0.9718, STD: 0.0081.
  Precision : 1) 0.9783, 2) 1.0000, 3) 1.0000. Average: 0.9928, STD: 0.0102.
  Recall : 1) 1.0000, 2) 0.9574, 3) 0.9574. Average: 0.9716, STD: 0.0201.
  Specificity: 1) 0.9333, 2) 1.0000, 3) 1.0000. Average: 0.9778, STD: 0.0314.
  MCC : 1) 0.9555, 2) 0.9059, 3) 0.9059. Average: 0.9224, STD: 0.0234.
  Features in: 2 features
    miR-125b
    TUBG mRNA
  Features out: 24 features
    lncRNA-RP11-513I15.6
    miR-1289
    lncRNA-RP11-583F2.2
    miR-1262
    BAX mRNA
    Cyclin E mRNA
    ATG16-L1
    lncRNA-MALAT
    P53 mRNA
    RAB11 mRNA
    miR-106b
    circ_0001345
    ALT
    AST
    ALP
    GGT
    T.Bilirubin
    D.Bilirubin
    AFP
    Albumin

TC
TG
HDL-C
LDL-C

MOLECULAR-IHC Data:
Accuracy : 1) 0.9667, 2) 1.0000, 3) 0.9831. Average: 0.9832, STD: 0.0136.
Precision : 1) 0.9574, 2) 1.0000, 3) 1.0000. Average: 0.9858, STD: 0.0201.
Recall : 1) 1.0000, 2) 1.0000, 3) 0.9787. Average: 0.9929, STD: 0.0100.
Specificity: 1) 0.8667, 2) 1.0000, 3) 1.0000. Average: 0.9556, STD: 0.0629.
MCC : 1) 0.9109, 2) 1.0000, 3) 0.9505. Average: 0.9538, STD: 0.0364.
Reduced Model with SFS:
Accuracy : 1) 0.9833, 2) 0.9661, 3) 0.9661. Average: 0.9718, STD: 0.0081.
Precision : 1) 0.9783, 2) 1.0000, 3) 1.0000. Average: 0.9928, STD: 0.0102.
Recall : 1) 1.0000, 2) 0.9574, 3) 0.9574. Average: 0.9716, STD: 0.0201.
Specificity: 1) 0.9333, 2) 1.0000, 3) 1.0000. Average: 0.9778, STD: 0.0314.
MCC : 1) 0.9555, 2) 0.9059, 3) 0.9059. Average: 0.9224, STD: 0.0234.
Features in: 2 features
miR-125b
TUBG mRNA
Features out: 15 features
lncRNA-RP11-513I15.6
miR-1289
lncRNA-RP11-583F2.2
miR-1262
BAX mRNA
Cyclin E mRNA
ATG16-L1
lncRNA-MALAT
P53 mRNA
RAB11 mRNA
miR-106b
circ_0001345
GSTP
PCNA
TNF

BIOCHEMICAL-IHC Data:
Accuracy : 1) 0.9333, 2) 0.8814, 3) 0.8644. Average: 0.8930, STD: 0.0293.
Precision : 1) 0.9362, 2) 0.9167, 3) 0.8824. Average: 0.9117, STD: 0.0222.
Recall : 1) 0.9778, 2) 0.9362, 3) 0.9574. Average: 0.9571, STD: 0.0170.
Specificity: 1) 0.8000, 2) 0.6667, 3) 0.5000. Average: 0.6556, STD: 0.1227.
MCC : 1) 0.8175, 2) 0.6230, 3) 0.5378. Average: 0.6595, STD: 0.1170.
Reduced Model with SFS:
Accuracy : 1) 0.9500, 2) 0.9661, 3) 0.8814. Average: 0.9325, STD: 0.0367.
Precision : 1) 0.9375, 2) 0.9787, 3) 1.0000. Average: 0.9721, STD: 0.0259.
Recall : 1) 1.0000, 2) 0.9787, 3) 0.8511. Average: 0.9433, STD: 0.0658.
Specificity: 1) 0.8000, 2) 0.9167, 3) 1.0000. Average: 0.9056, STD: 0.0820.
MCC : 1) 0.8660, 2) 0.8954, 3) 0.7332. Average: 0.8315, STD: 0.0706.
Features in: 2 features
TG
GSTP
Features out: 13 features
ALT
AST

ALP
GGT
T.Bilirubin
D.Bilirubin
AFP
Albumin
TC
HDL-C
LDL-C
PCNA
TNF

MOLECULAR-BIOCHEMICAL-IHC Data:
Accuracy : 1) 0.9667, 2) 0.9831, 3) 0.9831. Average: 0.9776, STD: 0.0077.
Precision : 1) 0.9574, 2) 0.9792, 3) 1.0000. Average: 0.9789, STD: 0.0174.
Recall : 1) 1.0000, 2) 1.0000, 3) 0.9787. Average: 0.9929, STD: 0.0100.
Specificity: 1) 0.8667, 2) 0.9167, 3) 1.0000. Average: 0.9278, STD: 0.0550.
MCC : 1) 0.9109, 2) 0.9474, 3) 0.9505. Average: 0.9363, STD: 0.0180.
Reduced Model with SFS:
Accuracy : 1) 0.9833, 2) 0.9661, 3) 0.9661. Average: 0.9718, STD: 0.0081.
Precision : 1) 0.9783, 2) 1.0000, 3) 1.0000. Average: 0.9928, STD: 0.0102.
Recall : 1) 1.0000, 2) 0.9574, 3) 0.9574. Average: 0.9716, STD: 0.0201.
Specificity: 1) 0.9333, 2) 1.0000, 3) 1.0000. Average: 0.9778, STD: 0.0314.
MCC : 1) 0.9555, 2) 0.9059, 3) 0.9059. Average: 0.9224, STD: 0.0234.
Features in: 2 features
miR-125b
TUBG mRNA
Features out: 27 features
lncRNA-RP11-513I15.6
miR-1289
lncRNA-RP11-583F2.2
miR-1262
BAX mRNA
Cyclin E mRNA
ATG16-L1
lncRNA-MALAT
P53 mRNA
RAB11 mRNA
miR-106b
circ_0001345
ALT
AST
ALP
GGT
T.Bilirubin
D.Bilirubin
AFP
Albumin
TC
TG
HDL-C
LDL-C
GSTP
PCNA
TNF
