# Supplementary material for: Integrating molecular, biochemical, and immunohistochemical features as predictors of hepatocellular carcinoma drug response using machine-learning algorithms
Source: Front Mol Biosci. 2024 Oct 16;11:1430794. doi: 10.3389/fmolb.2024.1430794 (PMC11521808; doi:10.3389/fmolb.2024.1430794)
Supplement: Supplementary file 1 [file DataSheet1.zip › Supplementary File 5.PDF]

MOLECULAR Data:
Accuracy : 1) 0.9500, 2) 0.9831, 3) 1.0000. Average: 0.9777, STD: 0.0208.
Precision : 1) 0.9375, 2) 1.0000, 3) 1.0000. Average: 0.9792, STD: 0.0295.
Recall : 1) 1.0000, 2) 0.9787, 3) 1.0000. Average: 0.9929, STD: 0.0100.
Specificity: 1) 0.8000, 2) 1.0000, 3) 1.0000. Average: 0.9333, STD: 0.0943.
MCC : 1) 0.8660, 2) 0.9505, 3) 1.0000. Average: 0.9388, STD: 0.0553.
Reduced Model with SFS:
Accuracy : 1) 0.9667, 2) 0.9831, 3) 0.9831. Average: 0.9776, STD: 0.0077.
Precision : 1) 0.9574, 2) 1.0000, 3) 1.0000. Average: 0.9858, STD: 0.0201.
Recall : 1) 1.0000, 2) 0.9787, 3) 0.9787. Average: 0.9858, STD: 0.0100.
Specificity: 1) 0.8667, 2) 1.0000, 3) 1.0000. Average: 0.9556, STD: 0.0629.
MCC : 1) 0.9109, 2) 0.9505, 3) 0.9505. Average: 0.9373, STD: 0.0187.
Features in: 2 features
miR-125b
TUBG mRNA
Features out: 12 features
lncRNA-RP11-513I15.6
miR-1289
lncRNA-RP11-583F2.2
miR-1262
BAX mRNA
Cyclin E mRNA
ATG16-L1
lncRNA-MALAT
P53 mRNA
RAB11 mRNA
miR-106b
circ_0001345

BIOCHEMICAL Data:
Accuracy : 1) 0.9500, 2) 0.9153, 3) 0.9322. Average: 0.9325, STD: 0.0142.
Precision : 1) 0.9375, 2) 0.9773, 3) 1.0000. Average: 0.9716, STD: 0.0258.
Recall : 1) 1.0000, 2) 0.9149, 3) 0.9149. Average: 0.9433, STD: 0.0401.
Specificity: 1) 0.8000, 2) 0.9167, 3) 1.0000. Average: 0.9056, STD: 0.0820.
MCC : 1) 0.8660, 2) 0.7687, 3) 0.8284. Average: 0.8210, STD: 0.0401.
Reduced Model with SFS:
Accuracy : 1) 0.9500, 2) 0.9831, 3) 0.9153. Average: 0.9494, STD: 0.0277.
Precision : 1) 0.9375, 2) 0.9792, 3) 1.0000. Average: 0.9722, STD: 0.0260.
Recall : 1) 1.0000, 2) 1.0000, 3) 0.8936. Average: 0.9645, STD: 0.0501.
Specificity: 1) 0.8000, 2) 0.9167, 3) 1.0000. Average: 0.9056, STD: 0.0820.
MCC : 1) 0.8660, 2) 0.9474, 3) 0.7942. Average: 0.8692, STD: 0.0626.
Features in: 2 features
ALT
TG
Features out: 10 features
AST
ALP
GGT
T.Bilirubin
D.Bilirubin
AFP
Albumin
TC
HDL-C
LDL-C

IHC Data:
Accuracy : 1) 0.9333, 2) 0.9492, 3) 0.8814. Average: 0.9213, STD: 0.0290.
Precision : 1) 0.9362, 2) 0.9783, 3) 1.0000. Average: 0.9715, STD: 0.0265.
Recall : 1) 0.9778, 2) 0.9574, 3) 0.8511. Average: 0.9288, STD: 0.0556.
Specificity: 1) 0.8000, 2) 0.9167, 3) 1.0000. Average: 0.9056, STD: 0.0820.
MCC : 1) 0.8175, 2) 0.8489, 3) 0.7332. Average: 0.7999, STD: 0.0489.
Reduced Model with SFS:
Accuracy : 1) 0.9333, 2) 0.9661, 3) 0.8305. Average: 0.9100, STD: 0.0578.
Precision : 1) 0.9362, 2) 0.9787, 3) 1.0000. Average: 0.9716, STD: 0.0265.
Recall : 1) 0.9778, 2) 0.9787, 3) 0.7872. Average: 0.9146, STD: 0.0900.
Specificity: 1) 0.8000, 2) 0.9167, 3) 1.0000. Average: 0.9056, STD: 0.0820.
MCC : 1) 0.8175, 2) 0.8954, 3) 0.6553. Average: 0.7894, STD: 0.1000.
Features in: 1 features
GSTP
Features out: 2 features
PCNA
TNF

MOLECULAR-BIOCHEMICAL Data:
Accuracy : 1) 0.9500, 2) 0.9831, 3) 1.0000. Average: 0.9777, STD: 0.0208.
Precision : 1) 0.9375, 2) 1.0000, 3) 1.0000. Average: 0.9792, STD: 0.0295.
Recall : 1) 1.0000, 2) 0.9787, 3) 1.0000. Average: 0.9929, STD: 0.0100.
Specificity: 1) 0.8000, 2) 1.0000, 3) 1.0000. Average: 0.9333, STD: 0.0943.
MCC : 1) 0.8660, 2) 0.9505, 3) 1.0000. Average: 0.9388, STD: 0.0553.
Reduced Model with SFS:
Accuracy : 1) 0.9667, 2) 0.9831, 3) 0.9831. Average: 0.9776, STD: 0.0077.
Precision : 1) 0.9574, 2) 1.0000, 3) 1.0000. Average: 0.9858, STD: 0.0201.
Recall : 1) 1.0000, 2) 0.9787, 3) 0.9787. Average: 0.9858, STD: 0.0100.
Specificity: 1) 0.8667, 2) 1.0000, 3) 1.0000. Average: 0.9556, STD: 0.0629.
MCC : 1) 0.9109, 2) 0.9505, 3) 0.9505. Average: 0.9373, STD: 0.0187.
Features in: 2 features
miR-125b
TUBG mRNA
Features out: 24 features
lncRNA-RP11-513I15.6
miR-1289
lncRNA-RP11-583F2.2
miR-1262
BAX mRNA
Cyclin E mRNA
ATG16-L1
lncRNA-MALAT
P53 mRNA
RAB11 mRNA
miR-106b
circ_0001345
ALT
AST
ALP
GGT
T.Bilirubin
D.Bilirubin
AFP
Albumin

TC
TG
HDL-C
LDL-C

MOLECULAR-IHC Data:
Accuracy : 1) 0.9500, 2) 0.9831, 3) 1.0000. Average: 0.9777, STD: 0.0208.
Precision : 1) 0.9375, 2) 1.0000, 3) 1.0000. Average: 0.9792, STD: 0.0295.
Recall : 1) 1.0000, 2) 0.9787, 3) 1.0000. Average: 0.9929, STD: 0.0100.
Specificity: 1) 0.8000, 2) 1.0000, 3) 1.0000. Average: 0.9333, STD: 0.0943.
MCC : 1) 0.8660, 2) 0.9505, 3) 1.0000. Average: 0.9388, STD: 0.0553.
Reduced Model with SFS:
Accuracy : 1) 0.9667, 2) 0.9831, 3) 0.9831. Average: 0.9776, STD: 0.0077.
Precision : 1) 0.9574, 2) 1.0000, 3) 1.0000. Average: 0.9858, STD: 0.0201.
Recall : 1) 1.0000, 2) 0.9787, 3) 0.9787. Average: 0.9858, STD: 0.0100.
Specificity: 1) 0.8667, 2) 1.0000, 3) 1.0000. Average: 0.9556, STD: 0.0629.
MCC : 1) 0.9109, 2) 0.9505, 3) 0.9505. Average: 0.9373, STD: 0.0187.
Features in: 2 features
miR-125b
TUBG mRNA
Features out: 15 features
lncRNA-RP11-513I15.6
miR-1289
lncRNA-RP11-583F2.2
miR-1262
BAX mRNA
Cyclin E mRNA
ATG16-L1
lncRNA-MALAT
P53 mRNA
RAB11 mRNA
miR-106b
circ_0001345
GSTP
PCNA
TNF

BIOCHEMICAL-IHC Data:
Accuracy : 1) 0.9500, 2) 0.9661, 3) 0.9831. Average: 0.9664, STD: 0.0135.
Precision : 1) 0.9375, 2) 0.9787, 3) 1.0000. Average: 0.9721, STD: 0.0259.
Recall : 1) 1.0000, 2) 0.9787, 3) 0.9787. Average: 0.9858, STD: 0.0100.
Specificity: 1) 0.8000, 2) 0.9167, 3) 1.0000. Average: 0.9056, STD: 0.0820.
MCC : 1) 0.8660, 2) 0.8954, 3) 0.9505. Average: 0.9040, STD: 0.0350.
Reduced Model with SFS:
Accuracy : 1) 0.9333, 2) 0.9492, 3) 0.9153. Average: 0.9326, STD: 0.0138.
Precision : 1) 0.9362, 2) 0.9783, 3) 1.0000. Average: 0.9715, STD: 0.0265.
Recall : 1) 0.9778, 2) 0.9574, 3) 0.8936. Average: 0.9429, STD: 0.0359.
Specificity: 1) 0.8000, 2) 0.9167, 3) 1.0000. Average: 0.9056, STD: 0.0820.
MCC : 1) 0.8175, 2) 0.8489, 3) 0.7942. Average: 0.8202, STD: 0.0224.
Features in: 2 features
TG
GSTP
Features out: 13 features
ALT
AST

ALP
GGT
T.Bilirubin
D.Bilirubin
AFP
Albumin
TC
HDL-C
LDL-C
PCNA
TNF

MOLECULAR-BIOCHEMICAL-IHC Data:
Accuracy : 1) 0.9500, 2) 0.9831, 3) 1.0000. Average: 0.9777, STD: 0.0208.
Precision : 1) 0.9375, 2) 1.0000, 3) 1.0000. Average: 0.9792, STD: 0.0295.
Recall : 1) 1.0000, 2) 0.9787, 3) 1.0000. Average: 0.9929, STD: 0.0100.
Specificity: 1) 0.8000, 2) 1.0000, 3) 1.0000. Average: 0.9333, STD: 0.0943.
MCC : 1) 0.8660, 2) 0.9505, 3) 1.0000. Average: 0.9388, STD: 0.0553.
Reduced Model with SFS:
Accuracy : 1) 0.9667, 2) 0.9831, 3) 0.9831. Average: 0.9776, STD: 0.0077.
Precision : 1) 0.9574, 2) 1.0000, 3) 1.0000. Average: 0.9858, STD: 0.0201.
Recall : 1) 1.0000, 2) 0.9787, 3) 0.9787. Average: 0.9858, STD: 0.0100.
Specificity: 1) 0.8667, 2) 1.0000, 3) 1.0000. Average: 0.9556, STD: 0.0629.
MCC : 1) 0.9109, 2) 0.9505, 3) 0.9505. Average: 0.9373, STD: 0.0187.
Features in: 2 features
miR-125b
TUBG mRNA
Features out: 27 features
lncRNA-RP11-513I15.6
miR-1289
lncRNA-RP11-583F2.2
miR-1262
BAX mRNA
Cyclin E mRNA
ATG16-L1
lncRNA-MALAT
P53 mRNA
RAB11 mRNA
miR-106b
circ_0001345
ALT
AST
ALP
GGT
T.Bilirubin
D.Bilirubin
AFP
Albumin
TC
TG
HDL-C
LDL-C
GSTP
PCNA
TNF
