# Supplementary material for: Integrating molecular, biochemical, and immunohistochemical features as predictors of hepatocellular carcinoma drug response using machine-learning algorithms
Source: Front Mol Biosci. 2024 Oct 16;11:1430794. doi: 10.3389/fmolb.2024.1430794 (PMC11521808; doi:10.3389/fmolb.2024.1430794)
Supplement: Supplementary file 1 [file DataSheet1.zip › Supplementary File 6.PDF]

MOLECULAR Data:
Accuracy : 1) 0.9500, 2) 0.9831, 3) 1.0000. Average: 0.9777, STD: 0.0208.
Precision : 1) 0.9375, 2) 0.9792, 3) 1.0000. Average: 0.9722, STD: 0.0260.
Recall : 1) 1.0000, 2) 1.0000, 3) 1.0000. Average: 1.0000, STD: 0.0000.
Specificity: 1) 0.8000, 2) 0.9167, 3) 1.0000. Average: 0.9056, STD: 0.0820.
MCC : 1) 0.8660, 2) 0.9474, 3) 1.0000. Average: 0.9378, STD: 0.0551.
Reduced Model with SFS:
Accuracy : 1) 0.9500, 2) 0.9831, 3) 1.0000. Average: 0.9777, STD: 0.0208.
Precision : 1) 0.9375, 2) 0.9792, 3) 1.0000. Average: 0.9722, STD: 0.0260.
Recall : 1) 1.0000, 2) 1.0000, 3) 1.0000. Average: 1.0000, STD: 0.0000.
Specificity: 1) 0.8000, 2) 0.9167, 3) 1.0000. Average: 0.9056, STD: 0.0820.
MCC : 1) 0.8660, 2) 0.9474, 3) 1.0000. Average: 0.9378, STD: 0.0551.
Features in: 2 features
miR-125b
TUBG mRNA
Features out: 12 features
lncRNA-RP11-513I15.6
miR-1289
lncRNA-RP11-583F2.2
miR-1262
BAX mRNA
Cyclin E mRNA
ATG16-L1
lncRNA-MALAT
P53 mRNA
RAB11 mRNA
miR-106b
circ_0001345

BIOCHEMICAL Data:
Accuracy : 1) 0.9500, 2) 0.9831, 3) 1.0000. Average: 0.9777, STD: 0.0208.
Precision : 1) 0.9375, 2) 0.9792, 3) 1.0000. Average: 0.9722, STD: 0.0260.
Recall : 1) 1.0000, 2) 1.0000, 3) 1.0000. Average: 1.0000, STD: 0.0000.
Specificity: 1) 0.8000, 2) 0.9167, 3) 1.0000. Average: 0.9056, STD: 0.0820.
MCC : 1) 0.8660, 2) 0.9474, 3) 1.0000. Average: 0.9378, STD: 0.0551.
Reduced Model with SFS:
Accuracy : 1) 0.9500, 2) 0.9322, 3) 1.0000. Average: 0.9607, STD: 0.0287.
Precision : 1) 0.9375, 2) 0.9778, 3) 1.0000. Average: 0.9718, STD: 0.0259.
Recall : 1) 1.0000, 2) 0.9362, 3) 1.0000. Average: 0.9787, STD: 0.0301.
Specificity: 1) 0.8000, 2) 0.9167, 3) 1.0000. Average: 0.9056, STD: 0.0820.
MCC : 1) 0.8660, 2) 0.8069, 3) 1.0000. Average: 0.8910, STD: 0.0808.
Features in: 2 features
ALT
TG
Features out: 10 features
AST
ALP
GGT
T.Bilirubin
D.Bilirubin
AFP
Albumin
TC
HDL-C
LDL-C

IHC Data:
 Accuracy   : 1) 0.9500, 2) 0.9831, 3) 0.9831. Average: 0.9720, STD: 0.0156.
 Precision : 1) 0.9375, 2) 0.9792, 3) 1.0000. Average: 0.9722, STD: 0.0260.
 Recall   : 1) 1.0000, 2) 1.0000, 3) 0.9787. Average: 0.9929, STD: 0.0100.
 Specificity: 1) 0.8000, 2) 0.9167, 3) 1.0000. Average: 0.9056, STD: 0.0820.
 MCC   : 1) 0.8660, 2) 0.9474, 3) 0.9505. Average: 0.9213, STD: 0.0391.
 Reduced Model with SFS:
   Accuracy   : 1) 0.9500, 2) 0.9661, 3) 0.9661. Average: 0.9607, STD: 0.0076.
   Precision : 1) 0.9375, 2) 0.9787, 3) 1.0000. Average: 0.9721, STD: 0.0259.
   Recall   : 1) 1.0000, 2) 0.9787, 3) 0.9574. Average: 0.9787, STD: 0.0174.
   Specificity: 1) 0.8000, 2) 0.9167, 3) 1.0000. Average: 0.9056, STD: 0.0820.
   MCC   : 1) 0.8660, 2) 0.8954, 3) 0.9059. Average: 0.8891, STD: 0.0169.
   Features in: 1 features
     GSTP
   Features out: 2 features
     PCNA
     TNF

MOLECULAR-BIOCHEMICAL Data:
 Accuracy   : 1) 0.9500, 2) 0.9831, 3) 1.0000. Average: 0.9777, STD: 0.0208.
 Precision : 1) 0.9375, 2) 0.9792, 3) 1.0000. Average: 0.9722, STD: 0.0260.
 Recall   : 1) 1.0000, 2) 1.0000, 3) 1.0000. Average: 1.0000, STD: 0.0000.
 Specificity: 1) 0.8000, 2) 0.9167, 3) 1.0000. Average: 0.9056, STD: 0.0820.
 MCC   : 1) 0.8660, 2) 0.9474, 3) 1.0000. Average: 0.9378, STD: 0.0551.
 Reduced Model with SFS:
   Accuracy   : 1) 0.9500, 2) 0.9831, 3) 1.0000. Average: 0.9777, STD: 0.0208.
   Precision : 1) 0.9375, 2) 0.9792, 3) 1.0000. Average: 0.9722, STD: 0.0260.
   Recall   : 1) 1.0000, 2) 1.0000, 3) 1.0000. Average: 1.0000, STD: 0.0000.
   Specificity: 1) 0.8000, 2) 0.9167, 3) 1.0000. Average: 0.9056, STD: 0.0820.
   MCC   : 1) 0.8660, 2) 0.9474, 3) 1.0000. Average: 0.9378, STD: 0.0551.
   Features in: 2 features
     miR-125b
     TUBG mRNA
   Features out: 24 features
     lncRNA-RP11-513I15.6
     miR-1289
     lncRNA-RP11-583F2.2
     miR-1262
     BAX mRNA
     Cyclin E mRNA
     ATG16-L1
     lncRNA-MALAT
     P53 mRNA
     RAB11 mRNA
     miR-106b
     circ_0001345
     ALT
     AST
     ALP
     GGT
     T.Bilirubin
     D.Bilirubin
     AFP
     Albumin

TC
TG
HDL-C
LDL-C

MOLECULAR-IHC Data:
Accuracy   : 1) 0.9500, 2) 0.9831, 3) 1.0000. Average: 0.9777, STD: 0.0208.
Precision : 1) 0.9375, 2) 0.9792, 3) 1.0000. Average: 0.9722, STD: 0.0260.
Recall   : 1) 1.0000, 2) 1.0000, 3) 1.0000. Average: 1.0000, STD: 0.0000.
Specificity: 1) 0.8000, 2) 0.9167, 3) 1.0000. Average: 0.9056, STD: 0.0820.
MCC   : 1) 0.8660, 2) 0.9474, 3) 1.0000. Average: 0.9378, STD: 0.0551.
Reduced Model with SFS:
Accuracy   : 1) 0.9500, 2) 0.9831, 3) 1.0000. Average: 0.9777, STD: 0.0208.
Precision : 1) 0.9375, 2) 0.9792, 3) 1.0000. Average: 0.9722, STD: 0.0260.
Recall   : 1) 1.0000, 2) 1.0000, 3) 1.0000. Average: 1.0000, STD: 0.0000.
Specificity: 1) 0.8000, 2) 0.9167, 3) 1.0000. Average: 0.9056, STD: 0.0820.
MCC   : 1) 0.8660, 2) 0.9474, 3) 1.0000. Average: 0.9378, STD: 0.0551.
Features in: 2 features
miR-125b
TUBG mRNA
Features out: 15 features
lncRNA-RP11-513I15.6
miR-1289
lncRNA-RP11-583F2.2
miR-1262
BAX mRNA
Cyclin E mRNA
ATG16-L1
lncRNA-MALAT
P53 mRNA
RAB11 mRNA
miR-106b
circ_0001345
GSTP
PCNA
TNF

BIOCHEMICAL-IHC Data:
Accuracy   : 1) 0.9500, 2) 0.9831, 3) 1.0000. Average: 0.9777, STD: 0.0208.
Precision : 1) 0.9375, 2) 0.9792, 3) 1.0000. Average: 0.9722, STD: 0.0260.
Recall   : 1) 1.0000, 2) 1.0000, 3) 1.0000. Average: 1.0000, STD: 0.0000.
Specificity: 1) 0.8000, 2) 0.9167, 3) 1.0000. Average: 0.9056, STD: 0.0820.
MCC   : 1) 0.8660, 2) 0.9474, 3) 1.0000. Average: 0.9378, STD: 0.0551.
Reduced Model with SFS:
Accuracy   : 1) 0.9500, 2) 0.9661, 3) 0.9831. Average: 0.9664, STD: 0.0135.
Precision : 1) 0.9375, 2) 0.9787, 3) 1.0000. Average: 0.9721, STD: 0.0259.
Recall   : 1) 1.0000, 2) 0.9787, 3) 0.9787. Average: 0.9858, STD: 0.0100.
Specificity: 1) 0.8000, 2) 0.9167, 3) 1.0000. Average: 0.9056, STD: 0.0820.
MCC   : 1) 0.8660, 2) 0.8954, 3) 0.9505. Average: 0.9040, STD: 0.0350.
Features in: 2 features
TG
GSTP
Features out: 13 features
ALT
AST

ALP
GGT
T.Bilirubin
D.Bilirubin
AFP
Albumin
TC
HDL-C
LDL-C
PCNA
TNF

MOLECULAR-BIOCHEMICAL-IHC Data:
Accuracy : 1) 0.9500, 2) 0.9831, 3) 1.0000. Average: 0.9777, STD: 0.0208.
Precision : 1) 0.9375, 2) 0.9792, 3) 1.0000. Average: 0.9722, STD: 0.0260.
Recall : 1) 1.0000, 2) 1.0000, 3) 1.0000. Average: 1.0000, STD: 0.0000.
Specificity: 1) 0.8000, 2) 0.9167, 3) 1.0000. Average: 0.9056, STD: 0.0820.
MCC : 1) 0.8660, 2) 0.9474, 3) 1.0000. Average: 0.9378, STD: 0.0551.
Reduced Model with SFS:
Accuracy : 1) 0.9500, 2) 0.9831, 3) 1.0000. Average: 0.9777, STD: 0.0208.
Precision : 1) 0.9375, 2) 0.9792, 3) 1.0000. Average: 0.9722, STD: 0.0260.
Recall : 1) 1.0000, 2) 1.0000, 3) 1.0000. Average: 1.0000, STD: 0.0000.
Specificity: 1) 0.8000, 2) 0.9167, 3) 1.0000. Average: 0.9056, STD: 0.0820.
MCC : 1) 0.8660, 2) 0.9474, 3) 1.0000. Average: 0.9378, STD: 0.0551.
Features in: 2 features
miR-125b
TUBG mRNA
Features out: 27 features
lncRNA-RP11-513I15.6
miR-1289
lncRNA-RP11-583F2.2
miR-1262
BAX mRNA
Cyclin E mRNA
ATG16-L1
lncRNA-MALAT
P53 mRNA
RAB11 mRNA
miR-106b
circ_0001345
ALT
AST
ALP
GGT
T.Bilirubin
D.Bilirubin
AFP
Albumin
TC
TG
HDL-C
LDL-C
GSTP
PCNA
TNF
