# Supplementary material for: Integrating molecular, biochemical, and immunohistochemical features as predictors of hepatocellular carcinoma drug response using machine-learning algorithms
Source: Front Mol Biosci. 2024 Oct 16;11:1430794. doi: 10.3389/fmolb.2024.1430794 (PMC11521808; doi:10.3389/fmolb.2024.1430794)
Supplement: Supplementary file 1 [file DataSheet1.zip › Supplementary File 2.PDF]

```
MOLECULAR Data:
      Accuracy   : 1) 0.9667, 2) 0.9831, 3) 0.9831. Average: 0.9776, STD:
0.0077.
      Precision  : 1) 0.9574, 2) 0.9792, 3) 1.0000. Average: 0.9789, STD:
0.0174.
      Recall     : 1) 1.0000, 2) 1.0000, 3) 0.9787. Average: 0.9929, STD:
0.0100.
      Specificity: 1) 0.8667, 2) 0.9167, 3) 1.0000. Average: 0.9278, STD:
0.0550.
      MCC        : 1) 0.9109, 2) 0.9474, 3) 0.9505. Average: 0.9363, STD:
0.0180.
      Reduced Model with SFS:
                  Accuracy   : 1) 0.9500, 2) 0.9831, 3) 0.9831. Average:
0.9720, STD: 0.0156.
                  Precision  : 1) 0.9375, 2) 1.0000, 3) 1.0000. Average:
0.9792, STD: 0.0295.
                  Recall     : 1) 1.0000, 2) 0.9787, 3) 0.9787. Average:
0.9858, STD: 0.0100.
                  Specificity: 1) 0.8000, 2) 1.0000, 3) 1.0000. Average:
0.9333, STD: 0.0943.
                  MCC        : 1) 0.8660, 2) 0.9505, 3) 0.9505. Average:
0.9223, STD: 0.0398.
                  Features in: 2 features
                              miR-125b
                              TUBG mRNA
                  Features out: 12 features
                              lncRNA-RP11-513I15.6
                              miR-1289
                              lncRNA-RP11-583F2.2
                              miR-1262
                              BAX mRNA
                              Cyclin E mRNA
                              ATG16-L1
                              lncRNA-MALAT
                              P53 mRNA
                              RAB11 mRNA
                              miR-106b
                              circ_0001345

BIOCHEMICAL Data:
      Accuracy   : 1) 0.9167, 2) 0.8983, 3) 0.9322. Average: 0.9157, STD:
0.0139.
      Precision  : 1) 0.9348, 2) 0.9767, 3) 1.0000. Average: 0.9705, STD:
0.0270.
      Recall     : 1) 0.9556, 2) 0.8936, 3) 0.9149. Average: 0.9214, STD:
0.0257.
      Specificity: 1) 0.8000, 2) 0.9167, 3) 1.0000. Average: 0.9056, STD:
0.0820.
      MCC        : 1) 0.7735, 2) 0.7336, 3) 0.8284. Average: 0.7785, STD:
0.0388.
      Reduced Model with SFS:
                  Accuracy   : 1) 0.9500, 2) 0.9831, 3) 0.9492. Average:
0.9607, STD: 0.0158.
```

```
                 Precision  : 1) 0.9375, 2) 0.9792, 3) 0.9783. Average: 
0.9650, STD: 0.0194.
                 Recall     : 1) 1.0000, 2) 1.0000, 3) 0.9574. Average: 
0.9858, STD: 0.0201.
                 Specificity: 1) 0.8000, 2) 0.9167, 3) 0.9167. Average: 
0.8778, STD: 0.0550.
                 MCC        : 1) 0.8660, 2) 0.9474, 3) 0.8489. Average: 
0.8874, STD: 0.0430.
                 Features in: 2 features
                            ALT
                            TG
                 Features out: 10 features
                            AST
                            ALP
                            GGT
                            T.Bilirubin
                            D.Bilirubin
                            AFP
                            Albumin
                            TC
                            HDL-C
                            LDL-C

IHC Data:
      Accuracy   : 1) 0.9333, 2) 0.9153, 3) 0.9153. Average: 0.9213, STD: 
0.0085.
      Precision  : 1) 0.9362, 2) 0.9773, 3) 1.0000. Average: 0.9711, STD: 
0.0264.
      Recall     : 1) 0.9778, 2) 0.9149, 3) 0.8936. Average: 0.9288, STD: 
0.0357.
      Specificity: 1) 0.8000, 2) 0.9167, 3) 1.0000. Average: 0.9056, STD: 
0.0820.
      MCC        : 1) 0.8175, 2) 0.7687, 3) 0.7942. Average: 0.7935, STD: 
0.0199.
      Reduced Model with SFS:
                 Accuracy   : 1) 0.9500, 2) 0.9661, 3) 0.8644. Average: 
0.9268, STD: 0.0446.
                 Precision  : 1) 0.9375, 2) 0.9787, 3) 1.0000. Average: 
0.9721, STD: 0.0259.
                 Recall     : 1) 1.0000, 2) 0.9787, 3) 0.8298. Average: 
0.9362, STD: 0.0757.
                 Specificity: 1) 0.8000, 2) 0.9167, 3) 1.0000. Average: 
0.9056, STD: 0.0820.
                 MCC        : 1) 0.8660, 2) 0.8954, 3) 0.7056. Average: 
0.8223, STD: 0.0834.
                 Features in: 1 features
                            GSTP
                 Features out: 2 features
                            PCNA
                            TNF

MOLECULAR-BIOCHEMICAL Data:
      Accuracy   : 1) 0.9667, 2) 0.9661, 3) 0.9661. Average: 0.9663, STD: 
0.0003.
```

Precision : 1) 0.9574, 2) 1.0000, 3) 1.0000. Average: 0.9858, STD: 0.0201.

Recall : 1) 1.0000, 2) 0.9574, 3) 0.9574. Average: 0.9716, STD: 0.0201.

Specificity: 1) 0.8667, 2) 1.0000, 3) 1.0000. Average: 0.9556, STD: 0.0629.

MCC : 1) 0.9109, 2) 0.9059, 3) 0.9059. Average: 0.9076, STD: 0.0024.

Reduced Model with SFS:

Accuracy : 1) 0.9500, 2) 0.9831, 3) 0.9831. Average: 0.9720, STD: 0.0156.

Precision : 1) 0.9375, 2) 1.0000, 3) 1.0000. Average: 0.9792, STD: 0.0295.

Recall : 1) 1.0000, 2) 0.9787, 3) 0.9787. Average: 0.9858, STD: 0.0100.

Specificity: 1) 0.8000, 2) 1.0000, 3) 1.0000. Average: 0.9333, STD: 0.0943.

MCC : 1) 0.8660, 2) 0.9505, 3) 0.9505. Average: 0.9223, STD: 0.0398.

Features in: 2 features

miR-125b
TUBG mRNA

Features out: 24 features

lncRNA-RP11-513I15.6
miR-1289
lncRNA-RP11-583F2.2
miR-1262
BAX mRNA
Cyclin E mRNA
ATG16-L1
lncRNA-MALAT
P53 mRNA
RAB11 mRNA
miR-106b
circ_0001345
ALT
AST
ALP
GGT
T.Bilirubin
D.Bilirubin
AFP
Albumin
TC
TG
HDL-C
LDL-C

MOLECULAR-IHC Data:

Accuracy : 1) 0.9667, 2) 0.9661, 3) 0.9661. Average: 0.9663, STD: 0.0003.

Precision : 1) 0.9574, 2) 0.9787, 3) 1.0000. Average: 0.9787, STD: 0.0174.

Recall : 1) 1.0000, 2) 0.9787, 3) 0.9574. Average: 0.9787, STD: 0.0174.

Specificity: 1) 0.8667, 2) 0.9167, 3) 1.0000. Average: 0.9278, STD: 0.0550.

MCC : 1) 0.9109, 2) 0.8954, 3) 0.9059. Average: 0.9041, STD: 0.0065.

Reduced Model with SFS:

Accuracy : 1) 0.9500, 2) 0.9831, 3) 0.9831. Average: 0.9720, STD: 0.0156.

Precision : 1) 0.9375, 2) 1.0000, 3) 1.0000. Average: 0.9792, STD: 0.0295.

Recall : 1) 1.0000, 2) 0.9787, 3) 0.9787. Average: 0.9858, STD: 0.0100.

Specificity: 1) 0.8000, 2) 1.0000, 3) 1.0000. Average: 0.9333, STD: 0.0943.

MCC : 1) 0.8660, 2) 0.9505, 3) 0.9505. Average: 0.9223, STD: 0.0398.

Features in: 2 features

miR-125b
TUBG mRNA

Features out: 15 features

lncRNA-RP11-513I15.6
miR-1289
lncRNA-RP11-583F2.2
miR-1262
BAX mRNA
Cyclin E mRNA
ATG16-L1
lncRNA-MALAT
P53 mRNA
RAB11 mRNA
miR-106b
circ_0001345
GSTP
PCNA
TNF

BIOCHEMICAL-IHC Data:

Accuracy : 1) 0.9333, 2) 0.9492, 3) 0.9661. Average: 0.9495, STD: 0.0134.

Precision : 1) 0.9362, 2) 1.0000, 3) 1.0000. Average: 0.9787, STD: 0.0301.

Recall : 1) 0.9778, 2) 0.9362, 3) 0.9574. Average: 0.9571, STD: 0.0170.

Specificity: 1) 0.8000, 2) 1.0000, 3) 1.0000. Average: 0.9333, STD: 0.0943.

MCC : 1) 0.8175, 2) 0.8654, 3) 0.9059. Average: 0.8629, STD: 0.0361.

Reduced Model with SFS:

Accuracy : 1) 0.9500, 2) 0.9661, 3) 0.8814. Average: 0.9325, STD: 0.0367.

Precision : 1) 0.9375, 2) 0.9787, 3) 1.0000. Average: 0.9721, STD: 0.0259.

Recall : 1) 1.0000, 2) 0.9787, 3) 0.8511. Average: 0.9433, STD: 0.0658.
Specificity: 1) 0.8000, 2) 0.9167, 3) 1.0000. Average: 0.9056, STD: 0.0820.
MCC : 1) 0.8660, 2) 0.8954, 3) 0.7332. Average: 0.8315, STD: 0.0706.
Features in: 2 features
TG
GSTP
Features out: 13 features
ALT
AST
ALP
GGT
T.Bilirubin
D.Bilirubin
AFP
Albumin
TC
HDL-C
LDL-C
PCNA
TNF

MOLECULAR-BIOCHEMICAL-IHC Data:
Accuracy : 1) 0.9667, 2) 0.9831, 3) 0.9492. Average: 0.9663, STD: 0.0138.
Precision : 1) 0.9574, 2) 1.0000, 3) 1.0000. Average: 0.9858, STD: 0.0201.
Recall : 1) 1.0000, 2) 0.9787, 3) 0.9362. Average: 0.9716, STD: 0.0265.
Specificity: 1) 0.8667, 2) 1.0000, 3) 1.0000. Average: 0.9556, STD: 0.0629.
MCC : 1) 0.9109, 2) 0.9505, 3) 0.8654. Average: 0.9089, STD: 0.0348.
Reduced Model with SFS:
Accuracy : 1) 0.9500, 2) 0.9831, 3) 0.9831. Average: 0.9720, STD: 0.0156.
Precision : 1) 0.9375, 2) 1.0000, 3) 1.0000. Average: 0.9792, STD: 0.0295.
Recall : 1) 1.0000, 2) 0.9787, 3) 0.9787. Average: 0.9858, STD: 0.0100.
Specificity: 1) 0.8000, 2) 1.0000, 3) 1.0000. Average: 0.9333, STD: 0.0943.
MCC : 1) 0.8660, 2) 0.9505, 3) 0.9505. Average: 0.9223, STD: 0.0398.
Features in: 2 features
miR-125b
TUBG mRNA
Features out: 27 features
lncRNA-RP11-513I15.6
miR-1289
lncRNA-RP11-583F2.2
miR-1262

BAX mRNA
Cyclin E mRNA
ATG16-L1
lncRNA-MALAT
P53 mRNA
RAB11 mRNA
miR-106b
circ_0001345
ALT
AST
ALP
GGT
T.Bilirubin
D.Bilirubin
AFP
Albumin
TC
TG
HDL-C
LDL-C
GSTP
PCNA
TNF
