# Supplementary material for: Integrating molecular, biochemical, and immunohistochemical features as predictors of hepatocellular carcinoma drug response using machine-learning algorithms
Source: Front Mol Biosci. 2024 Oct 16;11:1430794. doi: 10.3389/fmolb.2024.1430794 (PMC11521808; doi:10.3389/fmolb.2024.1430794)
Supplement: Supplementary file 1 [file DataSheet1.zip › Supplementary File 3.PDF]

```
MOLECULAR Data:
      Accuracy   : 1) 0.9500, 2) 0.9661, 3) 0.9831. Average: 0.9664, STD:
0.0135.
      Precision  : 1) 0.9375, 2) 1.0000, 3) 1.0000. Average: 0.9792, STD:
0.0295.
      Recall     : 1) 1.0000, 2) 0.9574, 3) 0.9787. Average: 0.9787, STD:
0.0174.
      Specificity: 1) 0.8000, 2) 1.0000, 3) 1.0000. Average: 0.9333, STD:
0.0943.
      MCC        : 1) 0.8660, 2) 0.9059, 3) 0.9505. Average: 0.9075, STD:
0.0345.
      Reduced Model with SFS:
                  Accuracy   : 1) 0.9500, 2) 0.9831, 3) 0.9661. Average:
0.9664, STD: 0.0135.
                  Precision  : 1) 0.9375, 2) 0.9792, 3) 1.0000. Average:
0.9722, STD: 0.0260.
                  Recall     : 1) 1.0000, 2) 1.0000, 3) 0.9574. Average:
0.9858, STD: 0.0201.
                  Specificity: 1) 0.8000, 2) 0.9167, 3) 1.0000. Average:
0.9056, STD: 0.0820.
                  MCC        : 1) 0.8660, 2) 0.9474, 3) 0.9059. Average:
0.9064, STD: 0.0332.
                  Features in: 2 features
                              miR-125b
                              TUBG mRNA
                  Features out: 12 features
                              lncRNA-RP11-513I15.6
                              miR-1289
                              lncRNA-RP11-583F2.2
                              miR-1262
                              BAX mRNA
                              Cyclin E mRNA
                              ATG16-L1
                              lncRNA-MALAT
                              P53 mRNA
                              RAB11 mRNA
                              miR-106b
                              circ_0001345

BIOCHEMICAL Data:
      Accuracy   : 1) 0.8500, 2) 0.9322, 3) 0.8983. Average: 0.8935, STD:
0.0337.
      Precision  : 1) 0.8462, 2) 0.9778, 3) 0.9362. Average: 0.9200, STD:
0.0549.
      Recall     : 1) 0.9778, 2) 0.9362, 3) 0.9362. Average: 0.9500, STD:
0.0196.
      Specificity: 1) 0.4667, 2) 0.9167, 3) 0.7500. Average: 0.7111, STD:
0.1858.
      MCC        : 1) 0.5661, 2) 0.8069, 3) 0.6862. Average: 0.6864, STD:
0.0983.
      Reduced Model with SFS:
                  Accuracy   : 1) 0.9500, 2) 0.9322, 3) 0.9492. Average:
0.9438, STD: 0.0082.
```

Precision : 1) 0.9375, 2) 0.9778, 3) 1.0000. Average: 0.9718, STD: 0.0259.
Recall : 1) 1.0000, 2) 0.9362, 3) 0.9362. Average: 0.9574, STD: 0.0301.
Specificity: 1) 0.8000, 2) 0.9167, 3) 1.0000. Average: 0.9056, STD: 0.0820.
MCC : 1) 0.8660, 2) 0.8069, 3) 0.8654. Average: 0.8461, STD: 0.0277.
Features in: 2 features
ALT
TG
Features out: 10 features
AST
ALP
GGT
T.Bilirubin
D.Bilirubin
AFP
Albumin
TC
HDL-C
LDL-C

IHC Data:
Accuracy : 1) 0.9500, 2) 0.9661, 3) 0.9322. Average: 0.9494, STD: 0.0138.
Precision : 1) 0.9375, 2) 1.0000, 3) 1.0000. Average: 0.9792, STD: 0.0295.
Recall : 1) 1.0000, 2) 0.9574, 3) 0.9149. Average: 0.9574, STD: 0.0347.
Specificity: 1) 0.8000, 2) 1.0000, 3) 1.0000. Average: 0.9333, STD: 0.0943.
MCC : 1) 0.8660, 2) 0.9059, 3) 0.8284. Average: 0.8668, STD: 0.0317.
Reduced Model with SFS:
Accuracy : 1) 0.9500, 2) 0.9661, 3) 0.9661. Average: 0.9607, STD: 0.0076.
Precision : 1) 0.9375, 2) 0.9787, 3) 1.0000. Average: 0.9721, STD: 0.0259.
Recall : 1) 1.0000, 2) 0.9787, 3) 0.9574. Average: 0.9787, STD: 0.0174.
Specificity: 1) 0.8000, 2) 0.9167, 3) 1.0000. Average: 0.9056, STD: 0.0820.
MCC : 1) 0.8660, 2) 0.8954, 3) 0.9059. Average: 0.8891, STD: 0.0169.
Features in: 1 features
GSTP
Features out: 2 features
PCNA
TNF

MOLECULAR-BIOCHEMICAL Data:
Accuracy : 1) 0.9167, 2) 0.9492, 3) 0.9831. Average: 0.9496, STD: 0.0271.

Precision : 1) 0.9000, 2) 0.9783, 3) 1.0000. Average: 0.9594, STD: 0.0429.
Recall   : 1) 1.0000, 2) 0.9574, 3) 0.9787. Average: 0.9787, STD: 0.0174.
Specificity: 1) 0.6667, 2) 0.9167, 3) 1.0000. Average: 0.8611, STD: 0.1416.
MCC   : 1) 0.7746, 2) 0.8489, 3) 0.9505. Average: 0.8580, STD: 0.0721.
Reduced Model with SFS:
Accuracy   : 1) 0.9500, 2) 0.9831, 3) 0.9661. Average: 0.9664, STD: 0.0135.
Precision : 1) 0.9375, 2) 0.9792, 3) 1.0000. Average: 0.9722, STD: 0.0260.
Recall   : 1) 1.0000, 2) 1.0000, 3) 0.9574. Average: 0.9858, STD: 0.0201.
Specificity: 1) 0.8000, 2) 0.9167, 3) 1.0000. Average: 0.9056, STD: 0.0820.
MCC   : 1) 0.8660, 2) 0.9474, 3) 0.9059. Average: 0.9064, STD: 0.0332.
Features in: 2 features
miR-125b
TUBG mRNA
Features out: 24 features
lncRNA-RP11-513I15.6
miR-1289
lncRNA-RP11-583F2.2
miR-1262
BAX mRNA
Cyclin E mRNA
ATG16-L1
lncRNA-MALAT
P53 mRNA
RAB11 mRNA
miR-106b
circ_0001345
ALT
AST
ALP
GGT
T.Bilirubin
D.Bilirubin
AFP
Albumin
TC
TG
HDL-C
LDL-C

MOLECULAR-IHC Data:
Accuracy   : 1) 0.9500, 2) 0.9661, 3) 0.9831. Average: 0.9664, STD: 0.0135.
Precision : 1) 0.9375, 2) 1.0000, 3) 1.0000. Average: 0.9792, STD: 0.0295.

Recall : 1) 1.0000, 2) 0.9574, 3) 0.9787. Average: 0.9787, STD: 0.0174.
Specificity: 1) 0.8000, 2) 1.0000, 3) 1.0000. Average: 0.9333, STD: 0.0943.
MCC : 1) 0.8660, 2) 0.9059, 3) 0.9505. Average: 0.9075, STD: 0.0345.
Reduced Model with SFS:
Accuracy : 1) 0.9500, 2) 0.9831, 3) 0.9661. Average: 0.9664, STD: 0.0135.
Precision : 1) 0.9375, 2) 0.9792, 3) 1.0000. Average: 0.9722, STD: 0.0260.
Recall : 1) 1.0000, 2) 1.0000, 3) 0.9574. Average: 0.9858, STD: 0.0201.
Specificity: 1) 0.8000, 2) 0.9167, 3) 1.0000. Average: 0.9056, STD: 0.0820.
MCC : 1) 0.8660, 2) 0.9474, 3) 0.9059. Average: 0.9064, STD: 0.0332.
Features in: 2 features
miR-125b
TUBG mRNA
Features out: 15 features
lncRNA-RP11-513I15.6
miR-1289
lncRNA-RP11-583F2.2
miR-1262
BAX mRNA
Cyclin E mRNA
ATG16-L1
lncRNA-MALAT
P53 mRNA
RAB11 mRNA
miR-106b
circ_0001345
GSTP
PCNA
TNF

BIOCHEMICAL-IHC Data:
Accuracy : 1) 0.9000, 2) 0.9322, 3) 0.9322. Average: 0.9215, STD: 0.0152.
Precision : 1) 0.8824, 2) 0.9778, 3) 0.9388. Average: 0.9330, STD: 0.0392.
Recall : 1) 1.0000, 2) 0.9362, 3) 0.9787. Average: 0.9716, STD: 0.0265.
Specificity: 1) 0.6000, 2) 0.9167, 3) 0.7500. Average: 0.7556, STD: 0.1293.
MCC : 1) 0.7276, 2) 0.8069, 3) 0.7818. Average: 0.7721, STD: 0.0331.
Reduced Model with SFS:
Accuracy : 1) 0.9500, 2) 0.9661, 3) 0.9661. Average: 0.9607, STD: 0.0076.
Precision : 1) 0.9375, 2) 0.9787, 3) 1.0000. Average: 0.9721, STD: 0.0259.

Recall : 1) 1.0000, 2) 0.9787, 3) 0.9574. Average: 0.9787, STD: 0.0174.
Specificity: 1) 0.8000, 2) 0.9167, 3) 1.0000. Average: 0.9056, STD: 0.0820.
MCC : 1) 0.8660, 2) 0.8954, 3) 0.9059. Average: 0.8891, STD: 0.0169.
Features in: 2 features
TG
GSTP
Features out: 13 features
ALT
AST
ALP
GGT
T.Bilirubin
D.Bilirubin
AFP
Albumin
TC
HDL-C
LDL-C
PCNA
TNF

MOLECULAR-BIOCHEMICAL-IHC Data:
Accuracy : 1) 0.9000, 2) 0.9492, 3) 0.9831. Average: 0.9441, STD: 0.0341.
Precision : 1) 0.8824, 2) 0.9783, 3) 1.0000. Average: 0.9535, STD: 0.0511.
Recall : 1) 1.0000, 2) 0.9574, 3) 0.9787. Average: 0.9787, STD: 0.0174.
Specificity: 1) 0.6000, 2) 0.9167, 3) 1.0000. Average: 0.8389, STD: 0.1723.
MCC : 1) 0.7276, 2) 0.8489, 3) 0.9505. Average: 0.8423, STD: 0.0911.
Reduced Model with SFS:
Accuracy : 1) 0.9500, 2) 0.9831, 3) 0.9661. Average: 0.9664, STD: 0.0135.
Precision : 1) 0.9375, 2) 0.9792, 3) 1.0000. Average: 0.9722, STD: 0.0260.
Recall : 1) 1.0000, 2) 1.0000, 3) 0.9574. Average: 0.9858, STD: 0.0201.
Specificity: 1) 0.8000, 2) 0.9167, 3) 1.0000. Average: 0.9056, STD: 0.0820.
MCC : 1) 0.8660, 2) 0.9474, 3) 0.9059. Average: 0.9064, STD: 0.0332.
Features in: 2 features
miR-125b
TUBG mRNA
Features out: 27 features
lncRNA-RP11-513I15.6
miR-1289
lncRNA-RP11-583F2.2
miR-1262

BAX mRNA
Cyclin E mRNA
ATG16-L1
lncRNA-MALAT
P53 mRNA
RAB11 mRNA
miR-106b
circ_0001345
ALT
AST
ALP
GGT
T.Bilirubin
D.Bilirubin
AFP
Albumin
TC
TG
HDL-C
LDL-C
GSTP
PCNA
TNF
